# Extracting Trends From NMR Data With TrAGICo: A Python Toolbox

**DOI:** 10.1002/mrc.5537

**Published:** 2025-06-12

**Authors:** Letizia Fiorucci, Francesco Bruno, Leonardo Querci, Adam Kubrak, Jlenia Bindi, Nebojša Rodić, Giulia Licciardi, Enrico Luchinat, Giacomo Parigi, Mario Piccioli, Enrico Ravera

**Affiliations:** ^1^ Centro Europeo di Risonanze Magnetiche Università degli Studi di Firenze Sesto Fiorentino Italy; ^2^ Dipartimento di Chimica “Ugo Schiff” Università degli Studi di Firenze Sesto Fiorentino Italy; ^3^ Consorzio Interuniversitario Risonanze Magnetiche di Metalloproteine Sesto Fiorentino Italy; ^4^ Florence Center for Data Science Università degli Studi di Firenze Florence Italy

**Keywords:** data analysis, paramagnetic NMR, Python, reaction monitoring

## Abstract

In this tutorial, we present *TrAGICo* (Trends Analysis Guided Interfaces Collection), a Python collection of functions for the extraction and analysis of experimental parameters from 1D and pseudo‐2D NMR spectra acquired on Bruker instruments. We demonstrate the application of TrAGICo through practical examples, highlighting its utility for various NMR applications, such as extraction of the chemical shift temperature dependence, relaxation studies, and reaction monitoring.

## Graphical abstract

*TrAGICo* is a Python collection of functions for the extraction and analysis of serial NMR spectroscopy data. In this tutorial, we show several practical examples, highlighting its utility for various chemical NMR applications, such as extraction of the chemical shift temperature dependence in paramagnetic systems, relaxation studies, and reaction monitoring.

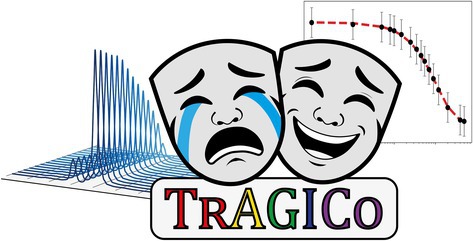

## 1 Introduction

NMR spectra are often collected in series, as many applications naturally involve the acquisition and analysis of sequential data. Common examples include studying the impact of temperature or chemical reactions, as well as examining relaxation processes and diffusion. Each spectrum in the series captures a snapshot of how these variables influence molecular environments over time or under varying conditions, and their combined analysis leads to detailed insights into the evolving patterns encoded in the spectra. Acquiring a series of spectra where the sampling timescale is a function of one of these modifications brings along several advantages, among which the possibility of gathering dynamic information on the effects that these phenomena have on the sample stands out. If one considers the sampling time of a series of spectra as the indirect dimension of a 2D experiment, fitting the evolution of signals in the indirect dimension with appropriate models that depend parametrically on the physical description of the source of modulation represents a valid approach to gain additional information on the system under investigation. Therefore, dealing with the analysis of series of spectra has become part of the routine in NMR spectroscopy. Such an undertaking used to come with sizable time overheads and thus, to simplify this task also to the non‐expert users, many software tools—either free or proprietary—have been designed to fulfill specific needs and to complete particular tasks (e.g., extracting relaxation or diffusion data). On the one hand, commercial programs are usually designed to perform specific analyses, often excelling in ease of use and extensive documentation. The drawback of commercial software is their black‐box nature, where the underlying algorithms and implementations remain largely inaccessible to users. Their highly specialized nature may also limit flexibility, requiring users to invest in multiple programs to cover different analytical needs, with significant additional costs. On the other hand, the NMR community has always been particularly active in producing custom scripts to enhance flexibility and expand the scope of analyses [1]. Not surprisingly, several tools have appeared for fitting and processing [2, 3, 4, 5] or simulation [6, 7, 8, 9], all the way to Spinach (a monument to the MR knowledge with a documentation that is beyond substantial [10, 11]). Drawbacks that are sometimes associated with custom software are that they may lack detailed documentation and examples, making them difficult for non‐experts to use and challenging broader adoption, and/or that they might rely upon proprietary languages (e.g., Matlab). These software usability issues need to be framed in the current request for FAIR data management from funding entities, as well as in the growing consciousness for open access in the “public money/public code” philosophy. As a result, there is a growing need for tools that balance accessibility, flexibility and transparency, which is reflected by the recent blooming of tools that are based on Python for readability by non‐programmers [12, 13, 14, 15, 16].

With the TrAGICo collection of functions, we aimed at making the analysis of series of NMR spectra easy to understand also for non‐specialists and to provide the users with increasing levels of freedom, depending on their level of expertise. In this tutorial, we show some archetypical applications to the extraction of chemical shifts and intensities.

## 2 Software

### 2.1 General Information and Dependencies

The program directory and test files can be downloaded at the link: https://github.com/letiziafiorucci/tragico.

The code has been developed and tested in Python (Version 3.12). The required additional dependencies include *numpy* (Version 2.0.2) [17], *matplotlib* (Version 3.9.2) [18], *lmfit* (Version 1.3.2) [19] and *nmrglue* (Version 0.10) [12]. The program should also work for different versions of the packages. Among the requirements, the most unstable is *matplotlib* (which has been extensively used for the graphical user interfaces) because changes in the API between versions might cause failures. For this reason, the graphical interfaces can be used to ease the process of input generation but their use is not mandatory.

The default data type read by TrAGICo is the Bruker format. However, with the inclusion of routines available in KLASSEZ^1^ (https://github.com/MetallerTM/klassez), other data formats are also supported. Some KLASSEZ's routines are used in TrAGICo and reported in the collection *f_functions.py*. By default, the program reads processed 1D or pseudo‐2D NMR spectra coming from the TopSpin software (vide infra). Pre‐processing of the spectra in such a program can be avoided by using the corresponding KLASSEZ processing functions (see TrAGICo documentation). In this tutorial, we will always refer to spectra from different Bruker instruments, already processed in TopSpin.

The code folder contains three Python scripts and detailed documentation: *main4test.py*, which contains few example codes, *f_fit.py*, containing all the functions that can be directly called in the user's main code—this is the only script that must be explicitly imported—and *f_functions.py*, which is a collection of general‐purpose functions used by *f_fit.py*.

An additional non‐executable script is present in the repository, named *manuscript_listings.py*, which collects all the listings present in this text and in the Supporting Information.

As TrAGICo is not provided as a standard Python package, therefore the associated scripts (*f_functions.py* and *f_fit.py*) must be placed in the same working directory as the user's .py script. It is also possible to use a Jupyter notebook file .ipynb, also in this case provided that the scripts are found in the same directory.

### 2.2 Structure of the Package, Inputs and Outputs

Throughout this tutorial, sections of the code will be presented as follows:while I/O files will be presented as follows:

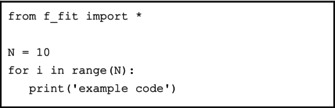

EXAMPLE I/O FILE :
line 1
line 2
line 3 




Terminal printouts are presented as follows:

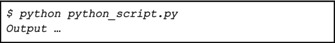

We can schematize TrAGICo in two main blocks, each pertaining to the specific data type that can be treated, i.e., series (or single) monodimensional spectra and series (or single) pseudo‐2D spectra. Of course, there is no substantial difference between handling a series of 1D spectra or a single pseudo‐2D spectrum, the only difference is in the way the script will need to read in the data. Regardless of the investigated data structure, the TrAGICo analysis follows the same two‐steps rationale. First, the intensity of the signals throughout the series is extracted from the spectra, then, the optimized parameters are fitted through the series with an appropriate model to describe their evolution. These two steps are described in detail in the following two subsections.

The decisional process that leads to the choice of the TRAGICO function to be called in the main script are summarized in the following scheme:

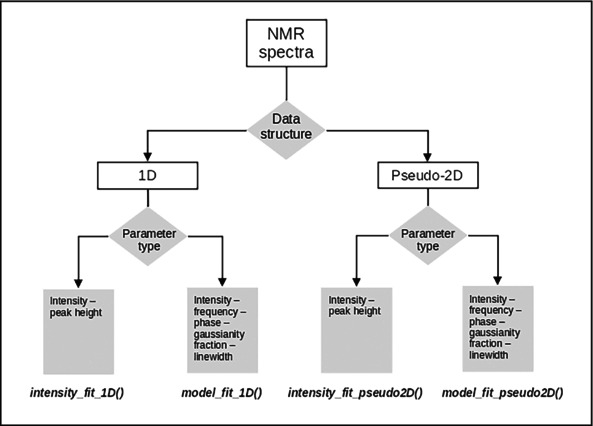

#### 2.2.1 The Cost Function for the Fit of the Spectra

The extraction of intensities is possible either as the highest point in a restricted region around the peak, by integration, or by deconvolution. In the latter case, the spectrum is modeled using the Voigt profile for the peaks and a polynomial baseline. This option also allows for studying the evolution of other parameters than the intensity, such as the chemical shifts or the linewidths.

When fitting the spectra in TrAGICo, the cost function is the squared norm of the difference between the real‐valued Fourier‐transformed NMR signal and the synthetic spectrum, composed of the hard model for the peak shapes combined with a fourth‐order polynomial representing the baseline (see Listing S1.2.1). To compensate for slight deviations from the pure Lorentzian shape, the Voigt model is used. The model signals are thus simulated, in the time domain, according to the equation:

(1) $$s\left(t\right)=k\;\exp\left[i\left(\mathrm{\omega t}+\phi \right)\right]\;\exp\left[-\left(1-x_{g}\right)\mathrm{\sigma t}/2\right]\;\exp\left[-x_{g}\Gamma^{2}t^{2}/2\right]$$

where $\Gamma =\sigma /\left(2\;\sqrt{2\;\ln\;2}\right)$. The parameters that describe each peak are the relative intensity k, the angular frequency with respect to the carrier ω, which will then be translated into the chemical shift δ, the full width at half maximum σ, the phase distortion ϕ, and the fraction of Gaussianity $x_{g}$ ( $x_{g}=0$ for pure Lorentzian, $x_{g}=1$ for pure Gaussian). At each call of the target function, the time‐domain signal of the peaks is computed, Fourier‐transformed, and summed with a 4th‐degree polynomial that serves as baseline:

(2) $$y=A+\mathrm{Bx}+Cx^{2}+Dx^{3}+Ex^{4}$$

where x is the portion of the frequency scale where the baseline is defined, and the coefficients A, B, C, D, and E are included as fitting variables.

#### 2.2.2 Evaluating the Accuracy of the Results

When estimating the intensity of the NMR signals via integration in TrAGICo, the integration error ε is computed on a user‐defined baseline‐only region as follows:

$$\varepsilon =N\;\mathrm{avg}\left\{S\left(n\right)-B\left(n\right)\right\}$$

where $\mathrm{avg}\left\{S\left(n\right)-B\left(n\right)\right\}$ is the mean value of the difference between the real‐valued spectral region *S* and the baseline *B* in the *n* points of the selected region, and *N* is the number of points of the integrated region. As a consequence, each peak region has a different error value.

Instead, if the height of the peak is chosen as an estimate for the signal intensity, the error will be set as the standard deviation in the error region, as this is the main source of uncertainty in this case. Therefore, the same error is used for all the peak regions.

A different estimate for uncertainty is used for modeling the peaks. The integral of each signal is first computed with the trapezoidal rule implemented in NumPy. Then, the error is estimated as the integral of the magnitude of the residual over a set of points that encloses a user‐defined percentage of the total integral. The default value is 95%, however, the user can customize it through the flag err_conf = <value from 0 to 1> of the model_fit_*n*D functions. For peaks whose residual regions extend beyond the fitting interval, an extra contribution proportional to the percentage of the missing peak area is added to the residuals. For instance, if a peak is cut in half by the fitting region, the error would consist of the error in the evaluated region plus an additional 50% to account for the portion that lies outside the fitting interval.

#### 2.2.3 Calibration of the Spectra in TrAGICo

In order to achieve an accurate quantification of the intensities throughout the series of spectra, as well as to correctly follow the chemical shifts, it is important to calibrate the spectra with respect to a reference signal. In TrAGICo, this processing can be performed through a dedicated flag in the main routines (vide infra).

To calibrate the spectra, the user is required to identify a “region of interest,” where the reference signal is located. Normally, a chemical shift referencing compound (e.g., DSS or TSP) is employed for this purpose. However, whatever signal can be chosen, given that it is present in all the experiments that compose the series.

At this point, assuming the reference peak to be in the correct position in the first experiment of the series, all the other spectra are circular‐shifted (using the function roll of the *numpy* package) with the aim of maximizing the superimposition of this user‐defined region throughout the series.

Of note, an equivalent procedure can be applied on the time‐domain dataset by replacing the circular shifting with the multiplication with a complex plane wave, whose frequency is the offset in Hz to be applied to the spectra. Such an operation translates in a convolution of the Fourier‐transformed data with a Dirac delta centered at the desired offset. This alternative approach actually performs better than the circular shifting in frequency‐domain because it is not restricted to the resolution of the chemical shift scale [20].

The function is implemented in the *f_functions.py* script of TrAGICo under the name calibration (reported in full in Listing S1.2.2), as well as in the function processing.align of the KLASSEZ package.

#### 2.2.4 The Behavior of TrAGICo

The core of TrAGICo consists of four functions, each of which is dedicated to a specific data format. Such a simple structure of the program encourages the tailoring of the available routines to accommodate more sophisticated analysis and integration of external programs.

Each of these functions, when called, will generate result folders, which collect output files and figures of the specific analysis. In Table S1.1, these functions and their arguments are presented in detail. For improved readability and comprehension, we will refer to these variables using the nomenclature given in *f_fit.py*.

In the specific case of (series of) 1D spectra, the function intensity_fit_1D (intensity obtained by head of the peak or integration) or model_fit_1D (intensity obtained via deconvolution) is in charge of setting up and administering the whole parameters extraction process, through a series of external guidelines passed by the user as arguments to the functions (depending on the function the arguments are positional or keyworded).

As the standard behavior of TrAGICo is to read the already processed spectra in Bruker format, the user is required to pass the parent directory of the whole dataset as path, and the experiments to read in such folder as list_path in the format “<expno>/pdata/<procno>.” Although these are mandatory parameters, and have to be specified anyways, the user can actually instruct the function to use externally‐processed spectra through the parameters Spectra and ppmscale, which represent the complex‐valued processed spectra (list of arrays) and the chemical shift scale (unique for the whole series), respectively. In addition, a series of acquisition and processing parameters must be passed through the variables acqupars and procpars, which can be conveniently generated by the functions param_acq and param_pr in *f_functions.py* from the *nmrglue* dictionary [12]. For the current version of TrAGICo this option is only available for the monodimensional dataset and will be expanded also to pseudo‐2D in future updates.

Before starting with the analysis, TrAGICo offers the possibility to calibrate the spectra with respect to a given signal: Such processing becomes active by declaring the interval of residence of the peak as cal_lim = (<ppm sx>, <ppm dx>).

At this point, the rest of the parameters diverge between the two employed functions, as the underlying methods are substantially different.

When studying intensity trends without hard modeling, the only information required from the user is the definition of the integration/peaking intervals. This can be done either by manually specifying the intervals in a text file and then passing its location through the fileinp argument, or by using a graphical interface that automatically generates such text files. The behavior of intensity_fit_1D during the analysis can be set via the area flag: if area = True, the intensity is estimated by integration, otherwise (area = False) the highest point in the marked region is taken. When the program starts, the peak selection interface opens, allowing users to define spectral regions by selecting and saving intervals. After all peaks are selected, switching to error estimation mode enables defining a peak‐free reference region, which is used for error estimation. The process is completed by saving and exiting.

When signals are not hard‐modeled, the computation of intensity values can be affected by baseline offsets. The baseline parameter of intensity_fit_1D, when set to True, enables the interactive computation of the baseline using a 4th‐degree polynomial, whose coefficients are automatically saved in a file. The path to this file can then be provided as baseline = <path to the coefficient file> to avoid recalculating the baseline every time. If baseline = False, no baseline correction is applied to the data. In the first case, a second interface—one for each selected region—will open, allowing the user to adjust these coefficients interactively before finalizing the correction.

For model_fit_1D and model_fit_pseudo2D, the generation of the initial guess for the fit occurs in two steps. Analogously to the intensity_ functions, also in this case, the procedure can be aided by graphical user interfaces.

In the first GUI, the user is asked to mark the region of the spectrum that they want to consider during the fit, and to mark the approximate peak position with the mouse. It is also possible to group peaks that belong to the same fine structure together. This information is saved in a file called *input_1*. For each region in *input_1*, the user can adjust the parameters of each simulated peak in order to match the appearance of the experimental spectrum, as well as to draft the baseline. These parameters and the baseline coefficients are thus saved in the *input_2* file. This process can be performed only once, as the paths of already existing *input_1* and *input_2* can be passed to model_fit_1D via the arguments file_inp1 and file_inp2, respectively.

For the actual fitting routine, it may be important to bind the parameters to vary in a restricted range, in order to drive the optimization process towards the correct solution. The argument limits1 serves this scope: It accepts a dictionary structured in the form “<parameter>”: (minval, maxval), where minval and maxval are the limit values that <parameter> can assume during the fitting process of the first spectrum. The other spectra in the series are expected to be very similar to the first one, thus it becomes convenient to approach their optimization in a sequential manner. The argument limits2, written with the same format as limits1, determines the allowed variability of the parameters with respect to the optimized parameters obtained as output of the fit of the first spectrum. The parameters that do not appear in these dictionaries are left unbound.

A further option that can affect the outcome of the fit is the introduction of regularizations on the parameters. Covariance is one of the main challenges in fitting NMR spectra. In order to tackle the optimization problem in presence of highly correlated parameters, regularization methods like the L_1_ and L_2_ regularization criteria have been implemented in the program. They can be used by setting the L1R and L2R parameters, which correspond to the scaling factor for the chosen regularization term. When they are equal to zero, the corresponding regularization is inactive.

The actual fit occurs if the dofit flag is set to True. The user can choose between two operation modes: fast = True, which is a single run with the Levenberg–Marquardt fitting algorithm, and fast = False, which makes a prior optimization with the Nelder–Mead simplex algorithm and refines it with a subsequent application of Levenberg–Marquardt. The latter option can be used for increased accuracy, at the cost of a sensible increase of the computational time due to the reduced efficiency of the simplex method.

The optimized parameters, fit reports, and the figures that display the outcomes of the fit are saved in a dedicated folder automatically created by the function.

The second part of the TrAGICo analysis consists in the fit of the trends obtained by the optimization of the parameters for each spectrum. The independent variable of the evolution is passed to the fit through the argument delays_list. Here, the user can choose between using the default model implemented in TrAGICo (i.e., a decaying exponential) by setting doexp = True, or by passing their own model function as f_int_fit. This model function must be of the form function(x, y, err_bars, **fargs), where x is the independent variable, y is the intensity collection, err_bar is an array with the uncertainties to be displayed as error bars, and fargs is the sequence of additional keyworded arguments that function can accept.

If intensity_fit_1D is used, it is implicit that the trend to fit concerns the intensities. With model_fit_1D, the employed model function allows to track not only the intensities but also chemical shifts, line widths, phases, and fractions of Gaussian (vide infra). Therefore, the parameter trend to fit must be specified through the Param argument.

The functions intensity_fit_pseudo2D and model_fit_pseudo2D resemble in the structure their 1D counterpart, except for the fact that they are adapted to read a different data format (i.e., the one of a pseudo‐2D experiment). For analyzing series of pseudo‐2D, the argument VCLIST is used to assign an identifier for each spectrum of the series.

The extra arguments in intensity_fit_pseudo2D concern how to compute the baseline and the intervals across the series. If prev_lims and prev_coeff are True, the same regions and the same baseline are used throughout the whole series of pseudo‐2Ds without alteration, respectively.

Furthermore, the parameter prev_guess of model_fit_pseudo2D instructs the fitting routine to use the same initial guess for all spectra of the series.

## 3 Use Cases

### 3.1 The Systems

In this tutorial, we guide the readers through a series of applications that are both standard in NMR data analysis and serve the scope of demonstrating the use of the functions included in TrAGICo. Throughout this tutorial, the examples are listed in order of increasing data structure complexity. In the interest of the reader who might be interested in a specific application, we summarize here the systems that we have chosen as representative. In each subsection, we will briefly remind the nature of the system selected for the demonstration.

All the data are deposited in Zenodo with DOI 10.5281/zenodo.15075841.

#### 3.1.1 NiSAL‐HDPT

**FIGURE 1:**
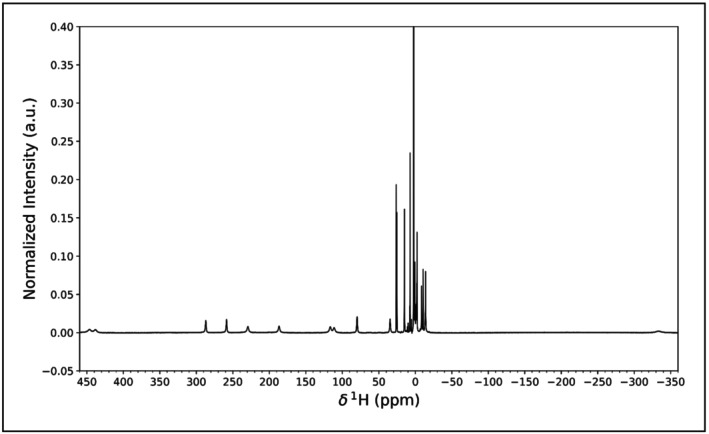
^1^H spectrum of NiSAL‐HDPT acquired at 298 K at 400 MHz ^1^H Larmor frequency. The assignment of the peaks is provided in reference [28].

Chemical shifts are arguably the most characteristic and important observables in NMR. They may change as a function of the concentration of the species in the mixture (including pH and ionic strength), but also of experimental measuring conditions, like temperature and magnetic field [21, 22]. Tracking chemical shift changes across a series of spectra is a very common task. One such example is represented by the analysis of the temperature dependence of the hyperfine shifts in paramagnetic complexes. NiSAL‐HDPT is an archetypical paramagnetic compound prepared by Ivano Bertini during his time in the group of Luigi Sacconi [23, 24]. This complex, where pentacoordination of the nickel center is sterically enforced, features hyperfine shifted signals spanning almost 1000 ppm. For its ease of preparation, it has been long used as a test system for the development of novel NMR hardware [25, 26, 27] and theoretical methods [28]. The spectrum of NiSAL‐HDPT is shown in Figure 1.

The spectra of D_4_‐methanol 99.8% (Bruker Biospin standard sample) and NiSAL (1 mM solution in deuterated chloroform, prepared as described in reference [24]) were measured at 9.4 T (400 MHz ^1^H Larmor frequency) on a Bruker Avance III spectrometer equipped with a room‐temperature selective high power ^1^H probe (SEL‐HP) [29]. The spectral window for methanol spectra was 4.2 ppm, and the excitation pulse was set to 8.9 μs. For NiSAL [28], the spectral width was set to 998 ppm, and excited with a 1 μs pulse. The total duty cycle of the experiment (0.1 s of recovery delay and 81.92 ms of acquisition) was optimized to saturate the residual solvent signal [30]. The temperature series was recorded in automation using the multizgte au program. The spectra were processed with a squared‐sine apodization to remove truncation artifacts from the FID and then phased.

#### 3.1.2 Human Ferredoxin 2

Paramagnetic metalloproteins represent a case in which the extraction of shifts is particularly challenging, as the signal linewidths can easily fall in the kHz range and severe spectral overlap is very often present. The availability of paramagnetic shift values is mandatory for a thorough characterization of the metal‐binding site [31, 32], as pseudocontact and contact shifts can be used to assess metal coordination and, in combination with quantum chemical calculations, to select candidate structures [33] or to perform experimental‐biased geometry optimizations [34, 35]. In particular, contact shifts are especially important in iron–sulfur proteins, where the highly symmetric electronic structure of the metallo‐cluster quenches pseudocontact effects [36].

As an example, we have selected oxidized FDX2, which is a paradigmatic case in the iron–sulfur (FeS) superfamily. The FeS cluster of FDX2 consists of an oxidized 2Fe‐2S core, where two antiferromagnetically coupled iron(III) ions are coordinated by four cysteine residues. While the ground state of this coupled system is diamagnetic (S = 0), its paramagnetic nature arises from the presence of low‐lying excited paramagnetic states [37, 38]. The electron relaxation time for [2Fe‐2S]^2+^ is among one of the slowest within the FeS clusters, hence the hyperfine‐shifted signals associated with the nuclear spins in the immediate proximity of the cluster suffer from severe line broadening which impairs detectability and results in strong signal overlapping. In such unfavorable conditions, it is extremely difficult, if not impossible, to obtain structural information from the analysis of contact shifts. For instance, in both redox states of the FDX2 cluster ([2Fe‐2S]^2+/+^), the paramagnetic broadening of β‐CH_2_ signals of the coordinating cysteines is so effective that signals are barely distinguishable within broad bands spanning several kHz in the ^1^H‐NMR spectrum.

An alternative approach to tackle this challenge is to change the observed nuclear spin: If ^13^C nuclei are detected instead of ^1^H, the paramagnetic relaxation is reduced by a factor of 16 [39, 40]. Nevertheless, ^13^C‐detected paramagnetic NMR spectra have their own difficulties, which make the development of appropriate analysis tools essential.

**FIGURE 2:**
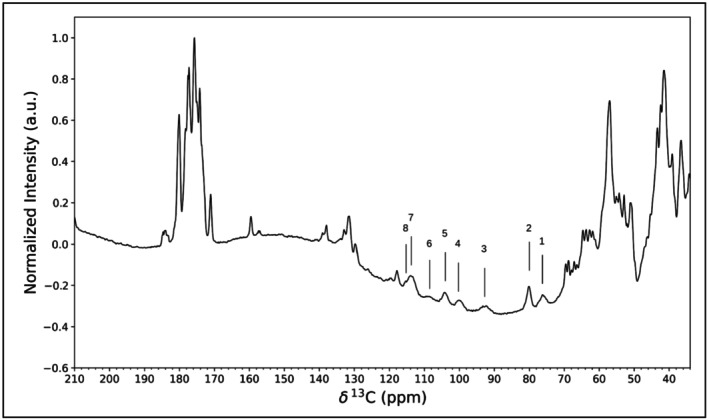
Human Ferredoxin‐2 ^13^C spectra acquired at 175 MHz ^13^C Larmor frequency at 298.0 K. The signals from alpha and beta cysteine carbons are marked with consecutive numbers from 1 to 8.

In the case of oxidized [2Fe‐2S]^2+^ FDX2, no additional field dependent effects are expected based on the paramagnetic nature of the cluster, therefore the use of ultra‐high magnetic fields can be beneficial. All the spectra resemble the one reported in Figure 2, where the paramagnetic peaks are labeled by consecutive numbers from 1 to 8 according to the assignment described in reference [41].

In FeS clusters, the expected nuclear spin relaxation mechanisms are Curie‐spin, Fermi contact and dipolar relaxation. It is often the case that we can adopt a simplified model where only the latter mechanism is considered, if one restricts the analysis to the longitudinal relaxation rate. A further simplification can be made by considering the electron spins fully localized on the Fe(III) ions. With this assumption, the paramagnetic relaxation enhancement due to the electron‐nucleus dipolar coupling depends on the inverse sixth power of the metal‐to‐nuclear spin distance. This makes longitudinal relaxation rate measurements valuable for obtaining structural information. Specifically, R_1_ relaxation rates of hyperfine‐shifted signals provide useful insights for refining the metal binding site, providing additional distance restraints for the FeS cluster [36, 42, 43, 44, 45]. R_1_ relaxation rates are also relatively easy to measure using paramagnetically‐tailored inversion recovery (IR) experiments [30, 46, 47, 48]. Each experimental parameter was optimized based on the expected relaxation rates of the system.

The ^13^C direct detected paramagnetic NMR spectra of FDX2 were acquired at 28.2 T (302 MHz ^13^C Larmor frequency) on a Bruker Neo spectrometer equipped with a cryogenically‐cooled TXO probehead [25]. Experiments were recorded with a superWEFT‐like pulse sequence [30], where relaxation delays as short as 1 ms were used. Inversion and excitation pulses have been calculated to cover an 80 ppm region because the paramagnetic signals appear in the 140–60 ppm region of the ^13^C spectrum. The overall duty cycle for the experiment (acquisition + recycle delay) was maintained below 100 ms in order to suppress diamagnetic regions of the protein and allow for the recording of a high number of transients over the whole experimental time. The temperatures in the series were 296.69, 299.59, 293.82, 290.96, 288.09, 285.22, 282.35, and 279.48 K, determined via the temperature calibration procedure described in section 4.2.1. Spectra were processed with a squared sine window function (SSB = 2) followed by an exponential line‐broadening of 40 Hz. The ^13^C inversion recovery (IR) spectra of FDX2 were acquired at 16.4 T (176 MHz ^13^C Larmor frequency) on a Bruker Neo spectrometer equipped with a carbon‐optimized cryogenically‐cooled TXO probehead [49]. The experiment was recorded at 298.0 K, for 17 different delays, ranging from 0.0001 to 1 s.

We obtained a conservative estimate of the longitudinal relaxation rates based on the X‐ray structure of FDX2 and on an estimate of the electron relaxation time: the T₁ relaxation times are not expected to exceed 20 ms. The total experiment duration was thus set to 128 ms. Based on the linewidths of the peaks, the acquisition time was kept as short as 65 ms, to minimize noise collection. The hyperfine shifted signals appear within an 80 ppm range around 120 ppm; therefore, 4096 time points were acquired with a 177 ppm spectral window, and the carrier frequency was set to 120 ppm. Finally, 17 relaxation delays were used: 100, 60, 40, 20, 15, 12, 10, 8, 7, 6, 5, 4, 3, 2, 1, 0.5, and 0.1 ms. The obtained spectra were processed using the same parameters as those used in the temperature series.

The protein concentration was 1.1 mM (quantified via UV/Vis signal of the cluster), buffered with 30 mM HEPES at pH 7.5 and in the presence of 150 mM of NaCl.

#### 3.1.3 In‐Cell Competition Experiments as an Example of Reaction Monitoring

Reaction monitoring strategies rely upon the quantification of different species that are formed. Since NMR is intrinsically quantitative, because the response of each nucleus within a sample is only dependent on its concentration (assuming full relaxation and homogeneous excitation throughout the spectrum), it is rather natural that NMR is applied to reaction monitoring. Usually, the means for quantification is integration, but it suffers from some limitations, mainly related to the spectral resolution.

In the case of Lorentzian peaks, the integration region that accounts for 99% of the signal intensity should be 64 times the linewidth of the signal [50]. In practical applications, it is quite uncommon to find such a signature peak for each chemical species that fulfill this requirement. With the wider diffusion of benchtop instruments for the task of process monitoring [51, 52], resolution becomes an even more important issue.

Therefore a sensible option is to fit the spectra in the indirect hard modeling approach [16, 53].

An excellent example along these lines is represented by monitoring drug‐target binding kinetics and thermodynamics within intact cells. Indeed, the non‐destructive nature of NMR makes it uniquely suited to investigate biomolecules inside living cells [54, 55]. In addition to studying the structural and dynamic properties of cellular proteins, in‐cell NMR can also be employed to screen the binding of potential drugs to a specific intracellular target, providing potency‐related information at an early stage of drug development [56]. To preserve cell viability in the spectrometer for prolonged times, in‐flow NMR bioreactors have been developed, which perfuse the cell sample with a constant flow of nutrients and remove metabolic byproducts, enabling time‐resolved NMR measurements on living cells [57, 58, 59]. As previously shown, these devices allow for real‐time monitoring of drug binding to an intracellular target and provide relevant insights on the kinetics of membrane permeability and—by competition binding—on the relative affinity of two different compounds for the same target [60, 61]. Recently, it was shown that ^19^F NMR detection of the ligand further extends the above approach to NMR‐invisible intracellular targets [62]. Fluorine is absent in living systems, providing virtually background‐free in‐cell spectra. Therefore, simple 1D ^19^F spectra can be recorded to quantify the fraction of fluorinated ligand bound to the target as a function of time.

**FIGURE 3:**
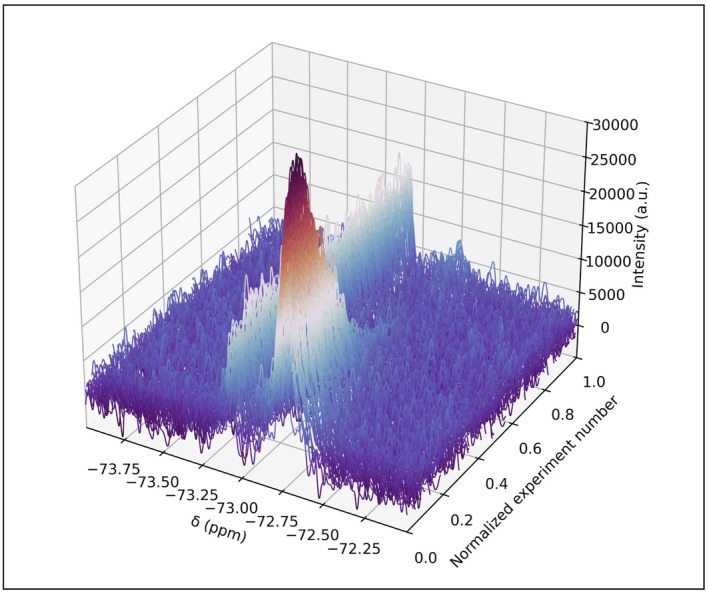
The entire time series of 296 ^19^F spectra for monitoring the fraction of ligand bound to HSP90_N_ in living cells as a function of time. Only the 2 ppm region that contains the signal of the extracellular and intracellular ligand is shown.

The dataset reported in reference [62] consists of four time series containing hundreds of time points each. Individual time series represent distinct competition binding experiments (i.e., monitoring the displacement of a ligand in the presence of increasing concentration of another). The ^19^F spectra contain two peaks in most cases (Figure 3, S2.1): one belonging to the extracellular fluorinated ligand, and the other to its intracellular counterpart bound to Hsp90_N_. The spectra feature partial peak overlap, severe baseline distortion, and relatively poor SNR for some of the spectra, which make this dataset an interesting target for testing quantification across the different series. In the absence of signal overlap, and with sufficient SNR, quantitative information can be obtained by signal integration, but fitting the spectra can improve quantitative analysis under target‐limiting conditions combined with poor instrument sensitivity and signal overlap.

^19^F NMR spectra were measured at 14.1 T (564.6 MHz ^19^F Larmor frequency) using a Bruker Avance III spectrometer equipped with a SEL‐HP probe [29], which was tuned at 564.6 MHz for ^19^F detection. Temperature was regulated at 310 K. To preserve cell viability, the experiments were run using a Bruker InSightMR flow system, and the cells immobilized in a gel matrix as described previously [63]. The ^19^F chemical shift scale was referenced to trichlorofluoromethane by setting the signal of trifluoroacetic acid in an external reference sample to −76.55 ppm. Acquisition was carried out with a pulse‐acquire sequence with a frequency offset of −66.7 ppm and a spectral window of 50.3 ppm. A series of ^19^F NMR spectra (512 scans and 1 s delay) was recorded with a time resolution of 11 min/spectrum for an overall duration of up to 66 h. The spectra were processed with 10 Hz exponential line broadening and phased.

#### 3.1.4 Olive Oil

**FIGURE 4:**
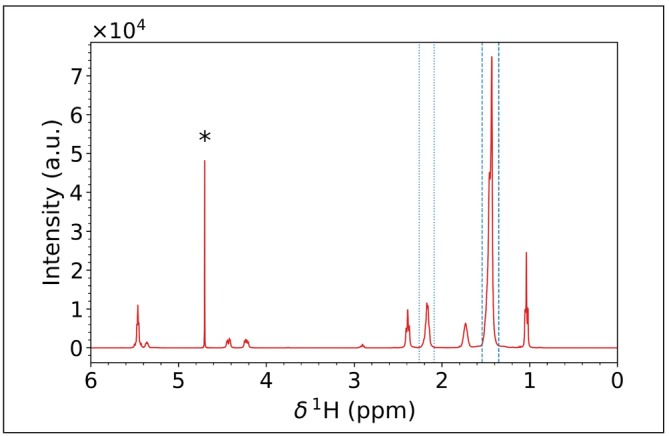
^1^H NMR spectrum of untreated olive oil acquired at 400 MHz at 298 K. The residual water signal, marked with an asterisk, arises from the deuterium oxide in the outer part of the coaxial tube (see methods). The considered ranges in Section 4.4.1 are marked with dashed and dotted blue lines.

Olive oil constitutes a popular, real‐world example of a complex viscous liquid. It is mostly composed of triglycerides, with the most abundant monomer being olein [64]. The investigation of the mobility of the relatively small triglyceride molecules in this liquid, where molecular interactions significantly slow molecular motions, is a very interesting field of application for relaxometry [65, 66]. In its neat form, the high viscosity makes the NMR signals quite broad (Figure 4). The broadening, combined with the spread of signals caused by the multiple species present in the natural mixture, makes their deconvolution quite challenging. We thus selected this case as an example where fitting the spectra might yield suboptimal results.

The spectra of pure olive oil were measured at 9.4 T (400 MHz ^1^H Larmor frequency) on a Bruker Avance III spectrometer, equipped with a 5 mm BBO probe. The spectral window was set to 10 ppm, with the carrier at 3.4 ppm. The excitation pulse was set to 17.1 μs. To achieve correct locking, the olive oil was sealed in a coaxial insert inside a 5 mm tube filled with D_2_O. Temperature was regulated at 298 K. During the experiment spectra with 32 scans and 16k points were acquired, which were zero‐filled to 32k. The spectra were processed with 0.2 Hz exponential modulation.

#### 3.1.5 High Resolution Relaxometry of Gadolinium Dissolved in Neat Ethylene Glycol

Nuclear relaxation rates can show field dependence, which can be further interpreted in terms of structure and dynamics. Traditionally, nuclear magnetic relaxation dispersion (NMRD) profiles, i.e., the relaxation rate as a function of the magnetic field, are acquired using fast field cycling (FFC) relaxometry [67, 68]. FFC offers a direct means of exploring low‐frequency dynamics by rapidly switching the magnetic field between different strengths and measuring the corresponding relaxation rates. Due to the low detection field (a few MHz ^1^H Larmor frequency) and poor field homogeneity, the main downside of FFC relaxometry is the lack of resolution. Until the 1990s, no technique was available to determine dynamics of complex systems in liquids, spanning from picoseconds up to microseconds, and with atomic resolution.

To overcome this issue, cycling between high and low magnetic fields using high resolution spectrometers has been experimented by Bryant and Redfield between the end of the 1900s and early 2000s [69, 70]. Later, this approach has been referred to as high‐resolution relaxometry (HRR) [71, 72]. Recently, an apparatus for HRR measurements has been developed by Bruker in the framework of the HiresMultiDyn project [73], with the name of fast sample shuttle system (FSS). During the simplest version of a HRR experiment, the sample is polarized at high field, then it is moved inside the magnet at different heights to expose the sample to the desired relaxation magnetic field, and, after a variable relaxation delay, the sample goes back to the high field position for detection.

**FIGURE 5:**
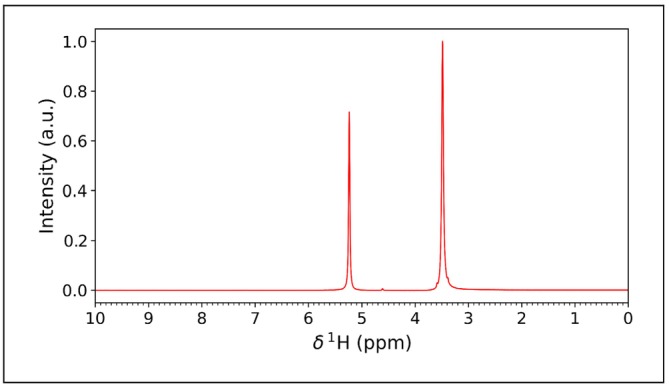
^1^H NMR static spectrum of ethylene glycol containing 10 μM of gadolinium(III) salt, acquired at 700 MHz, at 288 K.

The result of multiple HRR experiments is therefore a series of pseudo‐2D spectra, each at a different relaxation field. As examples, we show how it is possible to obtain the NMRD for the solvent protons in solutions of gadolinium(III) chloride dissolved in neat ethylene glycol for different concentrations of the salt (the 1D spectrum of ethylene glycol is shown in Figure 5). Because the FSS provides detection at high field, and therefore resolution, it enables the acquisition of T_1_ for both peaks of ethylene glycol, i.e., ‐CH₂‐ and ‐OH. The results will be compared with the usual FFC measurements, which measures a global T_1_ for the solvent.

Gadolinium(III) has seven unpaired electrons in the seven f‐orbitals, resulting in a non‐degenerate 8S ground state. This implies that electron spin relaxation mechanisms are rather inefficient, which in turn provides a very efficient mechanism for nuclear relaxation. The administration of gadolinium‐based contrast agents (CAs) in magnetic resonance imaging significantly improves tissue contrast by reducing both the longitudinal and transverse relaxation times of local water molecules. Despite the tempting feature of nine coordinated water molecules, gadolinium(III) ions are not used as such in clinical imaging because of their toxicity, and therefore, stable complexes are in use. Clinically, gadolinium‐based CAs are widely used for T_1_‐weighted imaging, where shorter T_1_ values produce brighter MRI signals, enhancing the visualization of anatomical structures and pathological changes. The study of the field dependence of the relaxivity is thus crucial in the rational design of innovative CAs.

The longitudinal magnetization decay/recovery curves at 26 different magnetic field values in the range 40–0.01 MHz were measured using the standard field cycling technique with a Stelar Spinmaster FFC‐2000‐1 T. The switching time, i.e., the time needed to change the field during the cycling, was set to 3 ms.

A fast sample shuttle system (FSS) [73] installed on a Bruker AVANCE NEO spectrometer operating at 16.4 T (700 MHz ^1^H Larmor frequency) has been used for the HRR measurements of the solvent protons in solutions of gadolinium(III) chloride in ethylene glycol, as a function of the magnetic field and for different concentrations of the salt.

The relaxation fields were set to 47, 125, 250, 500, 1000, 2000, 4000, 8000, and 15,500 mT.

### 3.2 Extracting Shifts Across Series of 1D Spectra

#### 3.2.1 The Temperature Dependence of Paramagnetic Shifts on a Small Model System

In this example, we show how to measure the hyperfine shifts as a function of the field on a small complex NiSAL‐HDPT, which is a paradigmatic example for paramagnetic NMR, featuring well resolved peaks across a range of about 1000 ppm.

Extracting an accurate dependence of the shifts on temperature intrinsically requires an accurate temperature calibration. We used 99.8% perdeuterated methanol to determine the correct temperature for each experiment: the methyl and the hydroxyl signals of methanol have different temperature dependence, which makes methanol an excellent tool for determining the temperature of the sample inside an NMR spectrometer [74]. After calibration, we extracted the shifts of NiSAL‐HDPT and analyzed them as a function of the corrected temperature. This first example aims at introducing the code execution strategy and output formats, which are valid also for all the other case studies in this tutorial. In particular, we focus here on extracting peak positions through fitting.

The chemical shift analysis across a temperature series starts with the definition of a *main.py* script, where the function model_fit_1D (see Table S1.1) from *f_fit.py* is called. The main script developed for this example looks like the following:


from f_fit import *
import numpy as np
path = 'path/to/spectra/folder/'
# in this case the experiments’ names correspond to consecutive numbers (from 1 to 12). The arange function takes as input (start, stop, step) and ends at stop-1.
num_sp = list(np.arange(1,13,1)) 
list_sp = [str(i)+'/pdata/1' for i in num_sp]
temp = []
for idx in range(len(list_sp)):
 _, _, ngdicp = nmr_spectra_1d(path+list_sp[idx])
 temp.append(ngdicp['acqus']['TE1'])
temp = np.array(temp)
# shift (ppm), lw (ppm), x_g (adim.), k (adim.), ph (rad), A-B-C-D-E (a.u.)
lim1 = {'shift':(-1,1), 'lw':(1e-4,2.5), 'ph':(-np.pi/20,np.pi/20),'A':(0,0), 'B':(0,0), 'C':(0,0), 'D':(0,0), 'E':(0,0)}
lim2 = {'shift':(0.9,1.1), 'lw':(0,0), 'ph':(0,0), 'xg':(0,0)}
_, _, (shift_tot, shift_tot_err), temp, _ = model_fit_1D(
 path,
 temp,
 list_sp,
 dofit=True,
 fast = True,
 limits1 = lim1,
 limits2 = lim2,
 file_inp1=None, 
 file_inp2=None,
 prev_fit=None,
 L1R = 0.0,
 L2R = 0.0, 
 Param='shift')




In line 1, the TrAGICo functions are imported. In the first lines of code, the variables that are passed to model_fit_1D are defined, and after that, there is the actual function calling. In this case, 13 spectra were analyzed.

The temperature values are read directly from the acqus file. The fit is performed using the Levenberg–Marquardt algorithm [75, 76] (leastsq in lmfit) on selected spectral regions, which include one or more peaks. The Param=”shift” flag defines the parameter that we want to study (in this case, the chemical shift).

During the fit, the baseline parameters were kept at the values defined in *input 2* (see dictionary lim1 in the code above).

For methanol, *input 1* is defined as follows:

**Table:** 

n. peak	name ^2^	ppm1	ppm2	v	mult ^3^
1	TRUE	3.466	3.217	3.32981	0
2	TRUE	4.906	4.81	4.86346	0

and *input 2* as follows:

**Table:** 

ppm1	ppm2	k	fwhm	ph	xg	A	B	C	D	E
3.5	3.2	0.0043	0.0037	0.0	0.2	0.0	0.0	0.0	0.0	0.0
4.9	4.8	0.0025	0.0029	0.0	0.2	0.0	0.0	0.0	0.0	0.0

Because file_inp1 and file_inp2 were both None, the program asks to assign a name to these two files, and then opens the GUI to create them (see the documentation for instructions on how to use the graphical interfaces). At the start of the actual fitting procedure, the terminal printout will look as follows:

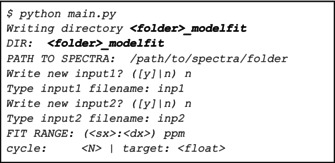

The output file, saved as *<folder>.out* in the result folder, presents a recap of the calibration procedure (if active), of *input 1* and *input 2* and the fit reports for each interval and each spectrum (see Supporting Information, Section S2).

In *<folder>_modelfit* , besides the output file, figures, integrals, Param values, W.M.J and uncertainties are automatically saved. Additionally, *lmfit* files collecting all the optimized fit parameters are saved. These files can be read by setting dofit = False when performing the fit is not necessary, but one just needs to check the optimized parameters (see Table S1.1).

**FIGURE 6:**
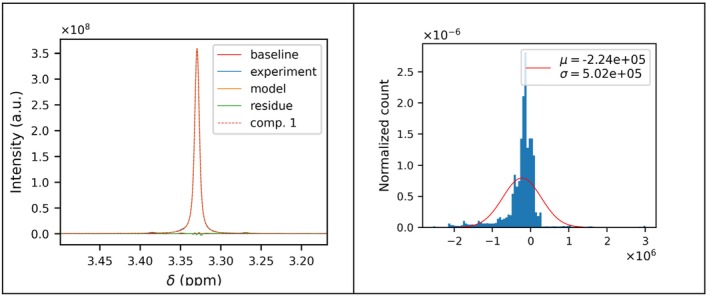
Fit figure (on the left) of CH_3_ peak of MeOH as the superposition of peak model, baseline, experimental ^1^H spectrum, and fit residual. The histogram of the residuals is compared with the corresponding normal distribution (on the right). These plots are generated for every fit interval and automatically saved in the modelfit folder.

The outcome of the fitting procedure is reported in Figure 6a, where the contribution to the spectrum of each component is shown. Figure 6b presents the histogram of the residuals after the fit (green curve in Figure 6a). Ideally, these residuals should follow a normal distribution to indicate a perfect fit. In this case, the residuals exhibit a slight polarization due to the use of a single signal to model the region, without taking the fine structure of the peak or the ^13^C satellite peaks into account. Nevertheless, the residuals are two orders of magnitudes smaller than the experimental signal, thus proving the success of the fit.

The corresponding Fit Report, automatically produced by *lmfit*, is reported below:


------------------------------
FIT RANGE: (3.47:3.22) ppm
I: 1 P: 1
------------------------------
Fit Report:
[[Fit Statistics]]
 # fitting method = leastsq
 # function evals = 43
 # data points = 4037
 # variables = 5
 chi-square = 1.5983e+15
 reduced chi-square = 3.9640e+11
 Akaike info crit = 107815.816
 Bayesian info crit = 107847.332
[[Variables]]
 shift_1: 3.32931441 +/- 1.3143e-06 (0.00%) (init = 3.32981)
 k_1: 0.004689 +/- 1.6972e-06 (0.04%) (init = 0.00434583)
 lw_1: 0.005346 +/- 3.8006e-06 (0.07%) (init = 0.00367081)
 ph_1: 0.01242673 +/- 2.9255e-04 (2.35%) (init = 0)
 xg_1: 0.56584136 +/- 0.00144026 (0.25%) (init = 0.2)
 A: 0 (fixed)
 B: 0 (fixed)
 C: 0 (fixed)
 D: 0 (fixed)
 E: 0 (fixed)
[[Correlations]] (unreported correlations are < 0.100)
 C(lw_1, xg_1) = +0.7690
 C(shift_1, ph_1) = -0.7546
 C(k_1, xg_1) = -0.7448
 C(k_1, lw_1) = -0.3079
n.peak Shift Integral
1 3.329 54936449643.506 +/- 435237381.724




In this report, the best‐fit parameters are shown, together with their uncertainty and covariance, to evaluate the quality of the fit. In this example, the uncertainties are extremely good, as a result of few components in the spectra and no baseline distortions—testified by the baseline parameters constrained to zero—and an excellent signal‐to‐noise ratio (SNR). In fact, little effort was needed to choose the proper variability ranges of fit parameters (this is not always the case, see Section 4.1.3).

With the best‐fit chemical shifts and their uncertainties at hand, it is possible to perform the temperature calibration using the chemical shift differences between the methyl and the hydroxyl groups using Equation (3) [74]:

(3) $$\begin{array}{c} T_{\text{corr}}=416.4745-38.5133\;\Delta \delta -36.0620\;{\left(\Delta \delta \right)}^{2}\\ +11.4869\;{\left(\Delta \delta \right)}^{3}-2.4340\;{\left(\Delta \delta \right)}^{4} \end{array}$$

where

(4) $$\Delta \delta =\delta_{\mathrm{OH}}-\delta_{CH_{3}}$$

**FIGURE 7:**
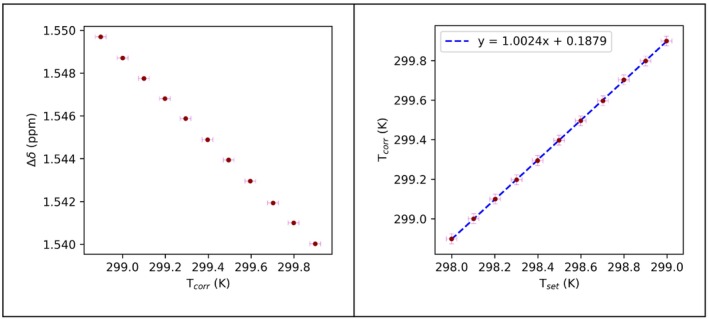
Plot of the difference in ^1^H chemical shift between the two signals of perdeuterated methanol versus the temperature computed with Equation (3) (panel a). In panel b, the plot of the correct temperatures versus the temperature set in the instrument. The regression line is indicated in blue. The horizontal error bars are ± 25 mK for each point [74]. Vertical error bars correspond to the sum of uncertainties from the fit of the two peaks (CH_3_ and OH). The code for the generation of this figure is reported in Listing S2.3.1.

With this function, the calibration curve of Figure 7a is obtained.

The linear regression equation for the plot of the temperature set on the instrument ( $T_{\mathrm{set}}$) and $T_{\text{corr}}$ (Figure 7b) was then used to compute the corresponding $T_{\text{corr}}$ for the Ni‐SAL‐HDPT temperature series of spectra.

Analogously to the MeOH case, the shifts were extracted using the model_fit_1D() function. The code listing, the output figures, and a section of the output file are reported in Section S2.2.4. In the function call, the only difference is in the definition of the temperature series, manually defined in this case, and in the allowed ranges for the fitting parameters. From the output section one can notice how, besides the very high SNR and the peaks being all singlets, the correlations among some variables are higher, leading to increased uncertainties.

**FIGURE 8:**
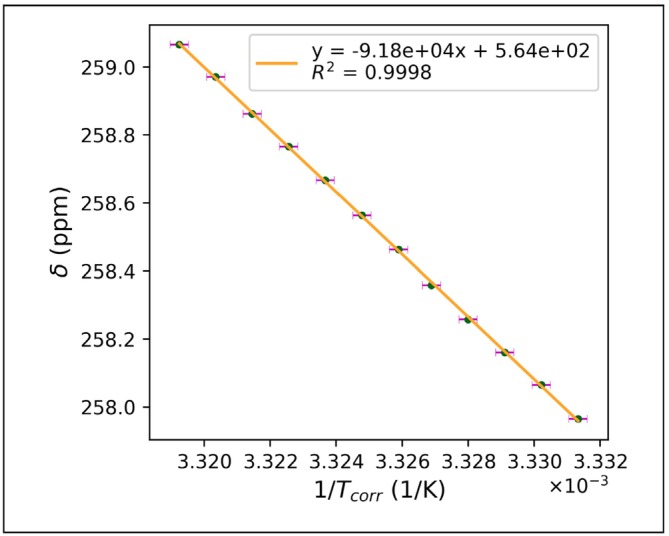
Plot of ^1^H chemical shift of ${\alpha_{2}}^{\prime }$ peak of Ni‐SAL‐HDPT versus the correct temperature. The vertical error bars correspond to the fit uncertainties and the horizontal error bars are set to ± 25 mK [74]. The plot of the entire spectrum acquired at 298 K is reported in Figure 1.

The Ni‐SAL‐HDPT shifts extracted from the fitting procedure were then plotted against the computed (correct) temperature, revealing an almost perfect correlation for all peaks (see Figure 8 and Section S2.7).

#### 3.2.2 Temperature Dependence of ^13^C Shift in the Oxidized Iron–Sulfur Protein Ferredoxin 2

For paramagnetic systems that have a larger number of signals, or where the shifts are smaller because of the different electronic properties, it is not necessarily the case that all the signals of interest are well resolved. This is the typical problem encountered for metalloproteins, where thousands of nuclei can respond to the NMR investigation. As an example, we have selected the oxidized iron–sulfur protein ferredoxin 2.

**FIGURE 9:**
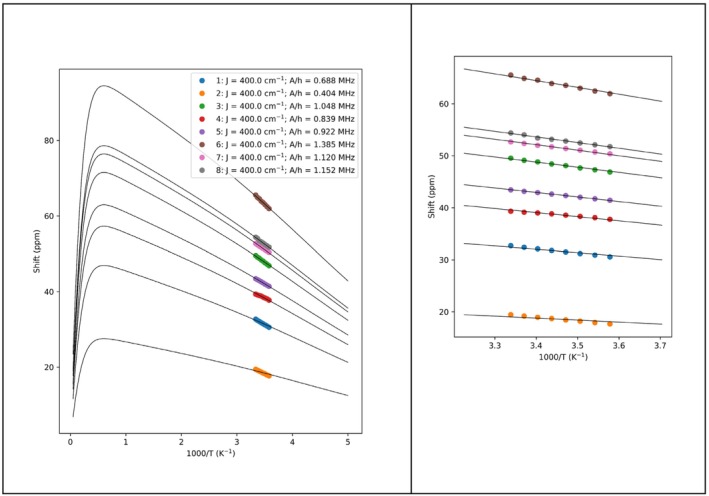
Temperature dependence of the human ferredoxin‐2 ^13^C chemical shifts. The black line represents the simulated data, whereas the dots mark the experimental chemical shifts extracted with Listing S3.1.1. The fitting of the temperature dependence of the shifts was performed according to the van Vleck equation using a *J* value of 400 cm^−1^. The fitting also provides, for each signal, the calculated hyperfine coupling constant, as reported in the inset of the left panel. In the right panel, the expansion of the experimental data, collected in the range 280–301 K.

Even though the quality of the recorded spectra is very high, signal identification is hampered by both serious broadening and overlap with diamagnetic peaks. However, such a limitation can be lifted if it is possible to fit the spectra. Therefore, we show how to apply an approach—similar to the one presented in the previous section—to obtain the temperature dependence of the paramagnetic ^13^C shifts of the signals near the iron–sulfur cluster in the ferredoxin protein. The chemical shifts of these peaks were extracted using the code listed in the Section S3.1. The situation here is far more challenging than the cases presented in the previous section, due to the strong baseline distortion that affects the spectral region of interest, coupled with the broad signal features. We can observe that, even in this case, the chemical shifts show a limited covariance with the baseline coefficients. To improve the stability of the fit, we applied an L1 regularization on the parameters through the dedicated flag. This regularization is applied as a constant factor, uniformly spread on the whole residuals, that adds a penalty based on the norm‐1 of the parameters. In addition, we used the Nelder–Mead algorithm coupled with Levenberg–Marquardt (fast = False, Listing S3.1.1). The experimental shifts plotted with respect to the acquisition temperature reveal a very good correlation, reflecting the stability of the method (Figure 9). The temperature dependence of the obtained shifts was modeled considering the paramagnetic contribution to the shifts as arising only from the contact contribution. Then, the shift values can be used to estimate the exchange coupling constant (J) and the Fermi contact coupling constants (A) through a minimization procedure (see Listing S3.2.1 and Figure 9).

### 3.3 Analysis of Intensity Trends via Integration and Modeling: ^19^F NMR to Monitor Binding and Unbinding in Living Cells

Given the intrinsic quantitative nature of NMR, it is a rather obvious choice to monitor the trends of chemical reactions through it. One particular example in reaction monitoring is the study of the ligand–target interactions in living cells to validate hits in the drug discovery pipeline. As an example, we have selected the analysis of the kinetics of ligand binding and unbinding using a fluorinated spy ligand. The first example in this section is obtaining the already published integral trends for the extracellular free ligand and for the same ligand bound to intracellular Hsp90_N_. (see fig. 4 in reference [62] and Section S4.1). The function used for integration was intensity_fit_1D with cal_lim = (‐68.00,‐78.00) and delta = 5, with two integration ranges selected through the graphical user interface (−73.233 to −73.033 ppm and −72.871 to −72.671 ppm, see TrAGICo documentation). Calibration limits should be selected in a way that they include a sizable portion of the spectral regions adjacent to the interested signals that contain no peaks. These noise‐only regions provide information on the pure baseline, preventing misalignment of spectra due to baseline shifts. The delta parameter expands the ppm scale by a user‐defined number of ppm units, with the purpose of facilitating the selection of an appropriate baseline. The script is as follows:


from f_fit import *
import numpy as np
path = 'path/to/spectra/folder/'
# the example covers one of our time series containing 368 experiments, starting from 101 and ending with 468
num_sp = list(np.arange(101, 469, 1)) 
list_sp = [str(i)+ for i in num_sp]
# this reads the delay directly from the acqus file
t = []
for i in range(len(list_sp)):
 acqus = open(path+list_sp[i]+"/acqus", "r").readlines()
 for line in acqus:
 if "##$DATE=" in line:
 date = float(line.split("=")[1])
 break
 t.append(date)
count = 0
delays = [0]
for i in range(1,len(t)):
 count += (t[i]-t[i-1])
 delays.append(count)
dir_result = intensity_fit_1D(
 path,
 delays,
 list_sp,
 area=True,
 cal_lim = (-68.00,-78.00),
 baseline=True, # if True, the baseline is subtracted
 delta = 5
 )




The contents of the resulting report, which were truncated for brevity, are shown below.


SPECTRA PATH:
path/to/spectra/folder
CALIBRATION: (-68.00000:-78.00000) ppm
in points
0 10 … -2 -1
in ppm
0.00000 0.03071 … -0.00614 -0.00307
Points:
1 0.000
2 0.003
…
295 0.997
296 1.000
Selected intervals (ppm):
1 -73.0330 -73.2330
2 -72.6710 -72.8710
===========================================
N. interval: 1
Coefficients
A -6.50990e+02
B 0.00000e+00
C 0.00000e+00
D 0.00000e+00
E 0.00000e+00
N. point Integral
0 237165.902 +/- 84289.975
1 339637.666 +/- 85825.077
…
294 387635.044 +/- 87707.922
295 392548.740 +/- 92285.070
N. interval: 2
Coefficients
A -5.00000e+02
B 0.00000e+00
C 0.00000e+00
D 0.00000e+00
E 0.00000e+00
N. point Integral
0 467900.601 +/- 84289.975
1 678428.643 +/- 85825.077
…
294 215049.460 +/- 87707.922
295 267157.420 +/- 92285.070




The report lists the calibration range, as well as the integration intervals selected by the user (two in our case). The user can access the baseline coefficients and the list of the obtained integrals for all the included spectra. For each of the selected intervals, integrals (with error bars) are plotted versus the normalized experiment number and saved as figures (see Section S4.2). The data used to create the figures are saved in three sets of text files, containing x‐axis, y‐axis, and error values, respectively.

As a second test, we compare the trends obtained through integration to those obtained by model fitting. This was performed on the ^19^F NMR time series using the model_fit_1D function (see Section 3.2.3). Each of the four time series required the adjustment of lim1 and lim2 to arrive at an optimal model fit in a reasonable amount of time/iterations. The allowed variations of the fitting parameters were based on prior inspection of the time series spectra. The chemical shifts were not allowed to vary for any of the initial guesses, i.e., in lim1; the same holds also for lim2 in three of the four cases. The assumption here is that the calibration using an external reference yielded spectra in which the chemical shifts of individual components remained stable throughout the respective time series.

It was only in a single time series that ‘shift’ = (0.9,1.1) yielded a better fit. In reaction monitoring experiments, peaks are obviously expected to vary in intensity, so we let k vary without limits, except in the initial guess, where it is simply allowed to take any positive value (non‐negativity constraint). In three of our four time series, we observed the decrease of linewidth for one of the peaks over time. This prompted setting the lw parameter in lim2 to (0.6, 1.1), (0.7, 1.1) and (0.3, 1.2) in the respective cases, thus allowing the model to follow the decreasing linewidth trend without using many iterations on trying to fit increasing linewidth. The fast argument was set to False because the Nelder–Mead algorithm improved the quality of the fitting (at the cost of computational time).

**FIGURE 10:**
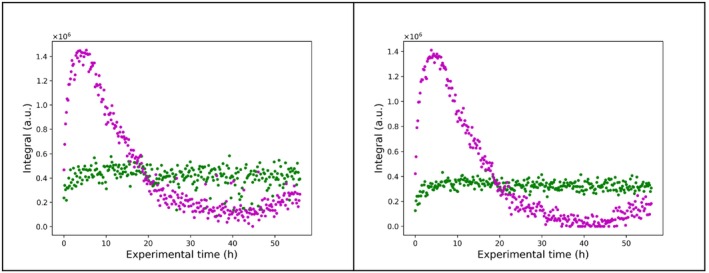
Comparison of trends resulting from integration of the signal of the free extracellular spy ligand (green) and of the spy ligand bound to HSP90N (magenta) in using intensity_fit_1D (panel a), which corresponds to the results published in reference [62], and model_fit_1D (panel b), which provides a smaller dispersion.

**FIGURE 11:**
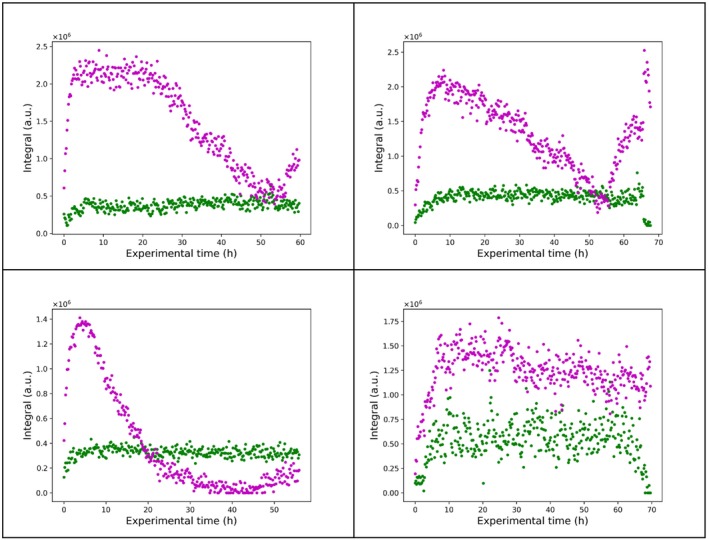
Reconstruction of previously published [62] integral trends in the four time series through the use of model_fit_1D in TrAGICo. Each time series corresponds to a different competition experiment, i.e., where the fluorinated spy ligand is displaced by increasing concentrations of a second non‐fluorinated ligand flowing through the bioreactor. The obtained intensity of the signals corresponding to the bound intracellular spy ligand and the free extracellular spy ligand are shown in magenta and green, respectively, for each time point in the series.

We found the introduction of model fitting to have positive effects on the dispersion of the data in the integral trend plots (Figures 10 and 11 created by directly reading the delays from the acqus file; Sections S4.2 and S4.3; code for plotting the trends in Section S4.4), especially in regions where the peak intensities of two ligand states were comparable, likely due to superior resolution of partial peak overlap in the case of the model as compared with regular integration. In reference [62], Bruker Dynamics Center was used to perform the quantification. The main bottleneck in this procedure was the baseline correction in Topspin, prior to import in Dynamics Center. On the contrary, model fitting in TrAGICo automatically accounts for the baseline. It is straightforward for the user to save the hard model(s) created by model_fit_1D for further applications. To this end, a conditional statement can be introduced to *f_fit.py* after the creation of the lor variable, i.e., an array that contains individual (Voigt) peaks that the user creates as initial guess for the fit. In our case, the peaks amount to two singlets belonging to two different compounds. At the end of the related for loop, all the Voigts are summed up together into a single array, thus saving the hard model. The code for saving the hard model (Section S4.5) should be inserted at line 1797 of *f_fit.py*, taking care that the sumcomp variable is initiated before the for loop at line 1789.

### 3.4 Analysis of 1D Relaxation Measurements

#### 3.4.1 Measuring T_1_ Relaxation in a Viscous Liquid

A rather intuitive way to measure T_1_ is through the IR experiment. When acquiring this experiment with the Bruker library sequence (t1ir), the experimental data is structured as a series of 1D ^1^H NMR spectra acquired in time‐domain, i.e., a pseudo‐2D experiment. As the name suggests, in these experiments, the direct dimension is Fourier‐transformed to yield a spectrum, whereas the other remains in the time domain. For complex organic or biological mixtures with multiple peaks, the analysis of the intensity trends across a pseudo‐2D IR spectrum provides the longitudinal relaxation rate R_1_ for each proton group, which carries information on the dynamic properties of the analyzed system. In this example, the acquired signals originate from protons in fatty acyl chains and the glycerol backbone in olive oil [65]. We here demonstrate the applicability of TrAGICo for the analysis of a pseudo two‐dimensional NMR dataset of an untreated olive oil sample. As an example, two peaks with different multiplicity will be considered in this section, which fall in the 1.543–1.352 ppm and 2.269–2.09 ppm ranges.

As previously mentioned in section 3.2.4, TrAGICo provides several options to analyze this type of data, which differ on how the intensity trends are extracted:

- as the integral of a user‐defined region of the transformed real‐valued spectrum (*main1.py*);
- as the highest point of a user‐defined region of the transformed real‐valued spectrum;
- as the integral of the peak model defined from the fit of a user‐defined region of the transformed real‐valued spectrum, with the same model used for monodimensional spectra (*main2.py*).

In this part of the tutorial, we will compare the outcomes of Strategies A and C for an IR experiment (Strategy B will be shown in section 4.4.3).

The following analysis can be conveniently performed in a folder (here TRAGICO_analysis) containing all Python scripts (.*py* extension), datasets (green), additional inputs (red), and results (blue).

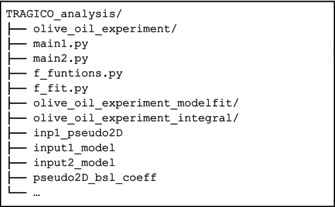

Case A:

The function used is intensity_fit_pseudo2D(), hence *main1.py* has the following structure:




### MAIN 1 ###
from f_fit import *
import numpy as np
# folder containing the spectra
path = 'path/to/TRAGICO_analysis/olive_oil_experiment/'
intensity_fit_pseudo2D(
 path,
 delays_list=[np.loadtxt(path+'1/vdlist')],
 list_path=['1'], 
 area=True, # True- Integration, False - Intensity
 VCLIST=[1.0],
 delta = 0.2,
 cal_lim = (2.5,2.3), 
 baseline=True, 
 doexp=False,
 f_int_fit = fit_IR,
 fargs={}, 
 fileinp=None,
 IR=True, # Inverts the order of the delays to make the interactive guess on the longest delay 
 err_lims_out= None, # (ppm1, ppm2)
 color_map = 'hsv')




In this section, a single pseudo‐2D spectrum is analyzed; therefore, delays_list and list_path are single‐element lists. As such, the timescale for the trend fit is read from the spectrum's VDLIST.

The area flag determines the intensity extraction method between the integration of the selected spectral interval (i.e., Method A, True) and the selection of the highest point in the interval (i.e., Method B, False), hence, in this case, area = True.

In either case, the fileinp looks like the following:


1.543 1.352
2.260 2.090




In the input definition through graphical interfaces, the transient of the pseudo‐2D associated with the shortest delay is used as default. As in this case, it is easier to define the inputs with respect to the longest delay (because it corresponds to the most sensitive spectrum), the flag IR is used to revert the ordering of the delays (IR = True).

In the result folder (olive_oil_experiment_integral), a second folder is created, named as the experiment number, where the plots and the intensity values are collected.

The structure of the result folder can thus be illustrated as follows:

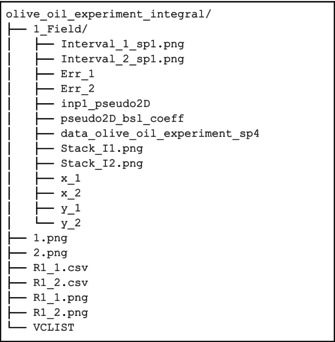

As mentioned before, the experiment was acquired at a single magnetic field; hence, we get as an output only one folder (1_Field). A more detailed description of the generated output can be found in Sections S5.1 and S5.2.

Because we are interested in the IR trend of the extracted intensity points, the data were fitted using an external function (fit_IR, Sections S5.3), which employs Equation (5) as model function.

(5) $$y=a+A\left(1-2\;\exp\left(-t/T_{1}\right)\right)$$

where $T_{1}$ is the longitudinal relaxation time, i.e., the parameter of interest, *t* is the delay (i.e., the independent variable), and the two factors *A* and *a* account for intensity and offset of the whole model function, respectively. One should also consider that as in an IR experiment the signals go from negative to positive, the time points close to the zero‐crossing are mostly populated by noise.

**FIGURE 12:**
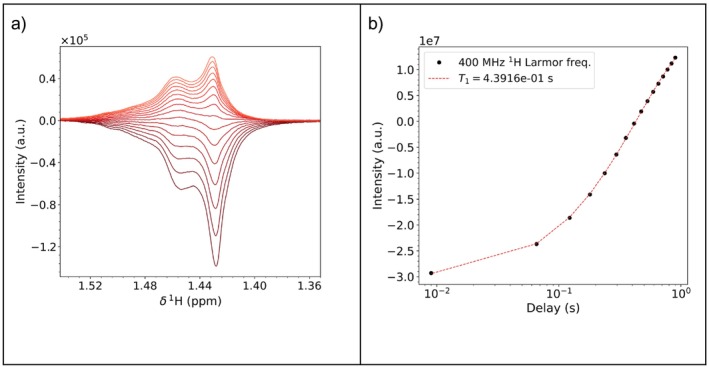
Output plots for changes of the peak corresponding to the methylene groups in the olive oil spectrum as a function of the increasing delays: appearance of the interest signals (a) and integrated intensity (b).

The resulting output plots are shown in Figure 12. The plots for the second peak can be found in Sections S5.4.

Case C:

To get the intensities via deconvolution, the function to be used is model_fit_pseudo_2D(). The main defined in this case is listed here below:




### MAIN 2 ###
from f_fit import *
import numpy as np
path = 'path/to/TRAGICO_analysis/olive_oil_experiment/'
list_path = [int(f) for f in os.listdir(path) if not f.startswith('.')]
list_path = [str(f) for f in np.sort(list_path)]
delays_list = [np.loadtxt(path+list_path[i]+'/vdlist')+3e-3 for i in range(len(list_path))]
lim1 = {'shift':(-0.05,0.05), 'lw':(1e-4,0.05), 'ph':(-np.pi/20,np.pi/20), 'k':(-1,1), 'B':(0,0), 'C':(0,0), 'D':(0,0), 'E':(0,0)}
lim2 = {'shift':(0.95,1.05), 'lw':(0,0), 'ph':(0,0), 'xg':(0,0)}
model_fit_pseudo2D(
 path, 
 delays_list,
 list_path, 
 cal_lim = (2.5,2.3), # limits for the calibration
 file_inp1 = None,
 file_inp2 = None,
 fast = True, 
 VCLIST=[1.0],
 dofit = True,
 limits1 = lim1, 
 limits2 = lim2,
 L1R = 0.0,
 L2R = 0.0,
 f_int_fit = fit_IR,
 fargs={}, 
 IR=True, # Inverts the order of the delays to make the interactive guess on the longest delay
 doexp=False)




Analogously to the previous section, the input files are generated based on the last transient of the pseudo‐2D. Importantly, here the relative intensity ‘k’ must fall within the −1 to 1 range, in order to allow for fitting the negative peaks as well. Moreover, the phase variation ranges should be restricted to avoid the optimization of the negative intensity with a phase variation. The input files used in this example, i.e., input1_model and input2_model, are reported in Sections S5.5. Contrary to Case A, the result folder olive_oil_experiment_modelfit for the modelfit approach has different output files:

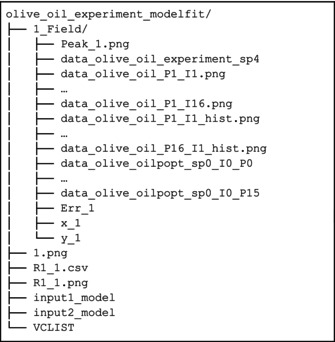

When performing this kind of analysis, it is recommended to focus on only one peak/multiplet due to longer calculation time. Examples and more detailed descriptions of the output files have been collected in Sections S5.6 and S5.7.

A comparison of the obtained R_1_ values for both signals between the two methods is reported in Table 1.

**TABLE 1:** Relaxation rates R_1_ (+/− error) for selected olive oil peaks (1.543:1.352 above, 2.269:2.089 below), where data was extracted with integration (Case A) and model fit (Case C) approach.

Data extraction method	R_1_ [s^−1^] +/− error
Integration	2.281 +/− 0.026 2.341 +/− 0.024
Model fit	2.338 +/− 0.024 2.448 +/− 0.033

It is apparent that the obtained R_1_ values depend on the applied method, but their relation remains the same, as well as the errors, which are comparable in both approaches. However, integration seems to be more accurate for these peaks, as it was expected on the basis of the considerations about the complexity of these signals previously described in the Section 4.1.4. Summing up, when extracting trends out of complex olive oil spectra beside the intensity method all others give reliable results. When the profile contains more than one relaxation rate, small deviations of their numerical value may not affect the correlation time τ_c_ that much, as the shape of the dispersion curve does.

#### 3.4.2 Measuring T_1_ Relaxation in a Paramagnetic Metalloprotein

In this example, we evaluate an IR pseudo 2D dataset measured on the paramagnetic iron–sulfur protein FDX2 in its oxidized state (the same system of Section 3.1.2) where the relatively long electron relaxation rates are expected to yield very short nuclear relaxation rates. We analyze the intensity trend of an IR to extract the longitudinal relaxation values (as in Section 3.4.1) using a modified version of the model_fit_pseudo2D function. This modified version also includes a different regularization function than those available in the standard code. This approach enhances the reproducibility of the results, and makes it possible to work with experimental data of lower quality, therefore saving time in the acquisition stage. To prove this point, the experiments were replicated for two different values of number of scans (NS): 16 k (LNS) and 128 k (HNS). The results are compared with those obtained through the built‐in T_1_ fitting method in TopSpin (*v4.0.8*) integrated software Dynamics Center (*v2.6.2*).

As already shown in Figure 2, the spectra display a strong baseline distortion in the region of interest and very broad spectral features. For these reasons, the present example requires fitting the spectra for extracting the intensities (see Case C in Section 3.4.1).

The adapted fitting protocol is divided in three fundamental steps (the modified version of the target function is available in code listing S6.1):

1. In the first round of fitting, the model_fit_pseudo2D function is applied.
2. In the second round, the whole pseudo‐2D spectrum is considered as a bidimensional array (*m* × *n*), where *m* is the number of delays and *n* is the number of experimental points. The initial guess for peaks and baseline is composed of the parameters obtained from the first fit round, which differs for each delay. These parameters are read from the files that are saved in the *_modelfit* folder at the end of Step 1. At this stage, the limits on the shift parameters are further tightened, while keeping the $x_{g}$, ϕ, and Γ fixed. The target contains the difference between the model spectra and the experiments, with the addition of a soft regularization term defined by the T_1_ profiles. The model for the intensities decay is the simple recovery profile: $1-2\exp\left(-t/T_{1}\right)$. The residue is normalized, and the regularization weight factor is set equal to 1. The addition of this regularization function does not imply that the integral values will be completely determined by the model of the intensity profile, but just that the results would be slightly adjusted in this sense and stabilized, compensating in part for the effect from the covariance among baseline parameters and the relative intensities of the peaks.
3. Finally, the intensity profiles are fitted using f_int_fit = fit_IR.

The intensity profiles are reported in Figure S6.2.1 for HNS and Figure S6.2.2 for LNS, respectively.

**FIGURE 13:**
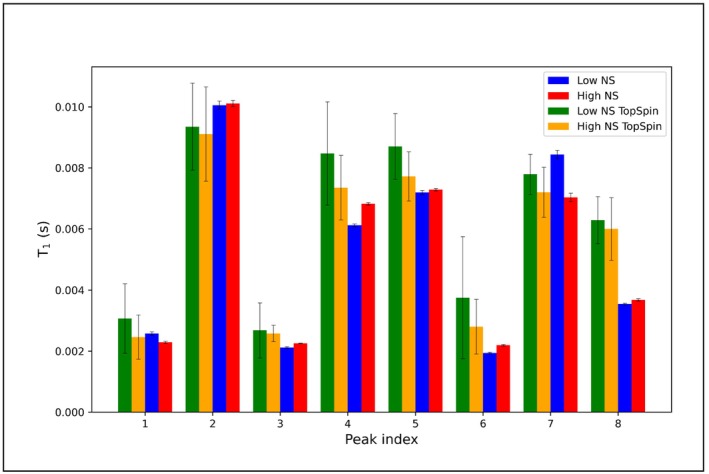
Bar plot of T_1_ values for cysteine peaks labeled from 1 to 8, going from the most upfield to the most downfield, obtained from the presented approach and from TopSpin integrated software Dynamic Center, for both LNS and HNS datasets.

The bar plot in Figure 13 shows that the error bars of the T_1_ extracted with this analysis are much smaller than those from the Bruker TopSpin software, reflecting the higher deviation from the model trend in the latter case (see Figures S6.3.1 and S6.3.2), and that simultaneous extraction of trends and peak quantitation makes the result more stable with respect to data quality [77]. In fact, the T_1_ from Bruker's software are systematically lower for the dataset with higher number of scans. Peaks 7 and 8 are those that present the highest variation between the two datasets and that differ the most from the TopSpin result, because of their strong overlap. This is a further indication that the fit of the spectra is crucial for obtaining accurate structural information in the present case, which is apparent by just visual inspection of the different spectra (see Figure S6.4.1).

#### 3.4.3 Analysis of Relaxation Measurements at Variable Magnetic Field—Paramagnetic Relaxation at Variable Fields

NMRD profiles, showing the field dependence of nuclear relaxation rates, offer the possibility to probe dynamic processes over a wide range of time scales and, in paramagnetic systems, to monitor the coordination of solvent molecules to paramagnetic metal ions. They are extremely useful for the development of MRI CAs because they permit not only to predict their efficiency in any magnetic field but also to understand and improve their mechanisms of action. In this example, we show the extraction of the longitudinal relaxation rates as a function of the magnetic field (NMRD profile) using the data acquired on solutions of GdCl_3_ in ethylene glycol acquired using an FSS system. The analysis is shown for two samples: neat ethylene glycol, and a solution of 10 μM of GdCl_3_ in ethylene glycol.

As in the case of the previous example (Section 3.4.1), we will compare the results from different intensity extraction methods (methods A—integral, B—intensity, and C—model fit) for the fit.

Bruker datasets from an FSS system come in a predefined format:

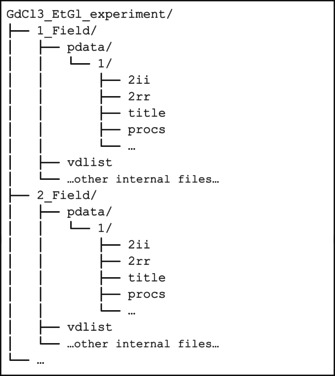

Inside the root dataset directory (GdCl3_EtGl_experiment), files for all relaxation fields are included in the same order in which they were sampled. The sampling of the relaxation fields occurs in a pseudo‐random order—in order to mitigate the potential impact of sample degradation over long periods of time, but TrAGICo can handle the fields in any order. Every subfolder (1_Field, 2_Field, …) maintains the structure of a pseudo‐2D, with files like the vdlist and the pdata folder, containing the spectral data (2ii, 2rr).

The main scripts are structured in the same way as in Section 4.4.1 because the only changes that are needed for the present case are that list_path, delays_list, and VCLIST have multiple entries, e.g., VCLIST should have the list of each field value that corresponds to each pseudo‐2D in the list_path.

Using a notation that is coherent with Section 3, the output files in this case are named according to the intensity extraction method: GdCl3_EtGl_experiment_integral, GdCl3_EtGl_experiment_intensity, GdCl3_EtGl_experiment_modelfit for methods A, B, and C, respectively. Each of these folders is structured as follows:

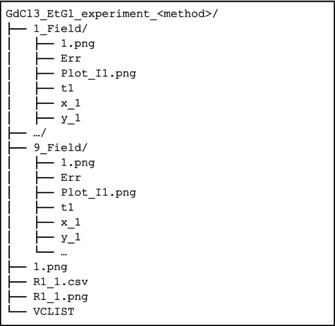

In the directory, TrAGICo generates a folder for each of the paths in list_path, i.e., one for each field. The results that are saved in each folder are analogous to those presented in the previous section. In addition to those, TrAGICo saves the NMRD profiles and all the R_1_ values in a dedicated file in the parent directory.

The main scripts for this example are reported in the Section S7.1. As shown in Section 3.1.5, the ethylene glycol spectrum features two peaks, which are relaxed by the gadolinium ion.

**FIGURE 14:**
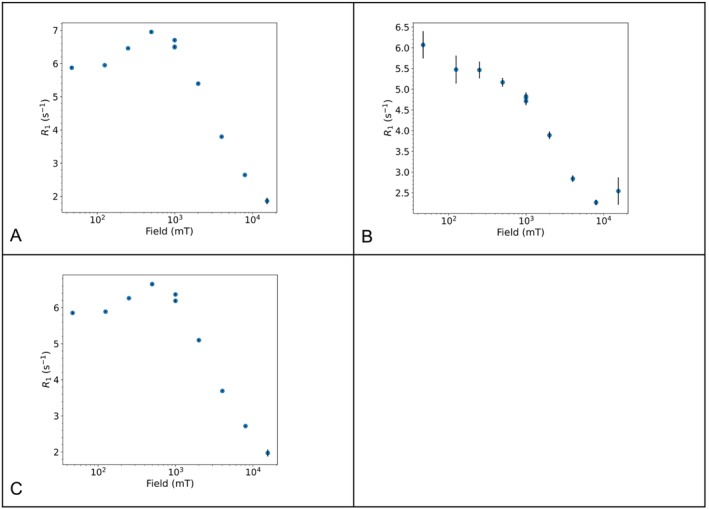
Examples of R_1_ plots of ethylene glycol with 10 μM of GdCl₃ obtained with (A) integration, (B) intensity, and (C) model fit, for ‐CH_2_‐ signal of ethylene glycol.

Examples of R_1_ plots of ethylene glycol with 10 μM GdCl₃ obtained using the three extraction methods (integral, model fit, and intensity) are shown in Figure 14.

In these plots, the R_1_ trends obtained with the integral method and the model fit method are very similar, while the intensity method shows larger error bars, and a different trend in R_1_ values.

**FIGURE 15:**
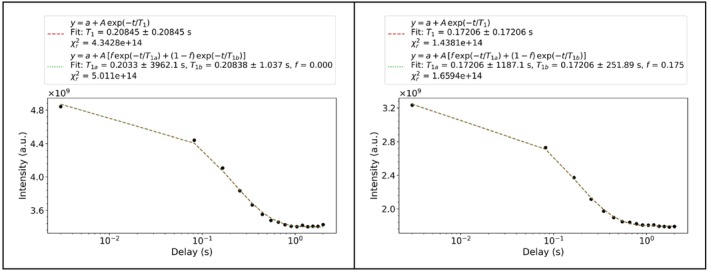
Plot generated using the intensity function of the two peaks of ethylene glycol, respectively, in the sample with 10 μM GdCl₃. Both trends were fitted with a mono‐exponential (red curve) and a bi‐exponential model (green curve).

The fit across the trend of the integrals (Method A) is shown in Figure 15, and it is performed using a monoexponential and a biexponential function, for both signals of ethylene glycol. The two fit lines are superimposed because the biexponential fit optimizes two curves with the same R_1_ value, so that their sum becomes equal to the monoexponential fit.

**FIGURE 16:**
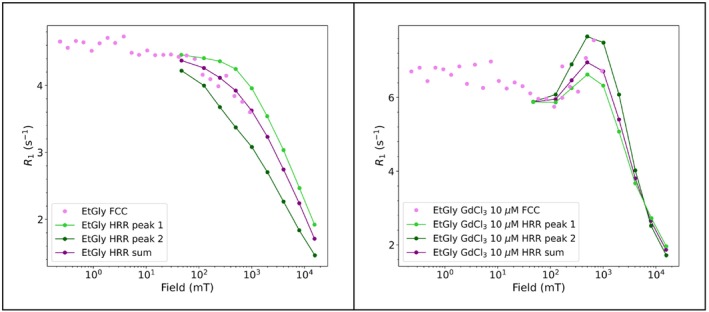
Plots showing the NMRD profiles of neat ethylene glycol (left) and ethylene glycol with 10 μM GdCl_3_ (right). Each figure compares FFC relaxometry data (pink) with HRR data (dot‐lines). The purple line corresponds to the R₁ of the integral of both peaks computed together.

The longitudinal relaxation rates R_1_ that are extracted with the fit can be compared with the low resolution relaxometry data obtained on the same samples using the FFC Stelar SpinMaster2000 relaxometer. Of course, the two signals have a different trend with respect to the overall profile provided by the FFC relaxometer, because it provides the relaxation of the sum of the two signals. Indeed, if we integrate over the two peaks in the HRR spectra and we extract the NMRD profiles, the result overlaps nicely with the FFC data for both samples (Figure 16).

Without the presence of the paramagnetic ion, R_1_ values decrease monotonically with the increase of the magnetic field. Introducing the gadolinium(III) ion, R_1_ values are higher, and they show a relaxivity peak as a consequence of the slow tumbling of gadolinium(III) ions and the longer correlation time for the electron‐nucleus dipole–dipole relaxation in ethylene glycol with respect to water [78].

## 4 Conclusions

With this tutorial based on the TrAGICo collection of functions, we aimed at making the analysis of series of NMR spectra easy to understand also for non‐specialists, and at the same time provide the users with increasing levels of freedom, depending on their level of expertise. To make each step of the analysis transparent to the user, we use a combination of Python scripts, which are easily interpretable and interoperable, with graphical user interfaces to automate and simplify each task. Finally, function‐based scripting allows the user to retain the choice of the model used to extract the information and control over the functions that are used to fit the data.

## Peer Review

The peer review history for this article is available at https://www.webofscience.com/api/gateway/wos/peer‐review/10.1002/mrc.5537.

## Supporting information


**Data S1.** Additional information on TrAGICo implementation.
**Data S2.** Chemical shift temperature dependence and calibration.
**Data S3.** FDX2 temperature dependence of the hyperfine shifts.
**Data S4.** Reaction monitoring with 19F NMR.
**Data S5.** Analysis of pseudo‐2D relaxation measurements for olive oil sample.
**Data S6.** FDX2 longitudinal relaxation rates fit.
**Data S7.** Analysis of relaxation measurements at variable magnetic field.

## Data Availability

The data that support the findings of this study are openly available in Zenodo at https://zenodo.org/records/105075842, reference number 105075842.

## Endnotes

^1^
The klassez package is a collection of classes and functions to handle NMR data, which is described in the Doctoral Thesis of F.B. [79]. It is organized into modules, each of which contains functions dedicated to specific processing tasks. Full documentation is available, after installation of the package, through the function open_doc, which displays the file klassez.pdf located in the “docs” subfolder of your install dir.

^2^
A FALSE flag in this column indicates that the corresponding peak will be treated only to improve the agreement between the model and the experiment, but it will not be considered for intensity (or other parameters) trend studies.

^3^
The multiplicity column defined in the input does not refer to the actual peaks' fine structure, but represents a numeric flag, where equal values correspond to peaks belonging to the same multiplet.
