# Supplementary material for: Extracting Trends From NMR Data With TrAGICo: A Python Toolbox
Source: Magn Reson Chem. 2025 Jun 12;63(8):628–54. doi: 10.1002/mrc.5537 (PMC12223958; doi:10.1002/mrc.5537)
Supplement: Supplementary file 1 — Data S1. Additional information on TrAGICo implementation. Data S2. Chemical shift temperature dependence and calibration. Data S3. FDX2 temperature dependence of the hyperfine shifts. Data S4. Reaction monitoring with 19F NMR. Data S5. Analysis of pseudo‐2D relaxation measurements for olive oil sample. Data S6. FDX2 longitudinal relaxation rates fit. Data S7. Analysis of relaxation measurements at variable magnetic field. [file MRC-63-628-s001.docx]

**Supporting Information**

**Extracting Trends from NMR Data with TrAGICo: A Python Toolbox**

Authors: Letizia Fiorucci^a,b,c,+,%,^*, Francesco Bruno^a,b,+,^*, Leonardo Querci^a,b^, Adam Kubrak^a,b,c^, Jlenia Bindi^a,b,c^, Nebojša Rodić^a,b^, Giulia Licciardi^a,b,c,$^, Enrico Luchinat^a,b,c^, Giacomo Parigi^a,b,c^, Mario Piccioli^a,b,c^, Enrico Ravera^a,b,c,d,*^

a - Centro Europeo di Risonanze Magnetiche, Università degli Studi di Firenze, Via Luigi Sacconi 6, 50019 Sesto Fiorentino, Italy

b - Dipartimento di Chimica “Ugo Schiff”, Università degli Studi di Firenze, Via della Lastruccia 3, 50019 Sesto Fiorentino, Italy

c - Consorzio Interuniversitario Risonanze Magnetiche di Metalloproteine, Via Luigi Sacconi 6, 50019 Sesto Fiorentino, Italy

d - Florence Center for Data Science, Università degli Studi di Firenze, Viale G.B. Morgagni 59, 50134 Firenze, Italy

+ - These authors contributed equally

% - Present address: Max Planck Institut Für Kohlenforschung, Kaiser-Wilhelm-Platz 1, 45470 Mülheim an der Ruhr, Germany

$ - Present address: Laboratoire des Biomolécules, LBM, Département de chimie, École normale supérieure, PSL University, Sorbonne Université, CNRS, 75005 Paris, France

* Correspondence to: Letizia Fiorucci ([fiorucci@kofo.mpg.de](mailto:fiorucci@kofo.mpg.de)), Francesco Bruno ([francesco.bruno@unifi.it](mailto:francesco.bruno@unifi.it)), Enrico Ravera (enrico.ravera@unifi.it)

[**S1. Additional information on TrAGICo implementation 2**](#_gnlm0q3dxkpz)

[S1.1 Arguments and description of the main routines in TrAGICo script 2](#_mftyscrf6fjk)

[S1.2 TRAGICO routines 7](#_av95yrfo5h93)

[**S2. Chemical shift temperature dependence and calibration 12**](#_sg6w3s5z6g59)

[S2.1. AU program for the acquisition of a series of spectra at increasing temperatures 12](#_y5djo9ms2b2h)

[S2.2. Output file for MeOH spectra 13](#_iyre61ort0dj)

[S2.3. Temperature calibration with MeOH 16](#_xz7gqj13pgfy)

[S2.4. Code listing for extraction of Ni-SAL-HDPT chemical shifts 17](#_cqmemsuctmot)

[S2.5. Ni-SAL-HDPT fit result plots 17](#_4yeddfntuhut)

[S2.6. Ni-SAL-HDPT output file 22](#_96e4nrww7rww)

[S2.7. Chemical shift temperature dependence Ni-SAL-HDPT 26](#_8129b381140j)

[**S3. FDX2 temperature dependence of the hyperfine shifts 31**](#_7uhxvmsuy8p1)

[S3.1 Extraction of chemical shift values 31](#_wmx4rh6tocf)

[S3.2 Modeling of chemical shift field dependence in exchange coupled systems 35](#_qt7zbyn6tspi)

[**S4. Reaction monitoring with 19F NMR 37**](#_qqcq7kzh3w7s)

[S4.1 3D snippets of sample datasets 37](#_fnoi3dgo8ba4)

[S4.2 Integral plots via intensity_fit_1D 38](#_zdj5rfcln7sp)

[S4.3 Integral plots via model_fit_1D 39](#_utezi3940sqj)

[S4.4 Code for plotting two integral trends simultaneously 40](#_941xg99nvavs)

[S4.5 Code for saving the hard model 41](#_h32wxyt6vw43)

[**S5. Analysis of pseudo-2D relaxation measurements for olive oil sample 41**](#_7icattsjjnta)

[S5.1. Result folder of intensity fit pseudo2D function 41](#_hic5hhd0dfbe)

[S5.2. Output file for intensity_fit_pseudo2D - data_olive_oil_experiment_sp4 46](#_1yfhjkcw10vs)

[S5.3. External function for intensity decay fit: fit_IR 47](#_po46lz1q0kjw)

[S5.4. Integral plots via intensity_fit_pseudo2D 49](#_pu1cgdpeayvk)

[S5.5. Input files for model_fit_pseudo2D 49](#_m3uowmjpr1uc)

[S5.6. Result folder of model fit pseudo2D function 50](#_l2u9ea295nmd)

[S5.7. Output file for model_fit_pseudo2D - data_olive_oil_experiment_sp4 58](#_813aiemvnl9f)

[**S6. FDX2 longitudinal relaxation rates fit 60**](#_x1crebhpmuc7)

[S6.1 Adapted target function for the regularization of IR intensity profiles in FDX fit 60](#_9dsujfqbsxwz)

[S6.2 IR intensity profiles from TRAGICO 64](#_s5d6ofzg2555)

[S6.3 IR intensity profiles from Topspin 67](#_r7e3mqwjpatp)

[S6.4 T1-encoded 13C FDX2 spectra 70](#_qeb3x0ipf0im)

[**S7. Analysis of relaxation measurements at variable magnetic field 71**](#_p9te3lctttjf)

[S7.1 Code listing for intensity analysis 71](#_70lktxsokptz)

[S7.2 Code listing for spectra modeling 72](#_7sljnbjg7dhd)

#

#

# S1. Additional information on TrAGICo implementation

## S1.1 Arguments and description of the main routines in TrAGICo script

| Table S1.1: Functions and argument of the main routines saved in f_fit.py. A more detailed description of the function's arguments is reported in the documentation available at: <https://github.com/letiziafiorucci/tragico/tree/main/docs>. | |
| --- | --- |
| function name  [brief description] | arguments (= default value), description |
| intensity_fit_  pseudo2D  [performs analysis of intensity trends in series (or single) pseudo-2D NMR data] | path, path to the folder collecting the experiments to be analyzed.  delays_list, list of floats or integers that identify the different transients.  list_path, list of strings representing the names of the folders inside path.  prev_lims = False, boolean, if True the same regions are used for all the pseudo2Ds.  prev_coeff = False, boolean, if True the same baseline coefficients are used for every pseudo2D. By construction, the same baseline is used for all the transients inside a single pseudo2D. Alternatively, a string can be passed, that represents the name of the text file where the baseline coefficient values are saved (one row of coefficients for each interval).  area=False, boolean, if True the computed intensities correspond to the trapezoidal integral of the selected region, otherwise the highest point in the region is selected.  VCLIST=None, list of floats or integers that identifies the different pseudo2Ds. These values can be directly read by the title of the experiment (if None is passed).  cal_lim = None, tuple of floats or integers, if None is passed the spectra are not calibrated, alternatively the calibration region has to be passed like (ppm1, ppm2).    baseline=False, boolean, if True the baseline is subtracted from the intensity.    delta=0, float or integer that represents the +/- ppm that are used to enlarge the window shown in the graphical interface and in the figures.  doexp=False, boolean, if True the intensity trends are fitted with an exponential function, otherwise the intensity trend is reported without any fit (if also the following argument is set to None).    f_int_fit=None, a function can be passed and used to fit the intensity trend.  fargs=None, the tuple of arguments used by f_int_fit, besides x and y.  fig_stack=True, boolean, if True produces figures of the selected region across each pseudo2D.  fileinp=”inp1_pseudo2D”, string, representing the filename of the file where the limits, in ppm, of the spectral regions are reported (one row for each interval). If does not exist it will be written with this name.  err_lims_out=None, tuple of floats or integers, representing the limits (ppm1, ppm2) of the region that will be used for error evaluation.  color_map=”viridis”, string, colormap for the fig_stack plots. |
| intensity_fit_  1D  [performs analysis of intensity trends in series (or single) 1D NMR data] | path, path to the folder collecting the experiments to be analyzed.  delays_list, list of floats or integers that identify the different transients.  list_path, list of strings representing the names of the folders inside path.  area=False, boolean, if True the computed intensities correspond to the trapezoidal integral of the selected region, otherwise the highest point in the region is selected.  cal_lim = None, tuple of floats or integers, if None is passed the spectra are not calibrated, alternatively the calibration region has to be passed like (ppm1, ppm2).    baseline=False, boolean or string, if True the baseline is subtracted from the intensity and the baseline coefficients are determined via graphical interface, if False the baseline is not generated and, if string it indicates the path to a txt file containing the baseline coefficients that should be used for each interval..    delta=0, float or integer that represents the +/- ppm that are used to enlarge the window shown in the graphical interface and in the figures.  doexp=False, boolean, if True the intensity trends are fitted with an exponential function, otherwise the intensity trend is reported without any fit (if also the following argument is set to None).    f_int_fit=None, a function can be passed and used to fit the intensity trend.  fargs=None, the tuple of arguments used by f_int_fit, besides x and y.  Spectra=None, list of numpy arrays of complex numbers or floats, that can be used alternatively to the one read from the path files. This can be used e.g. to treat simulated data or data from alternative formats.  ppmscale=None, numpy array of floats, frequency scale of the Spectra data.  fig_stack=True, boolean, if True produces figures of the selected region across the series of 1D spectra.  fileinp=”inp1_1D”, string, representing the filename of the file where the limits, in ppm, of the spectral regions are reported (one row for each interval). If does not exist it will be written with this name.  err_lims_out=None, tuple of floats or integers, representing the limits (ppm1, ppm2) of the region that will be used for error evaluation.  color_map=”viridis”, string, colormap for the fig_stack plots. |
| model_fit_  pseudo2D  [performs analysis of peak parameters trends, derived from modeling of the experimental lines, in series (or single) pseudo-2D NMR data] | path, path to the folder collecting the experiments to be analyzed.  delays_list, list of floats or integers that identify the different transients.  list_path, list of strings representing the names of the folders inside path.  cal_lim = None, tuple of floats or integers, if None is passed the spectra are not calibrated, alternatively the calibration region has to be passed like (ppm1, ppm2).  dofit=True, boolean, if True the fit of the spectra is performed, otherwise the figures and analysis of trends are executed directly.  prev_guess=False, boolean, if True the parameters’ initial guess for the first transient is the same as those defined by the user.  prev_fit=None, string, represents the directory where files from a previous fit have been saved and can be used when dofit=False. If the directory is empty and/or dofit=True then the files will be saved in the result folder and in this additional directory. If None, then the files will be saved only in the result directory.  file_inp1=None, string, name of the first input file. If None, the program will ask the user for that from the terminal.  file_inp2=None, string, name of the second input file. If None, the program will ask the user for that from the terminal.  fast=False, boolean, if True the least squares minimization algorithm available in lmfit will be used in the fit, otherwise a combination of Nelder-Mead and least squares minimization procedures will be used, one after the other.  limits1 = None, dictionary with (min value, max value) for the different variables used in the fit of the first transient of the pseudo2D.  limits2 = None, dictionary of the variation intervals to be applied to the variables fitted from the previous transient in the psedu2D to be used in the fit of the subsequent transient.  L1R = None, float or integer, that represents the scaling factor for the L_1_ regularization criterium.  L2R = None, float or integer, that represents the scaling factor for the L_2_ regularization criterium.  err_conf=0.95, float, the fraction of the peak area that has to be included in the error estimation region.  doexp=False, boolean, if True the intensity trends are fitted with an exponential function, otherwise the intensity trend is reported without any fit (if also the following argument is set to None).    f_int_fit=None, a function can be passed and used to fit the intensity trend.  fargs=None, the tuple of arguments used by f_int_fit, besides x and y. |
| model_fit_1D  [performs analysis of peak parameters trends, derived from modeling of the experimental lines, in series (or single) 1D NMR data] | path, path to the folder collecting the experiments to be analyzed.  delays_list, list of floats or integers that identify the different transients.  list_path, list of strings representing the names of the folders inside path.  cal_lim = None, tuple of floats or integers, if None is passed the spectra are not calibrated, alternatively the calibration region has to be passed like (ppm1, ppm2).  dofit=True, boolean, if True the fit of the spectra is performed, otherwise the figures and analysis of trends are executed directly.  prev_fit=None, string, represents the directory where files from a previous fit have been saved and can be used when dofit=False. If the directory is empty and/or dofit=True then the files will be saved in the result folder and in this additional directory. If None, then the files will be saved only in the result directory.  file_inp1=None, string, name of the first input file. If None, the program will ask the user for that from the terminal.  file_inp2=None, string, name of the second input file. If None, the program will ask the user for that from the terminal.  fast=False, boolean, if True the least squares minimization algorithm available in lmfit will be used in the fit, otherwise a combination of Nelder-Mead and least squares minimization procedures will be used, one after the other.  limits1 = None, dictionary with (min value, max value) for the different variables used in the fit of the first spectrum.  limits2 = None, dictionary of the variation intervals to be applied to the variables fitted from the previous spectrum to be used in the fit of the subsequent spectrum in the series.  L1R = None, float or integer, that represents the scaling factor for the L_1_ regularization criterium.  L2R = None, float or integer, that represents the scaling factor for the L_2_ regularization criterium.  err_conf=0.95, float, the fraction of the peak area that has to be included in the error estimation region.  doexp=False, boolean, if True the intensity trends are fitted with an exponential function, otherwise the intensity trend is reported without any fit (if also the following argument is set to None).    f_int_fit=None, a function can be passed and used to fit the intensity trend.  fargs=None, the tuple of arguments used by f_int_fit, besides x and y.  Spectra=None, list of numpy arrays of complex numbers or floats, that can be used alternatively to the one read from the path files. This can be used e.g. to treat simulated data or data from alternative formats.  ppmscale=None, numpy array of floats, frequency scale of the Spectra data.  acqupars=None, dictionary with acquisition parameters for Spectra. The keys have to be the same as those of an equivalent Bruker spectrum, i.e. “DE”, “SW”, “TD”, “o1” and, “SFO1”.  procpars=None, dictionary with processing parameters for Spectra. The keys have to be the same as those of an equivalent Bruker spectrum, i.e. “SR”, “SI”, “LB” and, “SSB”.  Param=None, string, the name of the variable that will be returned and written in the output files, besides the intensities, i.e. “shift”, “lw”, “k”, “xg” and, “phi”. |

## S1.2 TRAGICO routines

| Listing S1.2.1: Cost function implementation in TrAGICo. |
| --- |
| def fit_peaks_bsl_I([function arguments]):    cal = SR/sf1 - (ppm_scale[0]-ppm_scale[1])    def f_residue(param, ppm_scale, spettro, tensor_red, result=False):  par = param.valuesdict()  lor_list = []  lor_ph0_list = []  comp_list = []  prev=0  mult = tensor_red[:,-1]  sx, dx, zero = find_limits(tensor_red[0,1], tensor_red[0,2], ppm_scale)  sim_spectra = np.zeros_like(spettro, dtype='complex128')  sim_fid = np.zeros_like(spettro, dtype='complex128')    for ii in range(tensor_red.shape[0]):  if tensor_red[ii,0]=='true':  lor = t_voigt(t_aq, (par['shift_'+str(ii+1)]+cal-o1p)*sf1, 2*np.pi*par['lw_'+str(ii+1)]*sf1,  A=par['k_'+str(ii+1)], phi=par['ph_'+str(ii+1)], x_g=par['xg_'+str(ii+1)])  else:  lor = t_voigt(t_aq, (par['shift_'+str(ii+1)+'_f']+cal-o1p)*sf1, 2*np.pi*par['lw_'+str(ii+1)+'_f']*sf1,  A=par['k_'+str(ii+1)+'_f'], phi=par['ph_'+str(ii+1)+'_f'], x_g=par['xg_'+str(ii+1)+'_f'])    ### processing  lor *= em(lor, LB, SW)  lor *= qsin(lor, SSB)  ###  if result:  [computation of phase=0 spectrum]  sim_fid += lor.copy()  lor = ft(lor, SI, dw, o1p, sf1)[0]  sim_spectra += np.conj(lor)[::-1]  lor = np.conj(lor)[::-1].real  [check for Nan]  comp_list.append(lor[sx:dx])    if result:  [computation of phase=0 spectrum]  x = ppm_scale[sx:dx]-zero  corr_baseline = par['E']*x**4 + par['D']*x**3 + par['C']*x**2 + par['B']*x + par['A']  cost = np.max(spettro.real)    if result:  lor_ph0_list = np.array(lor_ph0_list)*cost  lor_list = np.array(lor_list)*cost  comp_list = np.array(comp_list)*cost  model = cost*(corr_baseline+sim_spectra[sx:dx].real)  res = model.real-spettro[sx:dx].real  res2plot = res.copy()  if L1R is not None:  sum_param = np.sum(np.array([np.abs(par[key]) for key in par.keys()]))/np.max(spettro.real)  res += L1R*sum_param  elif L2R is not None:  sum_param = np.sum(np.array([par[key]**2 for key in par.keys()]))/np.max(spettro.real)  res += L2R*sum_param  else:  pass  [figures generation]  if not result:  residuals = res  x = ppm_scale  corr_baseline = par['E']*x**4 + par['D']*x**3 + par['C']*x**2 + par['B']*x + par['A']  [check for Nan]  return residuals  else:    [return statement]      minner = lmfit.Minimizer(f_residue, param, fcn_args=(ppm_scale, spettro, tensor_red))  if dofit:  if not fast:  result = minner.minimize(method='Nelder', max_nfev=10000)  params = result.params  result = minner.minimize(params=params, method='leastsq', max_nfev=10000)  else:  result = minner.minimize(method='leastsq', max_nfev=5000)  else:  result = minner.minimize(method='Nelder', max_nfev=0)  popt = result.params    [return statement] |

| Listing S1.2.2: Routine used for the calibration of the spectra in TrAGICo. |
| --- |
| def calibration(ppm_scale, data, ppmsx, ppmdx, npoints=80, debug_fig=False):  """  Calibrates the spectra in 'data' with respect to the first one.  The calibration is performed by shifting the spectra in 'data' with respect to the first one.  The shift is calculated by minimizing the residue between the first spectrum and the others.  --------  Parameters:  - ppm_scale : 1darray  scale of the ppm axis  - data : 2darray  matrix with the spectra to be calibrated  - ppmsx : float  left limit of the calibration region  - ppmdx : float  right limit of the calibration region  - npoints : int  number of points to be used for the calibration  - debug_fig : bool  if True, shows a figure with the calibration process  -------  Returns:  - shift_cal : 1darray  array with the shifts in points  - shift_cal_ppm : 1darray  array with the shifts in ppm  - data_roll : 2darray  matrix with the calibrated spectra  """  print('Performing calibration...')  normalization = np.max(data[0,:])  def residue(param, ppm_scale, spettro0, spettro1, sx, dx, risultato=False):  par = param.valuesdict()  roll_spettro1 = np.roll(spettro1, int(par['shift']))  if not risultato:  res = np.abs(spettro0.real/normalization)-np.abs(roll_spettro1.real/normalization)  return res[npoints:-npoints]  else:  if debug_fig:  fig = plt.figure()  fig.set_size_inches(5.59,4.56)  plt.subplots_adjust(left=0.15,bottom=0.15,right=0.95,top=0.90)  ax = fig.add_subplot(1,1,1)  ax.tick_params(labelsize=6.5)  ax.plot(ppm_scale[sx:dx], spettro0.real/normalization, lw=0.5, label='spectra_0')  ax.plot(ppm_scale[sx:dx], roll_spettro1.real/normalization, lw=0.5, label='spectra_1')  ax.plot(ppm_scale[sx:dx], spettro1.real/normalization, lw=0.5, label='spectra_1 prima')  ax.set_xlabel(r'$\delta \, ^1$H (ppm)', fontsize=8)  ax.set_ylabel('Intensity (a.u.)', fontsize=8)  ax.ticklabel_format(axis='y', style='scientific', scilimits=(-2,2), useMathText=True)  ax.yaxis.get_offset_text().set_size(7)  ax.invert_xaxis()  ax.legend(fontsize=6)  plt.show()  return par['shift']  sx,dx,_ = find_limits(ppmsx, ppmdx, ppm_scale)  spettro0 = data[0,sx:dx]    if npoints*2>len(spettro0):  npoints = len(spettro0)//2    shift_cal = []  shift_cal_ppm = []  for i in range(data.shape[0]):  if i!=0:  param = lmfit.Parameters()  param.add('shift', value=0, max=npoints, min=-npoints)  param['shift'].set(brute_step=1)  spettro1 = data[i,sx:dx]  minner = lmfit.Minimizer(residue, param, fcn_args=(ppm_scale, spettro0, spettro1, sx, dx))  result = minner.minimize(method='brute', max_nfev=1000) popt = result.params  # print('Fit report')  # print(lmfit.fit_report(result))  shift = residue(popt, ppm_scale, spettro0, spettro1, sx, dx, risultato=True)  shift_cal.append(int(shift))  shift_cal_ppm.append(shift*(ppm_scale[0]-ppm_scale[1]))  else:  shift_cal.append(0)  shift_cal_ppm.append(0)  print('...done')  data_roll = np.zeros_like(data, dtype='float64')  for i in range(data.shape[0]):  data_roll[i,:] = np.roll(data[i,:], shift_cal[i])  return shift_cal, shift_cal_ppm, data_roll |

# S2. Chemical shift temperature dependence and calibration

## S2.1. AU program for the acquisition of a series of spectra at increasing temperatures

| int startExpno;  int expTime;  extern void PrintExpTime();  GETCURDATA;  startExpno = expno;  if (strlen(cmd) == 0)  {  i1=10;  GETINT("Enter number of experiments : ",i1);  }  else  {  if (1 != sscanf(cmd, "%d", &i1))  {  STOPMSG("illegal input");  }  }  expTime = 0;  TIMES(i1)  SETCURDATA;  expTime += CalcExpTime() + 210;  IEXPNO;  END  DEXPNO;  PrintExpTime(expTime, i1);  (void) unlink ("/tmp/expt");  expno = startExpno;  SETCURDATA;  TIMES(i1)  (void) sprintf(text,"running experiment # %d",loopcount1+1);  Show_status(text);  sleep(30);  TESET;  TEREADY(180,0.05);  ZG;  IEXPNO;  END  DEXPNO;  QUITMSG("--- multizg finished ---");  #include <inc/exptUtil>/* utilities for printing and calculation of  experiment time */ |
| --- |

## S2.2. Output file for MeOH spectra

A section of the output file obtained for the fit of the series of monodimensional spectra for the study of chemical shifts temperature dependence of NiSAL-HDPT sample is reported below.

SPECTRA PATH:

path/to/MeOH_tempcal_100122/

Points:

1 299.200

2 299.010

3 298.810

4 298.610

5 298.420

6 298.220

7 298.100

8 298.000

9 297.830

10 297.630

11 297.430

12 297.230

13 297.030

INPUT1: inp_1

n. peak name ppm1 ppm2 v mult

1 true 3.46600 3.21700 3.32981 0

2 true 4.90600 4.81000 4.86346 0

INPUT2: inp_2

n. peak i ppm1 ppm2 k fwhm phi xg A B C D E

1 0 3.5 3.2 4.34583e-03 3.67081e-03 0.00000e+00 2.00000e-01 0.000e+00 0.000e+00 0.000e+00 0.000e+00 0.000e+00

2 0 4.9 4.8 2.46078e-03 2.89195e-03 0.00000e+00 2.00000e-01 0.000e+00 0.000e+00 0.000e+00 0.000e+00 0.000e+00

====================================================================

====================================================================

(I: fit interval, P: N. point)

------------------------------

FIT RANGE: (3.47:3.22) ppm

I: 1 P: 1

------------------------------

Fit Report:

[[Fit Statistics]]

# fitting method = leastsq

# function evals = 43

# data points = 4037

# variables = 5

chi-square = 1.5983e+15

reduced chi-square = 3.9640e+11

Akaike info crit = 107815.816

Bayesian info crit = 107847.332

[[Variables]]

shift_1: 3.32931441 +/- 1.3143e-06 (0.00%) (init = 3.32981)

k_1: 0.00468964 +/- 1.6972e-06 (0.04%) (init = 0.00434583)

lw_1: 0.00534645 +/- 3.8006e-06 (0.07%) (init = 0.00367081)

ph_1: 0.01242673 +/- 2.9255e-04 (2.35%) (init = 0)

xg_1: 0.56584136 +/- 0.00144026 (0.25%) (init = 0.2)

A: 0 (fixed)

B: 0 (fixed)

C: 0 (fixed)

D: 0 (fixed)

E: 0 (fixed)

[[Correlations]] (unreported correlations are < 0.100)

C(lw_1, xg_1) = +0.7690

C(shift_1, ph_1) = -0.7546

C(k_1, xg_1) = -0.7448

C(k_1, lw_1) = -0.3079

n.peak Shift Integral

1 3.329 54936449643.506 +/- 435237381.724

[...]

------------------------------

FIT RANGE: (4.91:4.81) ppm

I: 2 P: 13

------------------------------

Fit Report:

[[Fit Statistics]]

# fitting method = leastsq

# function evals = 22

# data points = 1557

# variables = 2

chi-square = 7.3990e+14

reduced chi-square = 4.7582e+11

Akaike info crit = 41867.0851

Bayesian info crit = 41877.7861

[[Variables]]

shift_1: 4.88377336 +/- 1.2943e-06 (0.00%) (init = 4.881838)

k_1: 0.00245823 +/- 9.1703e-07 (0.04%) (init = 0.002455608)

lw_1: 0.003017795 (fixed)

ph_1: 0.02317096 (fixed)

xg_1: 0.1391737 (fixed)

A: 0 (fixed)

B: 0 (fixed)

C: 0 (fixed)

D: 0 (fixed)

E: 0 (fixed)

n.peak Shift Integral

1 4.884 28838896004.238 +/- 493094857.812

## S2.3. Temperature calibration with MeOH

| Listing S2.3.1: Code listing for the generation of Figure 7. |
| --- |
| # STARTING FROM THE CODE LISTING IN THE MAIN TEXT  import matplotlib.pyplot as plt  delta_ppm = shift_tot[:,1]-shift_tot[:,0]  delta_ppm_err = np.sqrt(shift_tot_err[:,0]**2+shift_tot_err[:,1]**2)  temp_err = 0.025 # in K  temp_corr = 416.4745 - (38.5133)*delta_ppm - 36.0620*((delta_ppm)**2) + 11.4869*((delta_ppm)**3) - 2.4340*((delta_ppm)**4)  fig = plt.figure()  ax = fig.add_subplot(111)  fig.set_size_inches(4.,3.5)  ax.errorbar(temp_corr, delta_ppm, xerr=temp_err, yerr=delta_ppm_err, fmt='.', color='darkred', ecolor='m', elinewidth=0.2, capsize=2, capthick=0.2)  ax.set_xlabel('T$_{corr}$ (K)')  ax.set_ylabel('$\Delta \delta$ (ppm)')  plt.tight_layout()  plt.savefig('temp_corr.png', dpi=600)  plt.show()  np.savetxt('/home/methanol_modelfit/temp_corr.txt', temp_corr)  fig = plt.figure()  ax = fig.add_subplot(111)  fig.set_size_inches(4.,3.5)  #trend line  y = np.polyfit(temp, temp_corr, 1)  y = np.poly1d(y)  #write the equation of the line  ax.plot(temp, y(temp), '--', color='blue', label=f'y = {y[1]:.4f}x + {y[0]:.4f}')  ax.plot(temp, temp_corr, 'o', color='magenta')  ax.set_xlabel('T$_{set}$ (K)')  ax.set_ylabel('T$_{corr}$ (K)')  plt.tight_layout()  plt.savefig('temp_corr_trend.png', dpi=600)  plt.legend()  plt.show() |

## S2.4. Code listing for extraction of Ni-SAL-HDPT chemical shifts

| Listing S2.4.1: Code listing for fit of NiSAL-HDPT temperature series of spectra. |
| --- |
| from f_fit import *  import numpy as np  path = 'path/to/spectra/folder/Nisal'  num_sp = list(np.arange(10,22,1)) # in this case the experiments are enumerated from 10 to 21  list_sp = [str(i)+'/pdata/1' for i in num_sp]  temp = []  for idx in range(len(list_sp)):  _, _, ngdicp = nmr_spectra_1d(path+list_sp[idx])  temp.append(ngdicp['acqus']['TE1'])  temp = np.array(temp)  # shift (ppm), lw (ppm), x_g (adim.), k (adim.), ph (rad), A-B-C-D-E (a.u.)  lim1 = {'shift':(-1,1), 'lw':(1e-4,12), 'ph':(-np.pi/20,np.pi/20), 'D':(0,0), 'E':(0,0)}  lim2 = {'shift':(0.2,1.8), 'lw':(0,0), 'ph':(0,0), 'xg':(0,0)}  _, shift_tot, x = model_fit_1D(  path, # path of the main spectra  temp, # list of temperatures  list_sp, # list of folders  dofit=True, # if True, the spectra are fitted  fast = True,  limits1 = lim1,  limits2 = lim2,  Param='shift') |

## S2.5. Ni-SAL-HDPT fit result plots

| Figure S2.5.1: Fit result plots for NiSAL-HDPT sample ^1^H temperature series of spectra. | |
| --- | --- |
| 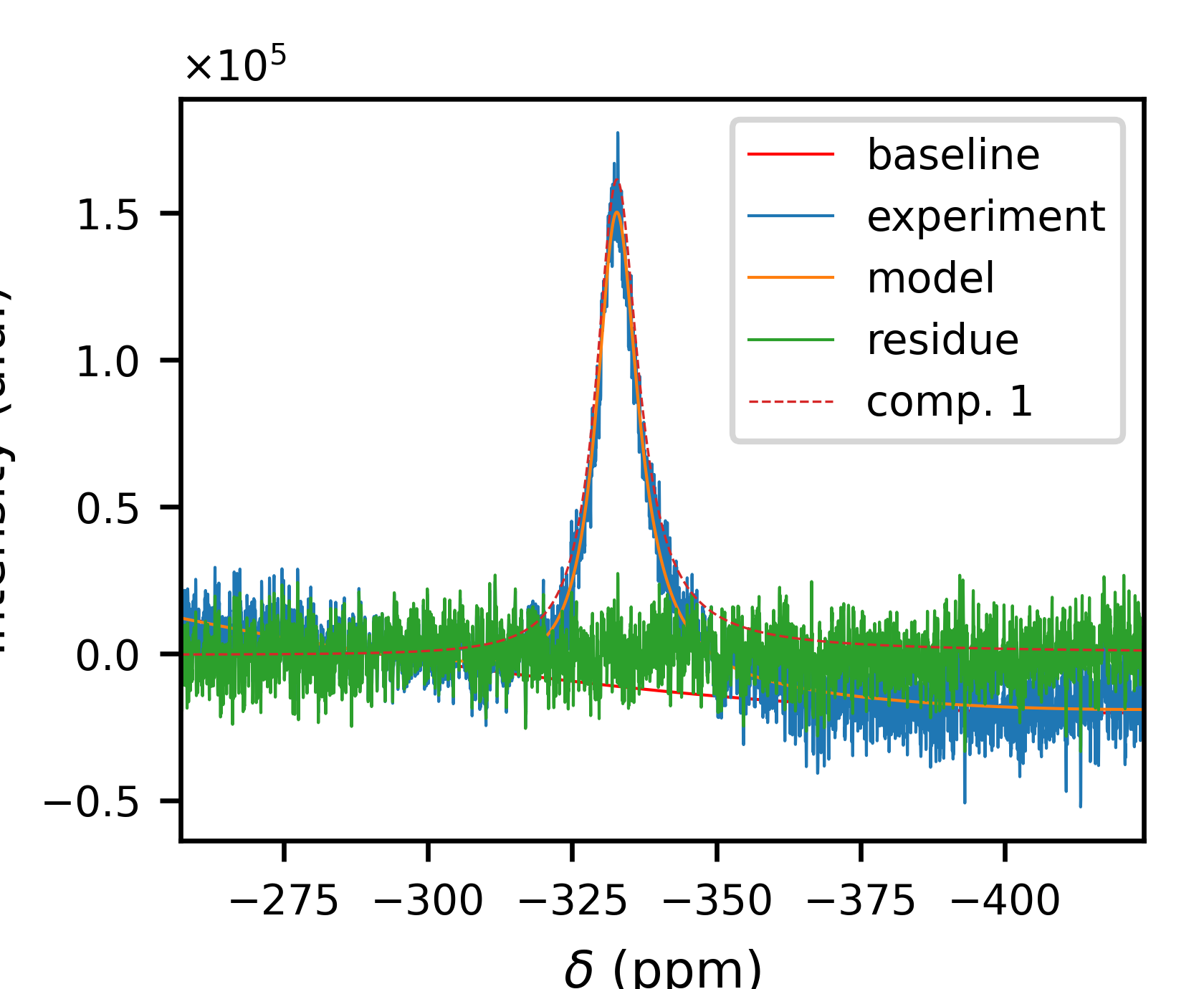 | 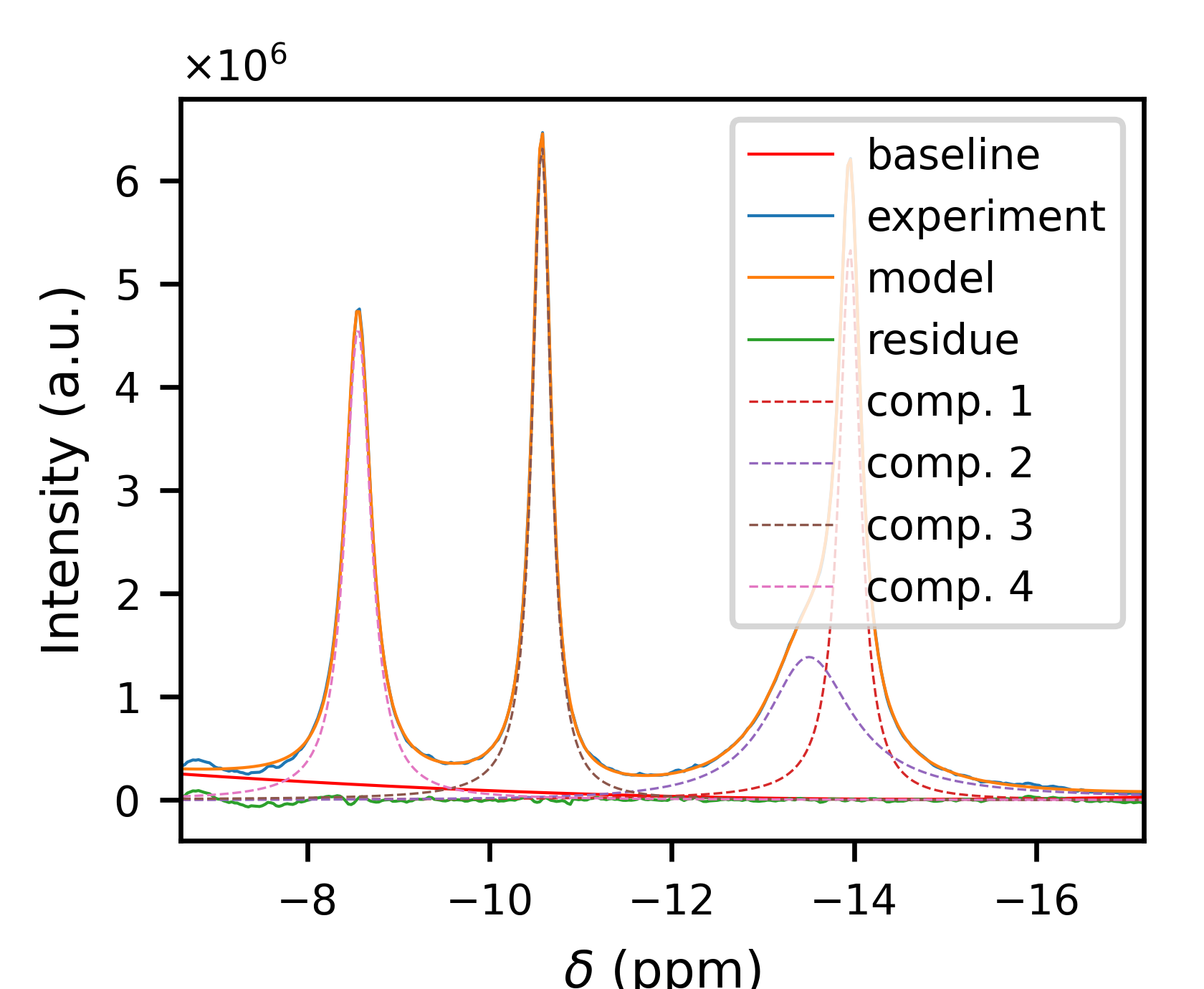 |
| 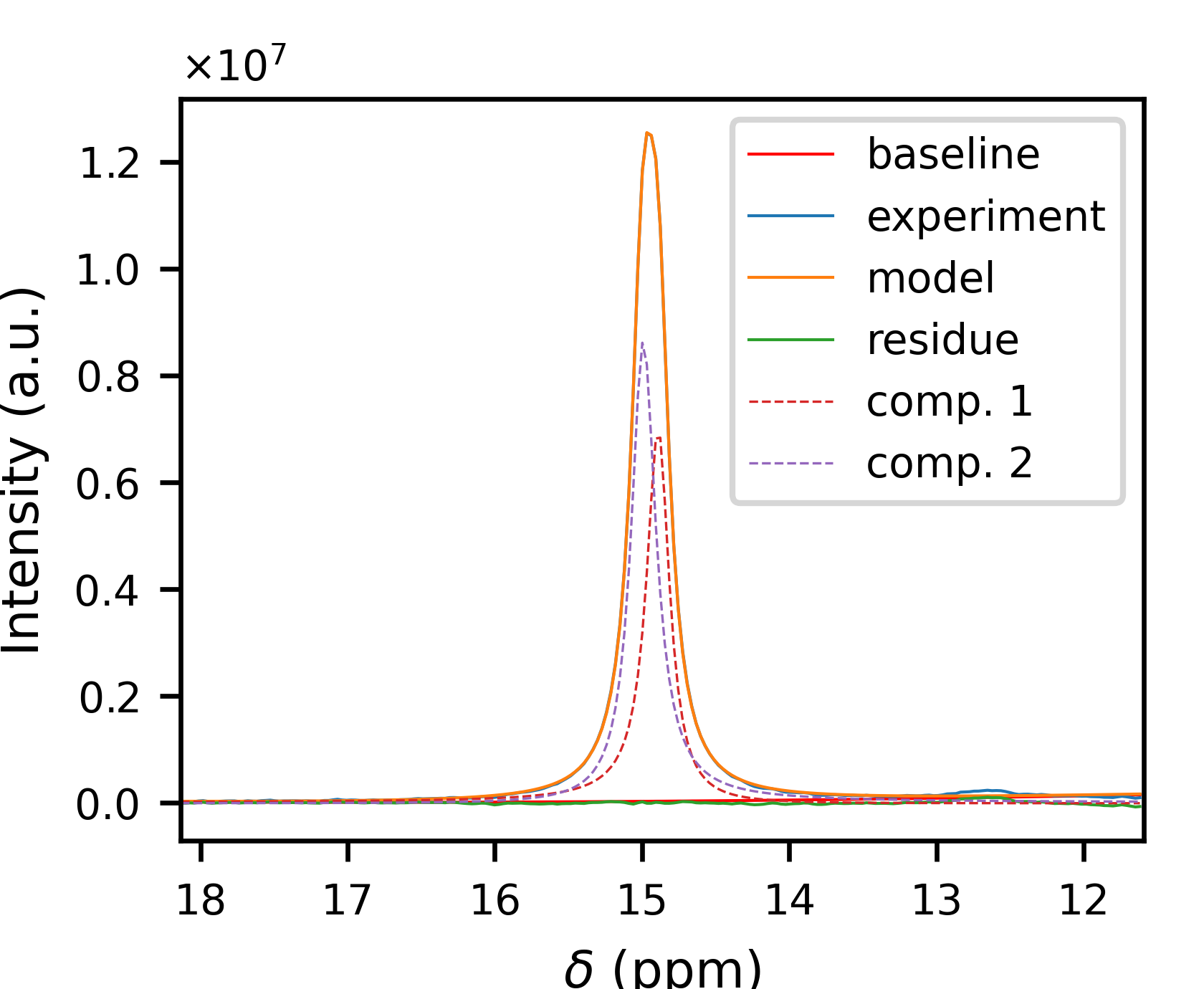 | 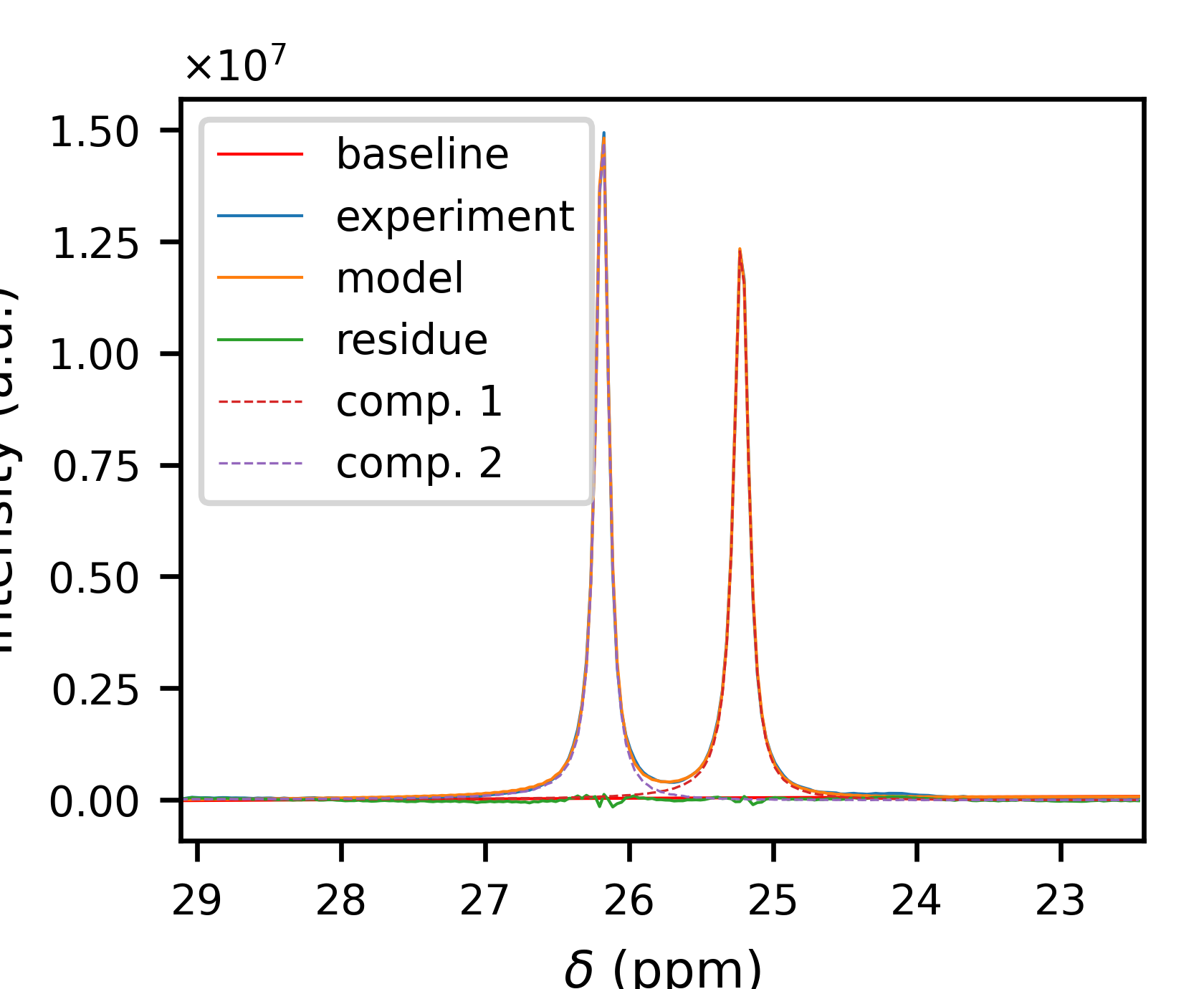 |
| 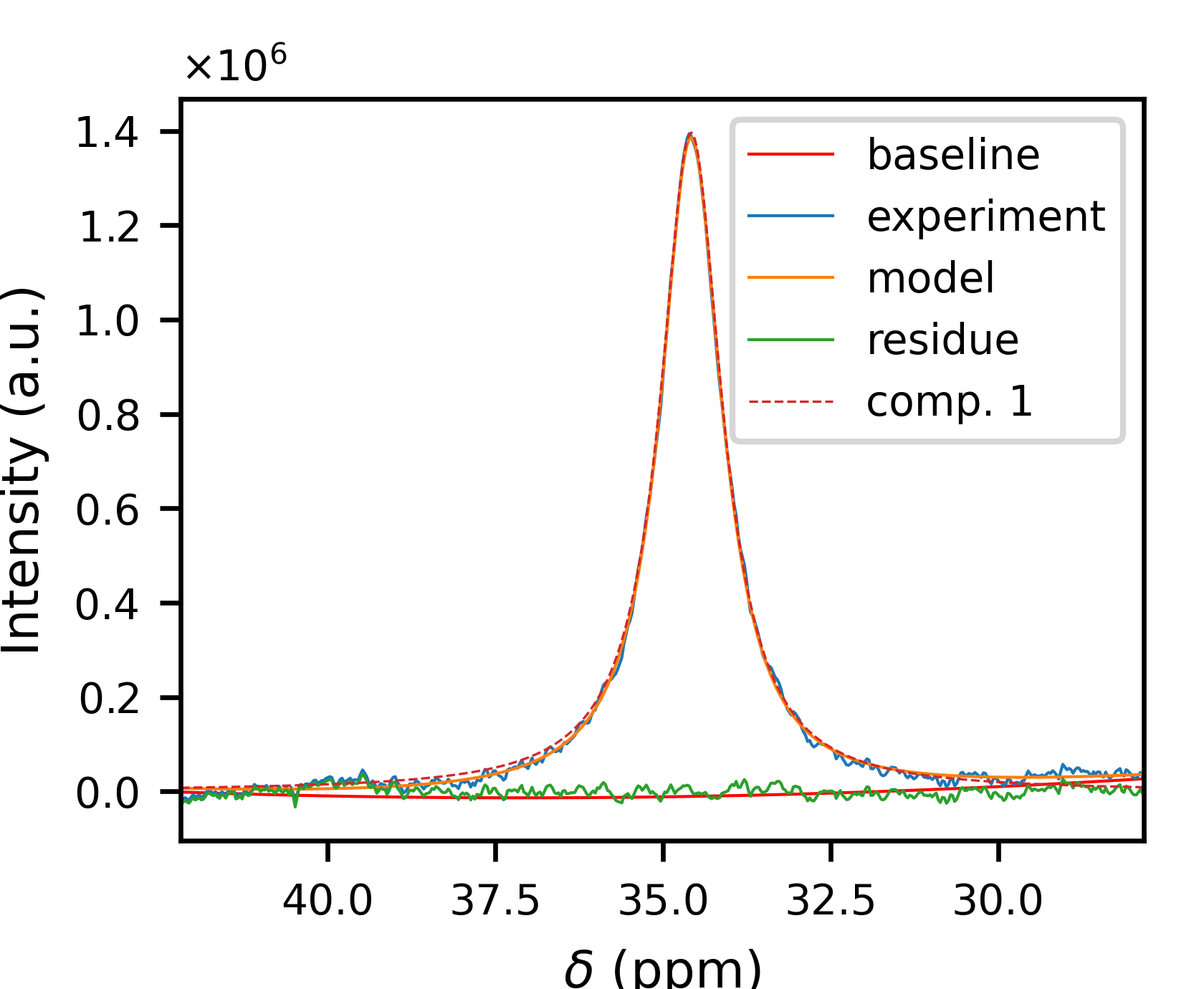 | 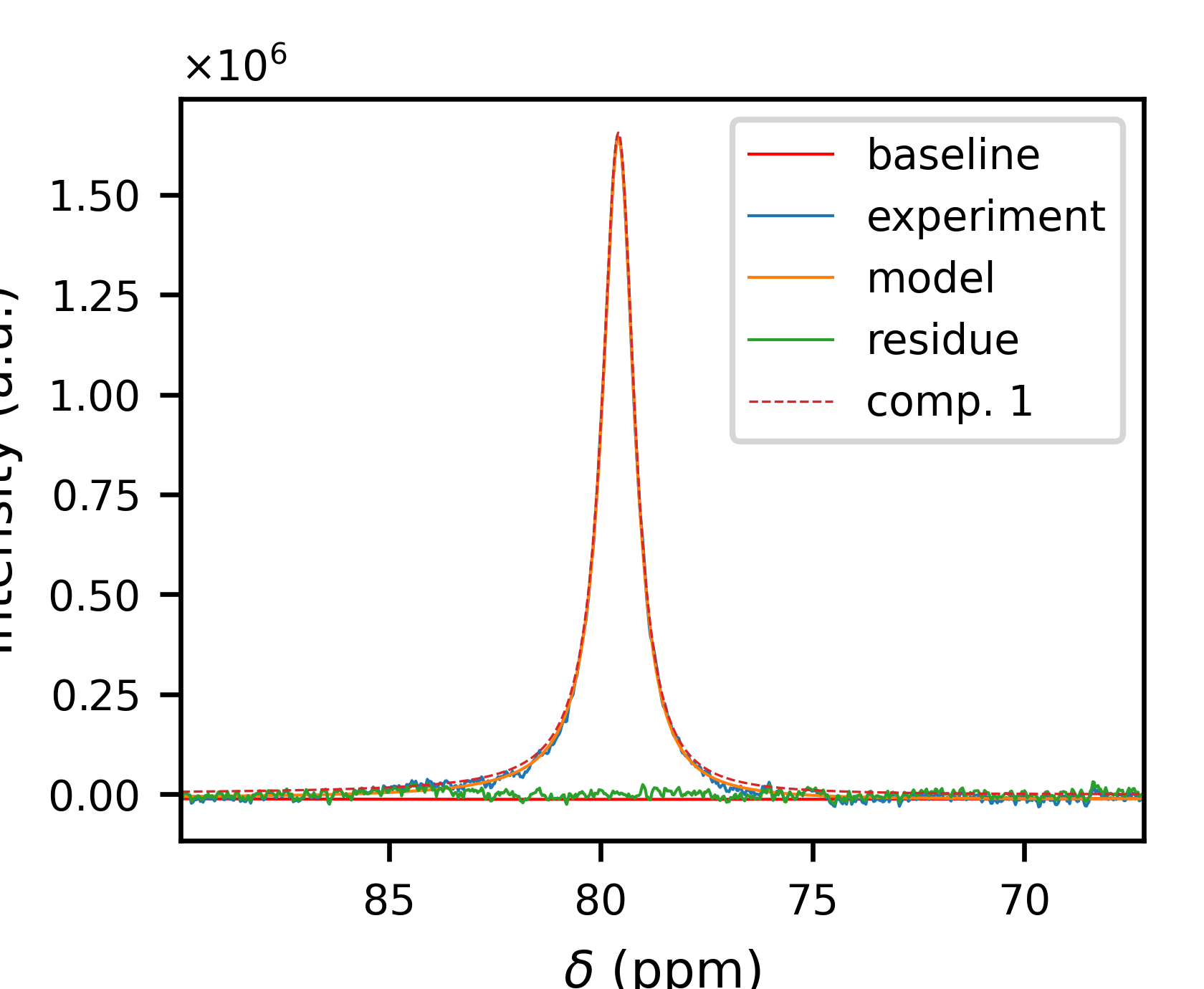 |
| 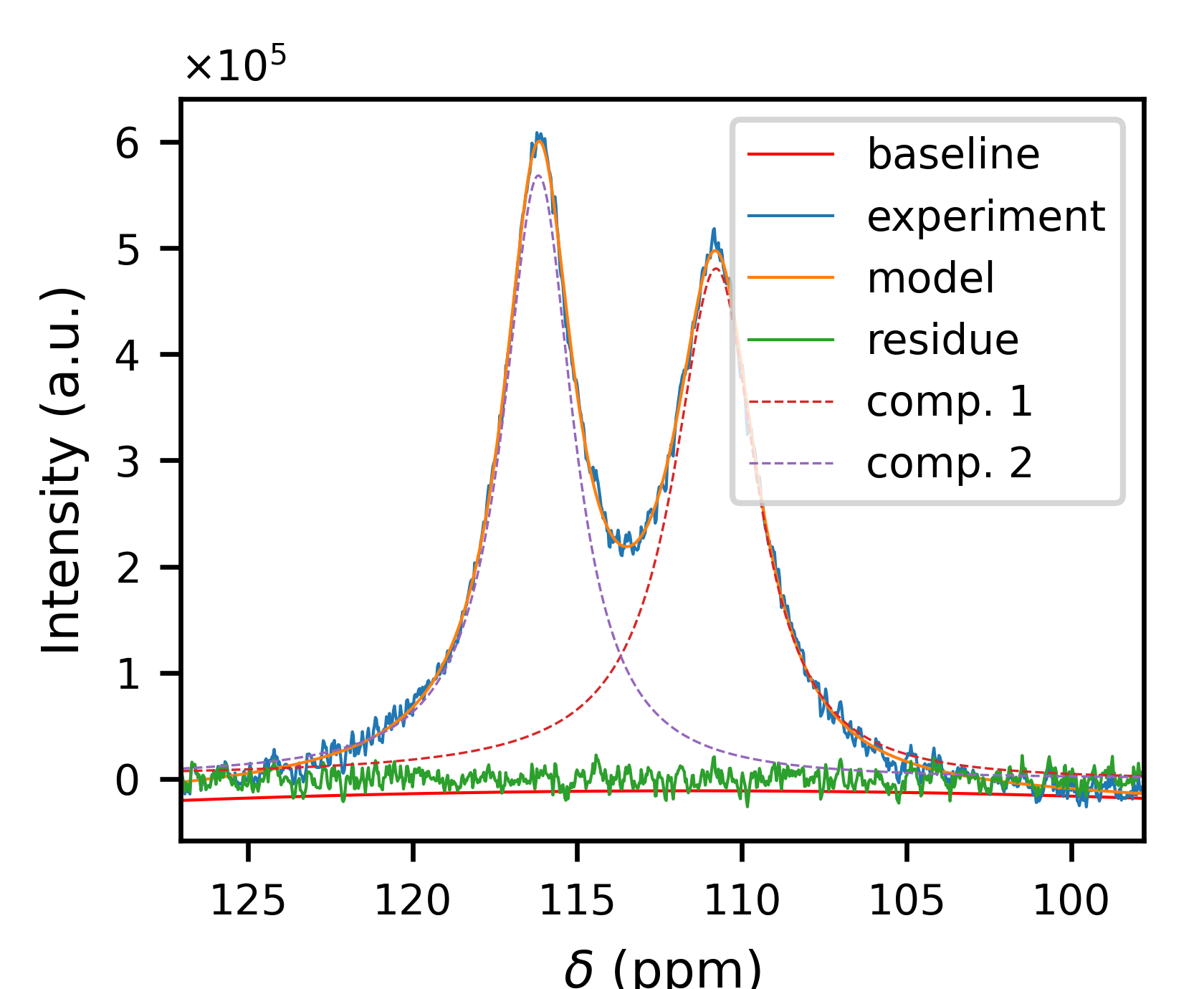 | 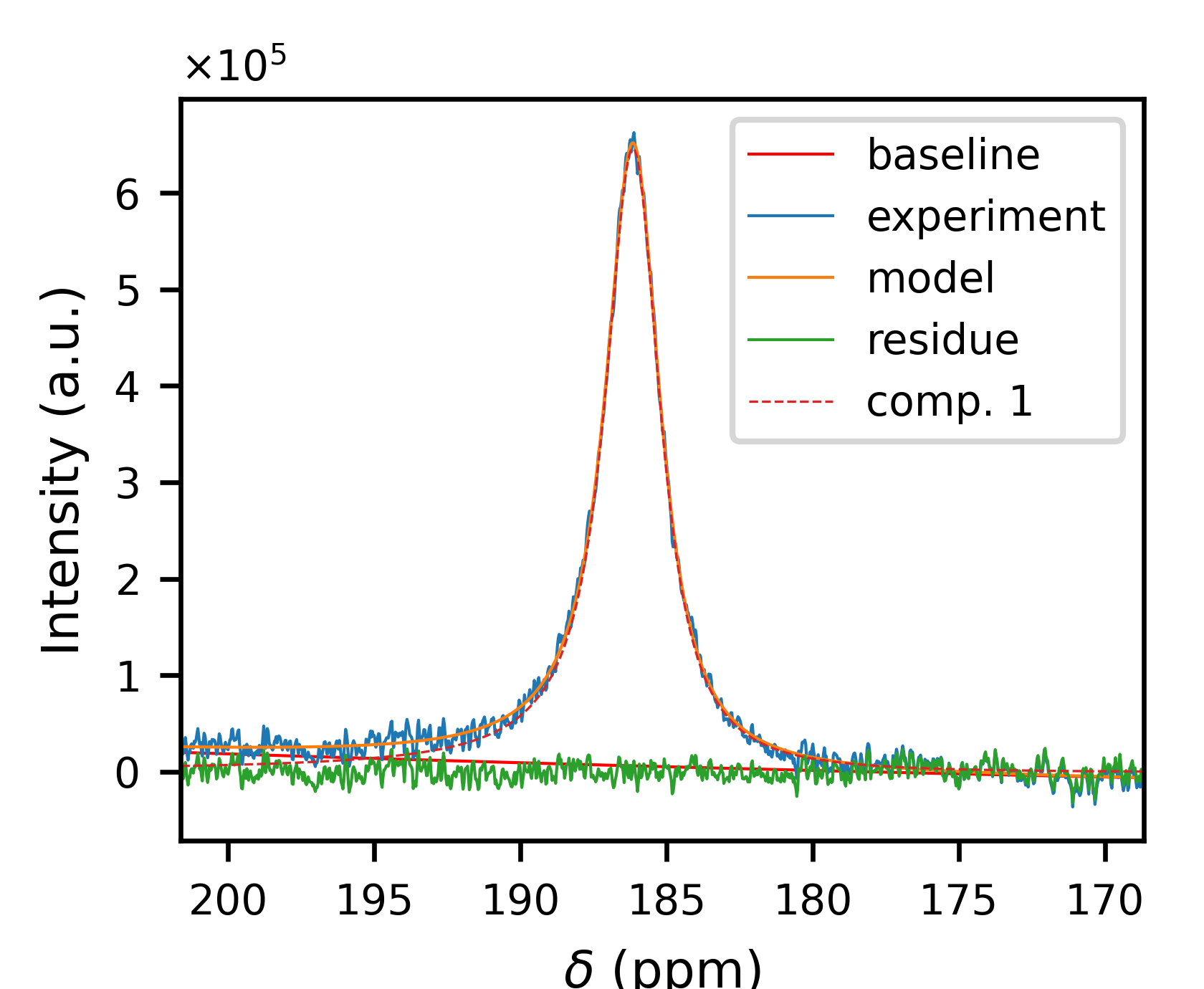 |
| 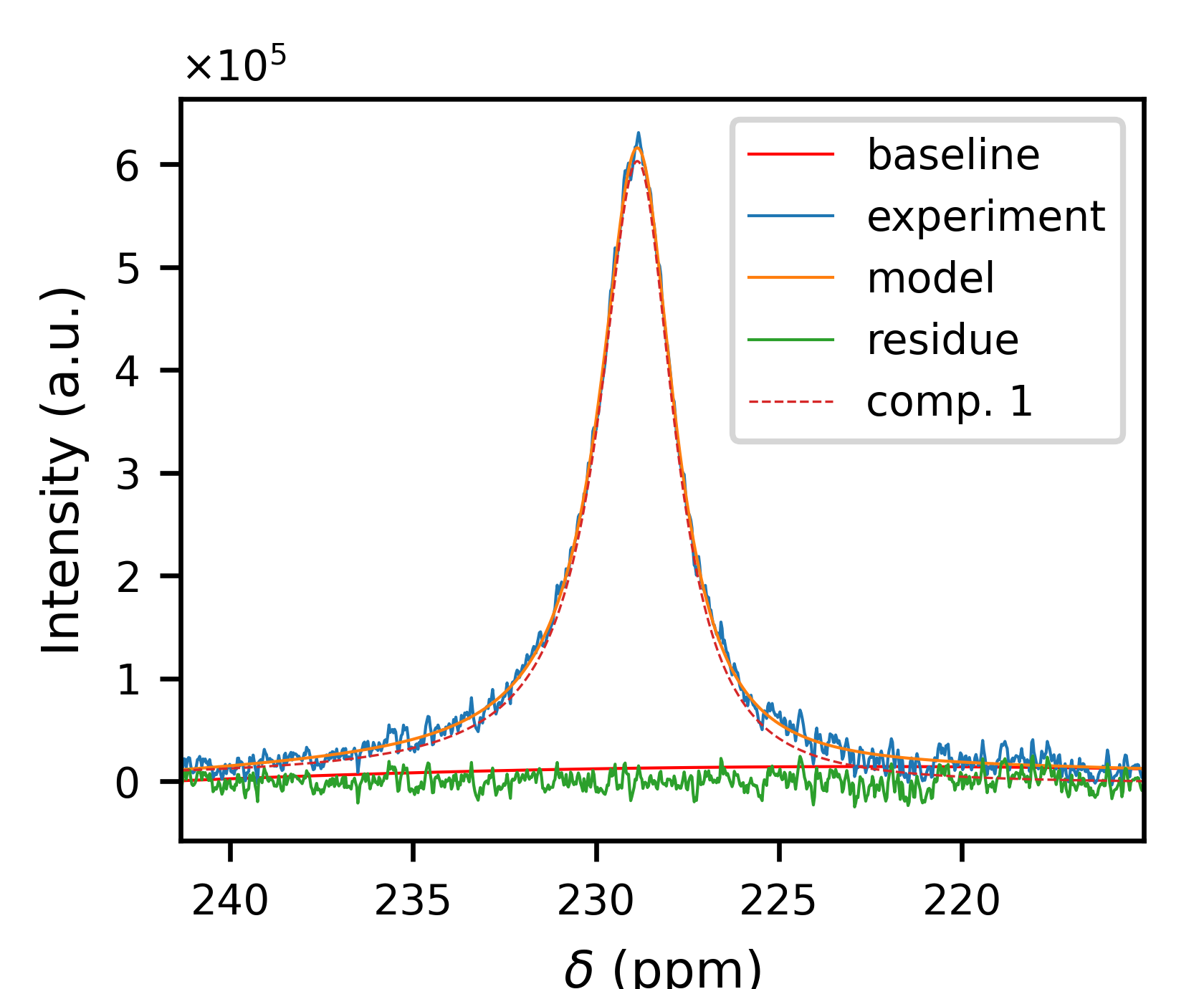 | 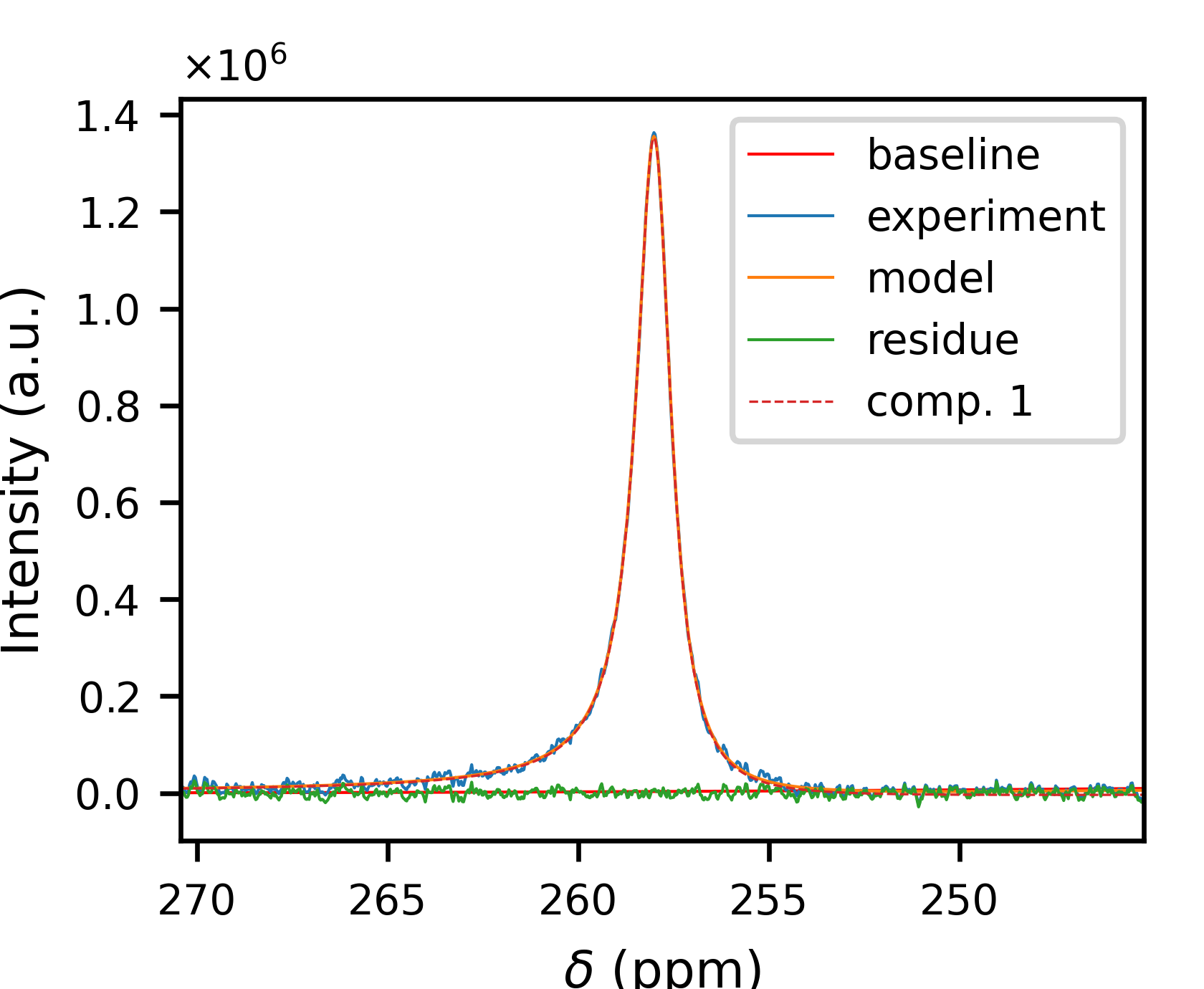 |
| 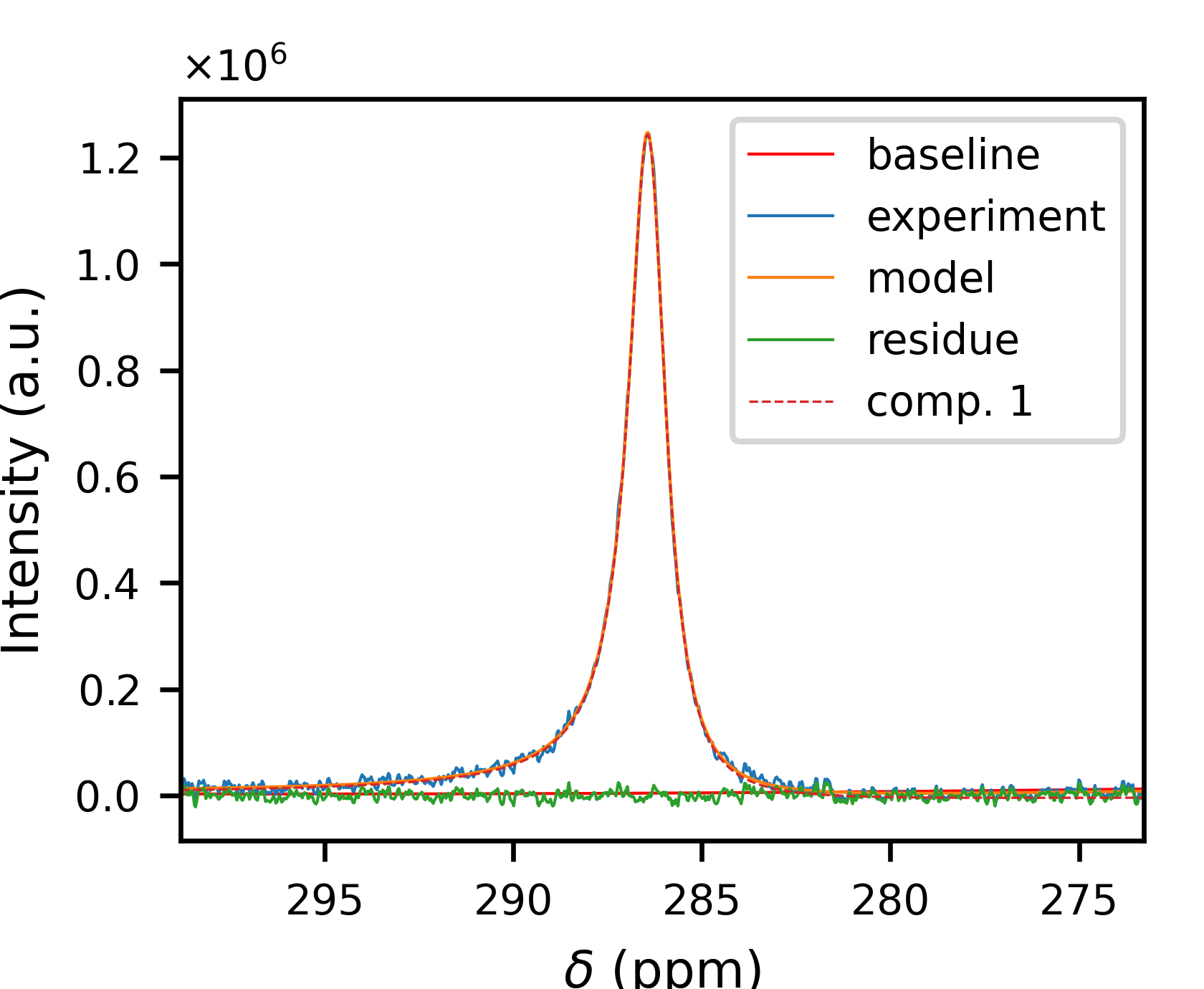 | 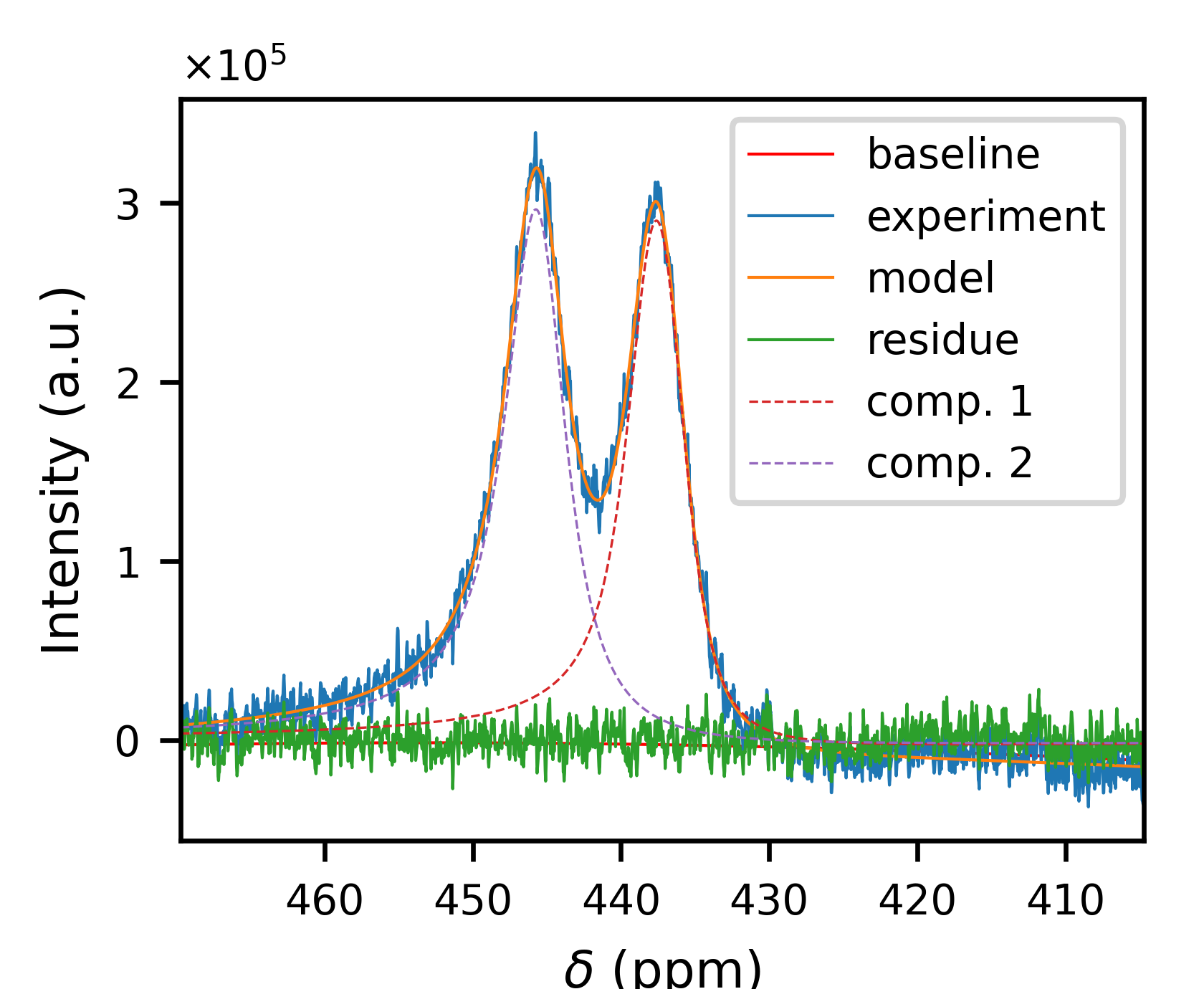 |

| Figure S2.5.2: Residuals histogram for NiSAL-HDPT sample temperature series of spectra modeling of figure S2.5.1. The order of the peaks is the same as figure S2.5.1. | |
| --- | --- |
| 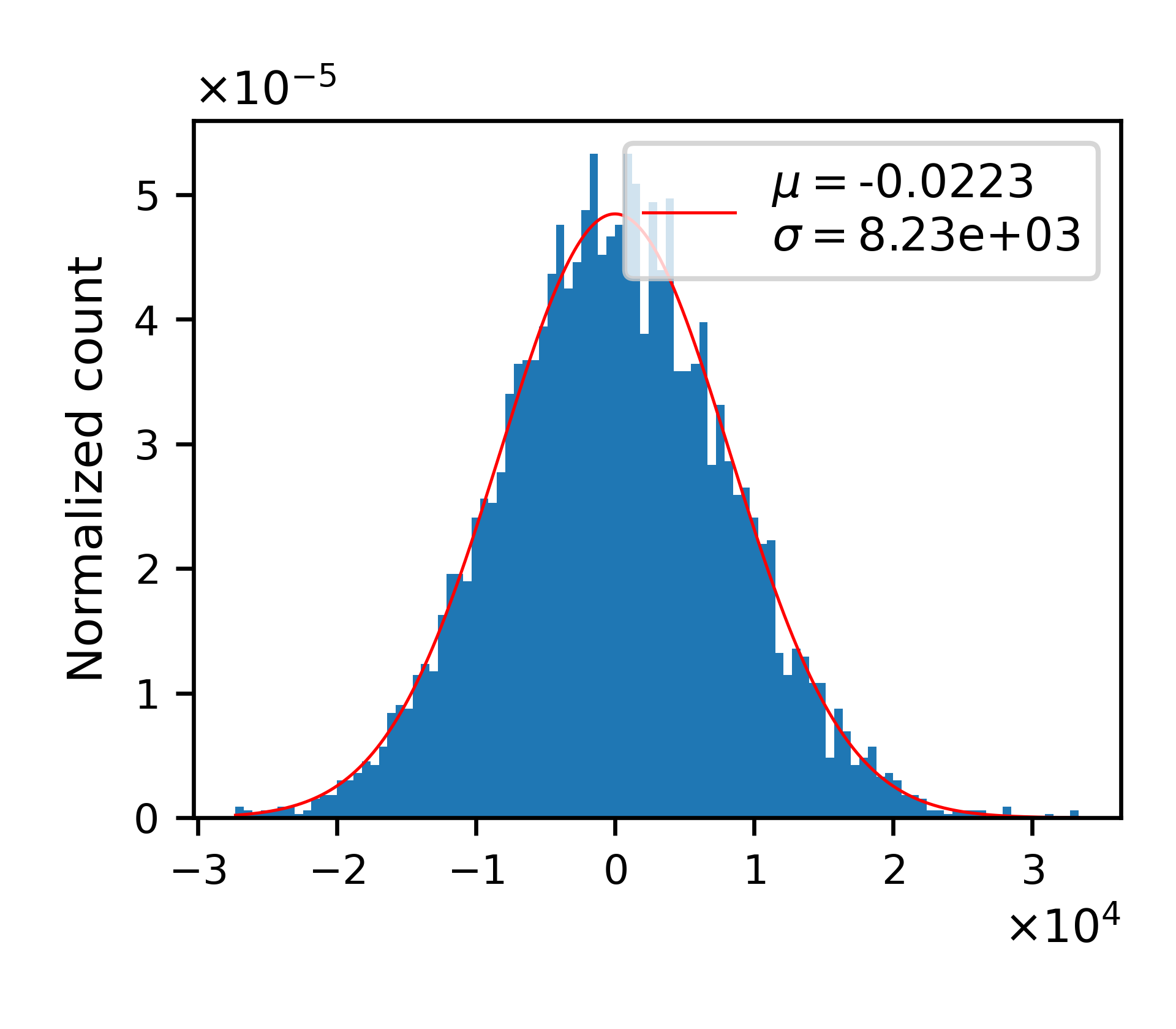 | 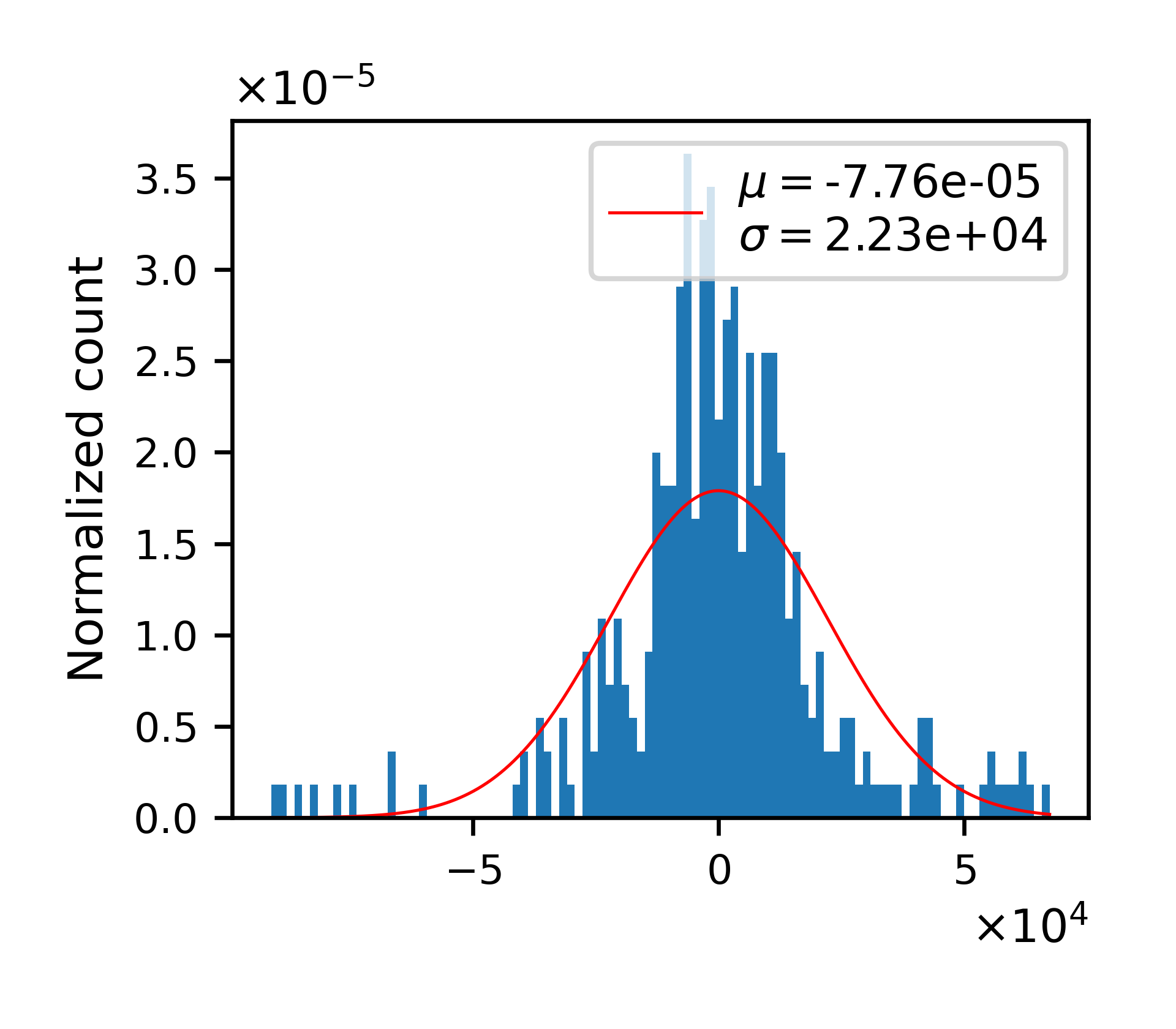 |
| 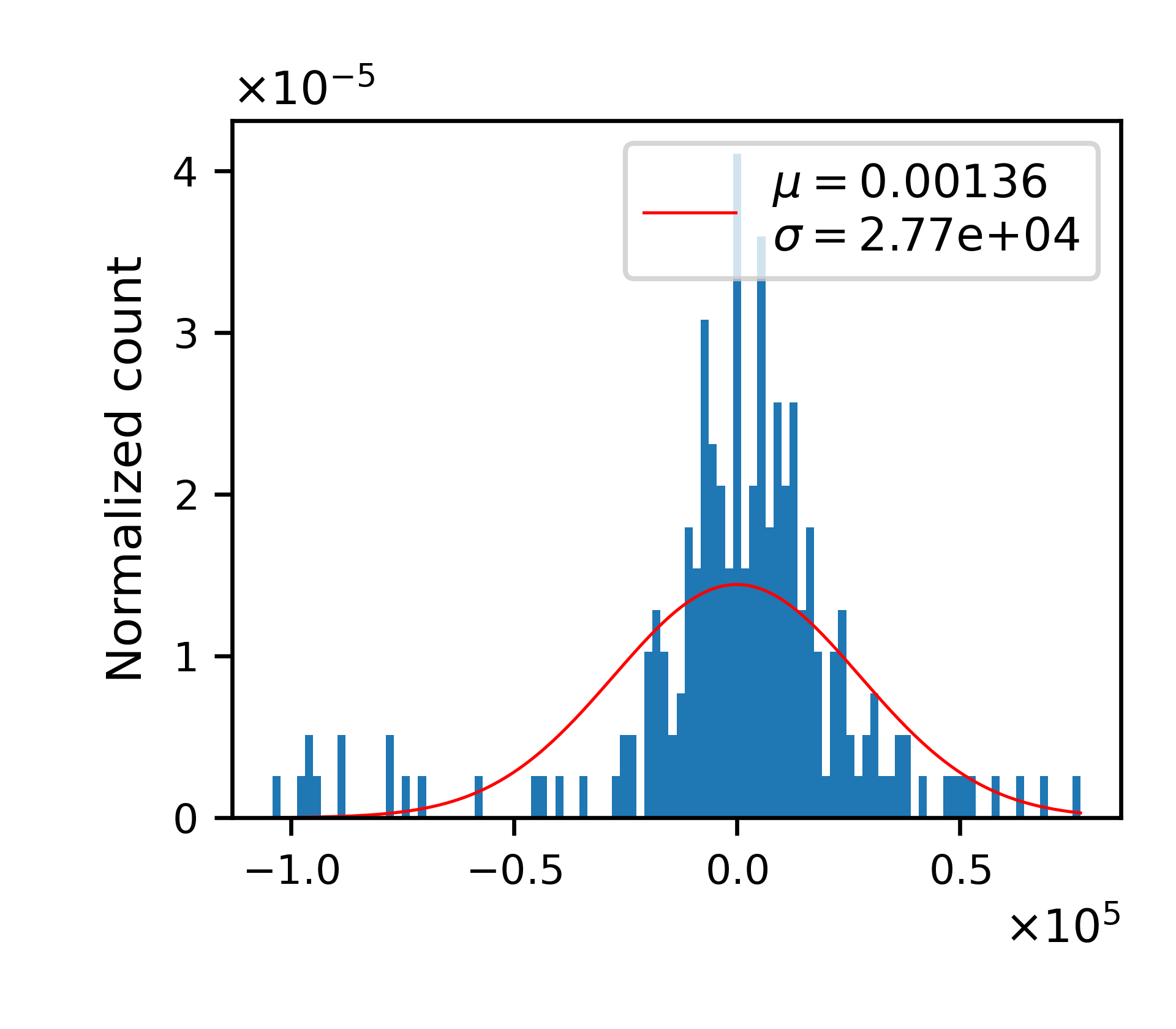 | 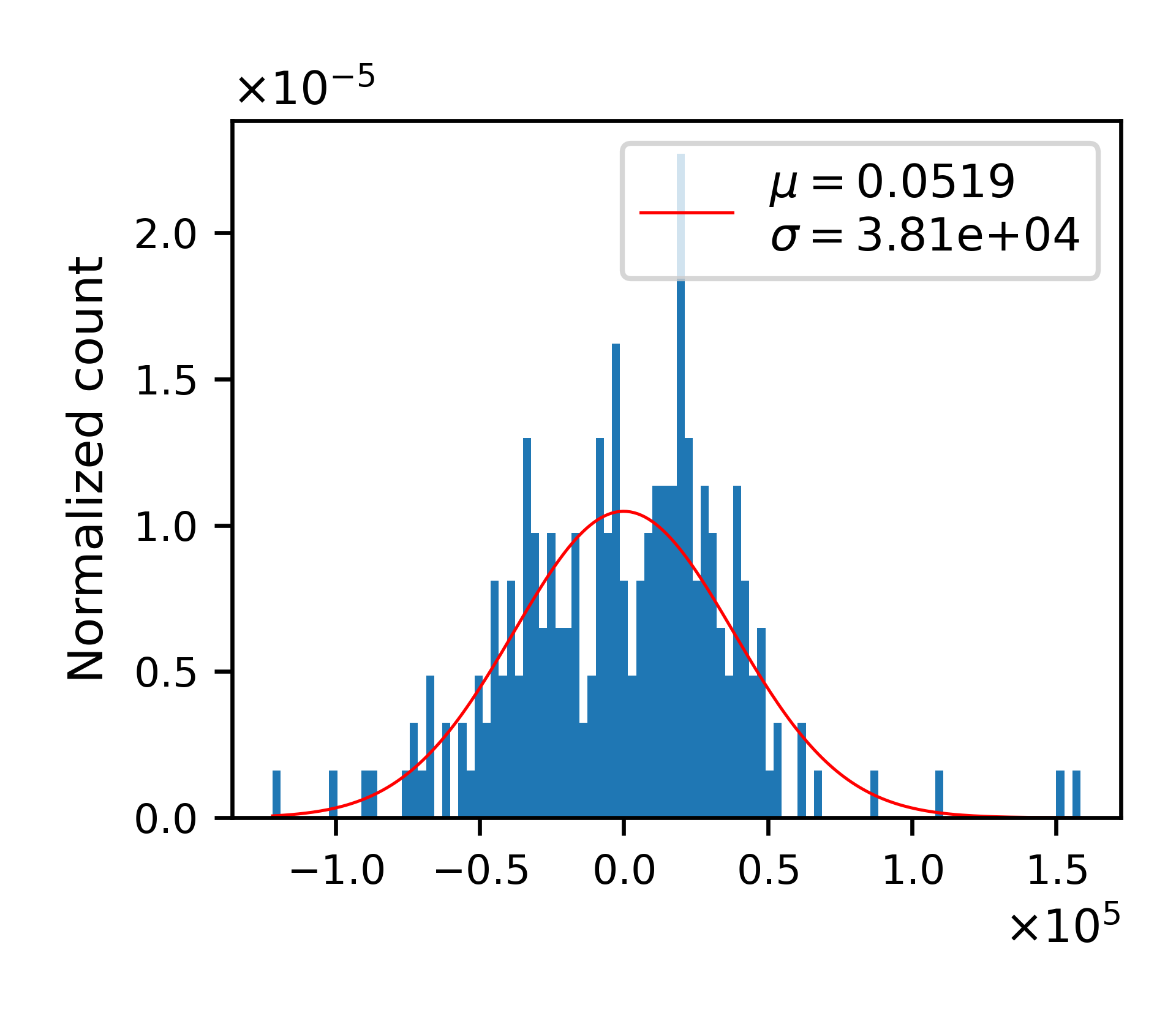 |
| 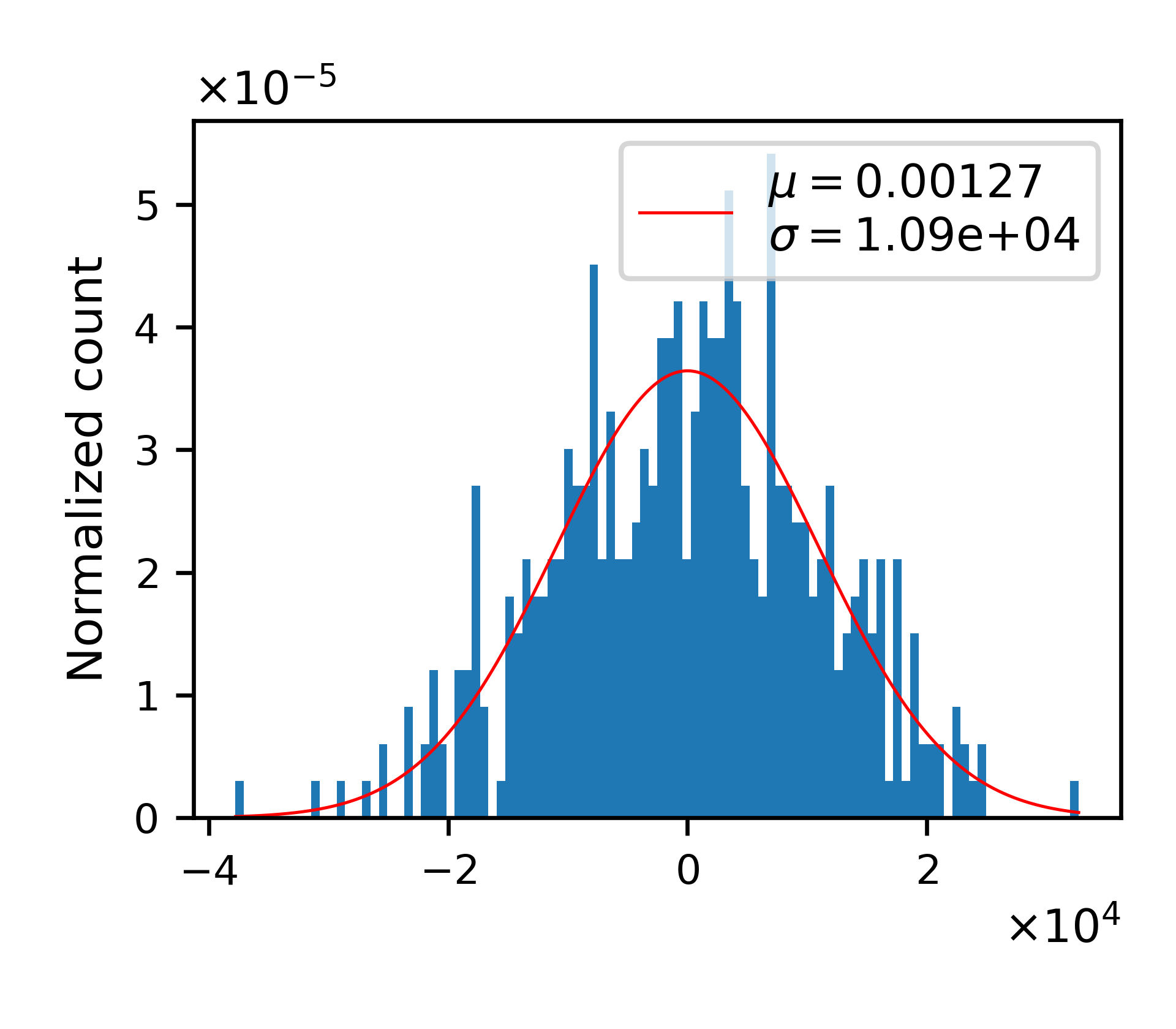 | 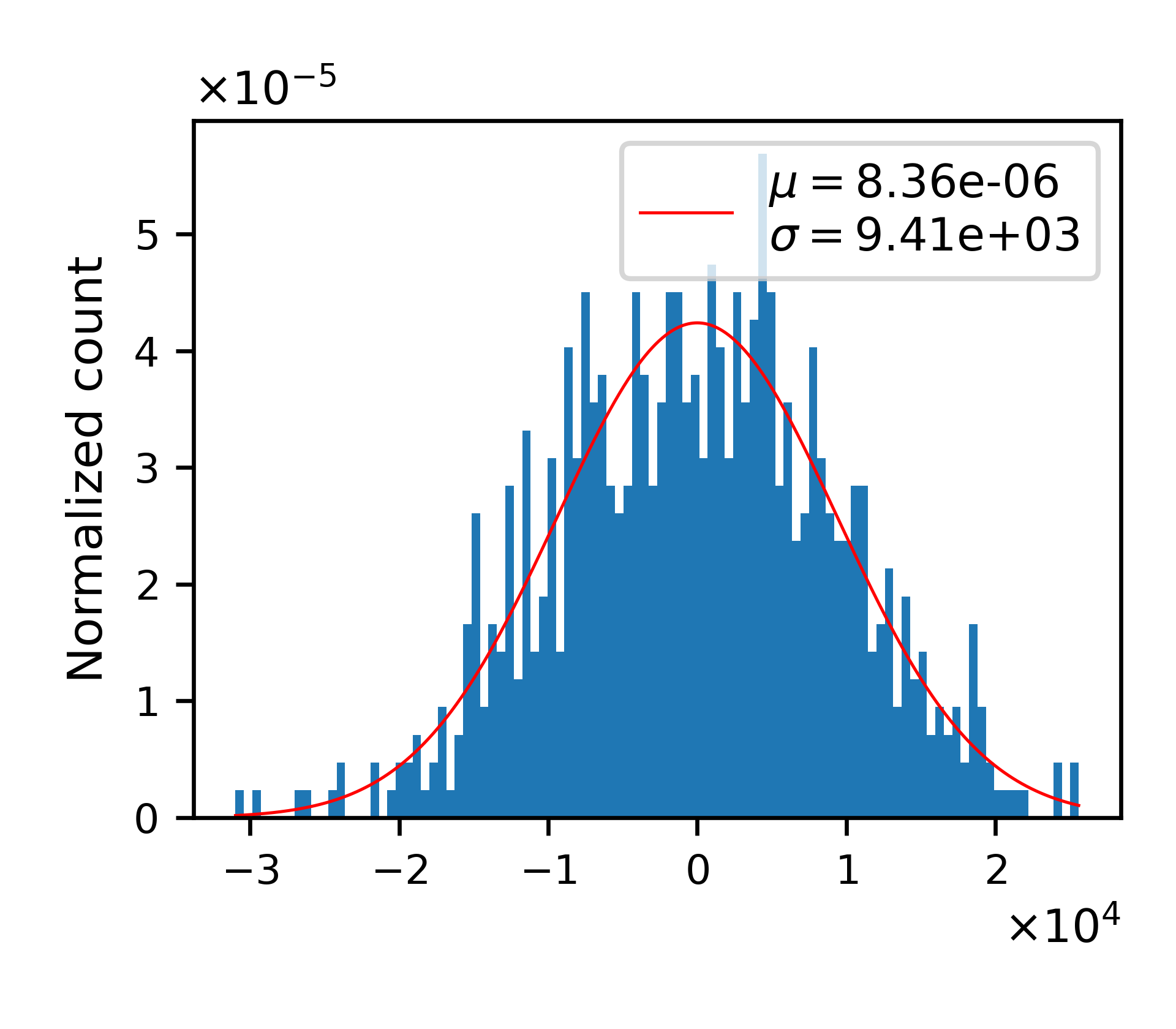 |
| 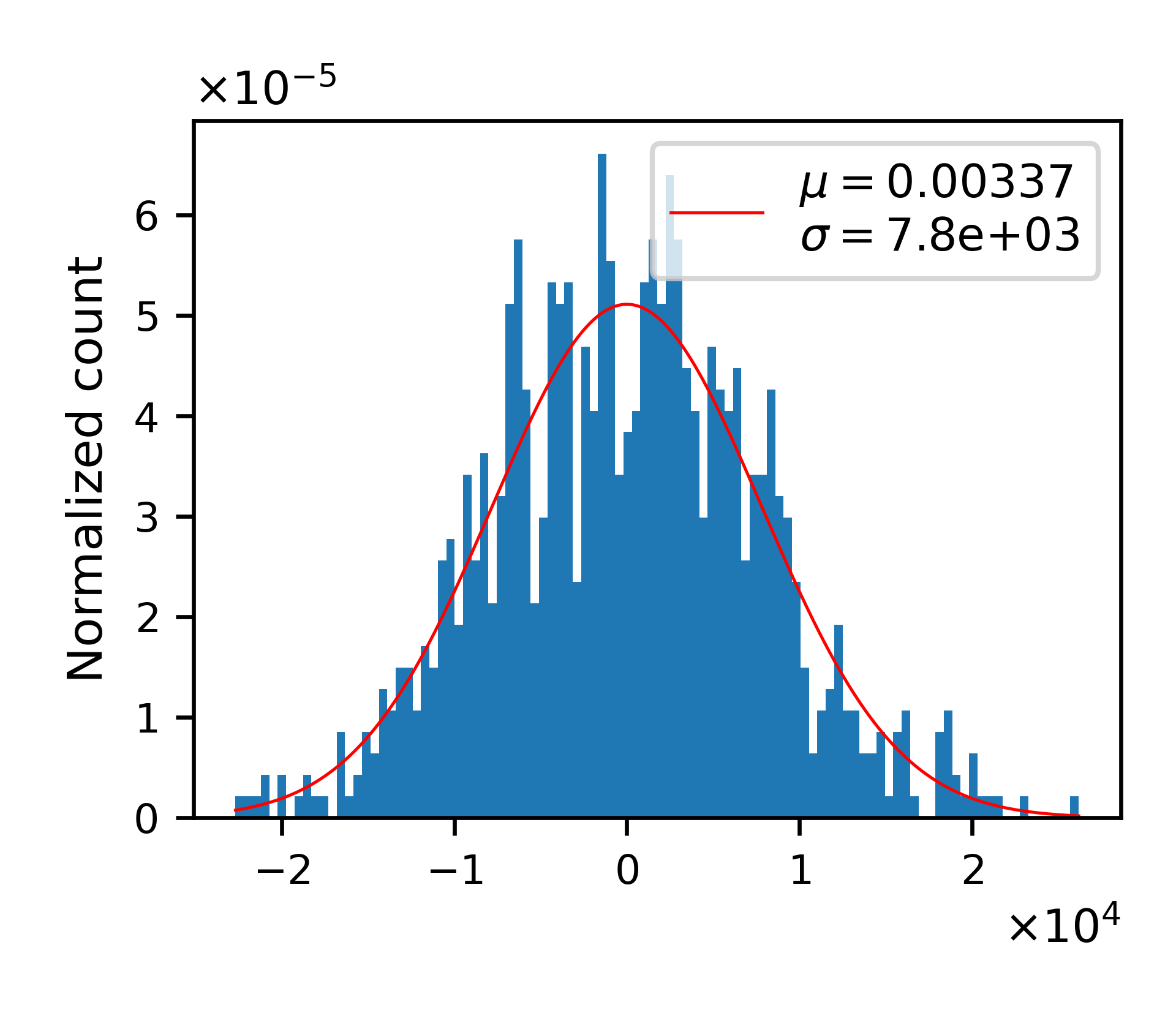 | 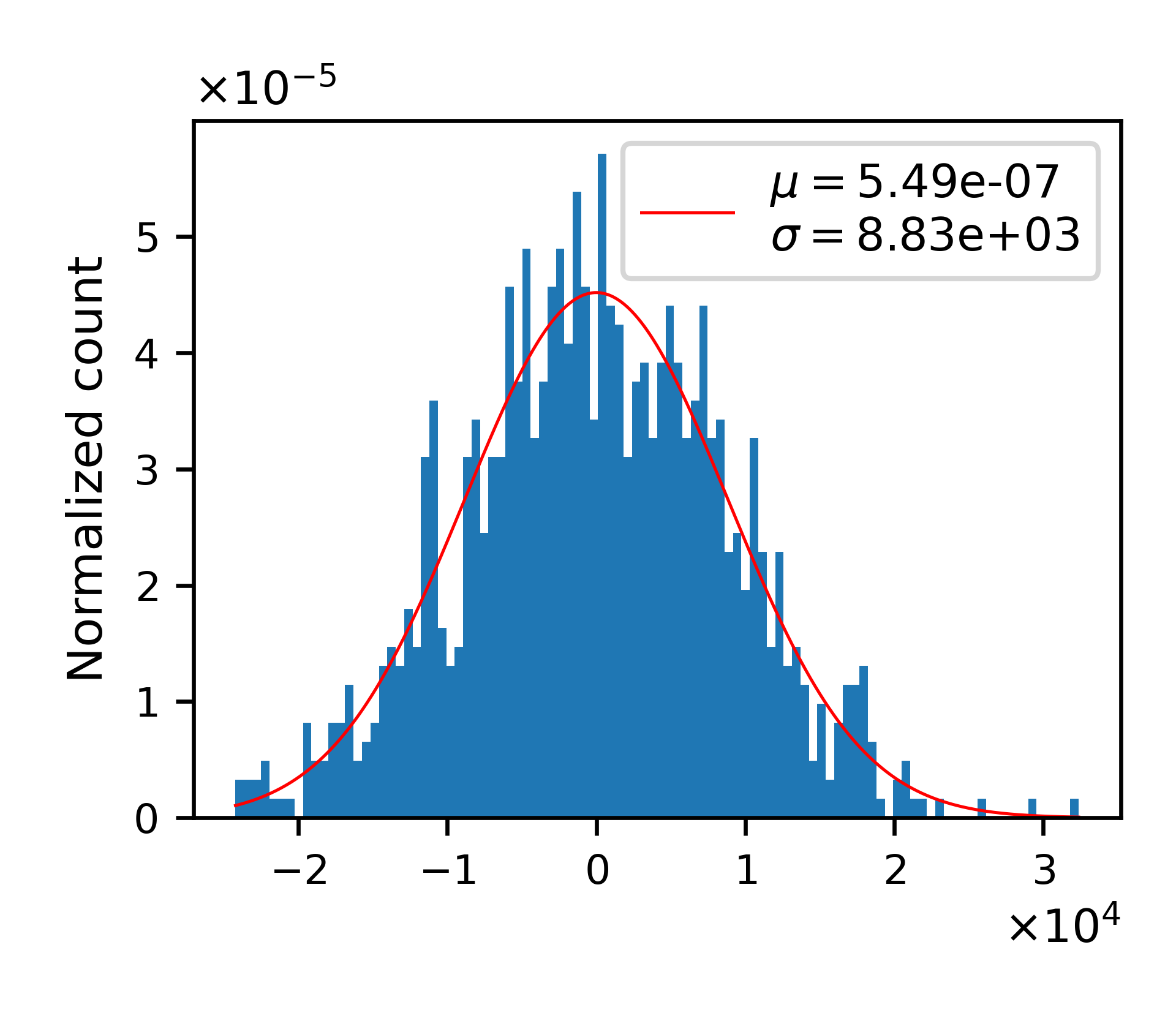 |
| 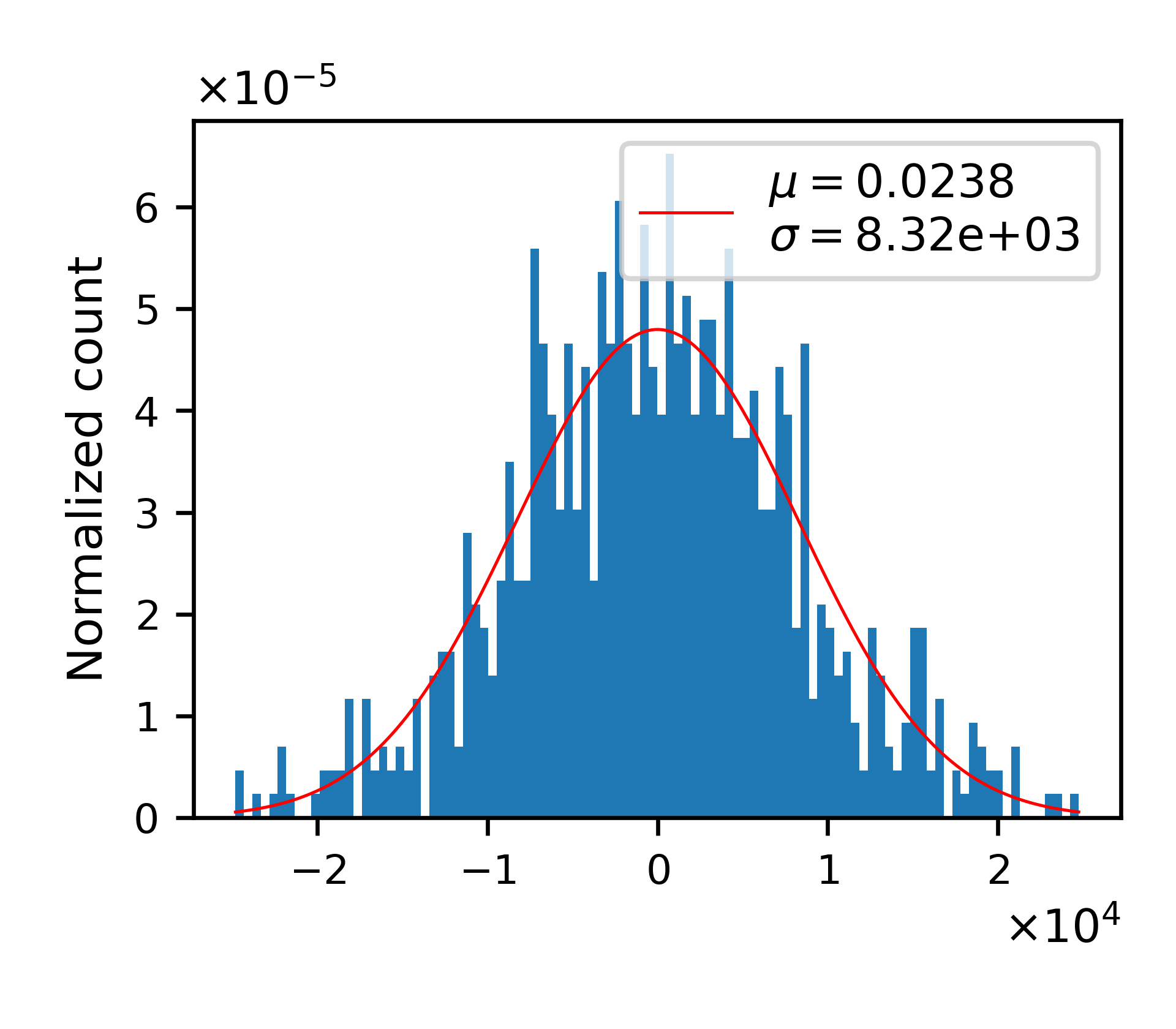 | 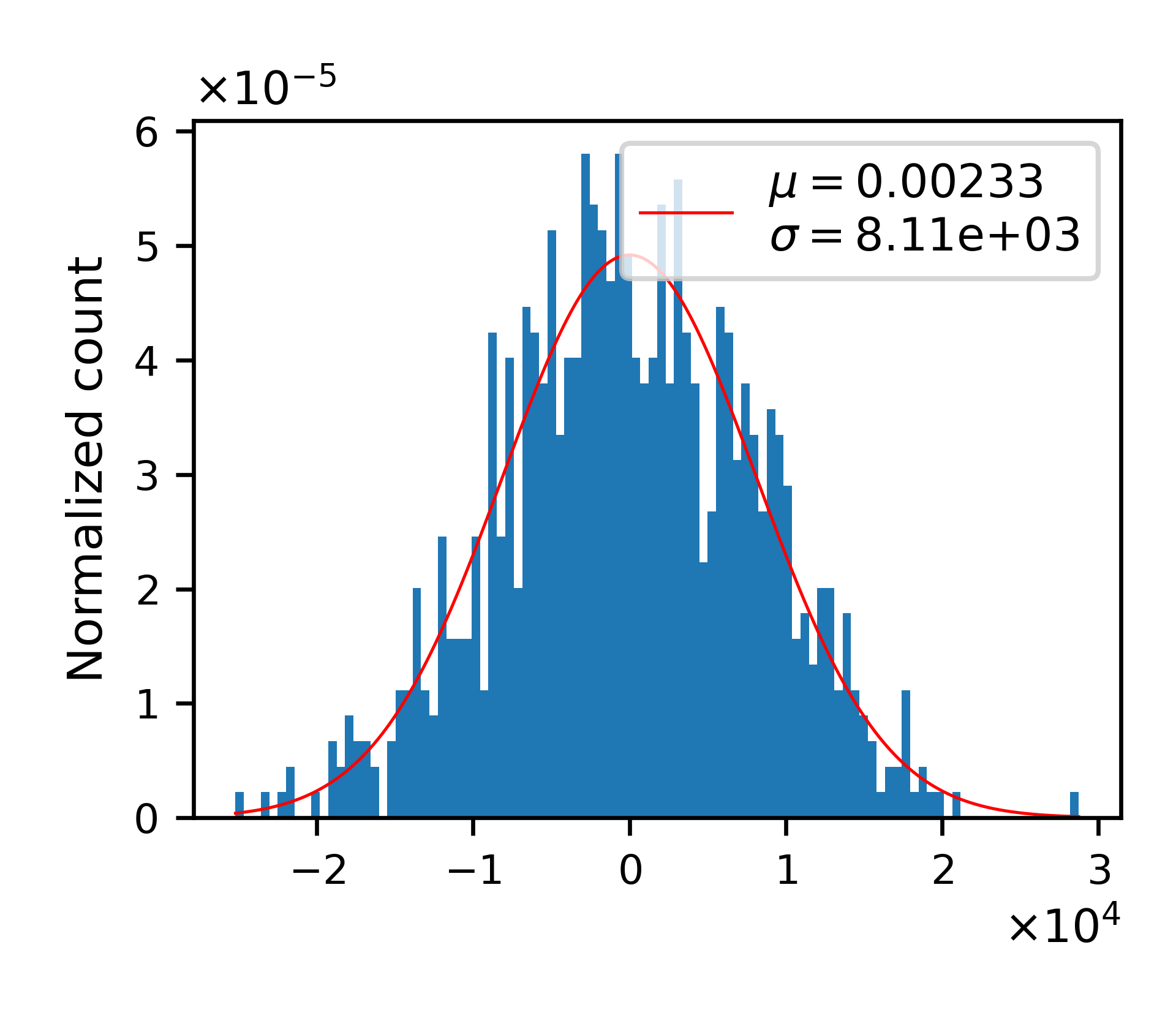 |
| 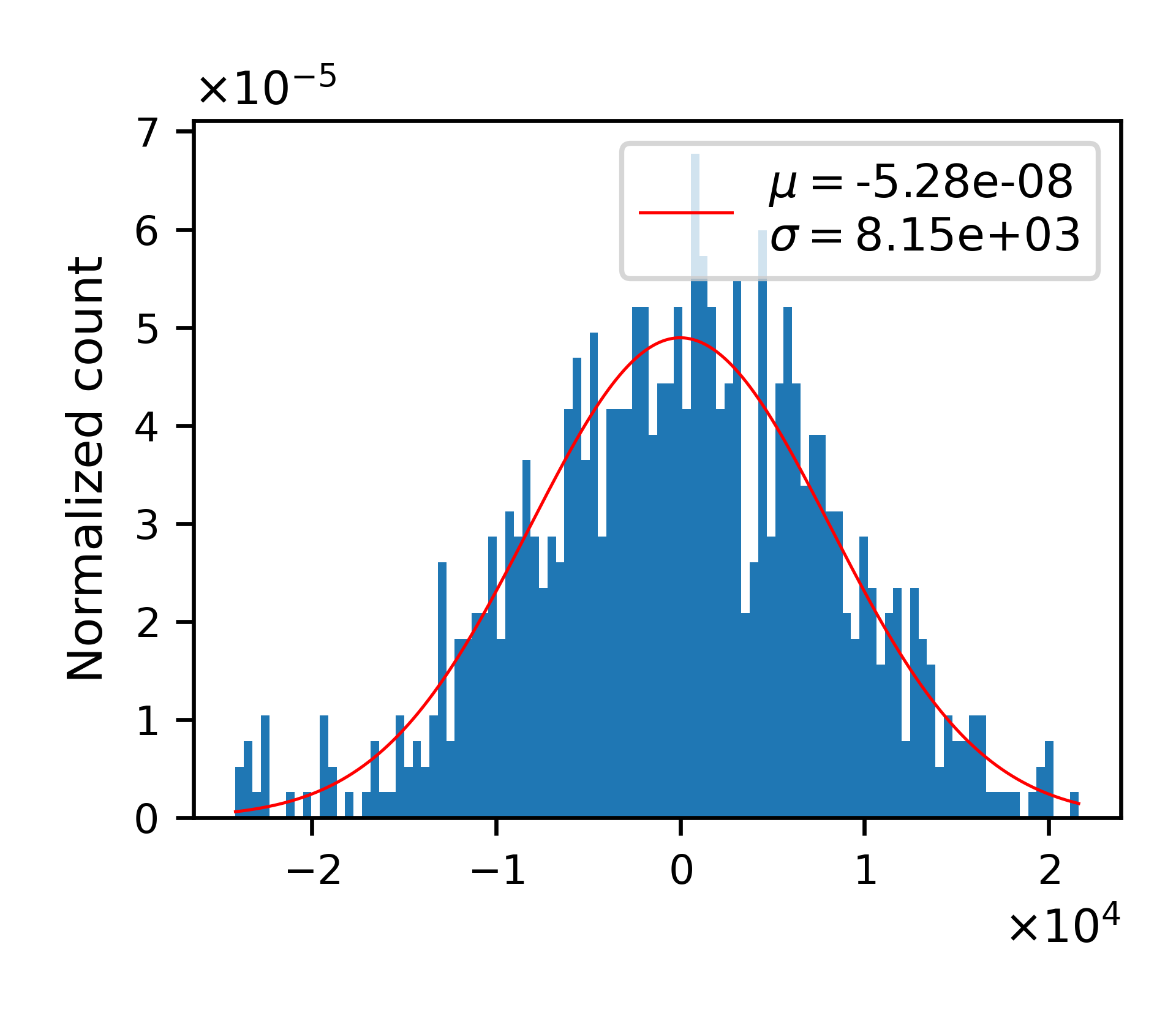 | 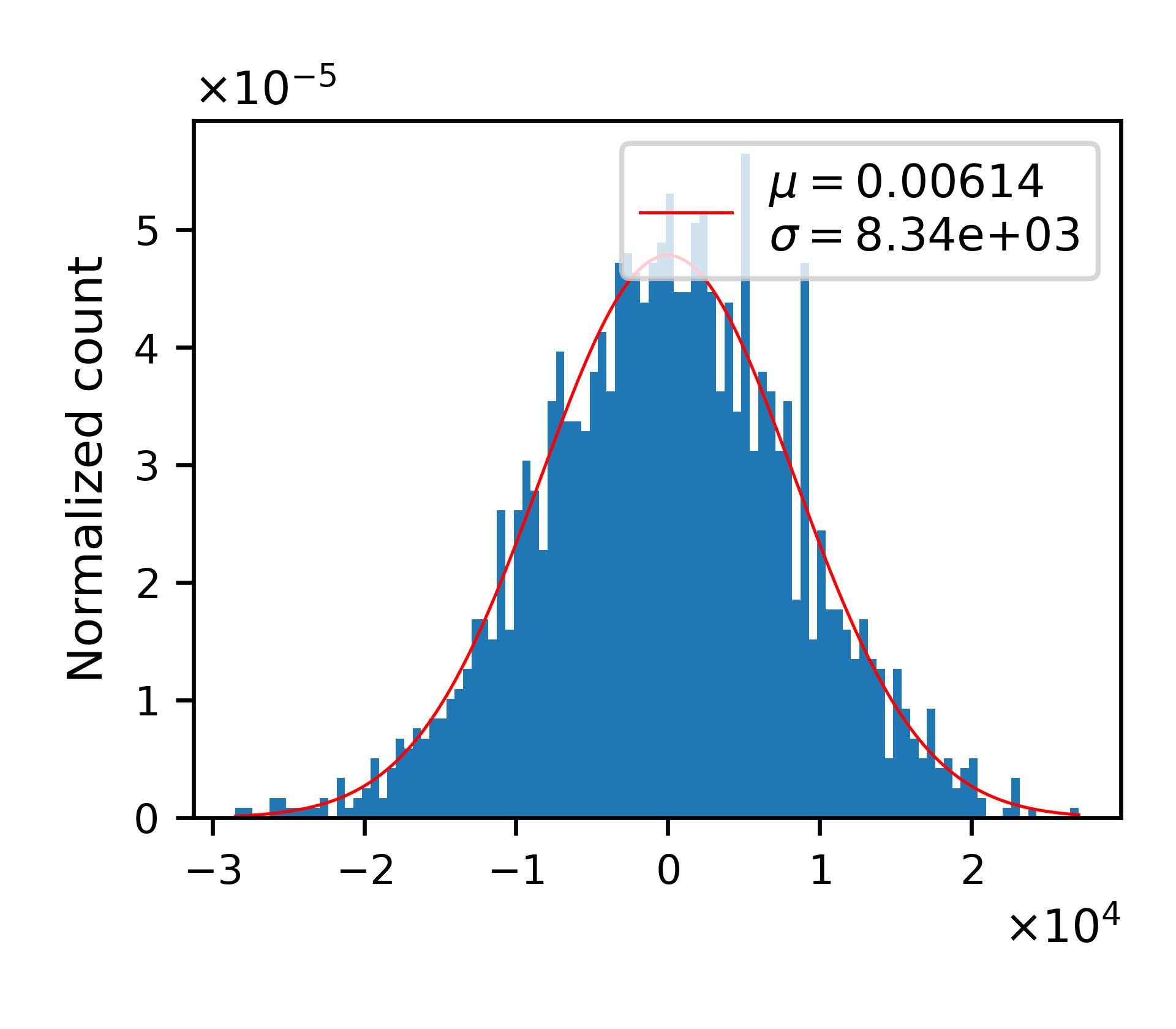 |

## S2.6. Ni-SAL-HDPT output file

SPECTRA PATH:

/path/to/spectra

Points:

1 299.000

2 298.900

3 298.800

4 298.700

5 298.600

6 298.500

7 298.400

8 298.300

9 298.200

10 298.100

11 298.000

12 297.900

INPUT1: nisal_genn_inp1_new

n. peak name ppm1 ppm2 v mult

1 true -257.13300 -424.05500 -332.46938 0

2 true -6.61000 -17.18100 -13.96285 0

3 true -6.61000 -17.18100 -13.41470 0

4 true -6.61000 -17.18100 -10.58259 0

5 true -6.61000 -17.18100 -8.57270 0

6 true 18.13500 11.59100 14.90646 0

7 true 18.13500 11.59100 14.96737 0

8 true 29.11600 22.42300 25.22998 0

9 true 29.11600 22.42300 26.17402 0

10 true 42.19600 27.82900 34.60947 0

11 true 89.92300 67.17900 79.61880 0

12 true 127.04300 97.79100 110.71118 0

13 true 127.04300 97.79100 116.22315 0

14 true 201.62500 168.66700 186.20383 0

15 true 241.35800 215.02300 228.83784 0

16 true 270.42900 245.16100 258.07260 0

17 true 298.81800 273.28300 286.48512 0

18 true 469.72100 404.71100 437.53133 0

19 true 469.72100 404.71100 445.93632 0

INPUT2: nisal_genn_inp2_new

n. peak i ppm1 ppm2 k fwhm phi xg A B C D E

1 0 -257.1 -424.1 2.99800e-05 1.12324e+01 0.00000e+00 2.00000e-01 0.000e+00 0.000e+00 0.000e+00 0.000e+00 0.000e+00

2 0 -6.6 -17.2 4.27765e-05 3.64732e-01 0.00000e+00 2.00000e-01 0.000e+00 0.000e+00 0.000e+00 0.000e+00 0.000e+00

3 1 -6.6 -17.2 2.25596e-05 1.08744e+00 0.00000e+00 2.00000e-01 0.000e+00 0.000e+00 0.000e+00 0.000e+00 0.000e+00

4 2 -6.6 -17.2 2.99800e-05 2.56663e-01 0.00000e+00 2.00000e-01 0.000e+00 0.000e+00 0.000e+00 0.000e+00 0.000e+00

5 3 -6.6 -17.2 1.95696e-05 2.49909e-01 0.00000e+00 2.00000e-01 0.000e+00 0.000e+00 0.000e+00 0.000e+00 0.000e+00

6 0 18.1 11.6 2.79226e-05 2.08875e-01 0.00000e+00 2.00000e-01 0.000e+00 0.000e+00 0.000e+00 0.000e+00 0.000e+00

7 1 18.1 11.6 2.79226e-05 2.04698e-01 0.00000e+00 2.00000e-01 0.000e+00 0.000e+00 0.000e+00 0.000e+00 0.000e+00

8 0 29.1 22.4 3.21889e-05 1.02603e-01 0.00000e+00 2.00000e-01 0.000e+00 0.000e+00 0.000e+00 0.000e+00 0.000e+00

9 1 29.1 22.4 3.21889e-05 6.84018e-02 0.00000e+00 2.00000e-01 0.000e+00 0.000e+00 0.000e+00 0.000e+00 0.000e+00

10 0 42.2 27.8 1.95696e-05 9.47026e-01 0.00000e+00 2.00000e-01 -8.000e-04 0.000e+00 0.000e+00 0.000e+00 0.000e+00

11 0 89.9 67.2 2.60065e-05 9.59851e-01 0.00000e+00 2.00000e-01 -9.000e-04 0.000e+00 0.000e+00 0.000e+00 0.000e+00

12 0 127.0 97.8 2.79226e-05 3.35101e+00 0.00000e+00 2.00000e-01 -1.030e-03 0.000e+00 0.000e+00 0.000e+00 0.000e+00

13 1 127.0 97.8 2.42218e-05 2.73322e+00 0.00000e+00 2.00000e-01 -1.030e-03 0.000e+00 0.000e+00 0.000e+00 0.000e+00

14 0 201.6 168.7 3.45606e-05 2.86991e+00 0.00000e+00 2.00000e-01 -1.000e-03 0.000e+00 0.000e+00 0.000e+00 0.000e+00

15 0 241.4 215.0 2.42218e-05 2.68173e+00 0.00000e+00 2.00000e-01 -5.000e-04 0.000e+00 0.000e+00 0.000e+00 0.000e+00

16 0 270.4 245.2 2.79226e-05 1.22991e+00 0.00000e+00 2.00000e-01 -5.000e-04 0.000e+00 0.000e+00 0.000e+00 0.000e+00

17 0 298.8 273.3 2.25596e-05 1.30869e+00 0.00000e+00 2.00000e-01 0.000e+00 0.000e+00 0.000e+00 0.000e+00 0.000e+00

18 0 469.7 404.7 2.99800e-05 6.12372e+00 0.00000e+00 2.00000e-01 -1.000e-04 0.000e+00 0.000e+00 0.000e+00 0.000e+00

19 1 469.7 404.7 3.45606e-05 6.41533e+00 0.00000e+00 2.00000e-01 -1.000e-04 0.000e+00 0.000e+00 0.000e+00 0.000e+00

====================================================================

====================================================================

(I: fit interval, P: N. point)

------------------------------

FIT RANGE: (-257.13:-424.06) ppm

I: 1 P: 1

------------------------------

Fit Report:

[[Fit Statistics]]

# fitting method = leastsq

# function evals = 144

# data points = 5481

# variables = 8

chi-square = 7.1027e+11

reduced chi-square = 1.2978e+08

Akaike info crit = 102400.344

Bayesian info crit = 102453.217

[[Variables]]

shift_1: -332.383456 +/- 0.04700967 (0.01%) (init = -332.4694)

k_1: 3.8022e-05 +/- 5.3745e-07 (1.41%) (init = 2.998e-05)

lw_1: 8.69015281 +/- 0.10630528 (1.22%) (init = 3.5)

ph_1: -0.12863248 +/- 0.00874588 (6.80%) (init = 0)

xg_1: 7.3471e-10 +/- 0.03696318 (5030955439.45%) (init = 0.2)

A: 2.3899e-04 +/- 4.3618e-06 (1.83%) (init = 0)

B: 2.2814e-06 +/- 1.4698e-07 (6.44%) (init = 0)

C: -2.4847e-08 +/- 8.6368e-10 (3.48%) (init = 0)

D: 0 (fixed)

E: 0 (fixed)

[[Correlations]] (unreported correlations are < 0.100)

C(B, C) = -0.9739

C(A, B) = -0.8010

C(shift_1, ph_1) = -0.7878

C(k_1, xg_1) = -0.7327

C(A, C) = +0.6772

C(k_1, C) = +0.6663

C(k_1, B) = -0.6545

C(xg_1, C) = -0.4274

C(xg_1, B) = +0.4207

C(lw_1, xg_1) = +0.3719

C(ph_1, A) = +0.2842

C(k_1, A) = +0.2818

C(k_1, lw_1) = +0.2386

C(shift_1, A) = -0.2128

C(lw_1, C) = +0.2002

C(lw_1, B) = -0.1962

C(xg_1, A) = -0.1592

n.peak Shift Integral

1 -332.383 72635005.949 +/- 32833156.988

------------------------------

FIT RANGE: (-257.13:-424.06) ppm

I: 1 P: 2

------------------------------

Fit Report:

[[Fit Statistics]]

# fitting method = leastsq

# function evals = 19

# data points = 5481

# variables = 5

chi-square = 7.0896e+11

reduced chi-square = 1.2947e+08

Akaike info crit = 102384.245

Bayesian info crit = 102417.290

[[Variables]]

shift_1: -332.526373 +/- 0.02931326 (0.01%) (init = -332.3835)

k_1: 3.7670e-05 +/- 2.1565e-07 (0.57%) (init = 3.8022e-05)

lw_1: 8.690153 (fixed)

ph_1: -0.1286325 (fixed)

xg_1: 7.34715e-10 (fixed)

A: 2.4796e-04 +/- 4.0564e-06 (1.64%) (init = 0.0002389909)

B: 1.9762e-06 +/- 1.2109e-07 (6.13%) (init = 2.281374e-06)

C: -2.3391e-08 +/- 7.0542e-10 (3.02%) (init = -2.484656e-08)

D: 0 (fixed)

E: 0 (fixed)

[[Correlations]] (unreported correlations are < 0.100)

C(B, C) = -0.9739

C(A, B) = -0.8541

C(A, C) = +0.7454

C(k_1, C) = +0.4282

C(k_1, B) = -0.4194

C(k_1, A) = +0.1997

n.peak Shift Integral

1 -332.526 71660585.833 +/- 33452505.754

------------------------------

## S2.7. Chemical shift temperature dependence Ni-SAL-HDPT

| Listing S2.7.1: Code listing for the generation of figure S2.5.1 and figures S2.5.2. |
| --- |
| from scipy.stats import chi2  from sklearn.metrics import r2_score  import matplotlib.pyplot as plt  from matplotlib.ticker import ScalarFormatter  import numpy as np  data_path = "/home/Nisal_modelfit/Param.txt"  temp_es_path = "/home/Nisal_modelfit/x_1.txt"  data = np.genfromtxt(data_path)  temp_es = np.genfromtxt(temp_es_path)  errors_path = "/home/Nisal_modelfit/Param_err.txt"  errors = np.genfromtxt(errors_path)  peaks = {f"pk{i+1}": data[:, i] for i in range(19)}  temp_set_MeOH_path ='/home/methanol_modelfit/x_1.txt'  temp_set_MeOH = np.genfromtxt(temp_set_MeOH_path)  # correct temperature MeOH  temp_corr_MeOH_path = '/home/methanol_modelfit/temp_corr.txt'  temp_corr_MeOH = np.genfromtxt(temp_corr_MeOH_path)  x1 = temp_set_MeOH  y1 = temp_corr_MeOH  coefficients = np.polyfit(x1, y1, 1)  a, b = coefficients  temp_corr_nisal = a*temp_es + b  inv = 1/temp_corr_nisal  temp_err = 0.025  new_xerr = temp_err / (temp_corr_nisal**2)  ppp = [peaks[f"pk{i}"] for i in range(1, 20)] # Extract pk1, pk2, ..., pk19  for i, y in enumerate(ppp):  fig = plt.figure() # Create a new figure for each array  ax = fig.add_subplot(1, 1, 1)  fig.set_size_inches(4.5, 4)    # Plot data with error bars  plt.errorbar(inv, y, xerr=new_xerr, yerr=errors[:, i], fmt='.', color='darkgreen',  ecolor='m', elinewidth=0.9, capsize=2, capthick=0.2)  plt.title(f"Peak {i+1}")    # Fit data to y = ax + b  aa, bb = np.polyfit(inv, y, 1) # 1 for linear fit    # Compute the fitted values  yfit = aa * inv + bb  # Calculate R^2 score  r2 = r2_score(y, yfit)  plt.errorbar(inv, yfit, color='orange', label=f"y = {aa:.2e}x + {bb:.2e}\n$R^2$ = {r2:.4f}")    ax.xaxis.set_major_formatter(ScalarFormatter(useMathText=True))  ax.ticklabel_format(style='sci', axis='x', scilimits=(0, 0))  plt.xlabel("$1/T_{corr}$ (1/K)", fontsize=12)  plt.ylabel("$\delta$ (ppm)", fontsize=12)  plt.legend()  plt.tight_layout()    fig.savefig(f"plot_{i+1}_nisal.png", dpi=600, bbox_inches='tight')  plt.show() |

| Figure S2.7.1: Scatter plot of ^1^H chemical shifts versus the inverse of the calibrated temperature for the NiSAL-HDPT series of spectra. | |
| --- | --- |
| 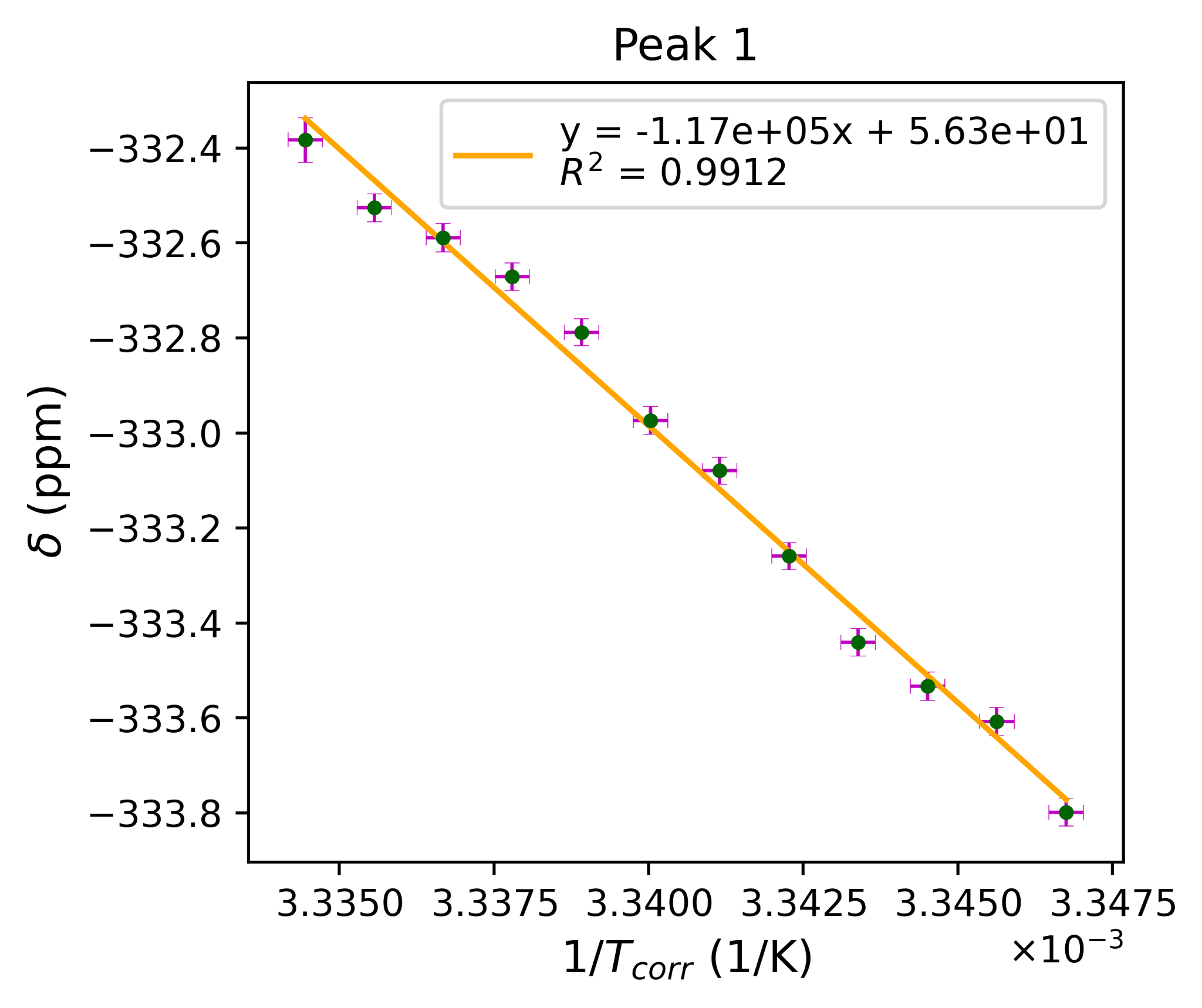 | 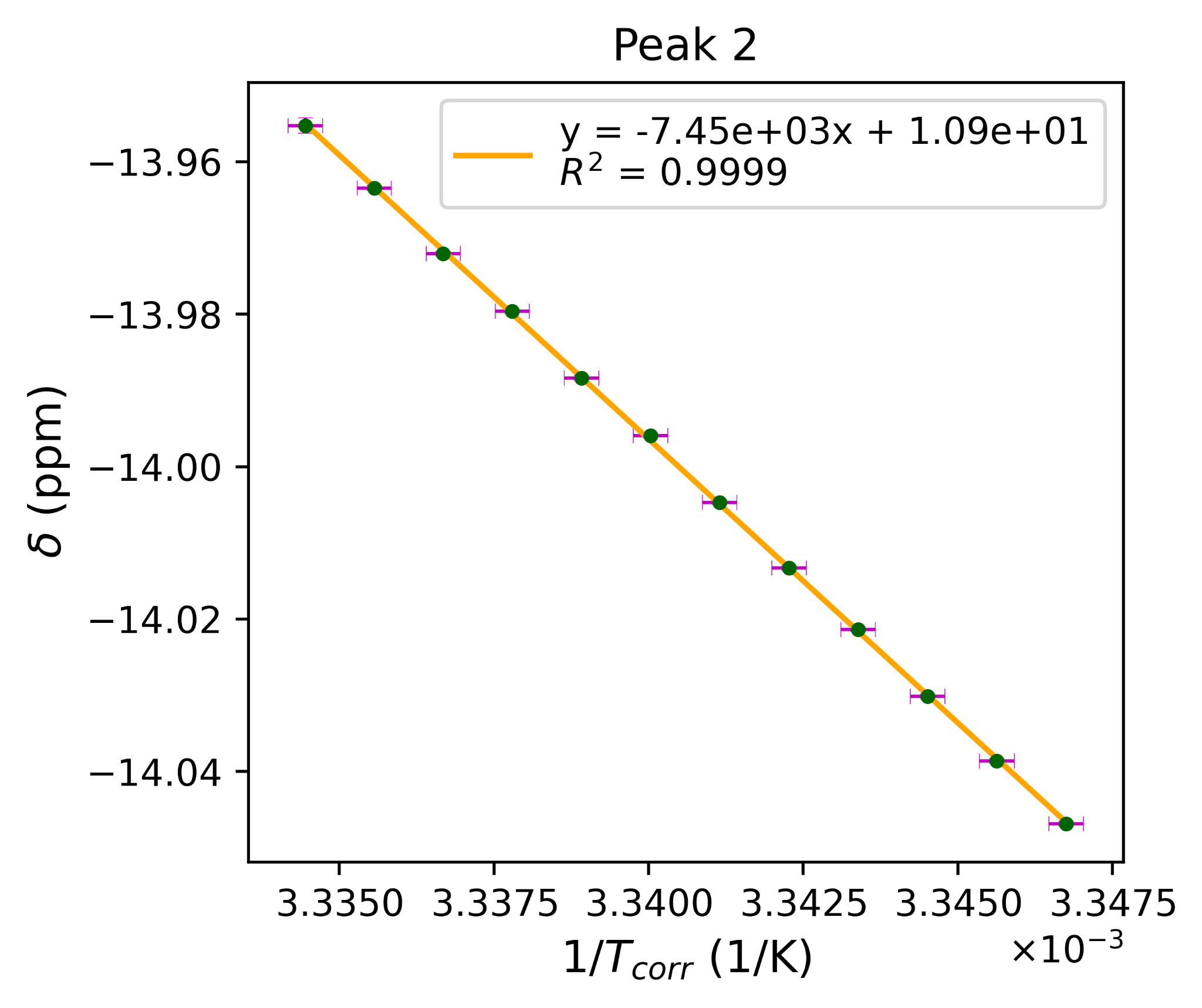 |
| 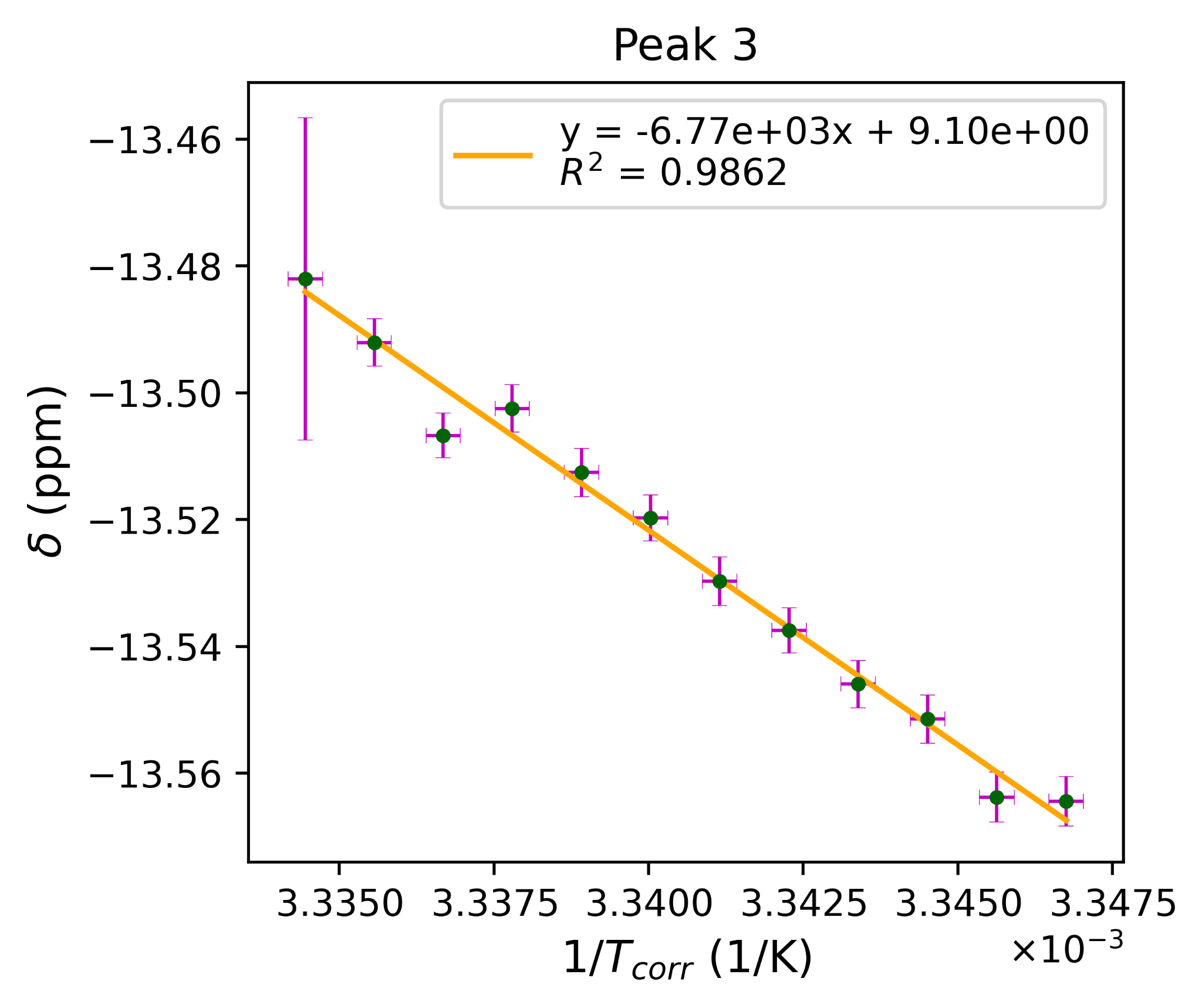 | 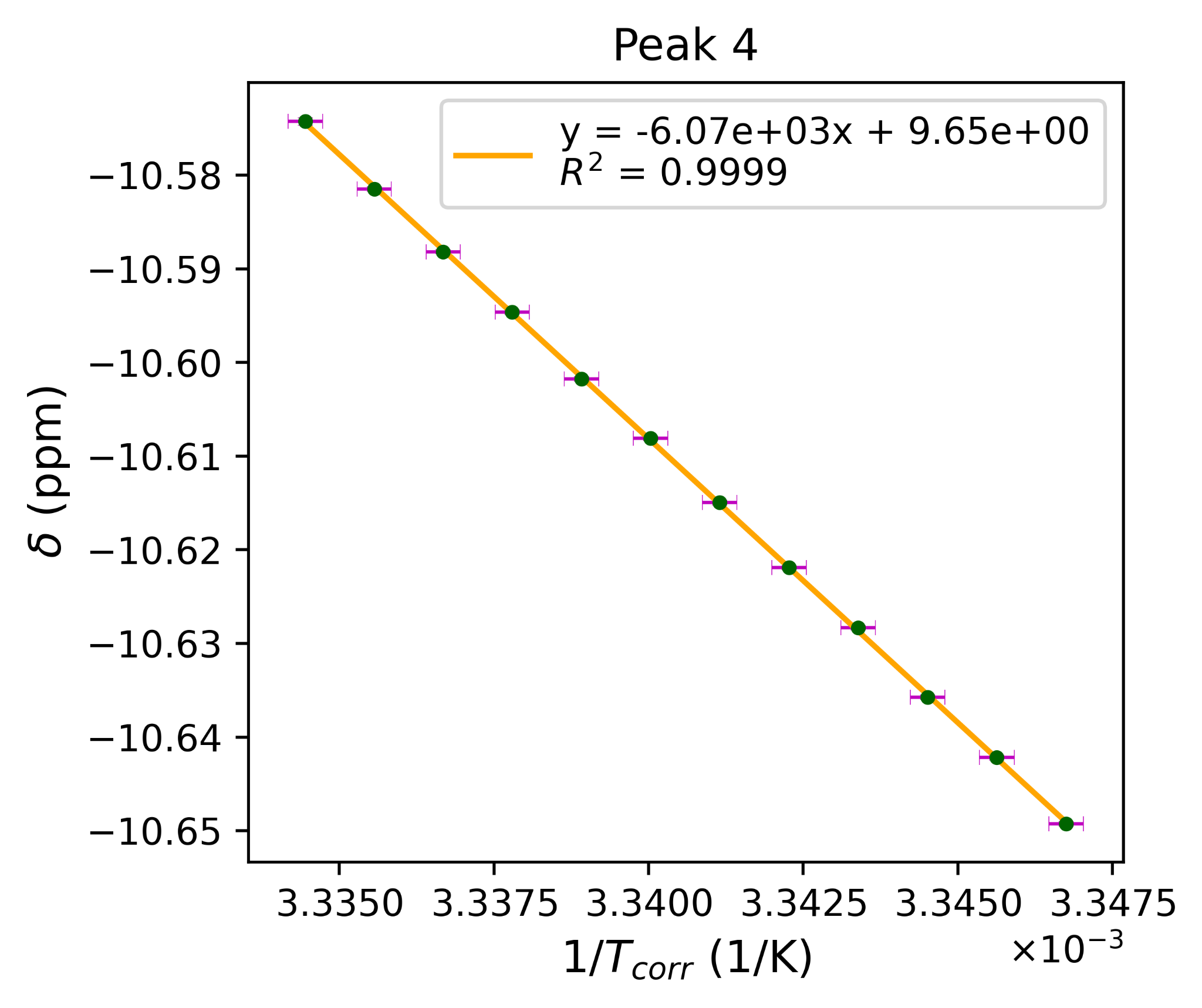 |
| 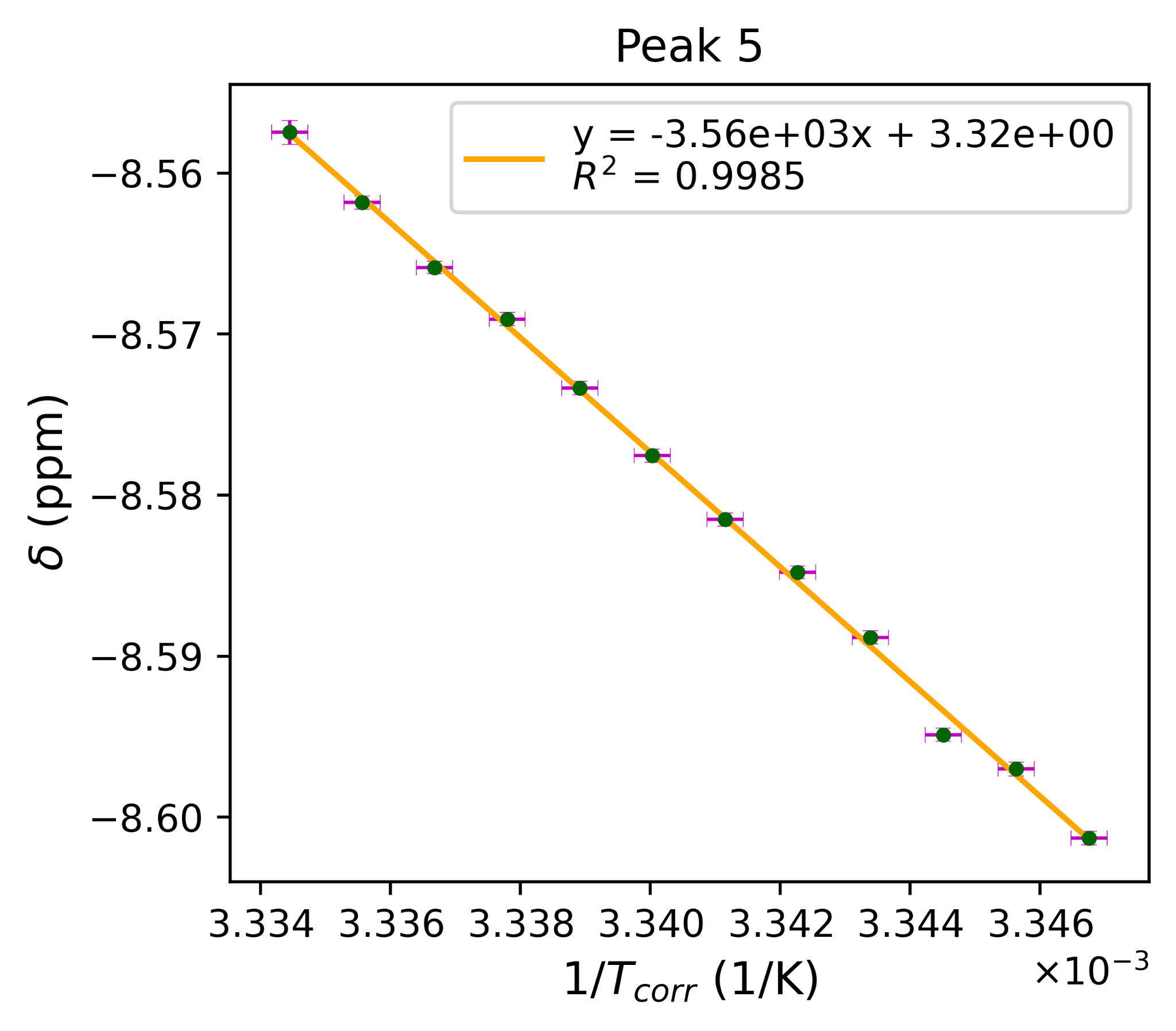 | 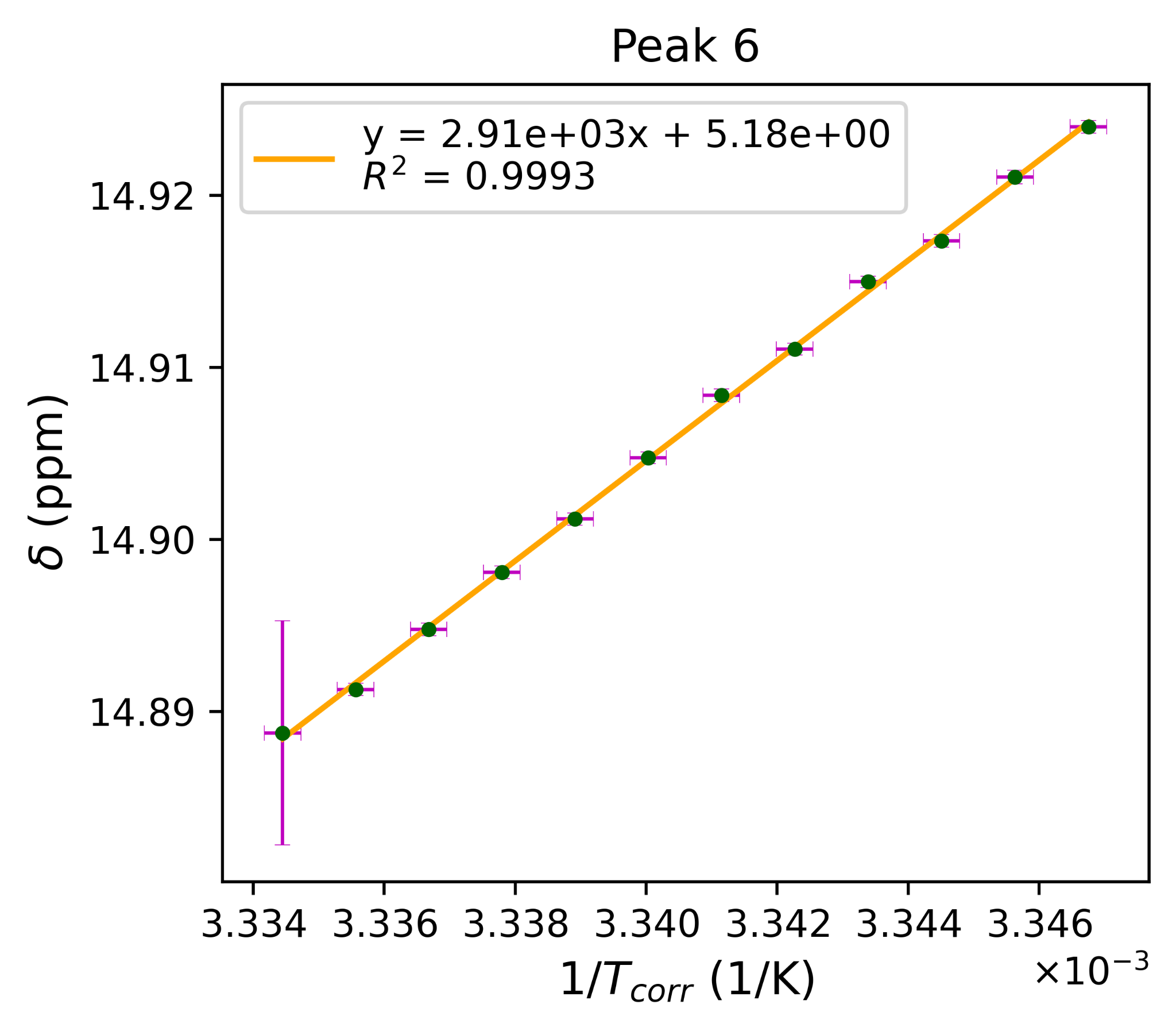 |
| 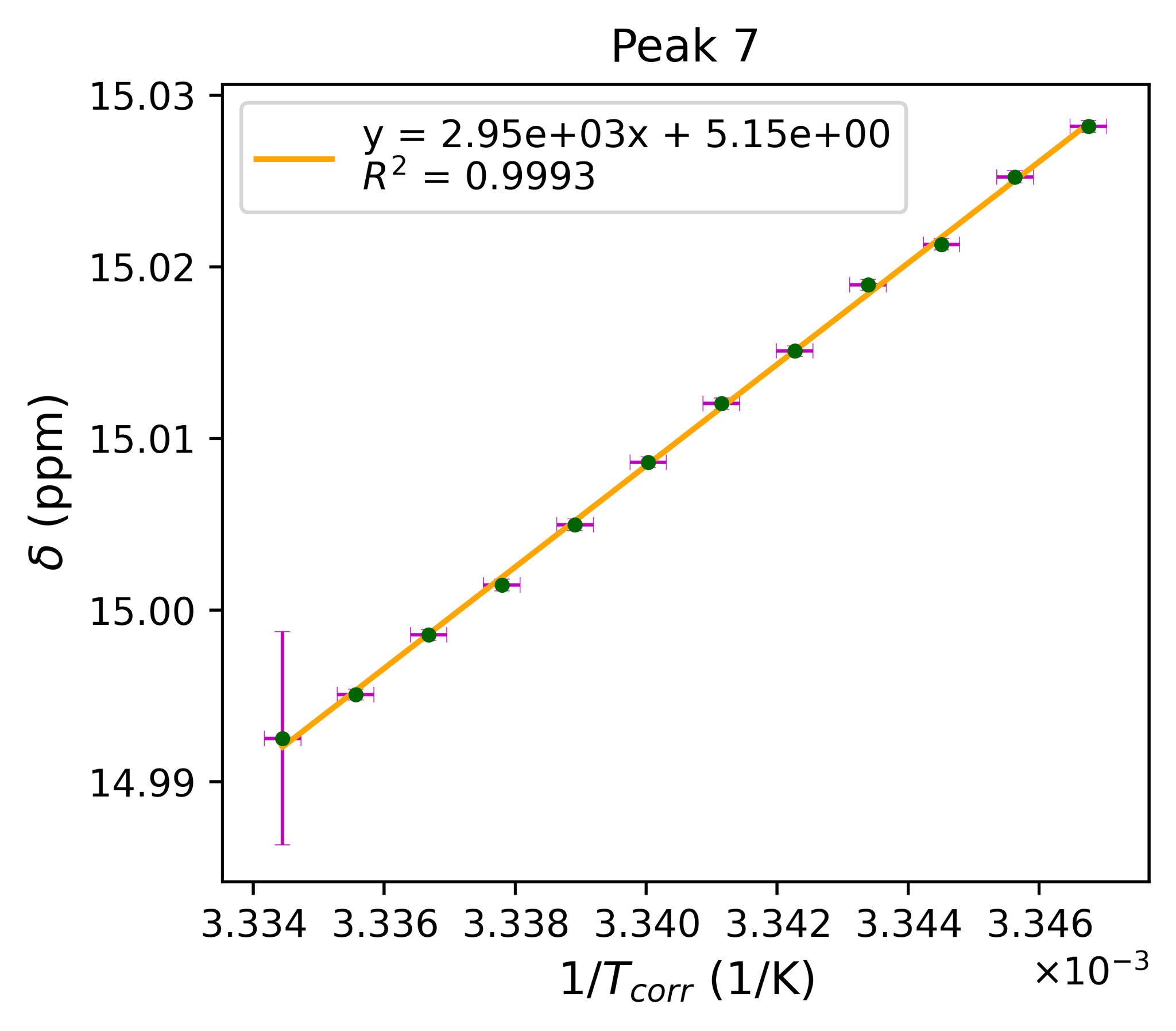 | 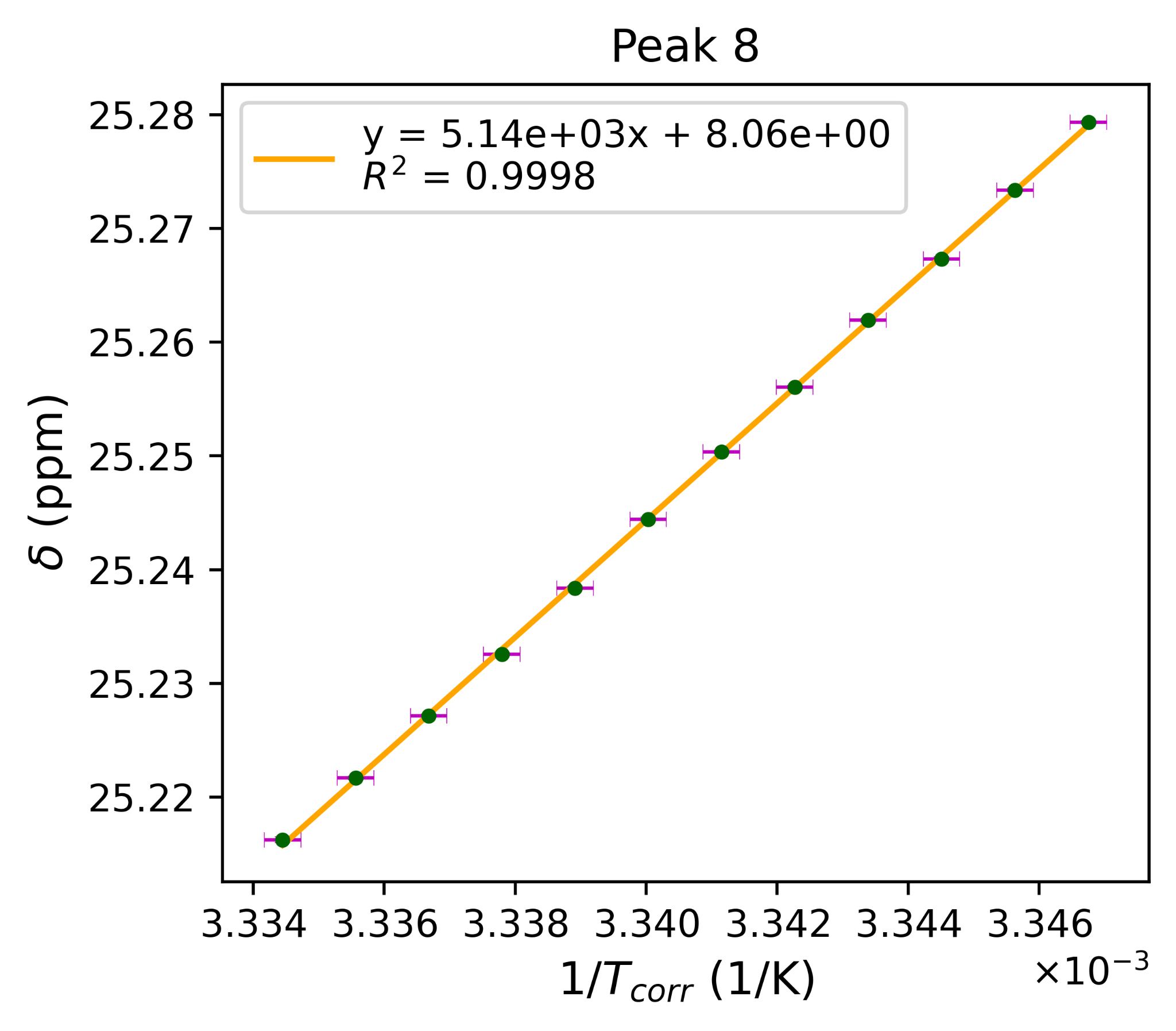 |
| 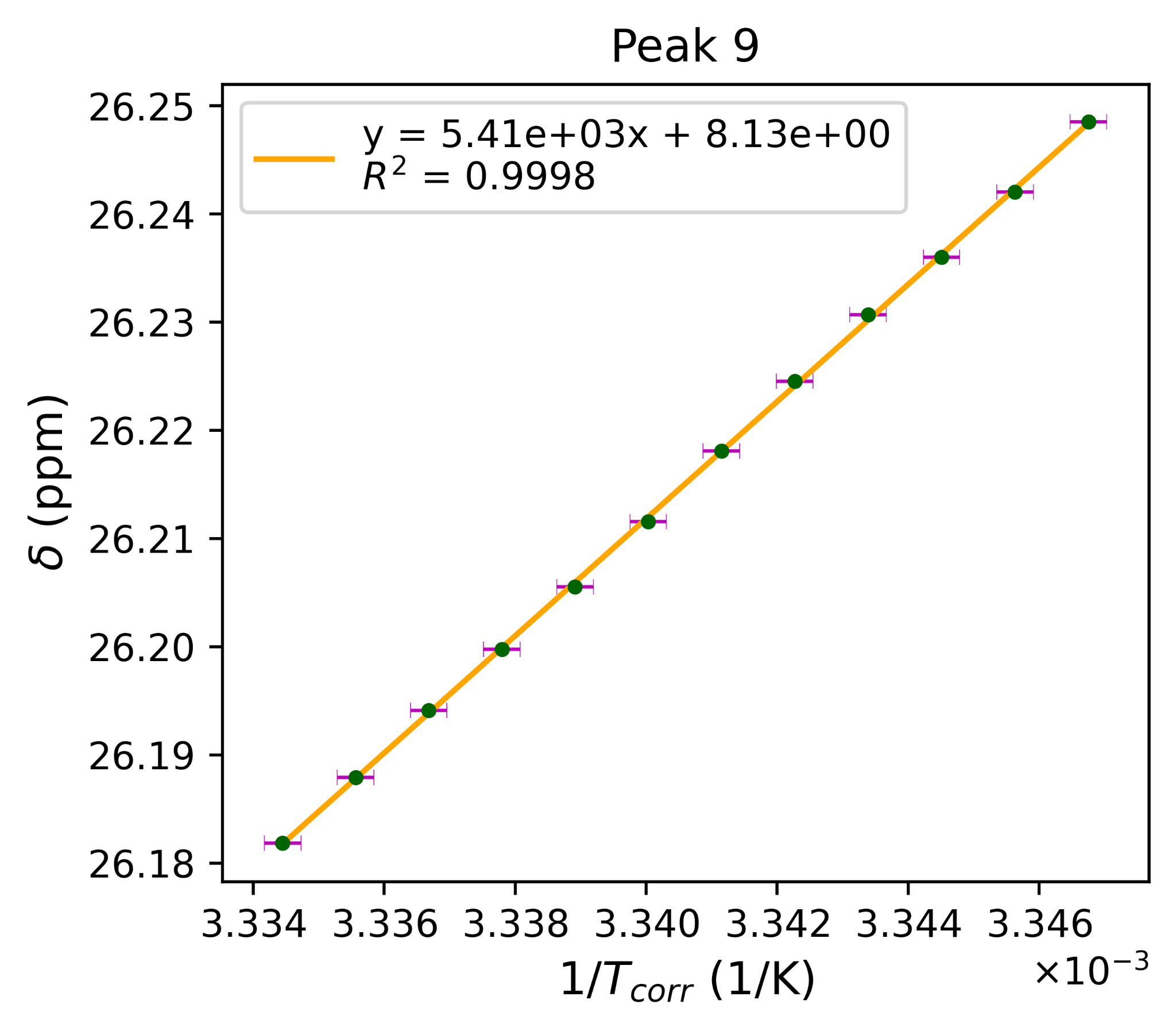 | 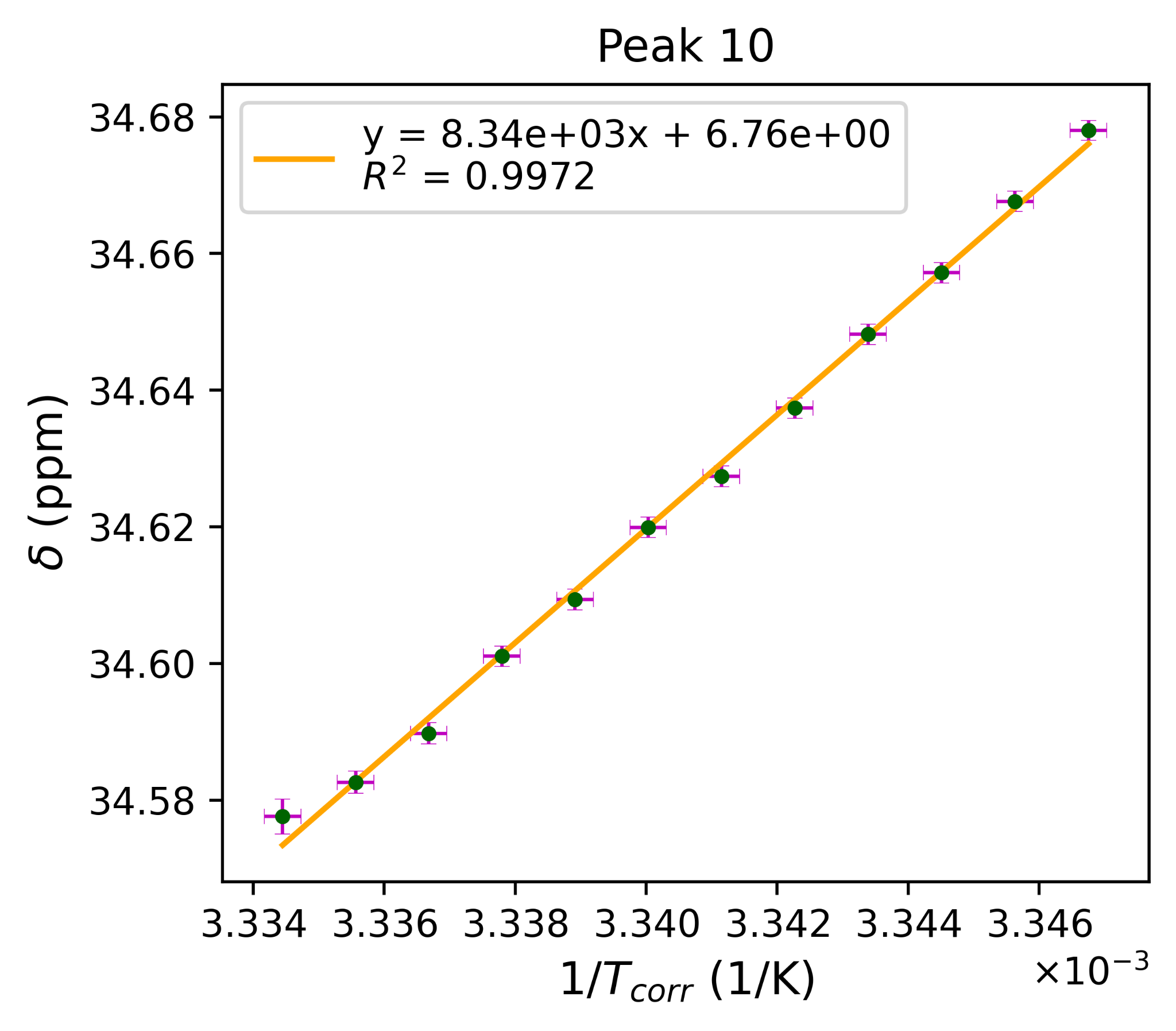 |
| 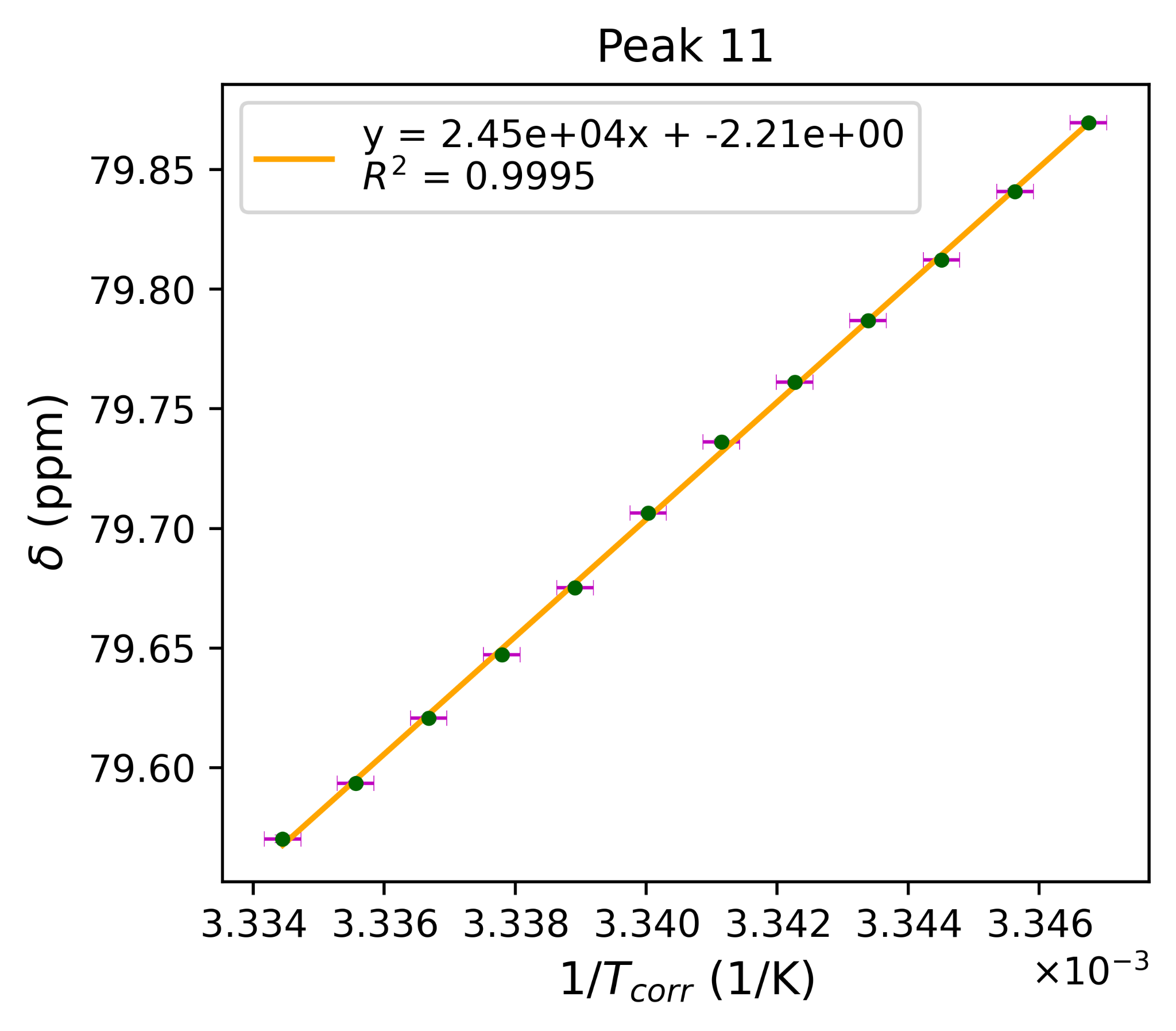 | 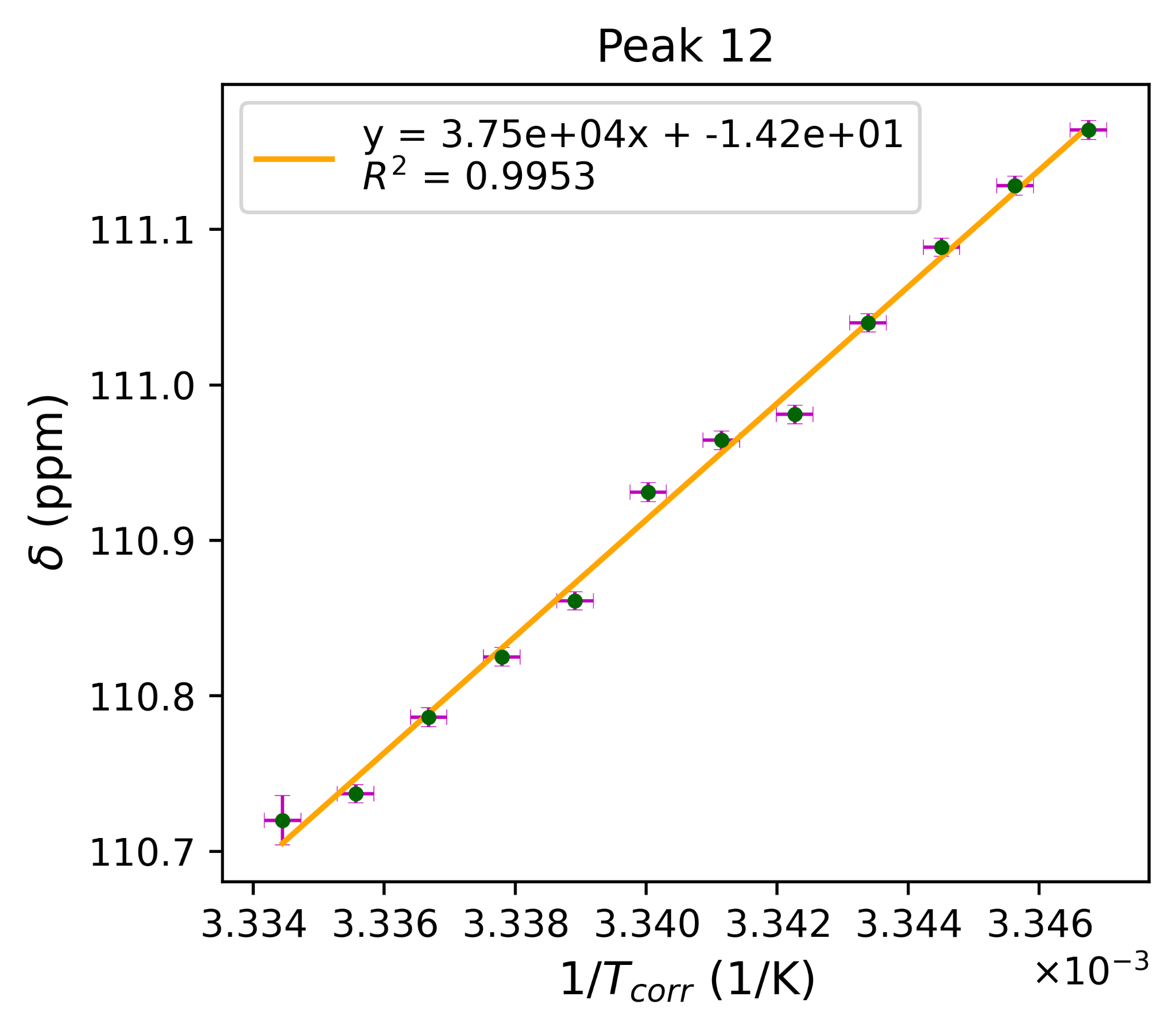 |
| 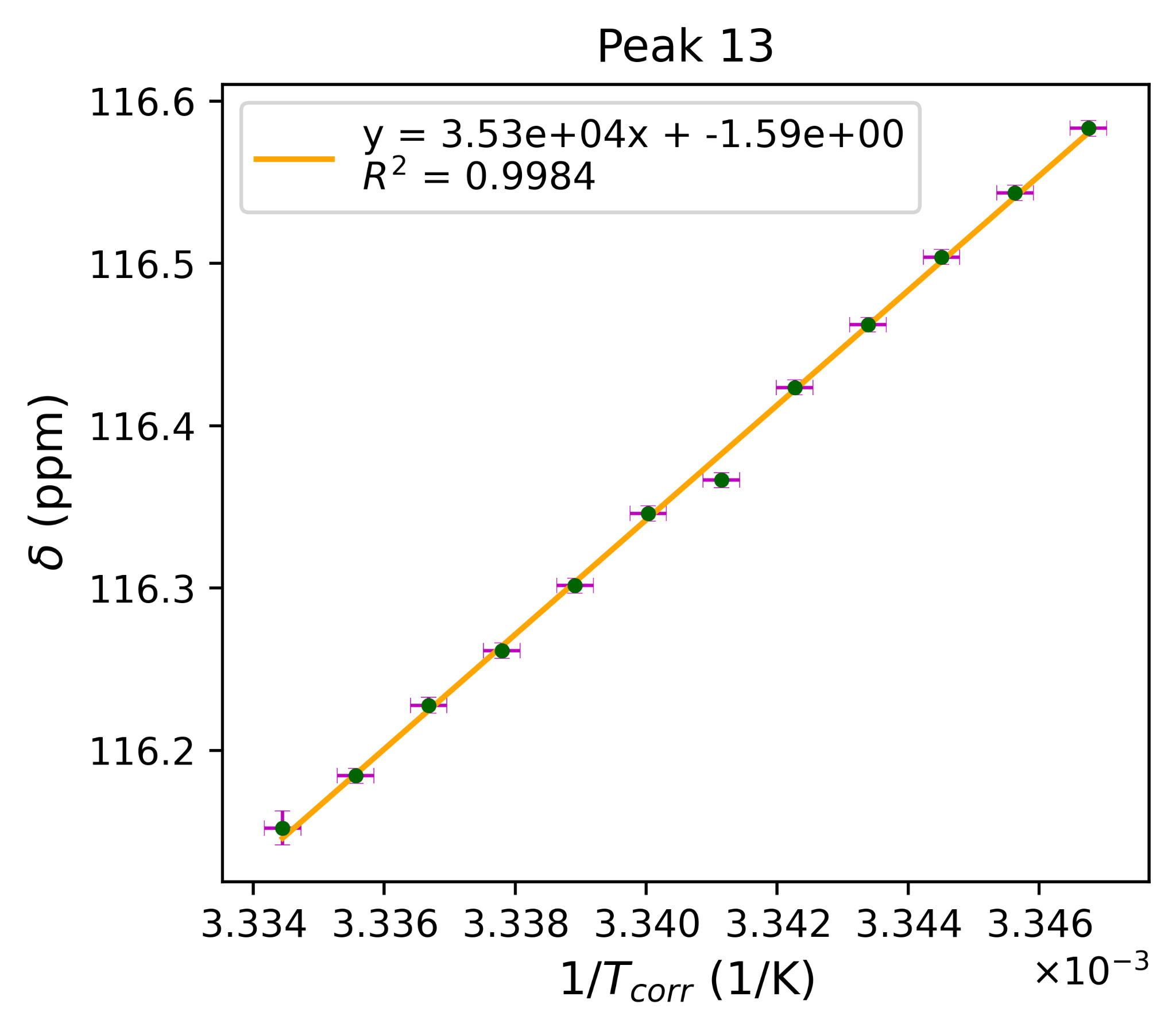 | 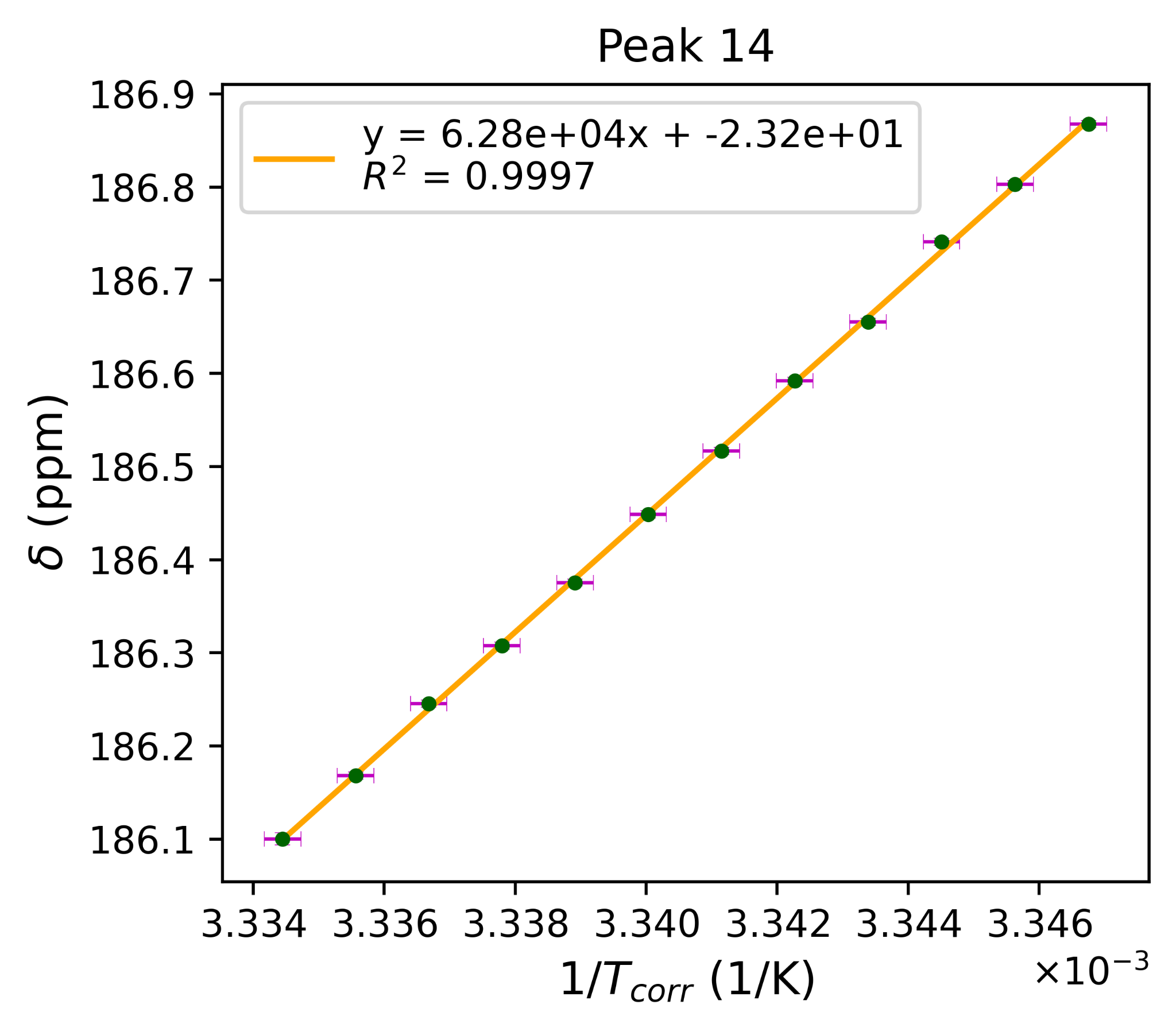 |
| 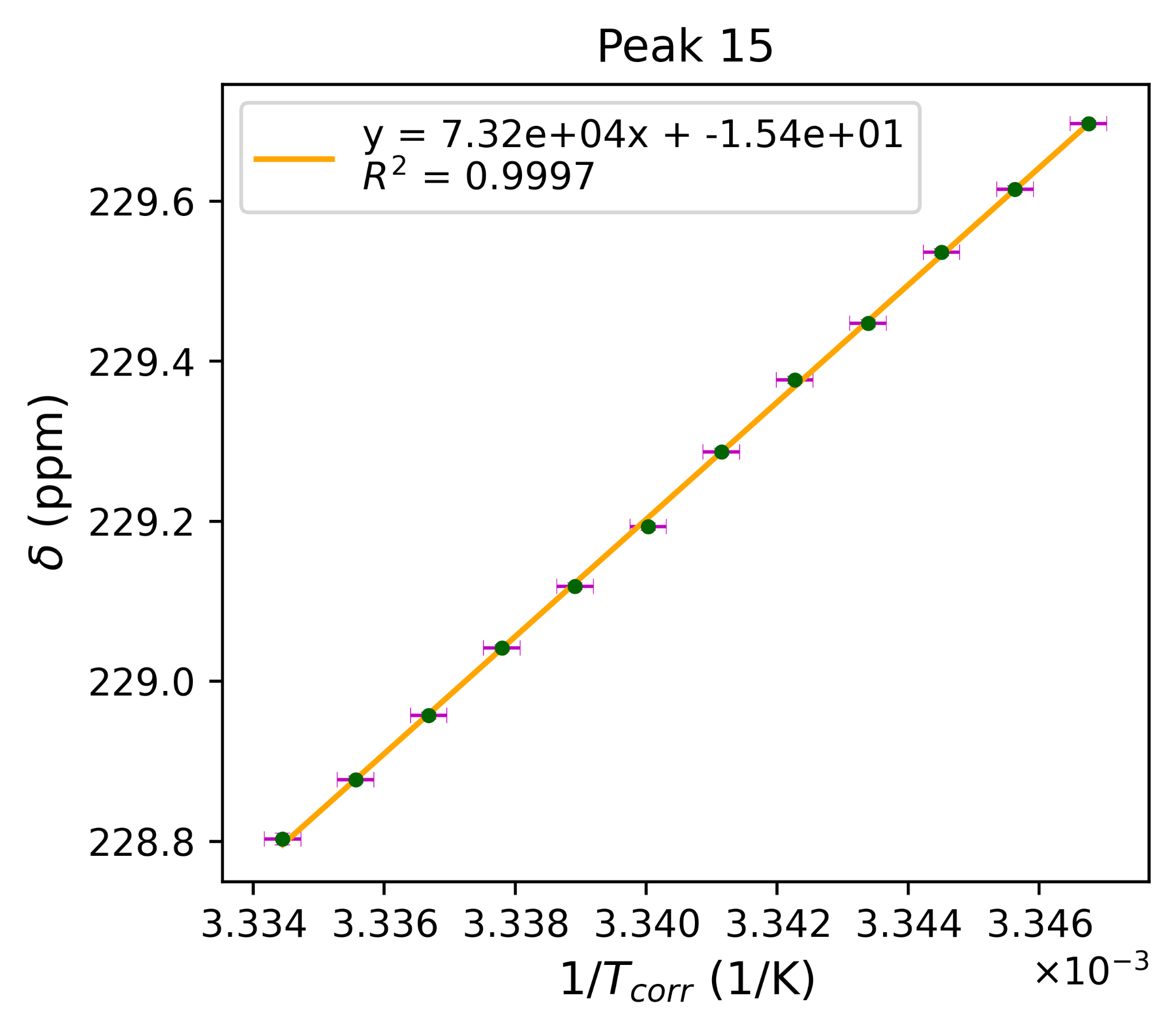 | 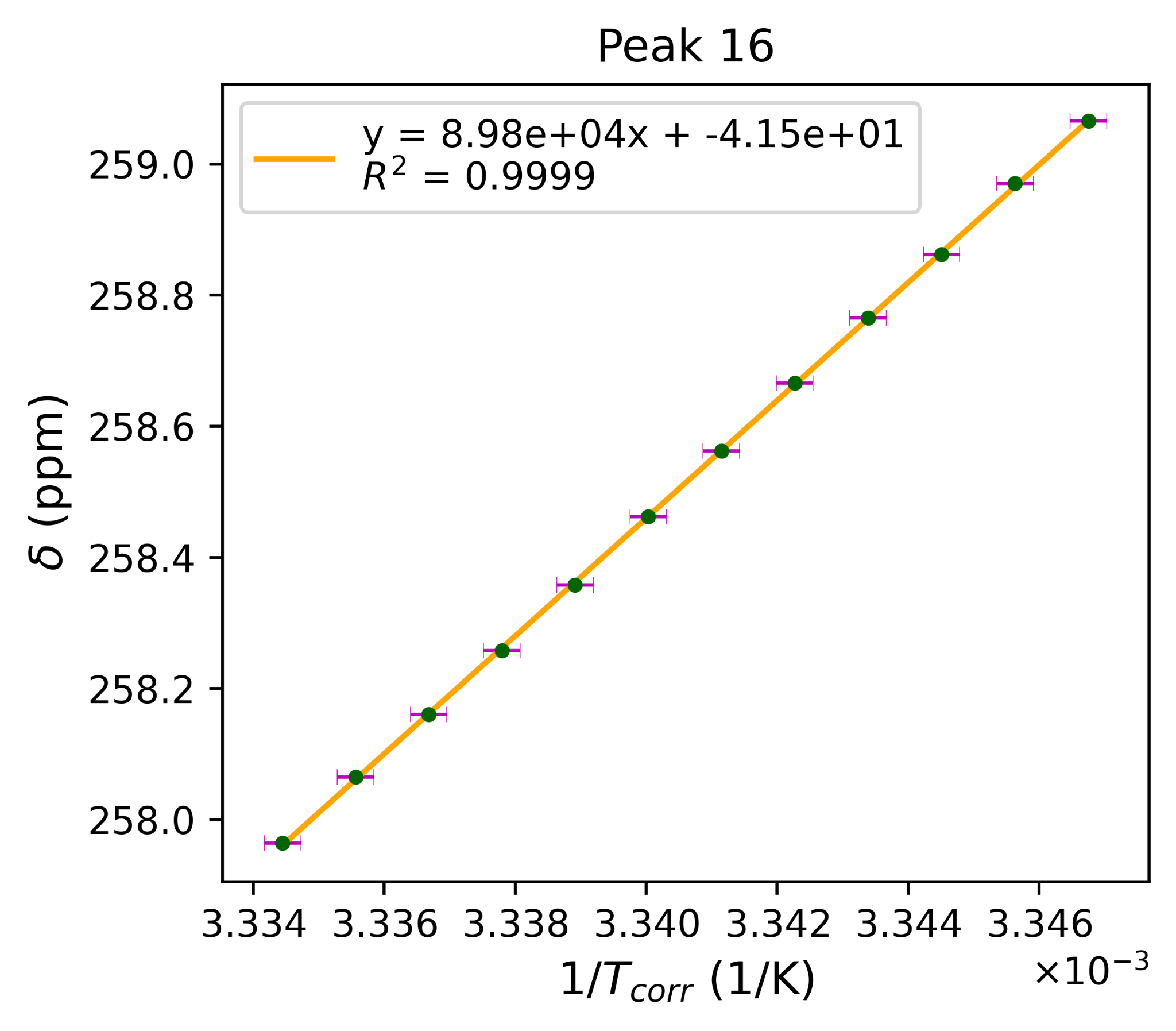 |
| 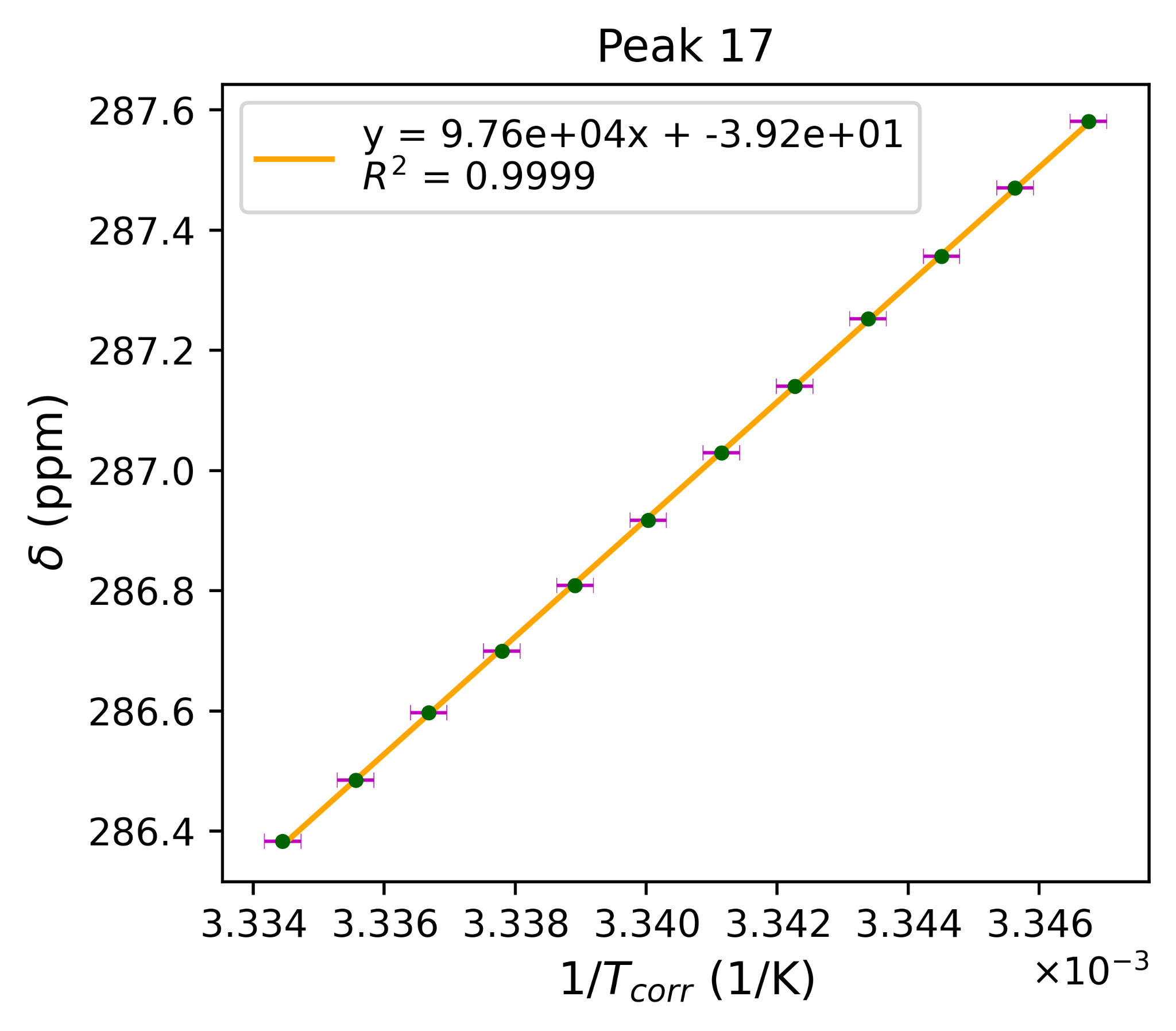 | 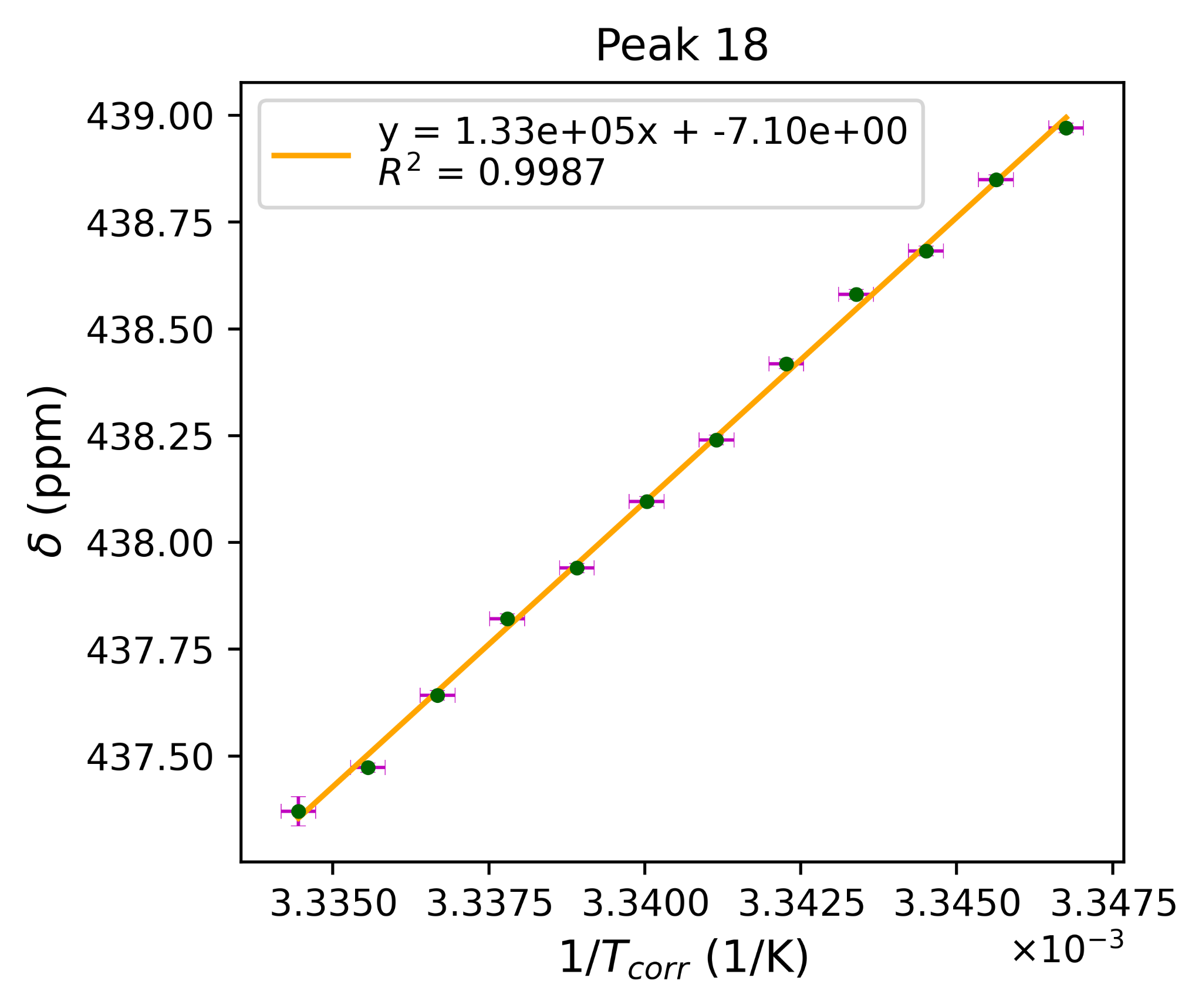 |
| 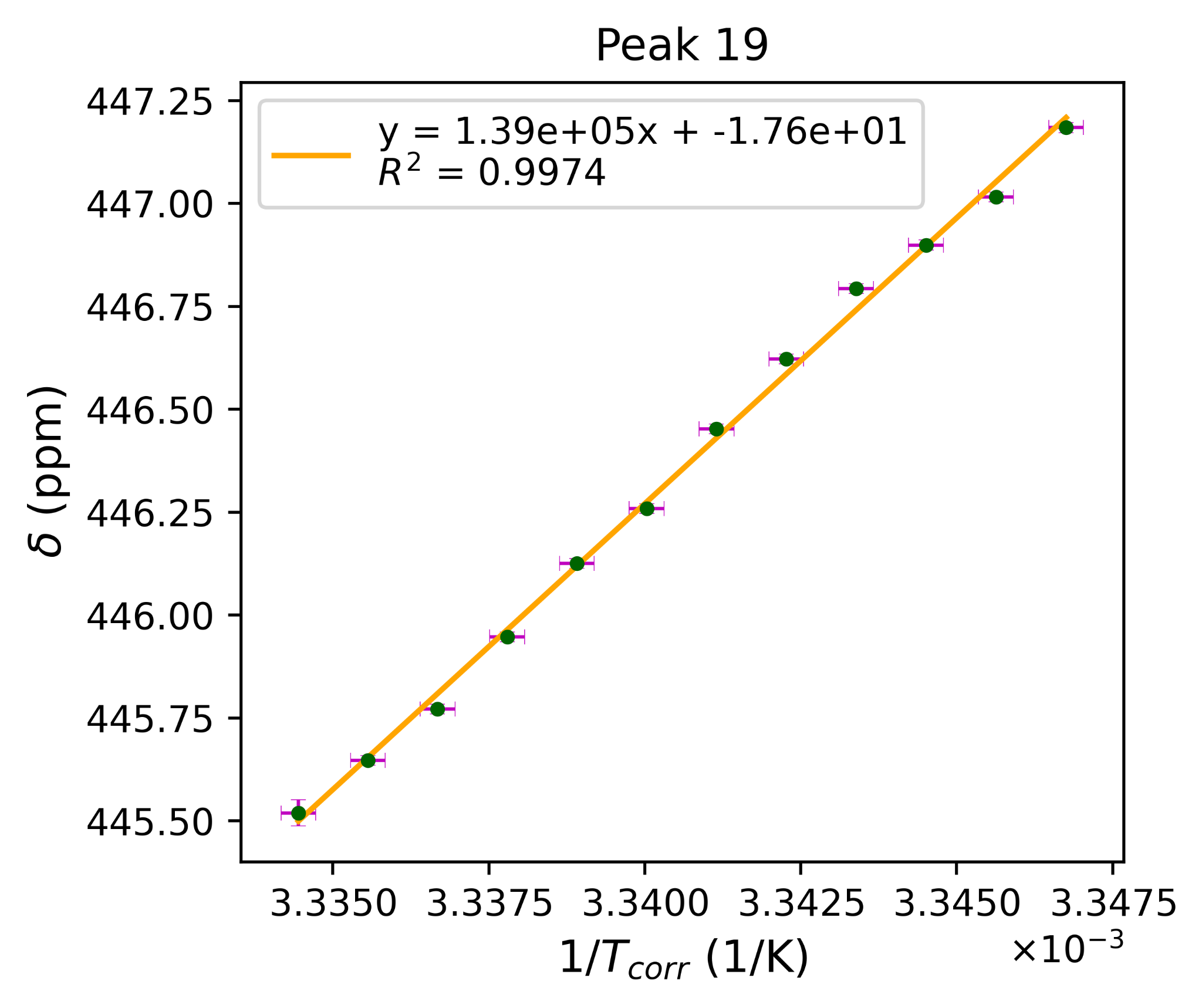 |  |

# S3. FDX2 temperature dependence of the hyperfine shifts

## S3.1 Extraction of chemical shift values

| Listing S3.1.1: Code listing for the extraction of the ^13^C chemical shifts. Notice that the temperature has been corrected according to a previously performed calibration, as described in section 4.2.1 of the manuscript. |
| --- |
| from f_fit import *  import numpy as np  def cal_temp(T):  #temperature calibration at 1200 MHz  T_cal = []  for t in T:  T_cal.append(t*0.955944+9.81982517+2)  return T_cal  path = 'path/to/spectra/'  num_sp = list(np.arange(23,31,1))  list_sp = [str(i)+'/pdata/10' for i in num_sp]  temp = []  for idx in range(len(list_sp)):  _, _, ngdicp = nmr_spectra_1d(path+list_sp[idx])  temp.append(ngdicp['acqus']['TE'])  temp = cal_temp(temp) #calibration of the temperature at 1200 MHz  temp = np.array(temp)  lim1 = {'shift':(-4,4), 'lw':(1e-4,2.5), 'ph':(-np.pi/10,np.pi/10), 'k':(0,1)}  lim2 = {'shift':(0.5,1.5),'k':(0,0),'lw':(0,0), 'ph':(0,0), 'xg':(0,0), 'A':(0.9,1.1), 'B':(0.9,1.1), 'C':(0.9,1.1), 'D':(0.9,1.1), 'E':(0.9,1.1)}  dir_res, _, shift_tot, temp, *_ = model_fit_1D(  path,  temp,  list_sp,  cal_lim = None,  dofit=True,  prev_fit = None,  fast = False,  limits1 = lim1,  limits2 = lim2,  L1R = 1000,  L2R = None,  Param = "shift",  procpars = {'SSB':2}  )  shift_tot = shift_tot[0] |

| Figure S3.1.1: Fit result plots and corresponding residuals histogram for the temperature serie of ^13^C spectra of ferredoxin sample. | |
| --- | --- |
| 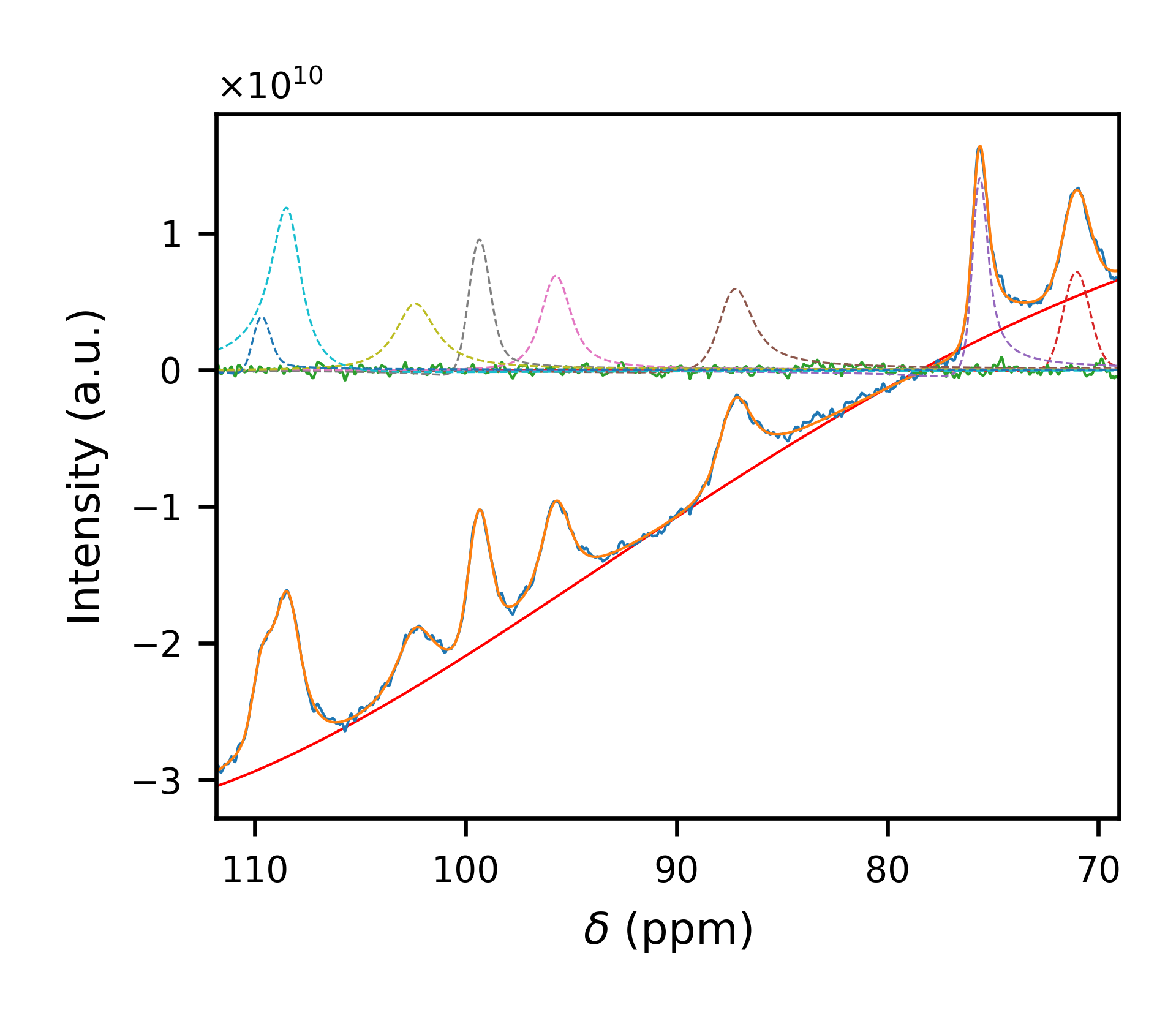 | 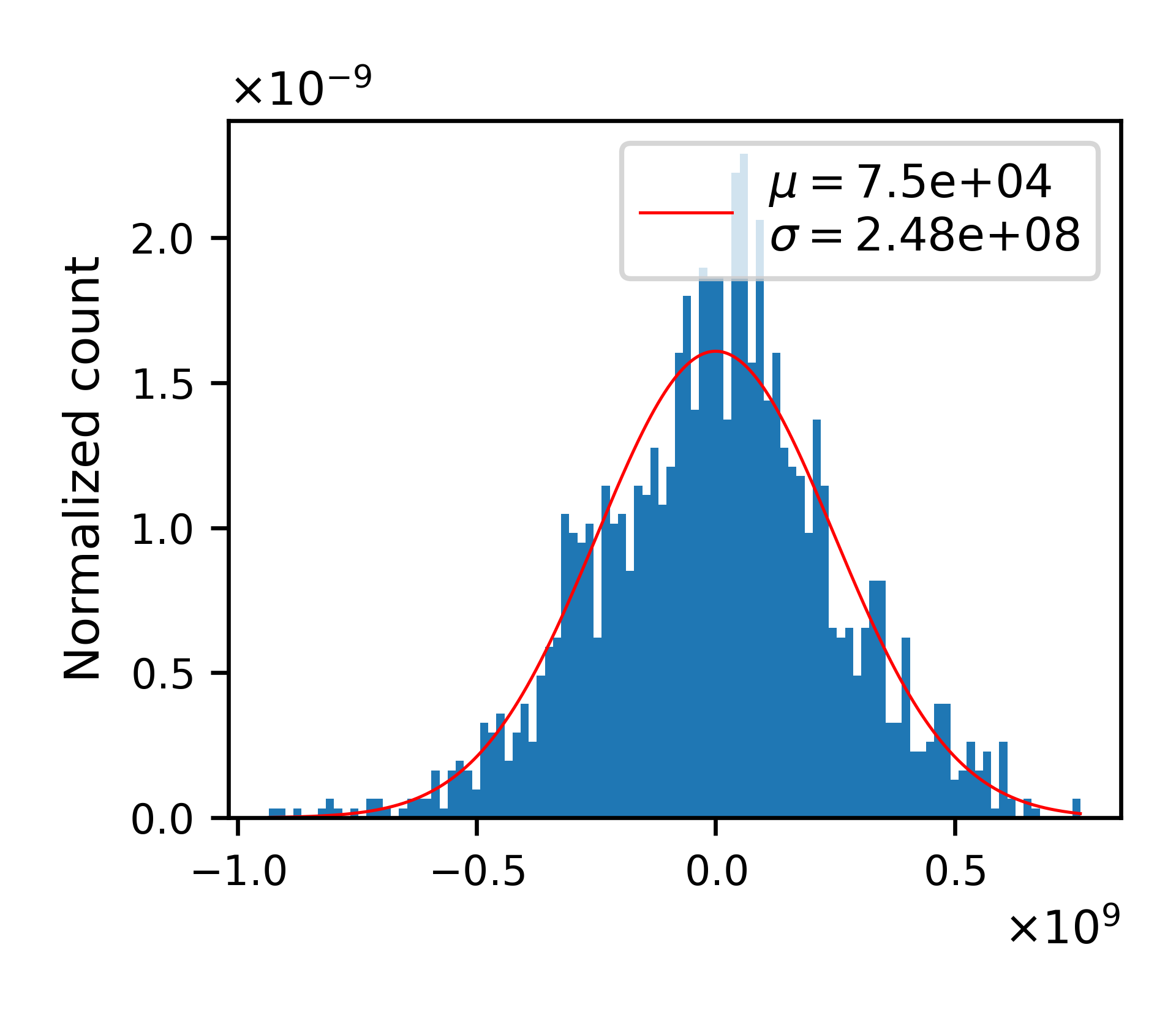 |
| 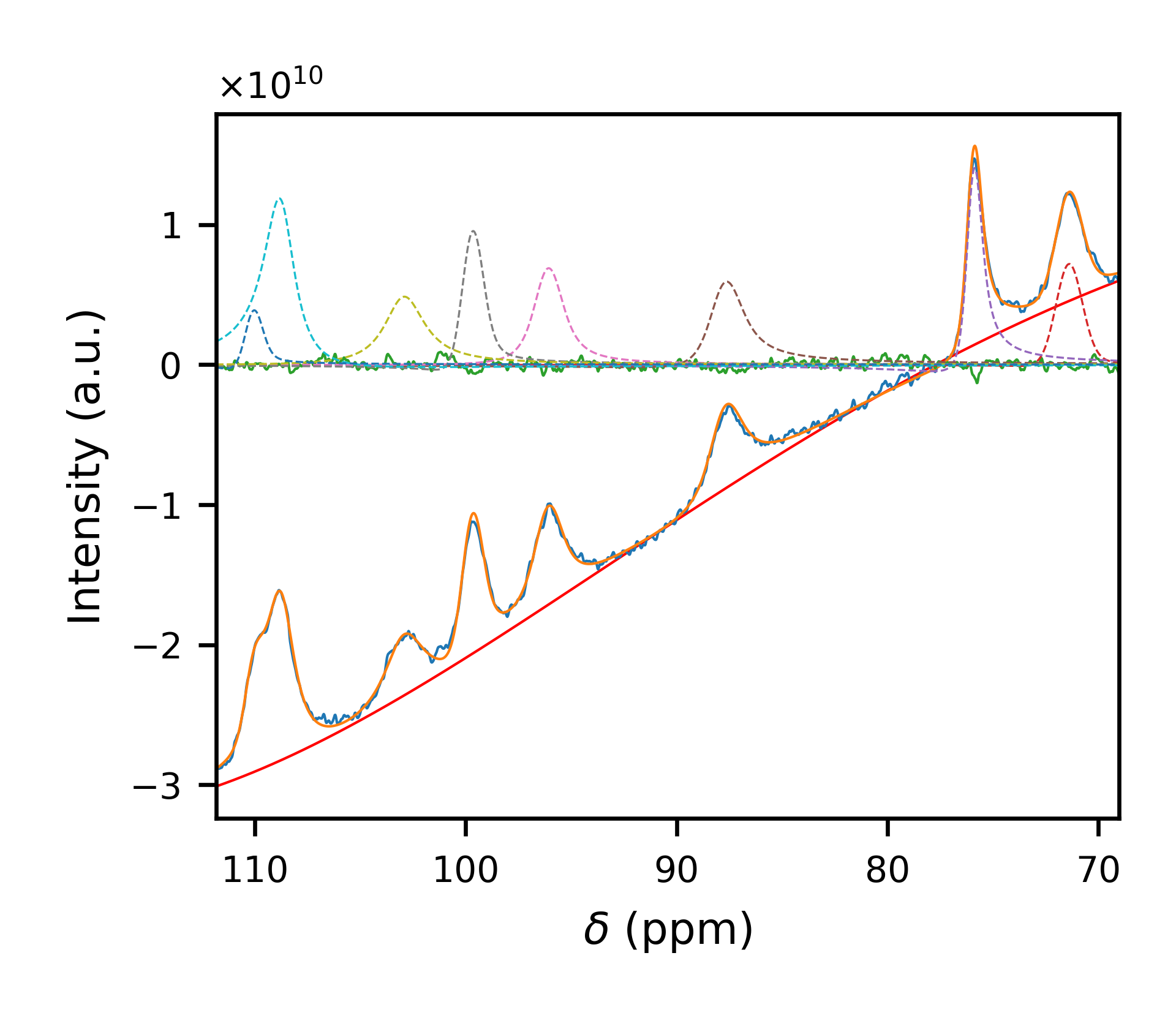 | 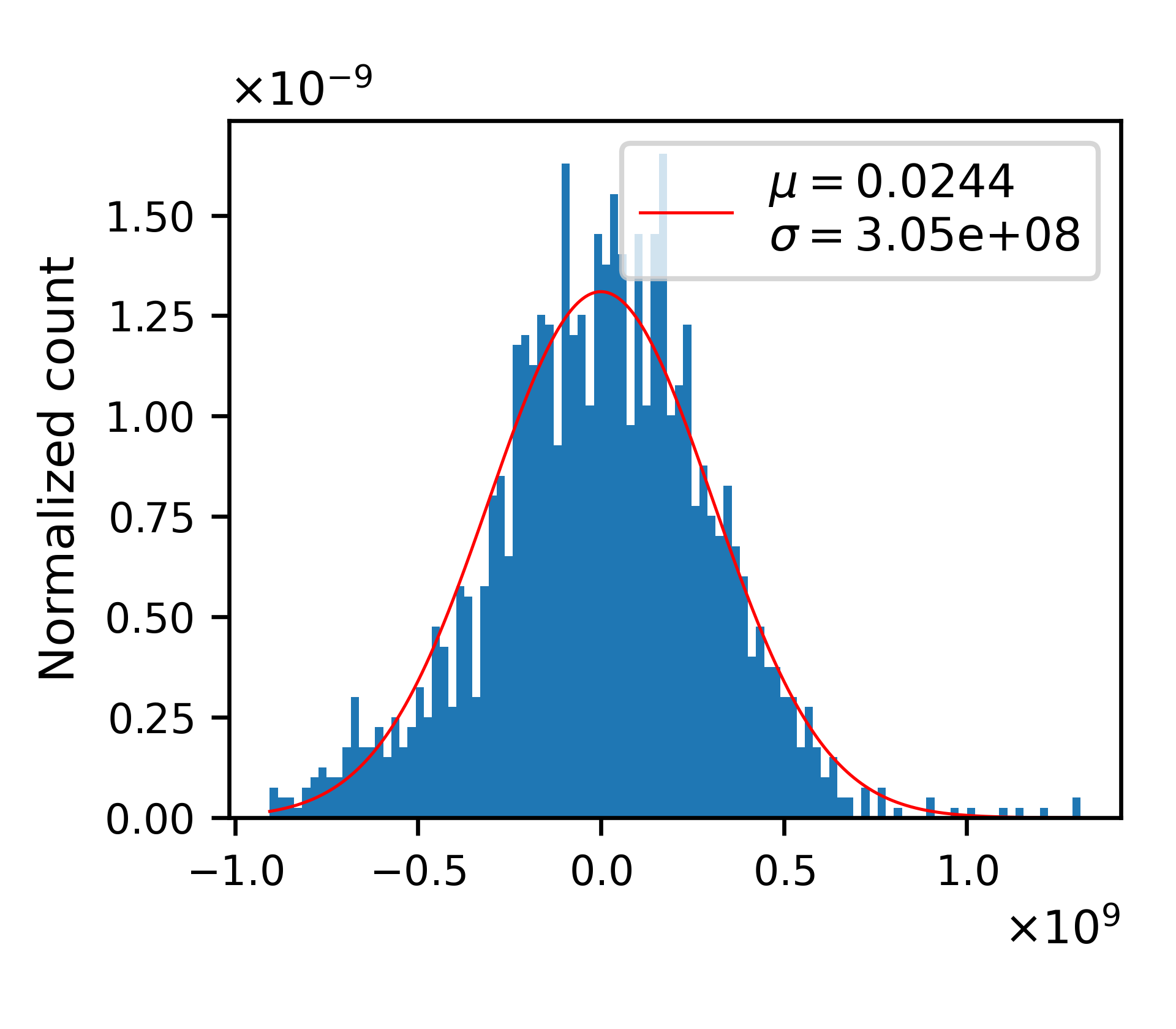 |
| 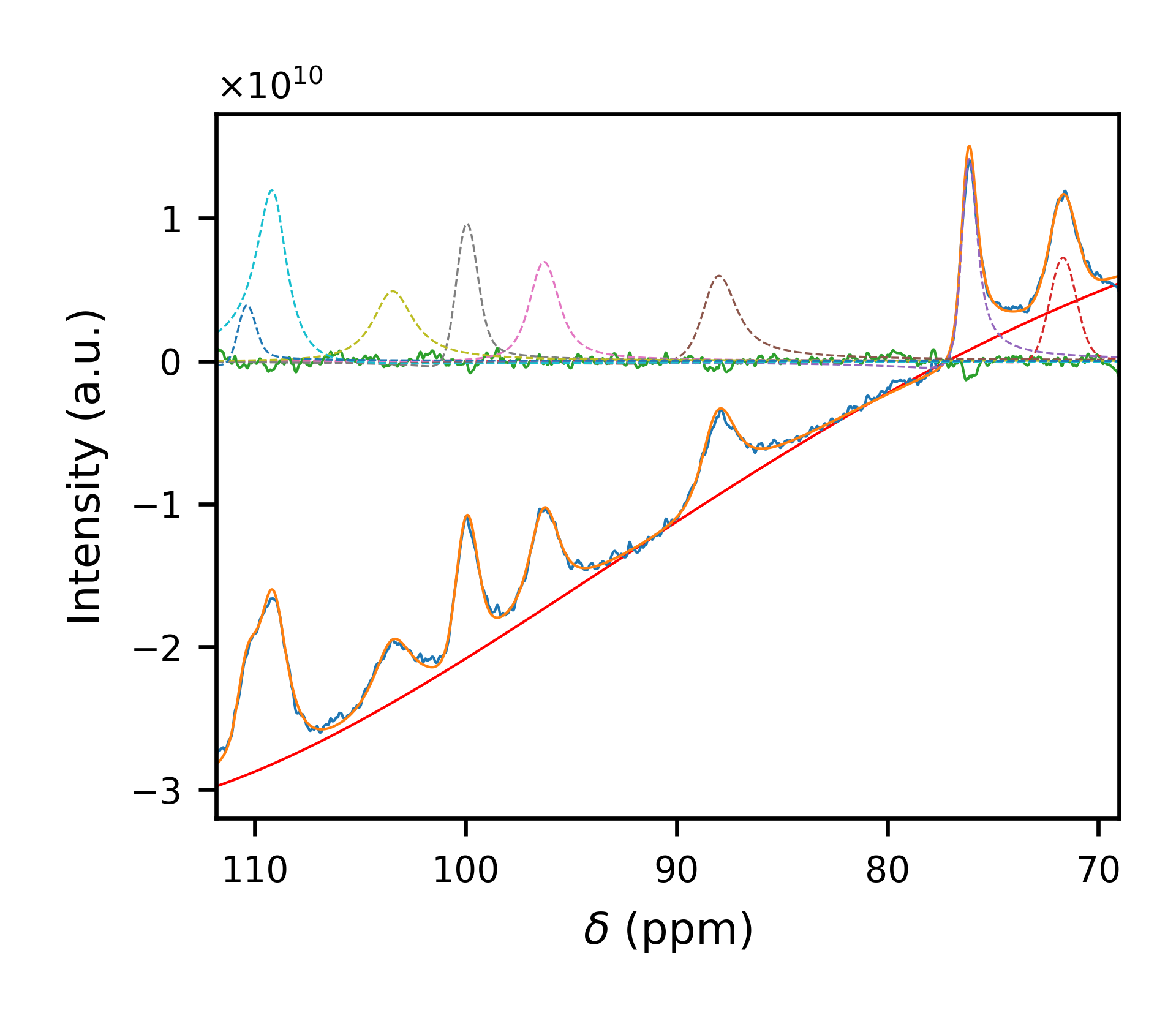 | 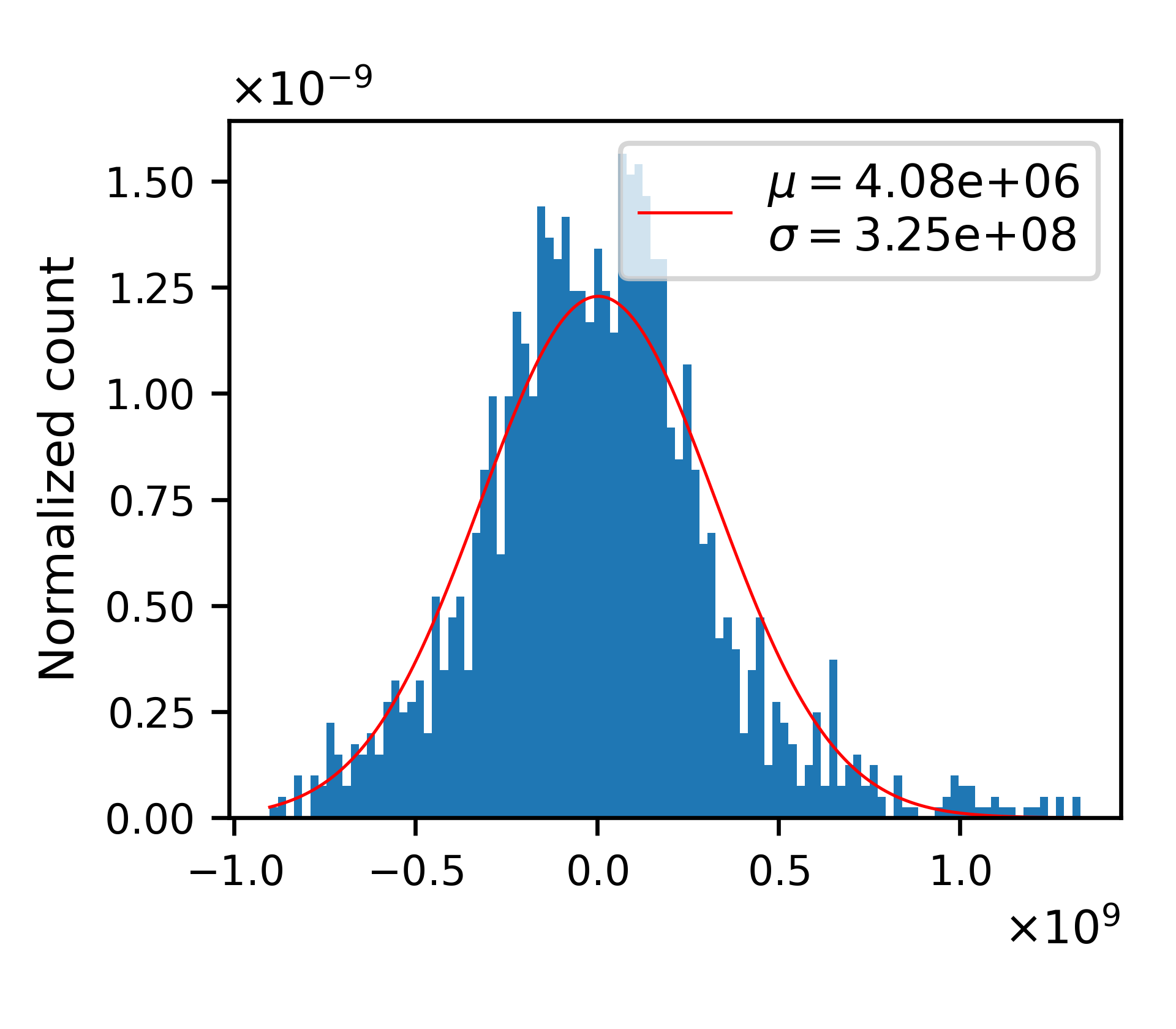 |
| 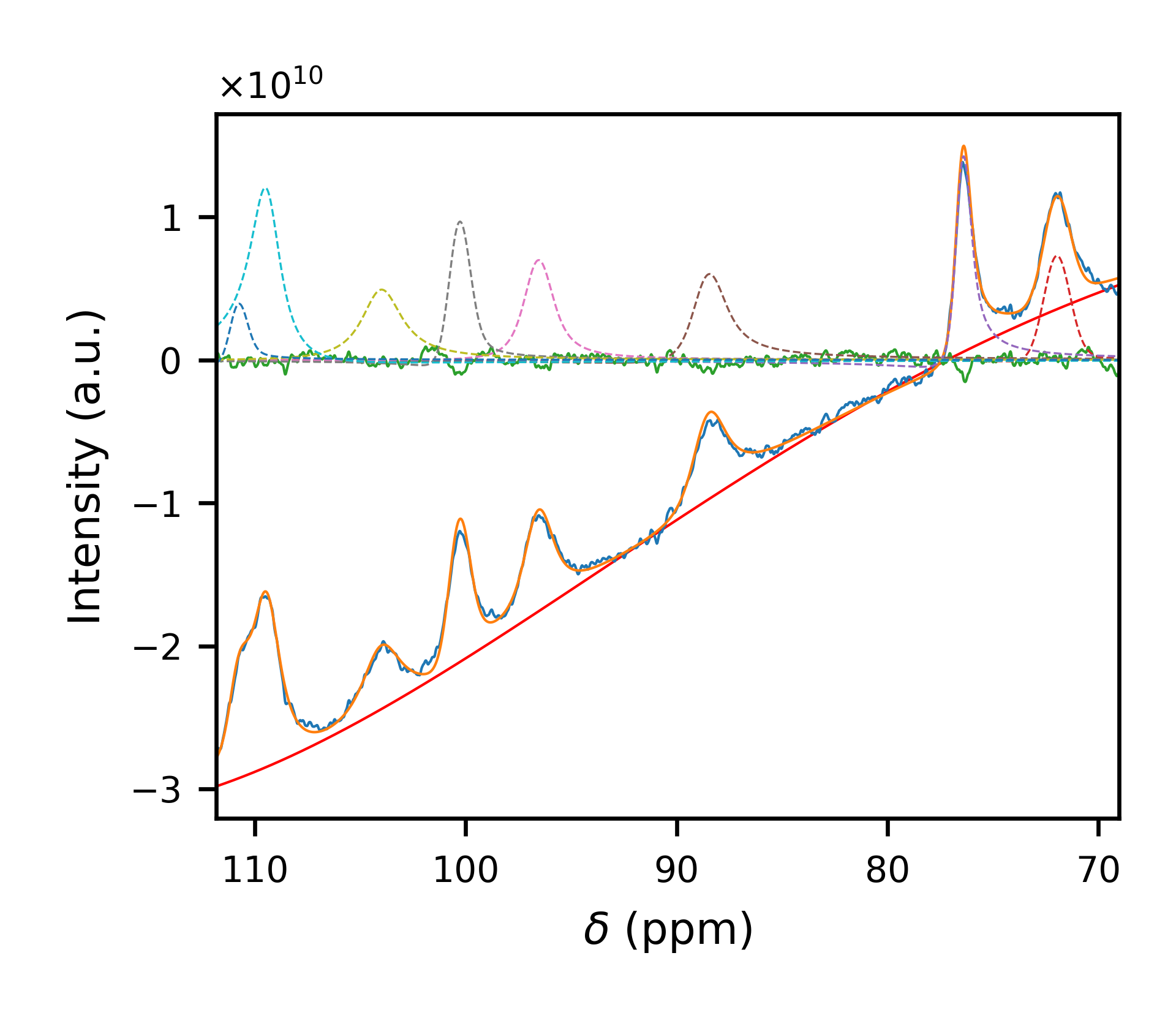 | 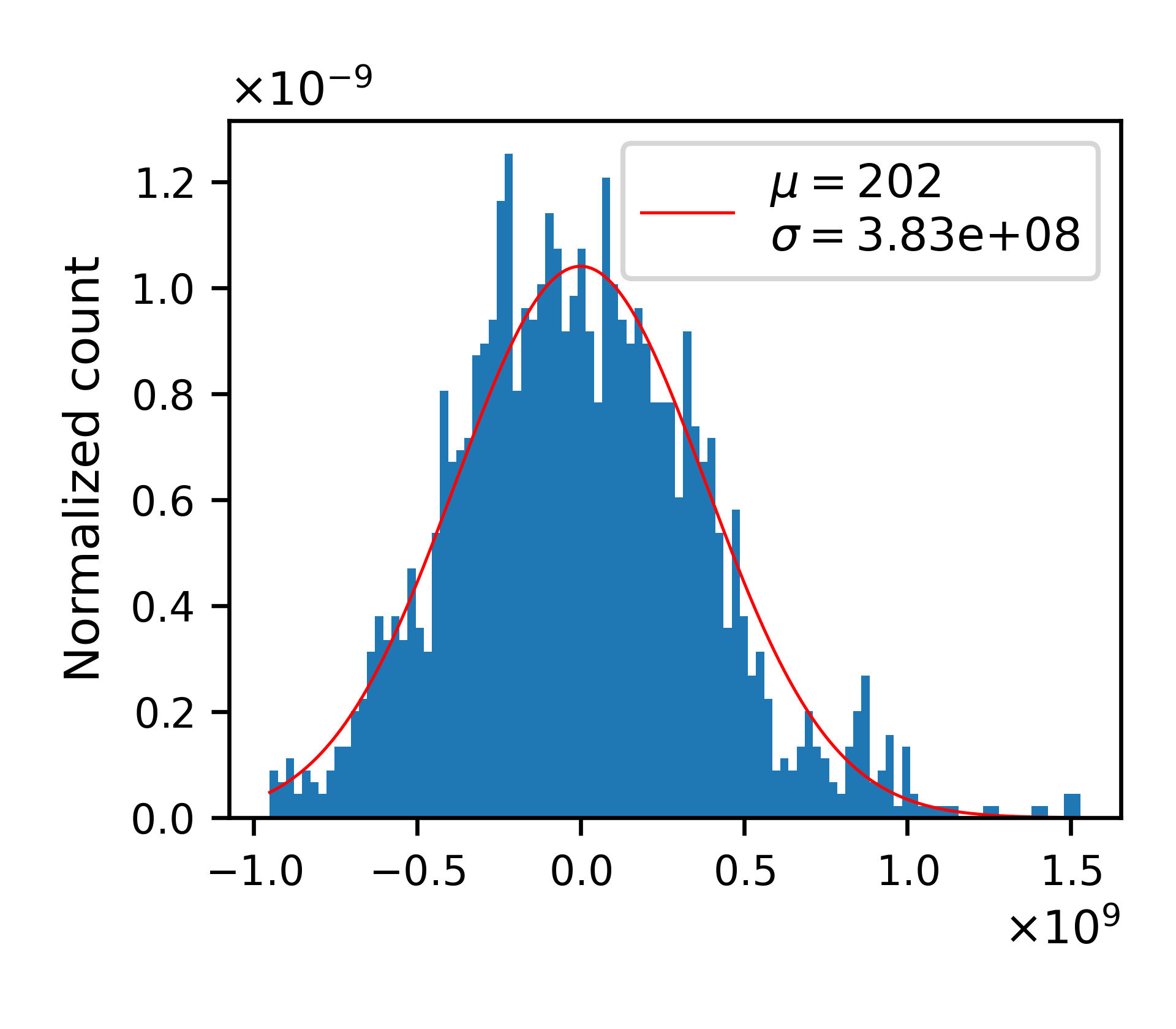 |
| 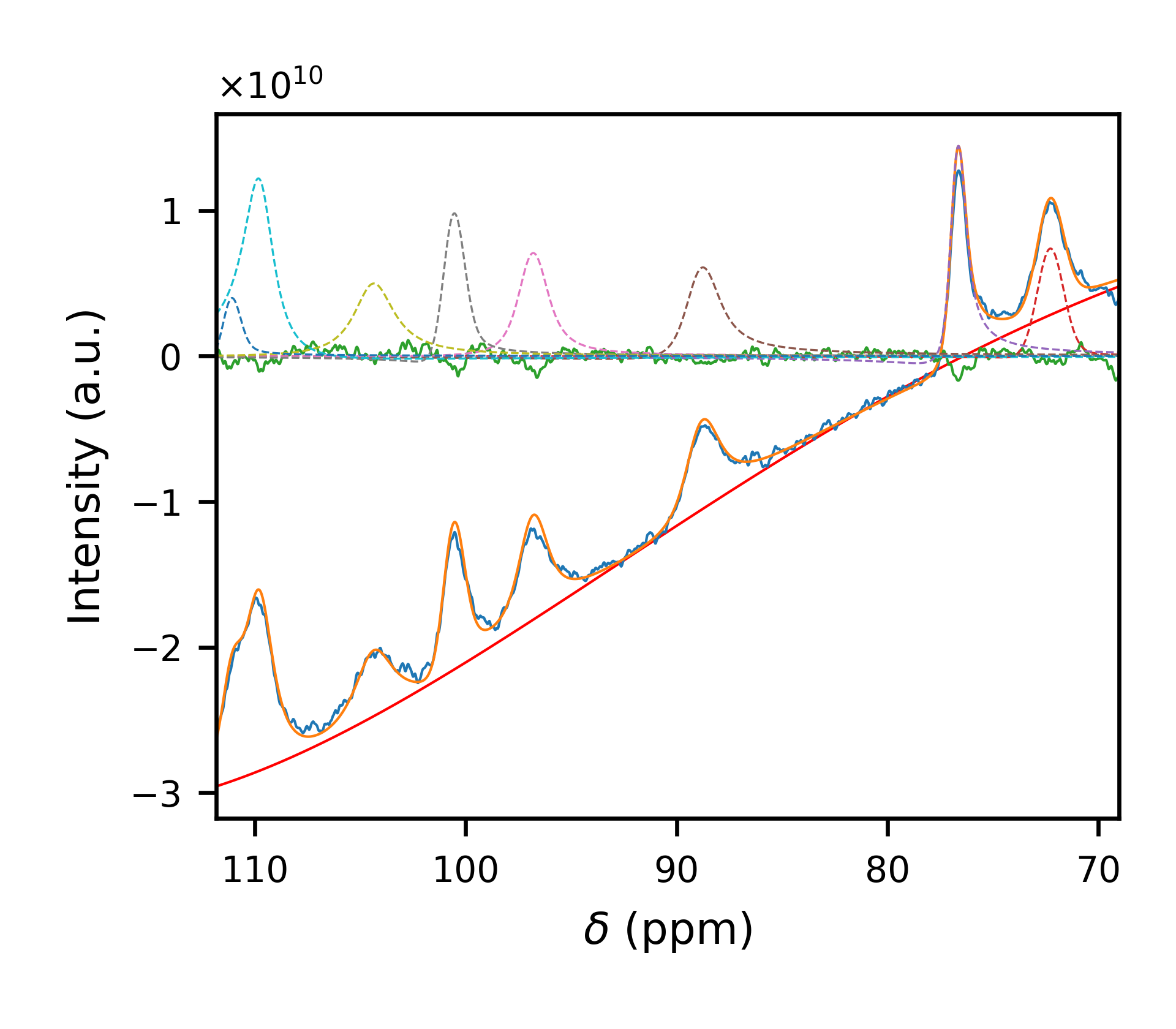 | 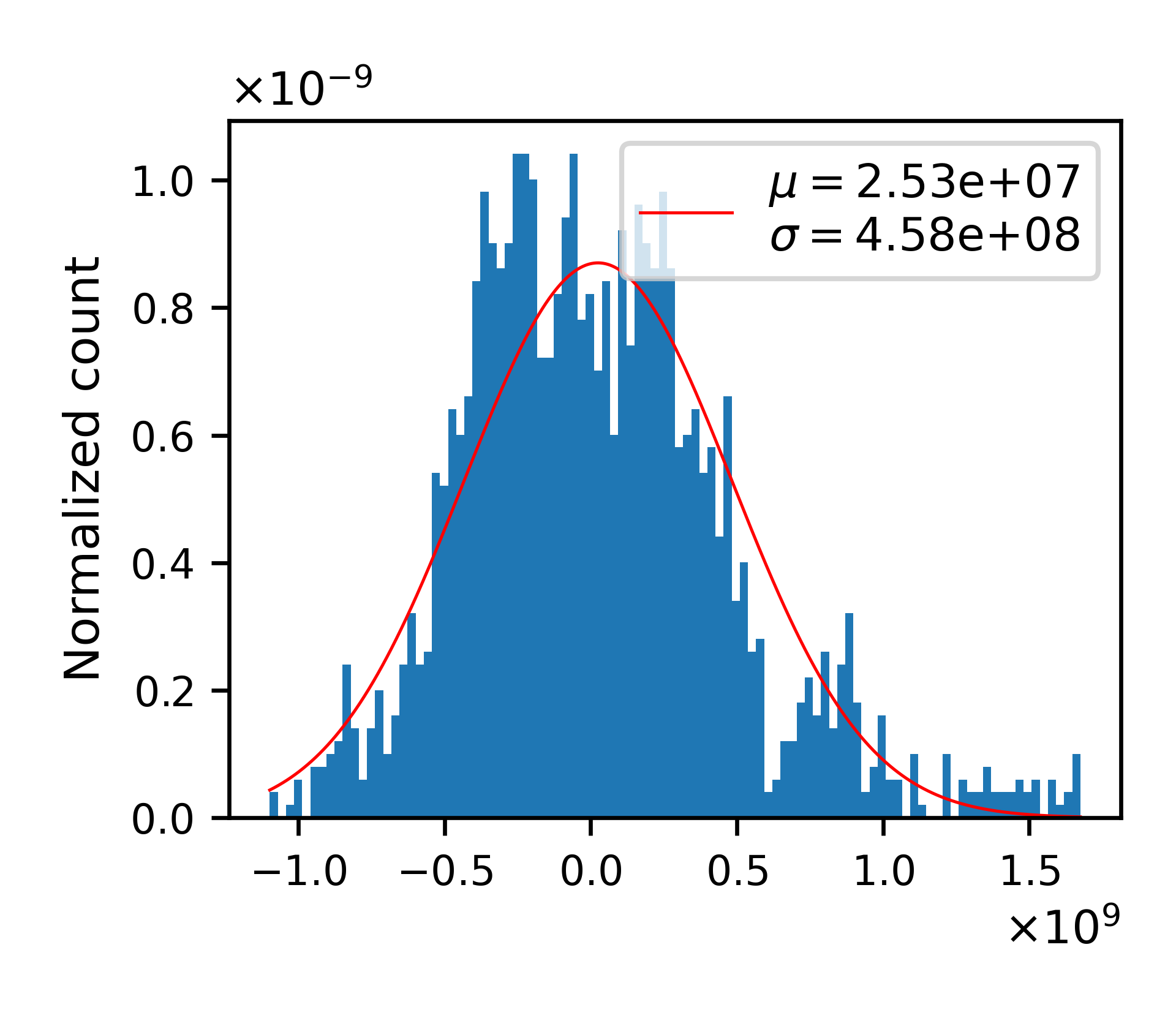 |
| 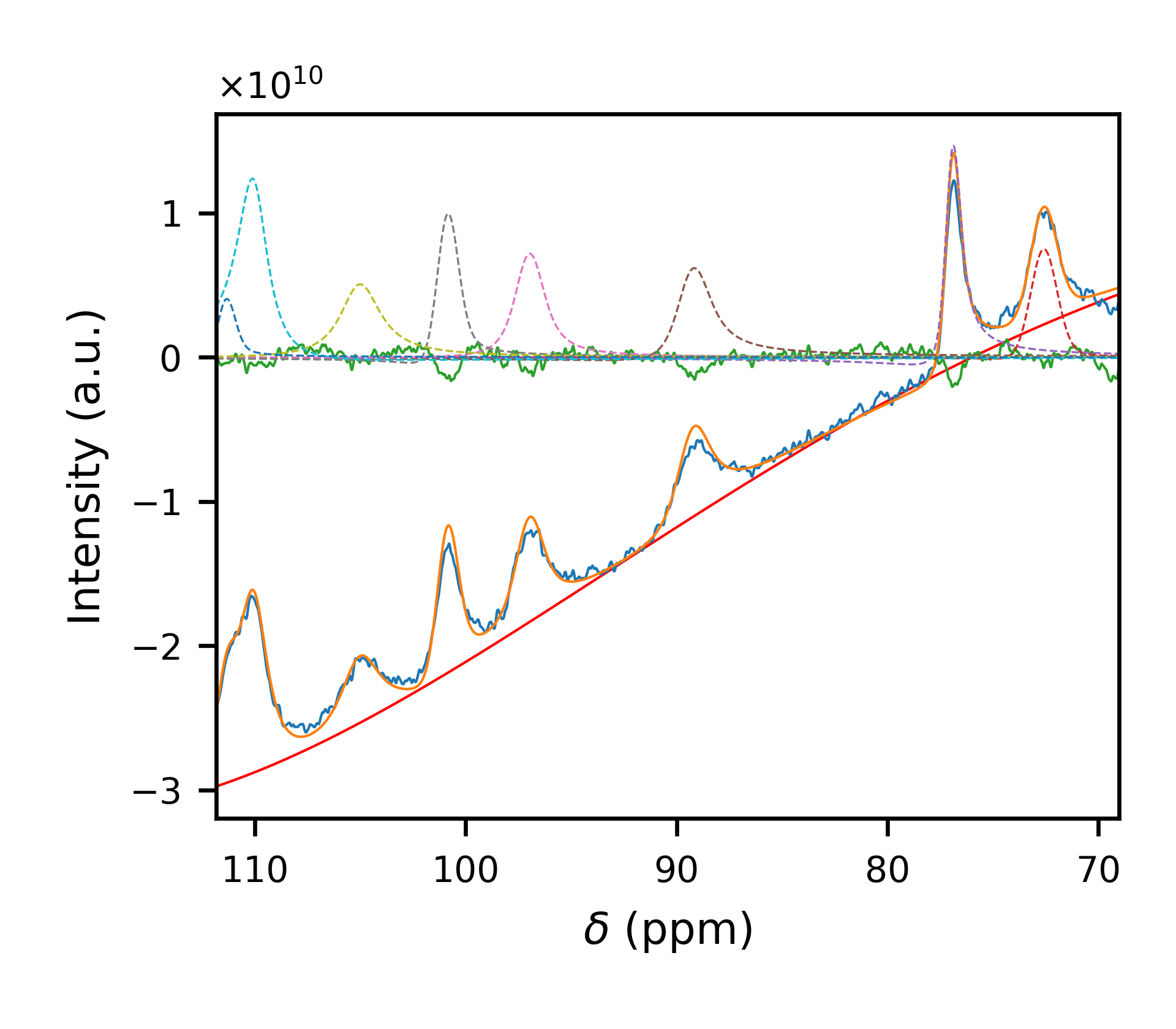 | 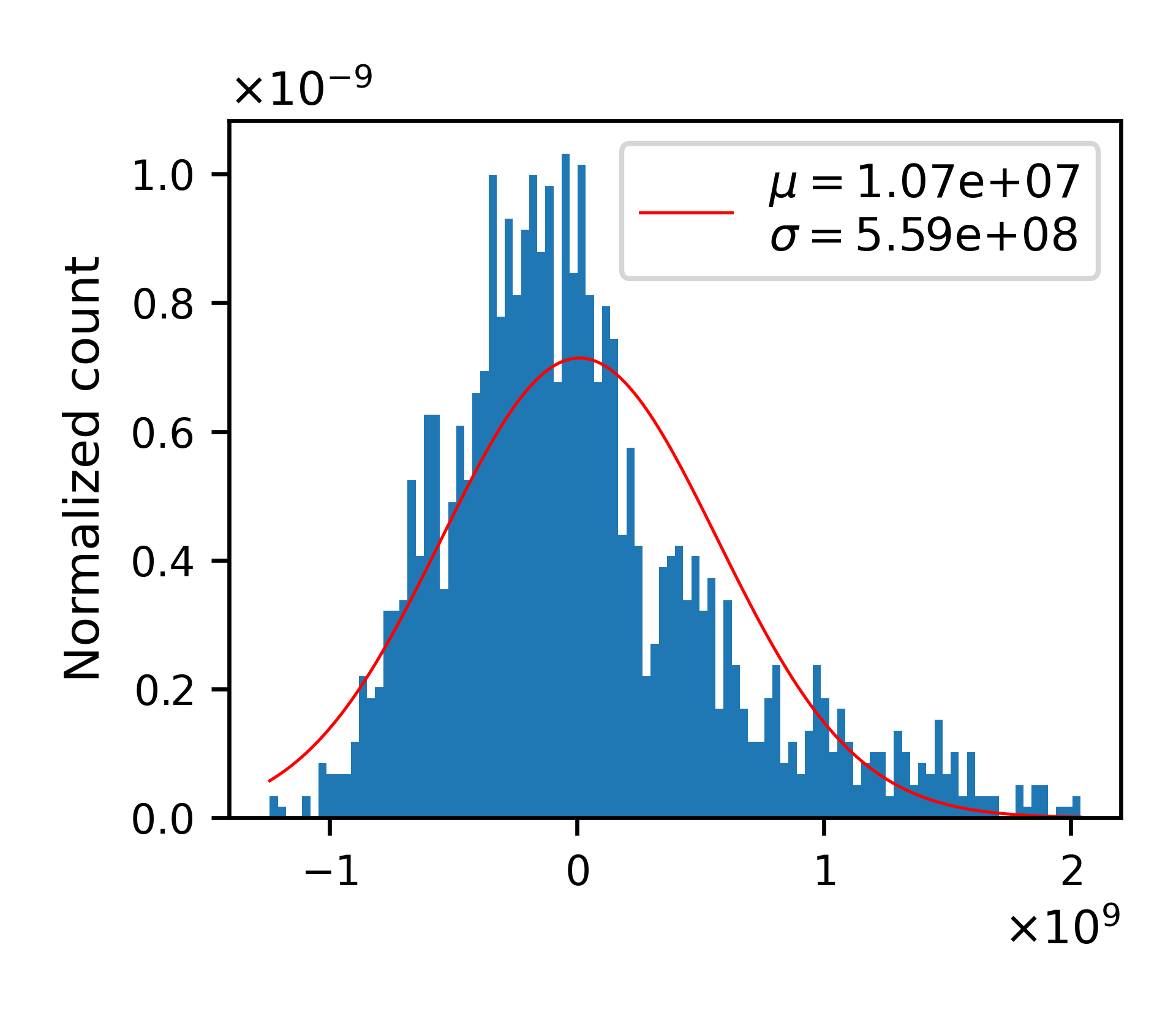 |
| 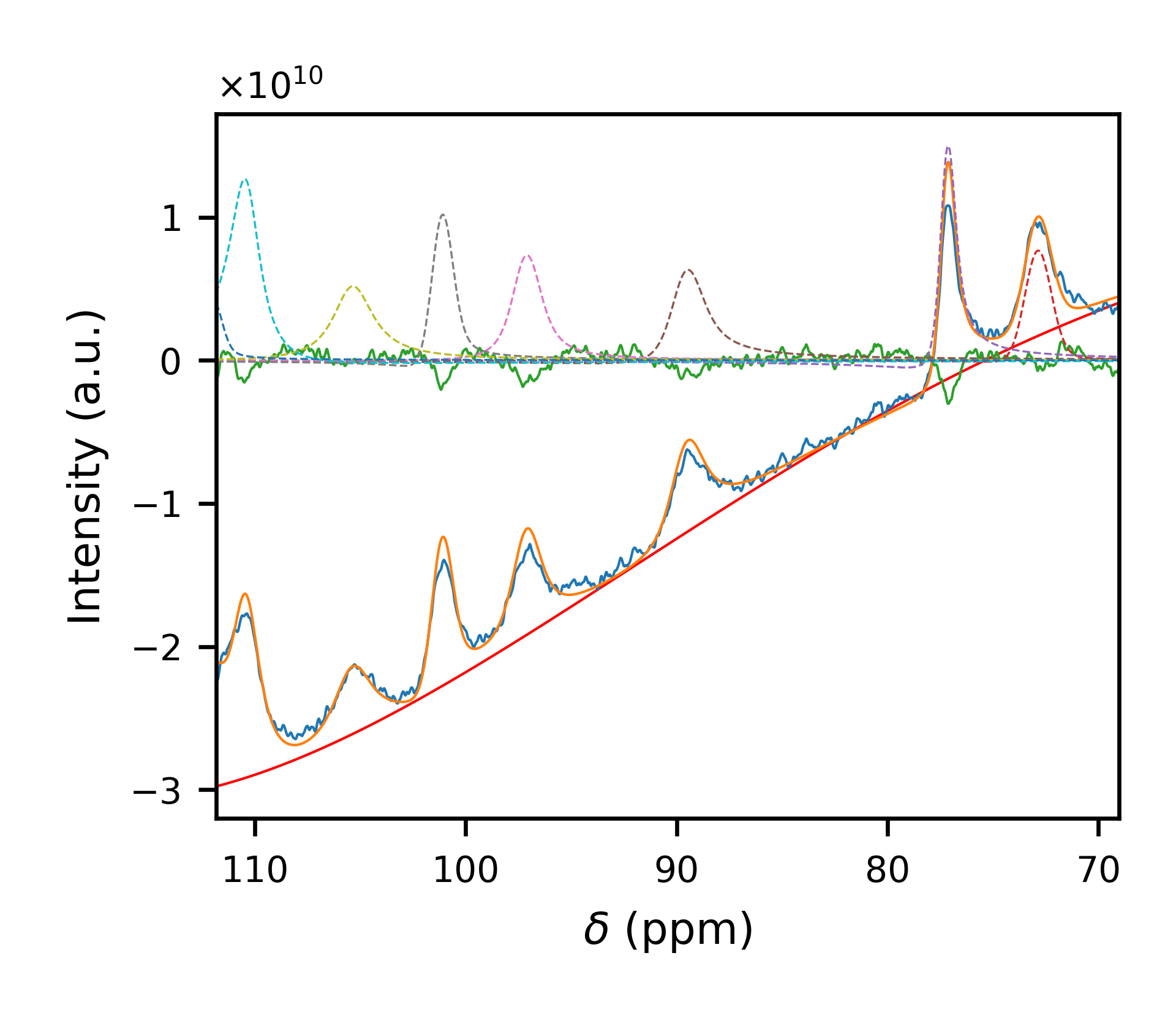 | 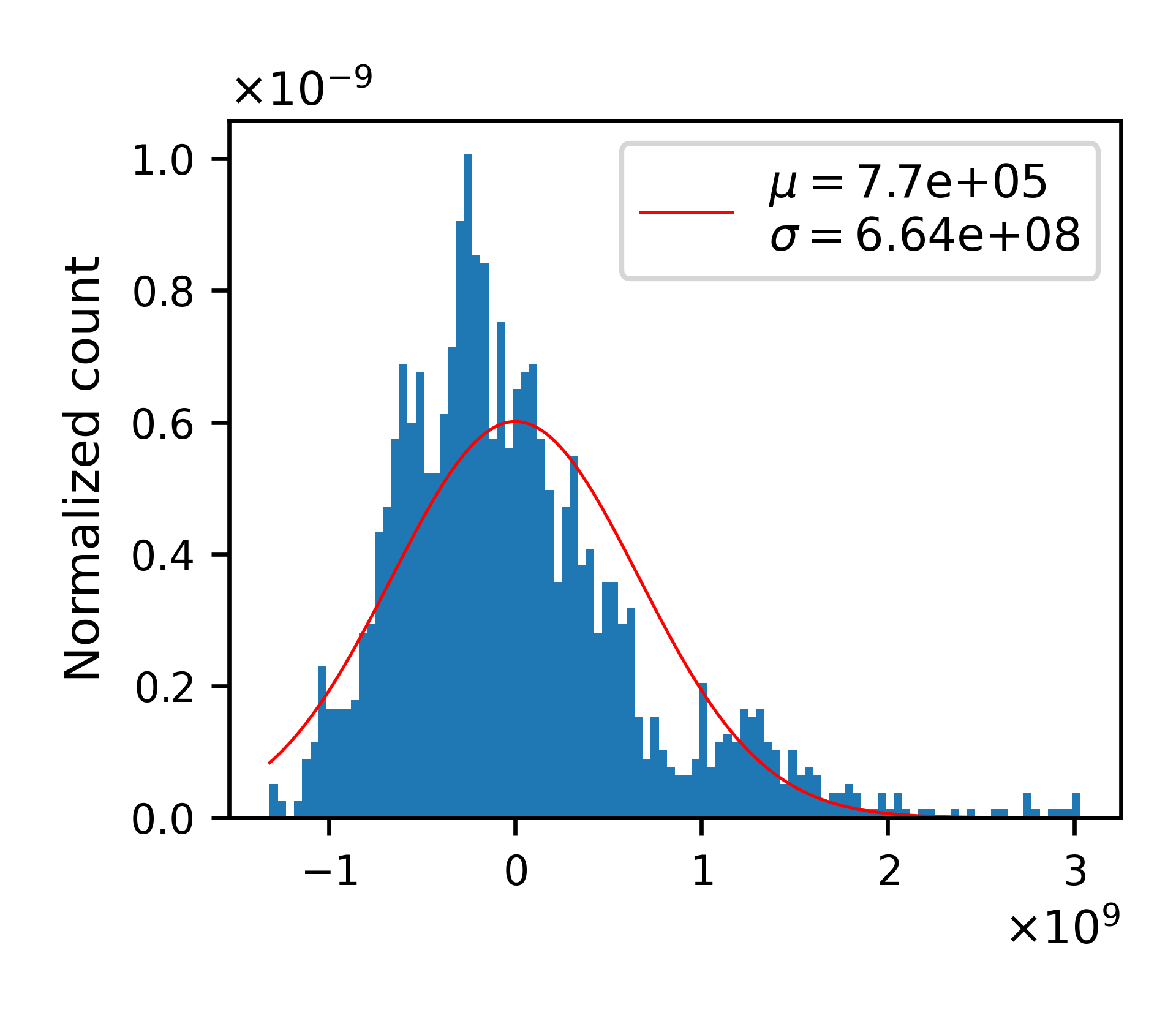 |
| 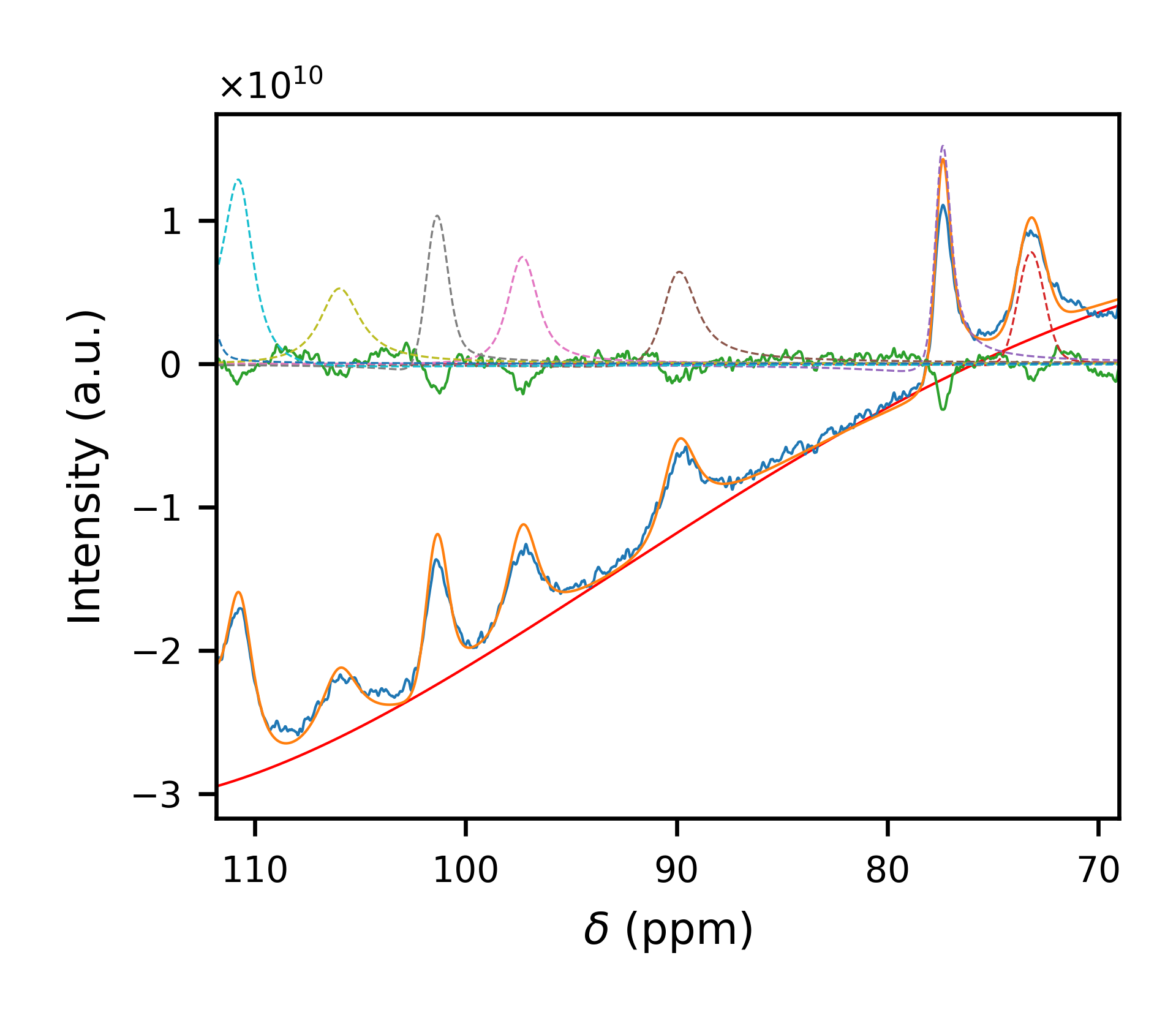 | 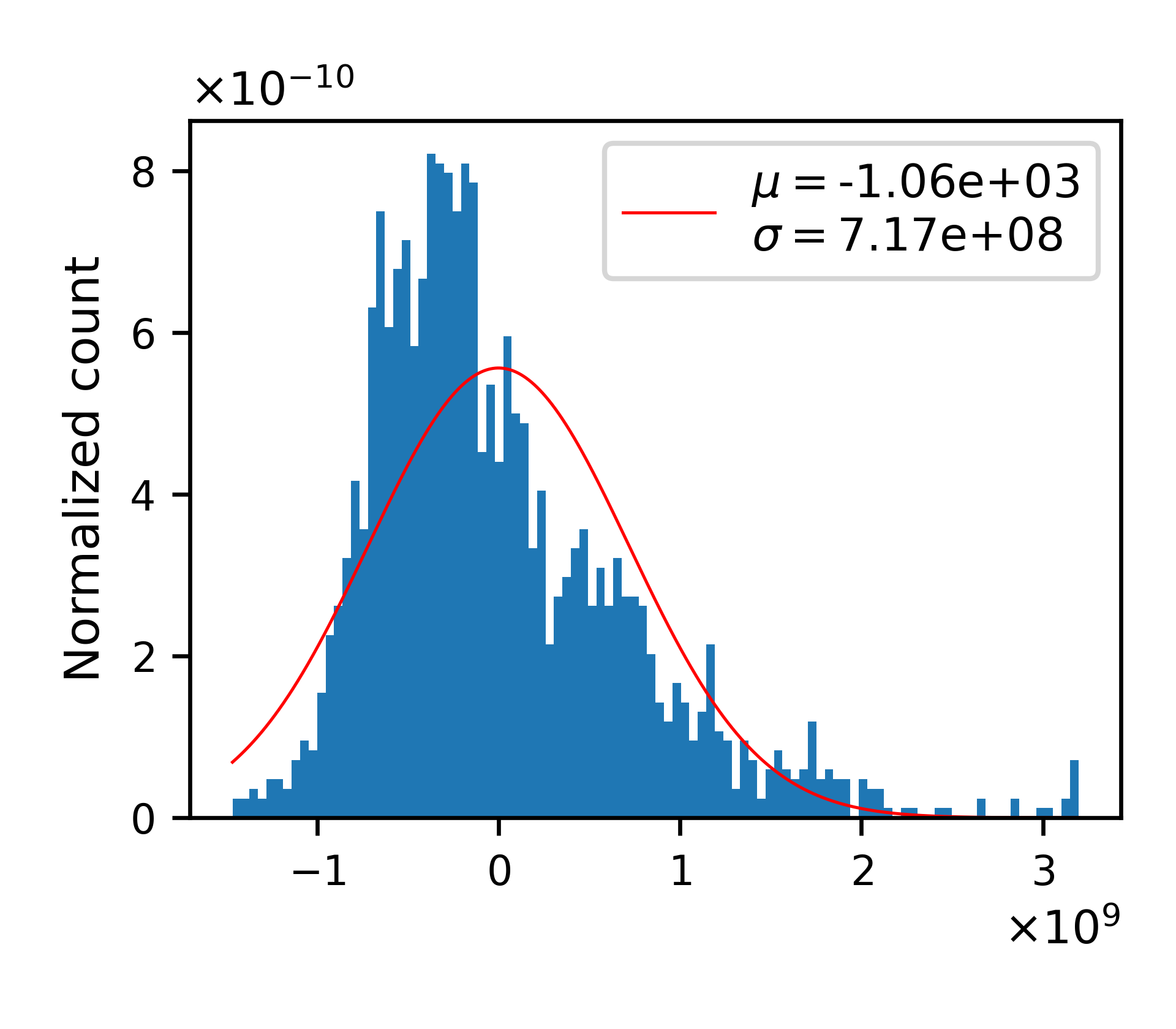 |

## S3.2 Modeling of chemical shift field dependence in exchange coupled systems

| Listing S3.2.1: Code listing for the fit of the ^13^C chemical shift of ferredoxin protein. |
| --- |
| import matplotlib.pyplot as plt  import numpy as np  import scipy.constants  import lmfit  def calc_shift(temp, S2, S3, A_h, J, gammaC=267/4):  def energy(Si, J):  return 1/2*J*(Si*(Si+1))  conv = 1.9865e-23 # from cm-1 to J  kB = scipy.constants.k  muB = scipy.constants.physical_constants['Bohr magneton'][0]  ge = 2.0023  pref = 2*np.pi*ge*muB/(3*kB*gammaC*temp)  sum1 = 0  sum2 = 0  for s in np.arange(np.abs(S2-S3), S2+S3+1, 1):  sum1 += 1/2*s*(s+1)*(2*s+1)*np.exp(-energy(s, J*conv)/(kB*temp))  sum2 += (2*s+1)*np.exp(-energy(s, J*conv)/(kB*temp))  sum1 *= pref*A_h*1e6  shift = sum1/sum2  return shift  def T_model(param, temp, shift, result=False, T_long=None, name=None):    S = 5/2  par = param.valuesdict()  J = par['J']  A = [par['A_'+str(i)] for i in range(shift.shape[1])]  res = []  conshift = np.zeros((len(temp), shift.shape[1]))  for i in range(len(temp)):  for j in range(shift.shape[1]):  conshift_s = calc_shift(temp[i], S, S, A[j], J)  conshift[i,j] = conshift_s  res.append(conshift_s-shift[i,j])  if result:  cont_list = []  for j in range(shift.shape[1]):  cont_list.append([calc_shift(t, S, S, A[j], J) for t in T_long])  fig = plt.figure()  ax = fig.add_subplot(111)  fig.set_size_inches(8, 10)  for i in range(shift.shape[1]):  ax.plot(1000/T_long, cont_list[i], lw = 0.7, c='k')  for i in range(shift.shape[1]):  line, = ax.plot(1000/temp, shift[:,i], 'o', label=f'{i+1}: J = {J:.1f} '+r'cm$^{-1}$'+f'; A/h = {A[i]:.3f} MHz')  ax.plot(1000/temp, conshift[:,i], '--', c=line.get_color())  plt.legend()  plt.xlabel(r'1000/T (K$^{-1}$)', fontsize=13)  plt.ylabel('Contact shift (ppm)', fontsize=13)  ax.tick_params(axis='both', which='major', labelsize=13)  plt.savefig(name+'.png', dpi=600)  plt.close()  return conshift  else:  return res  J = 300 # cm^-1  A_h = np.ones(8)*0.8  dia_shift = np.array([26.8, 56.27, 29.13, 24.4, 59.8, 27.8, 58, 57.24])  param = lmfit.Parameters()  param.add('J', value=J, min=0, max=1000, vary=False)  [param.add('A_'+str(i), value=A_h[i], min=0, max=3, vary=True) for i in range(shift_tot.shape[1])]  minner = lmfit.Minimizer(T_model, param, fcn_args=(temp, shift_tot))  result = minner.minimize(method='leastsq', max_nfev=30000)  popt = result.params  T_model(popt, temp, shift_tot, result=True, T_long=np.arange(200, 20000, 10), name=dir_res+'/'+'J_'+str(J)) |

# S4. Reaction monitoring with ^19^F NMR

##

## S4.1 3D snippets of sample datasets

| 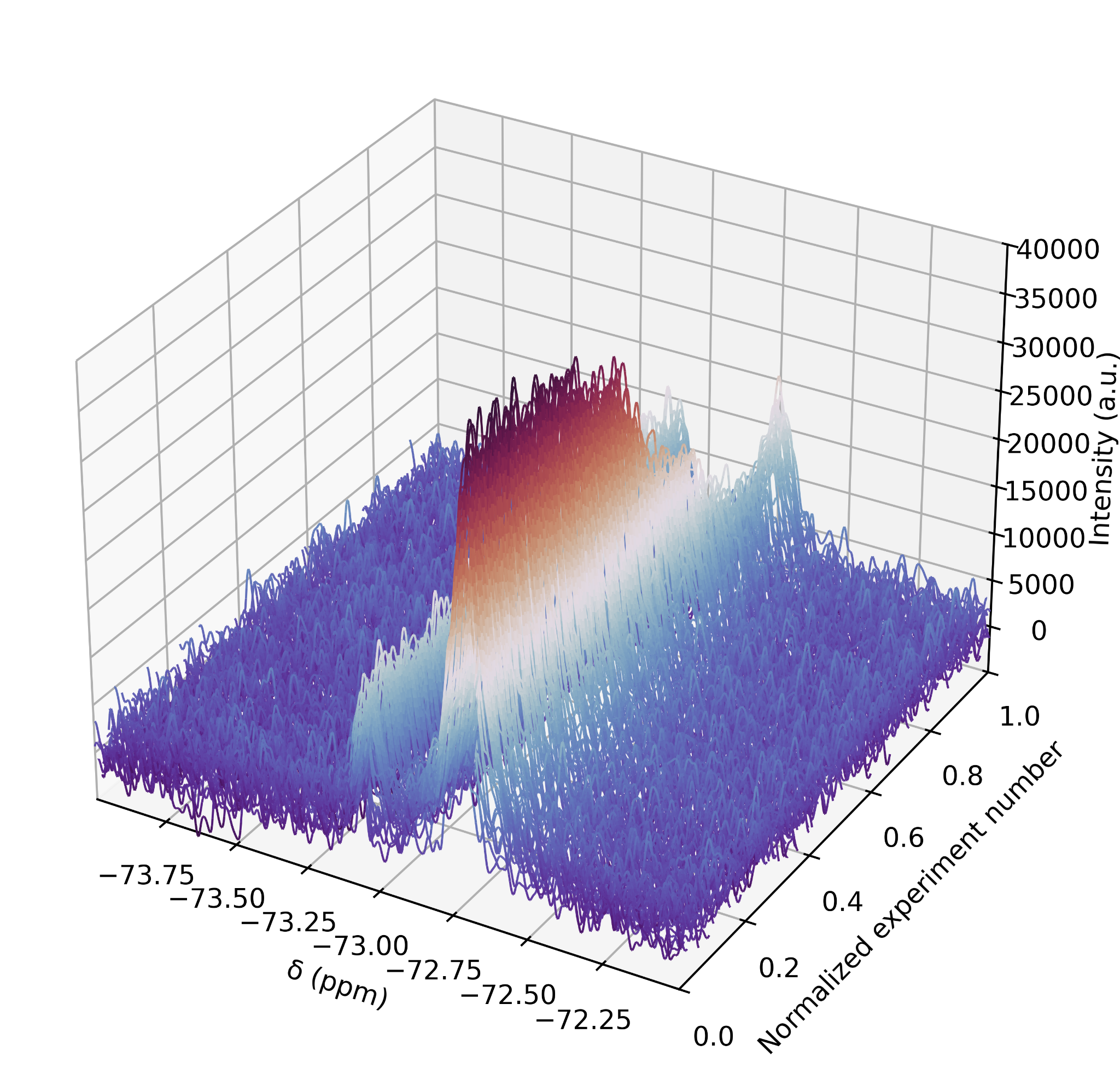 | 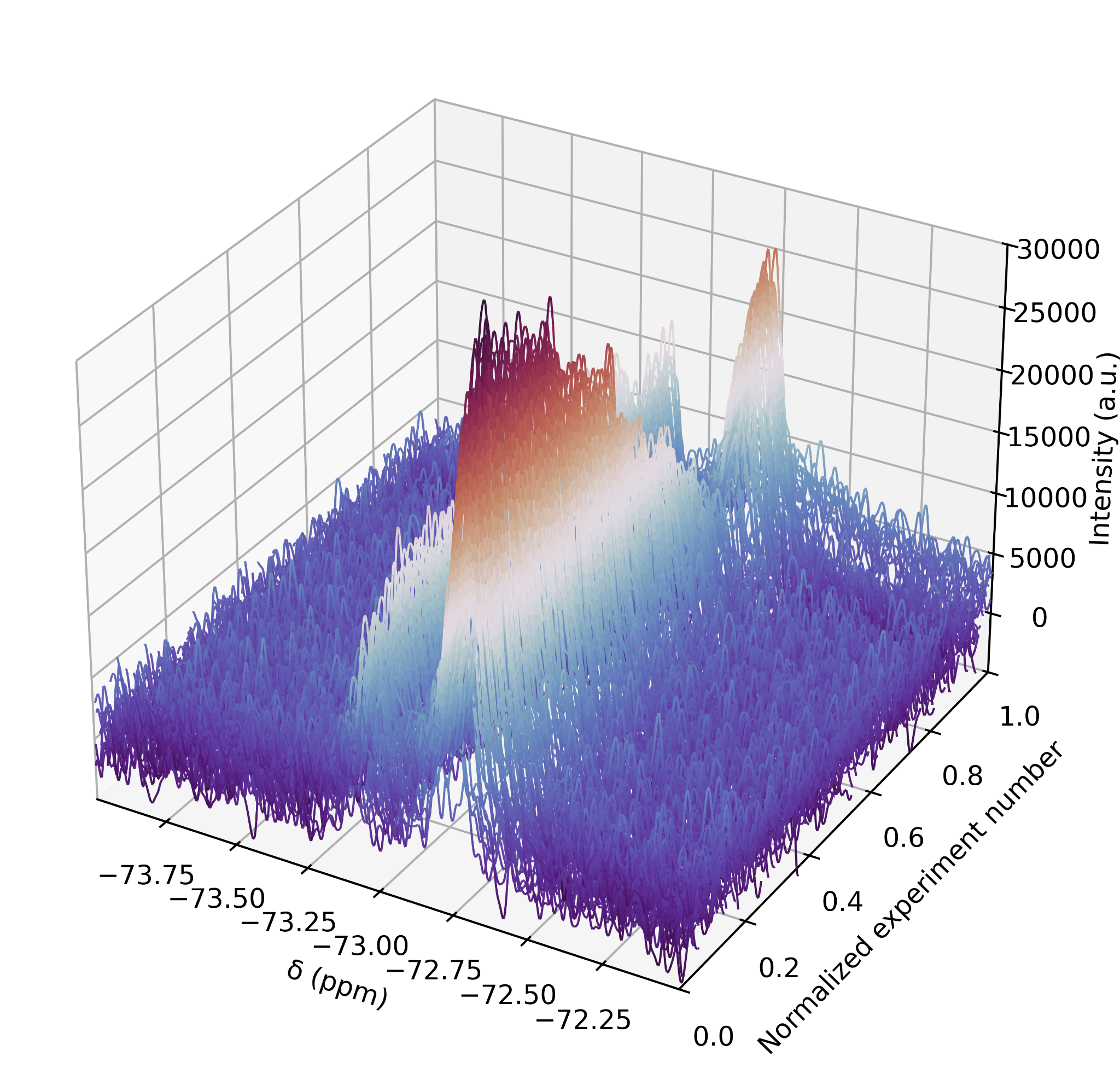 |
| --- | --- |
| 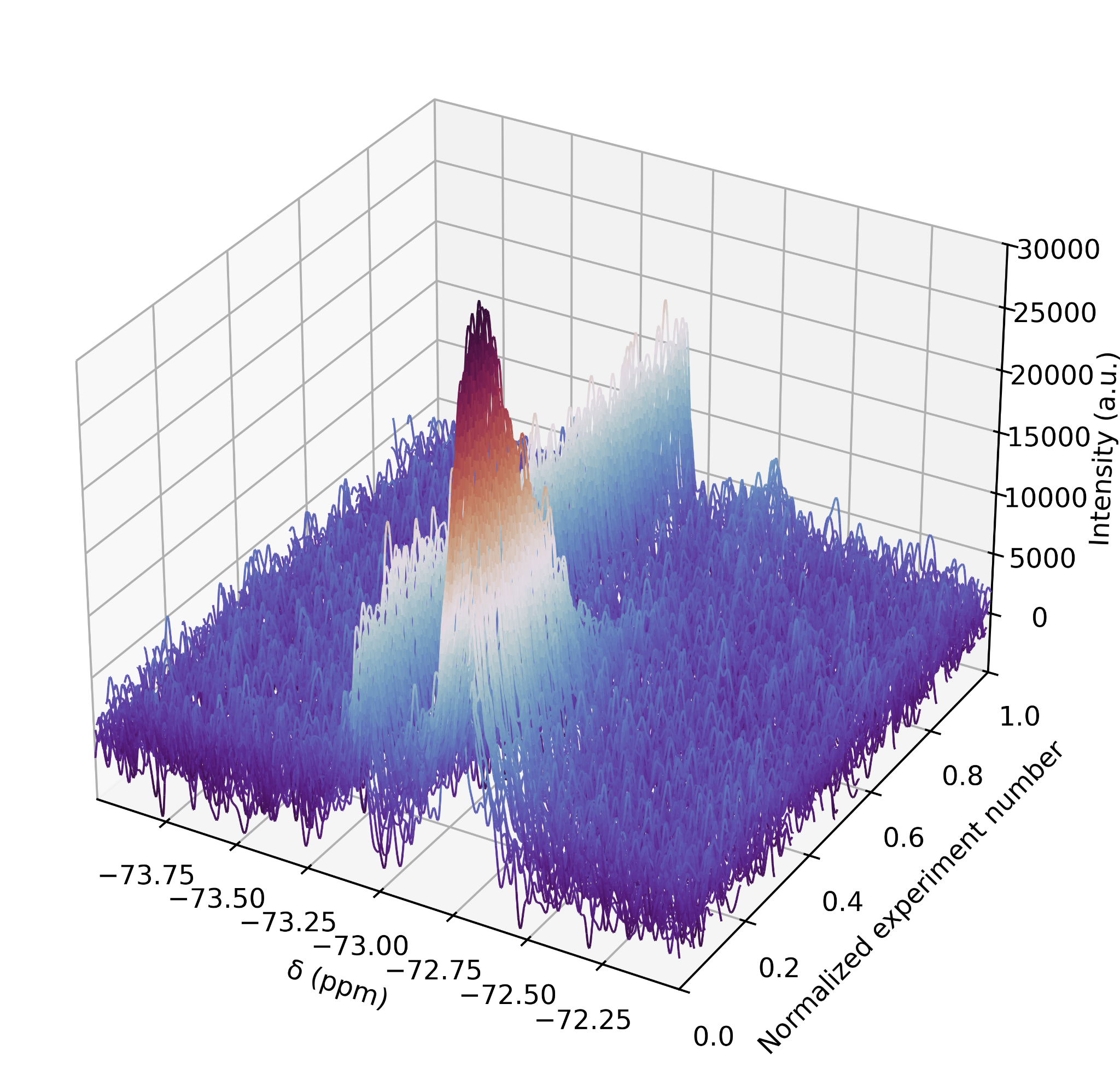 | 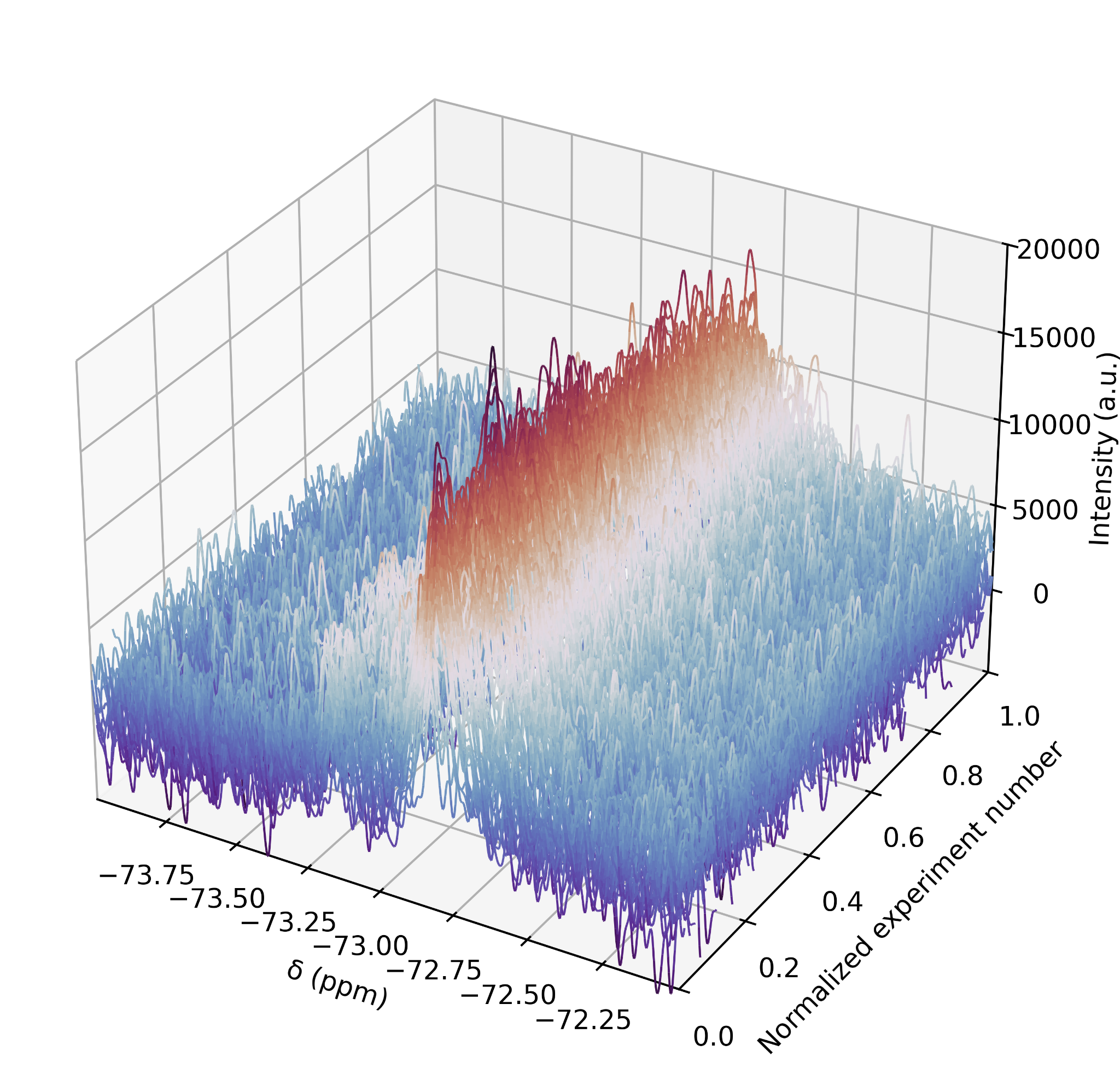 |

## S4.2 Integral plots via intensity_fit_1D

| Interval_1.png | Interval_2.png |
| --- | --- |
| 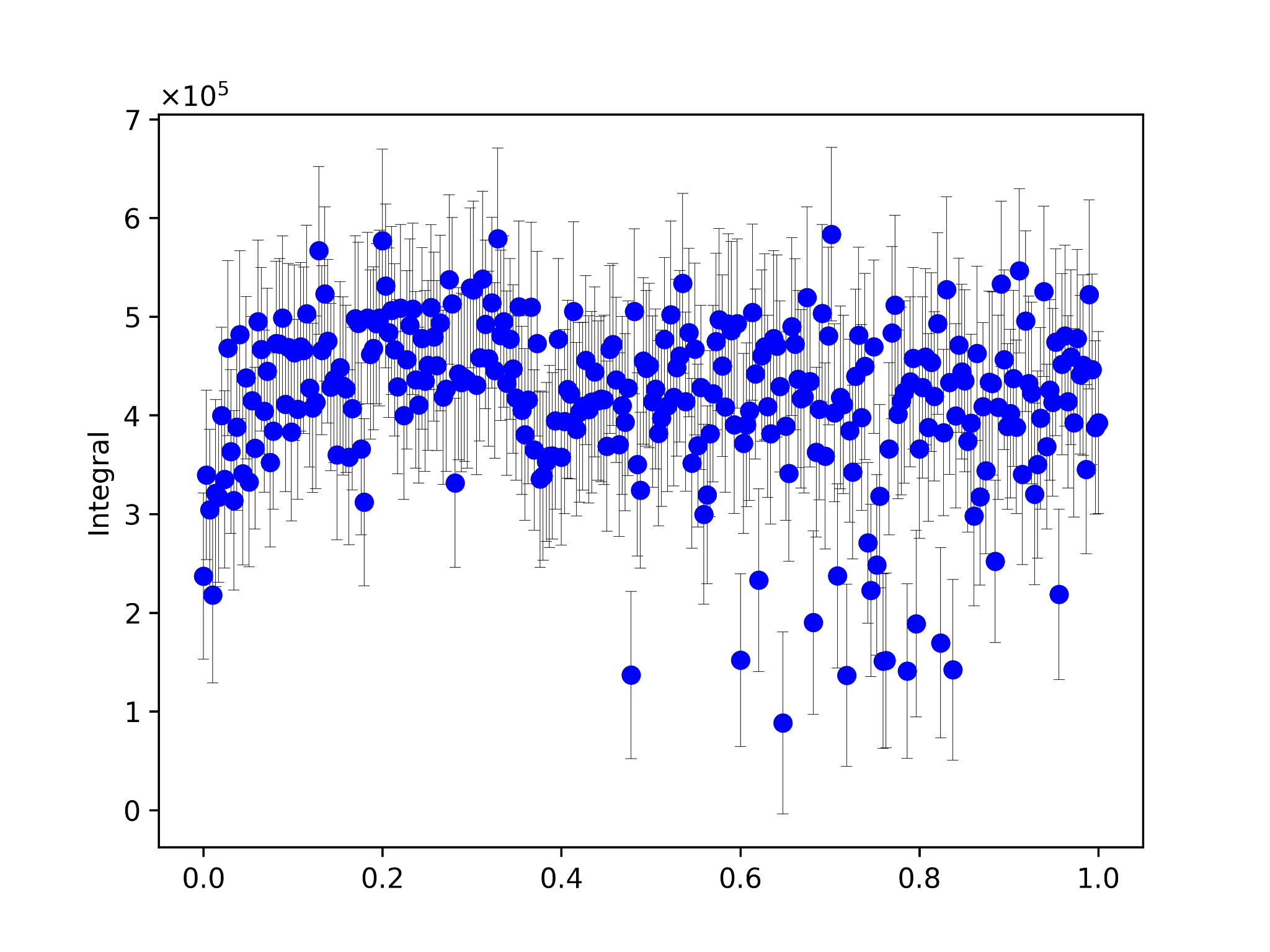 | 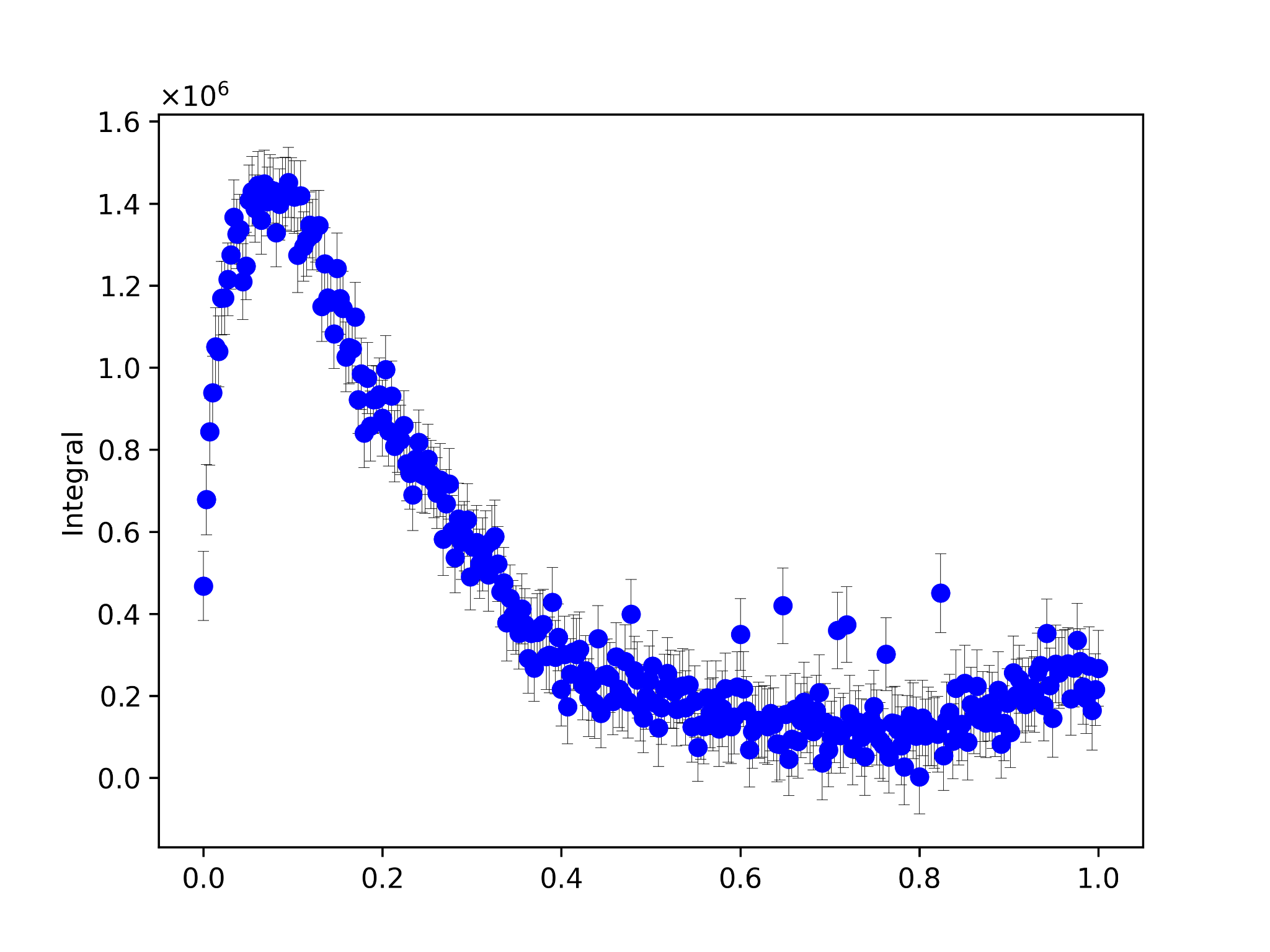 |
| 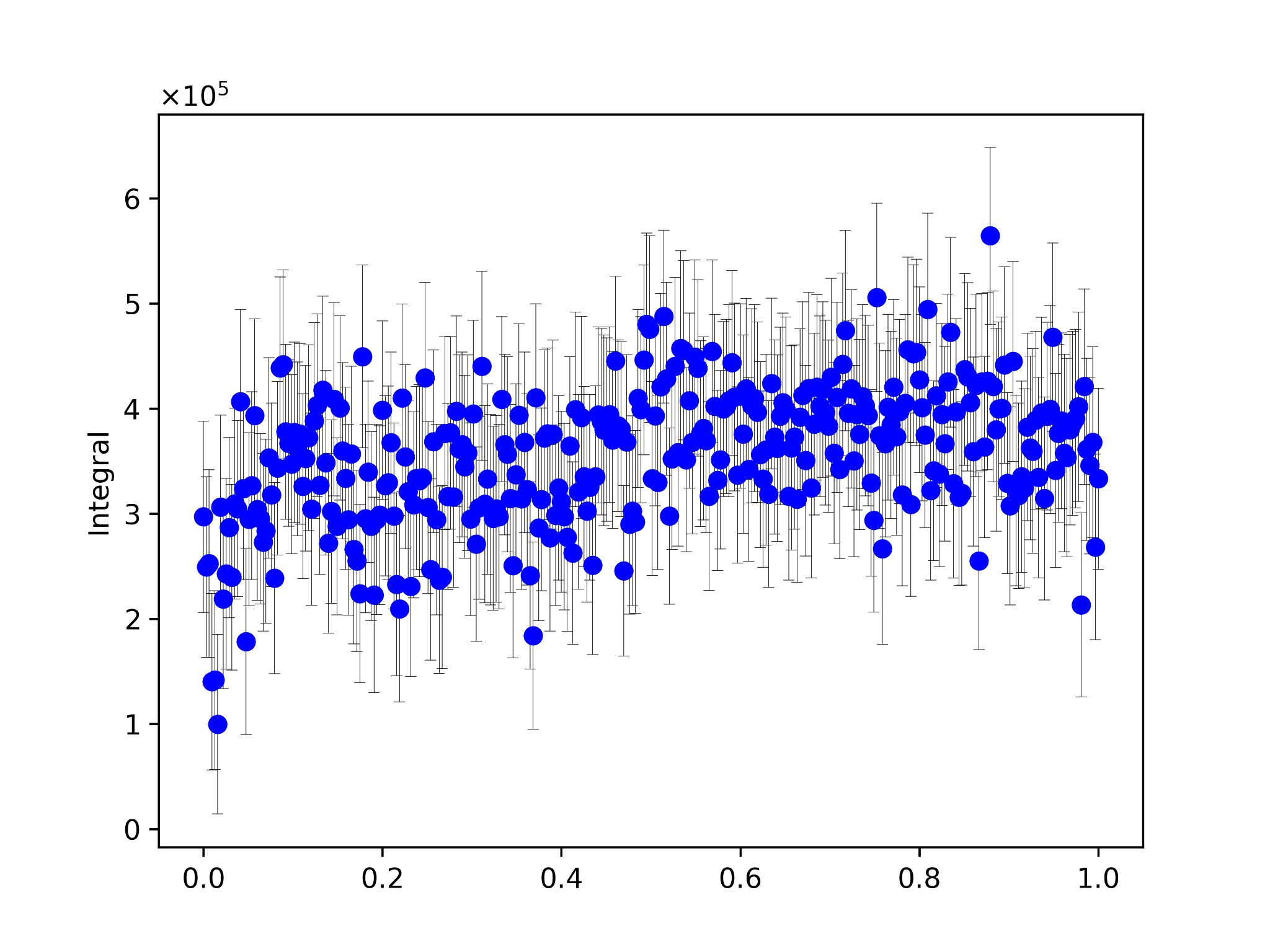 | 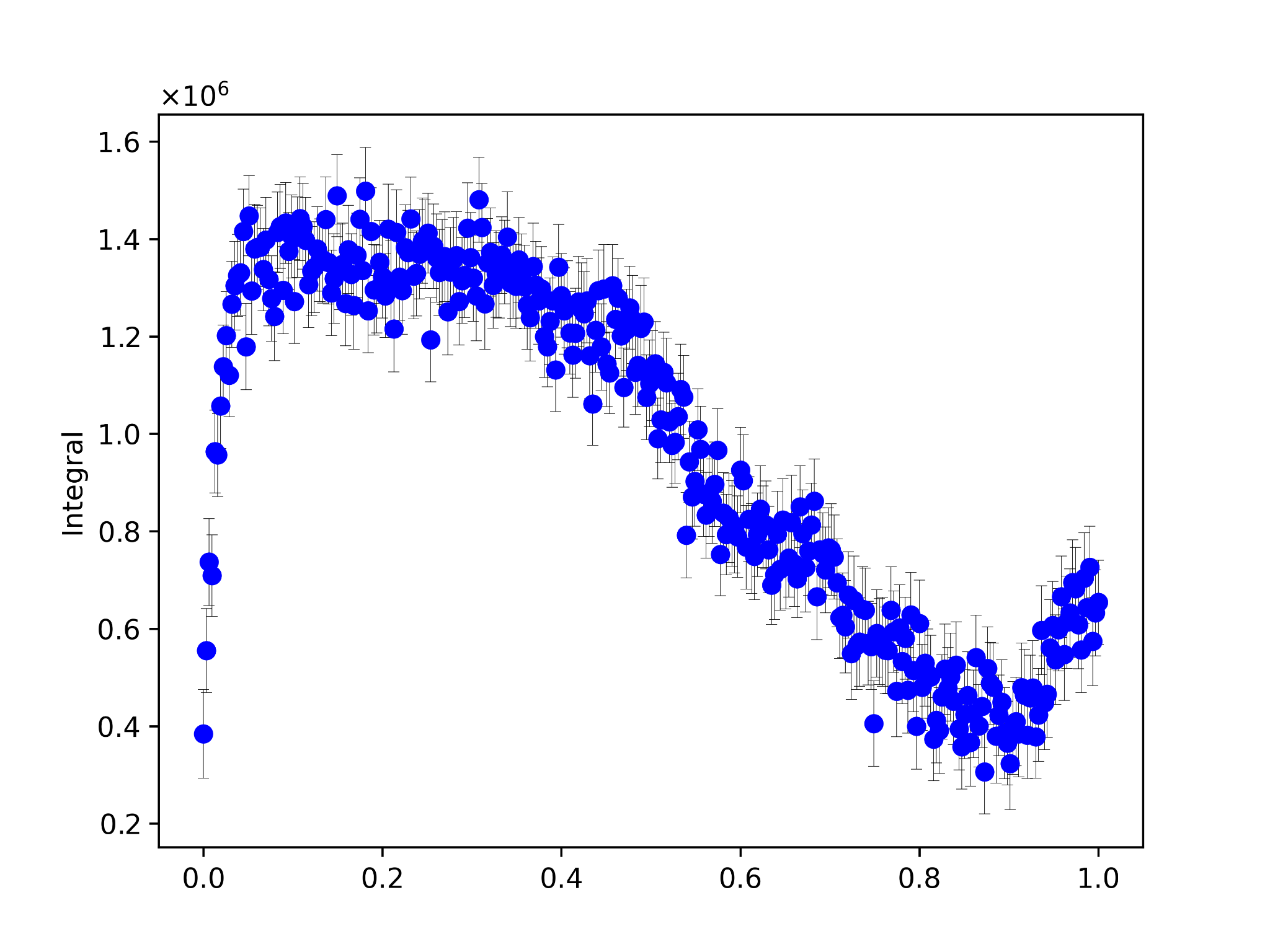 |
| 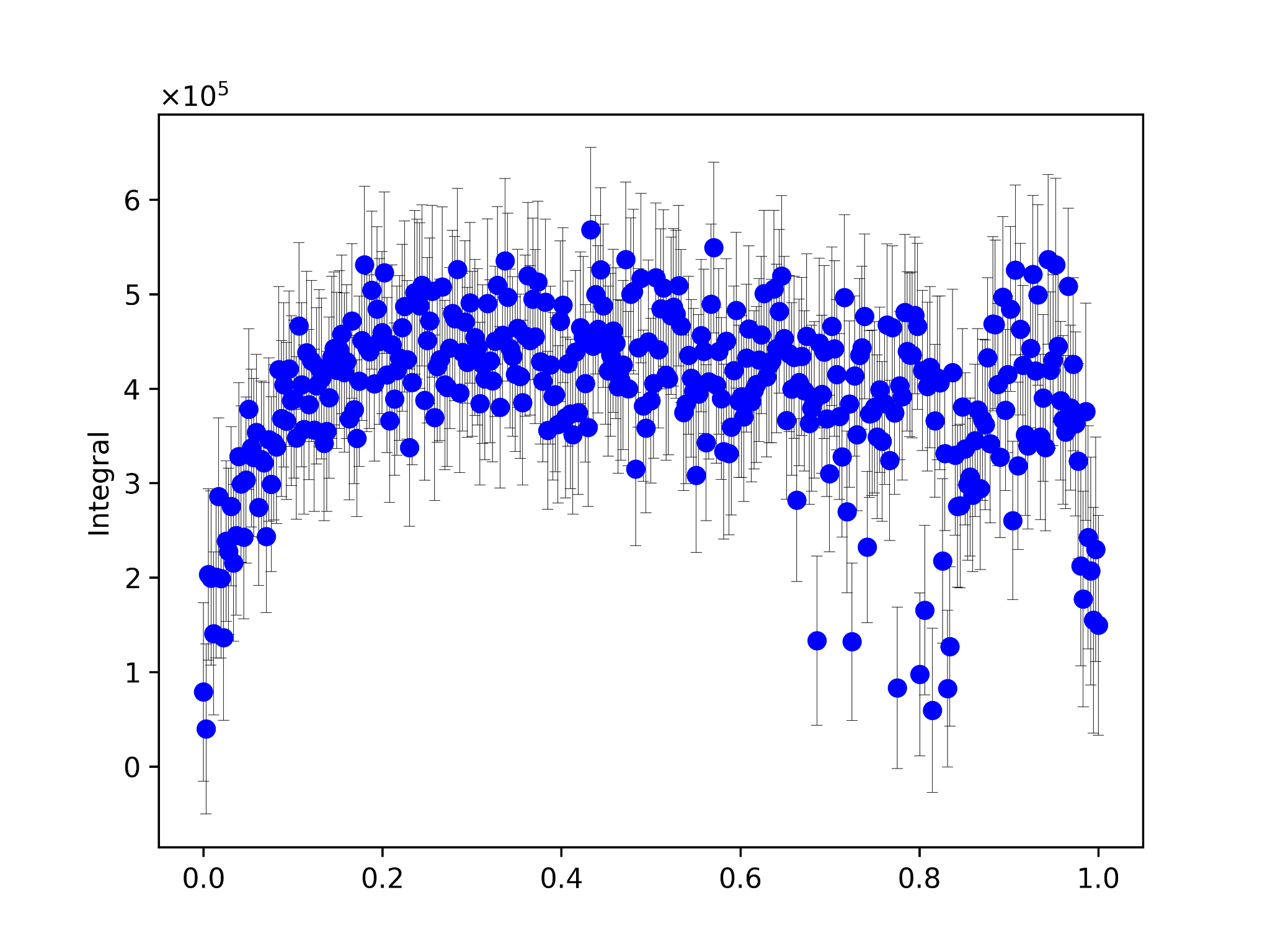 | 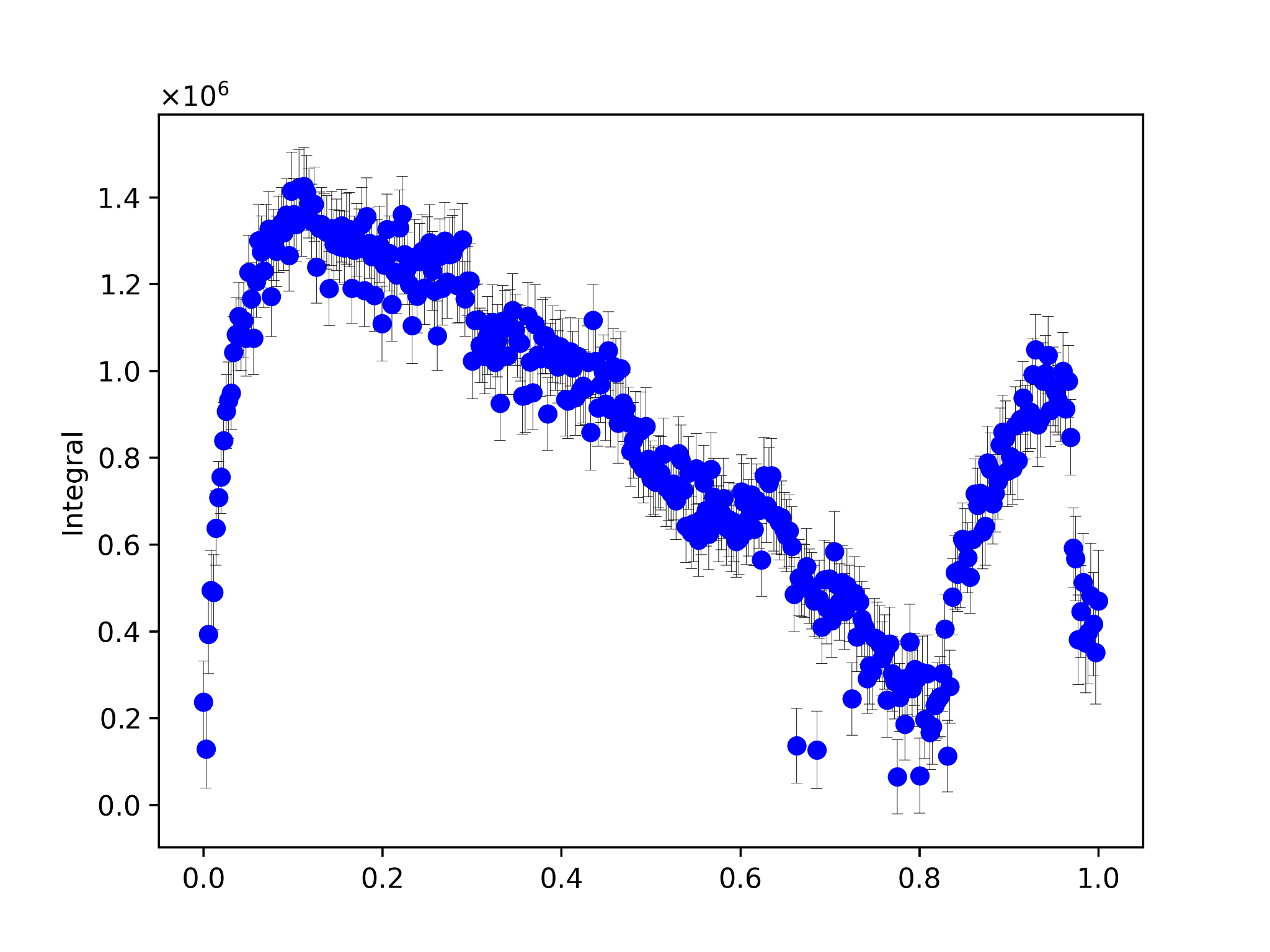 |
| 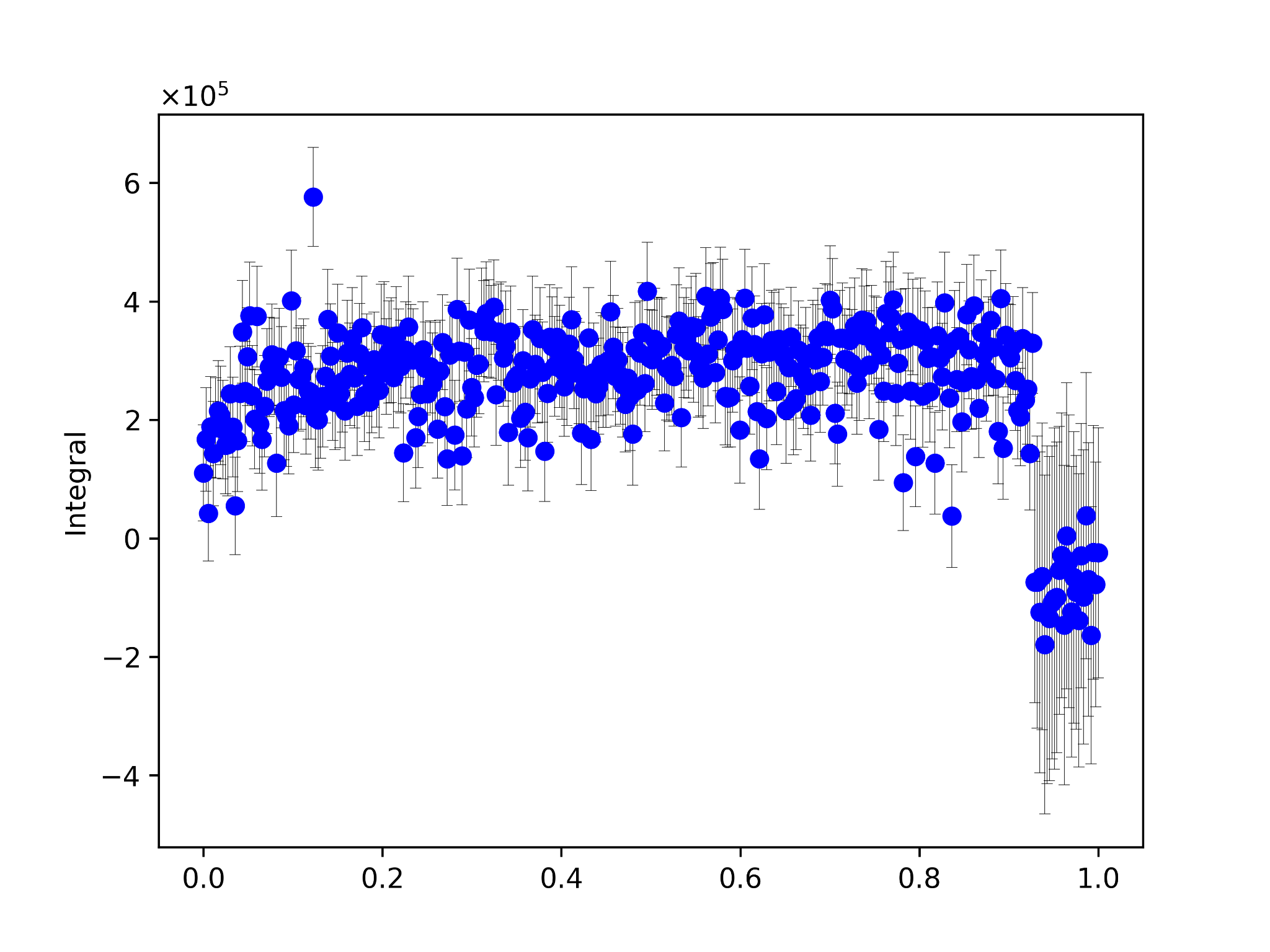 | 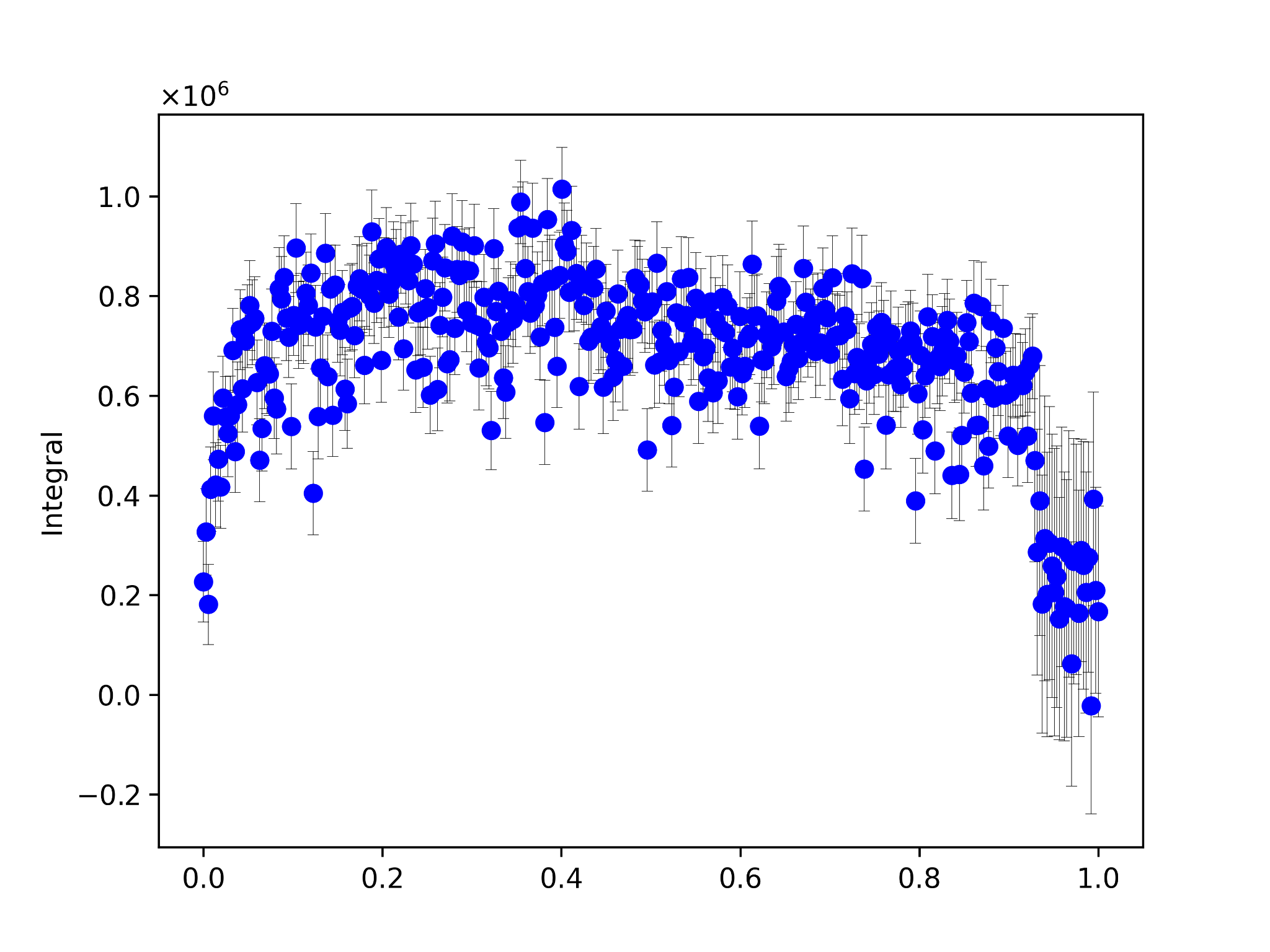 |

## S4.3 Integral plots via model_fit_1D

| Peak_1.png | Peak_2.png |
| --- | --- |
| 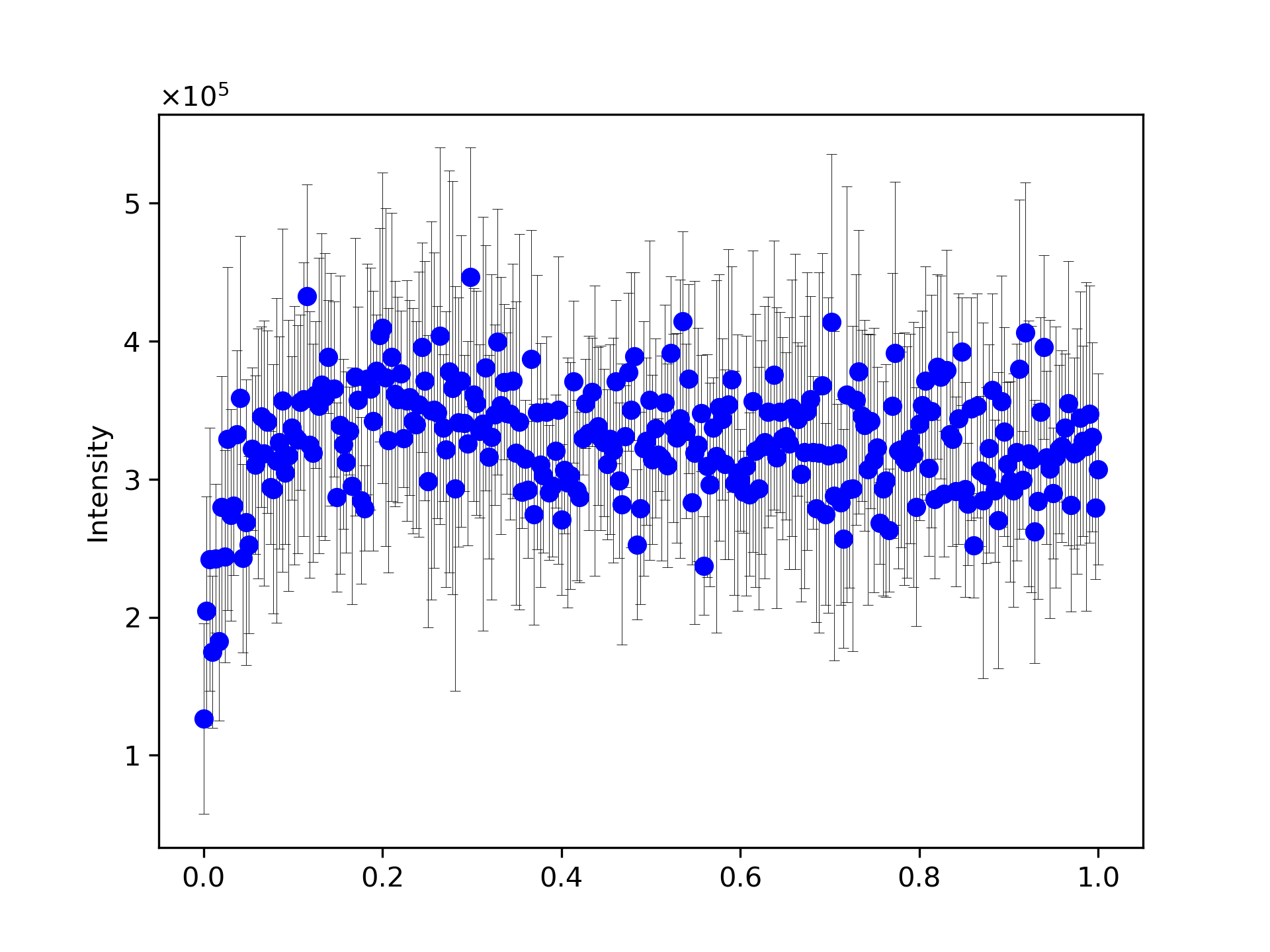 | 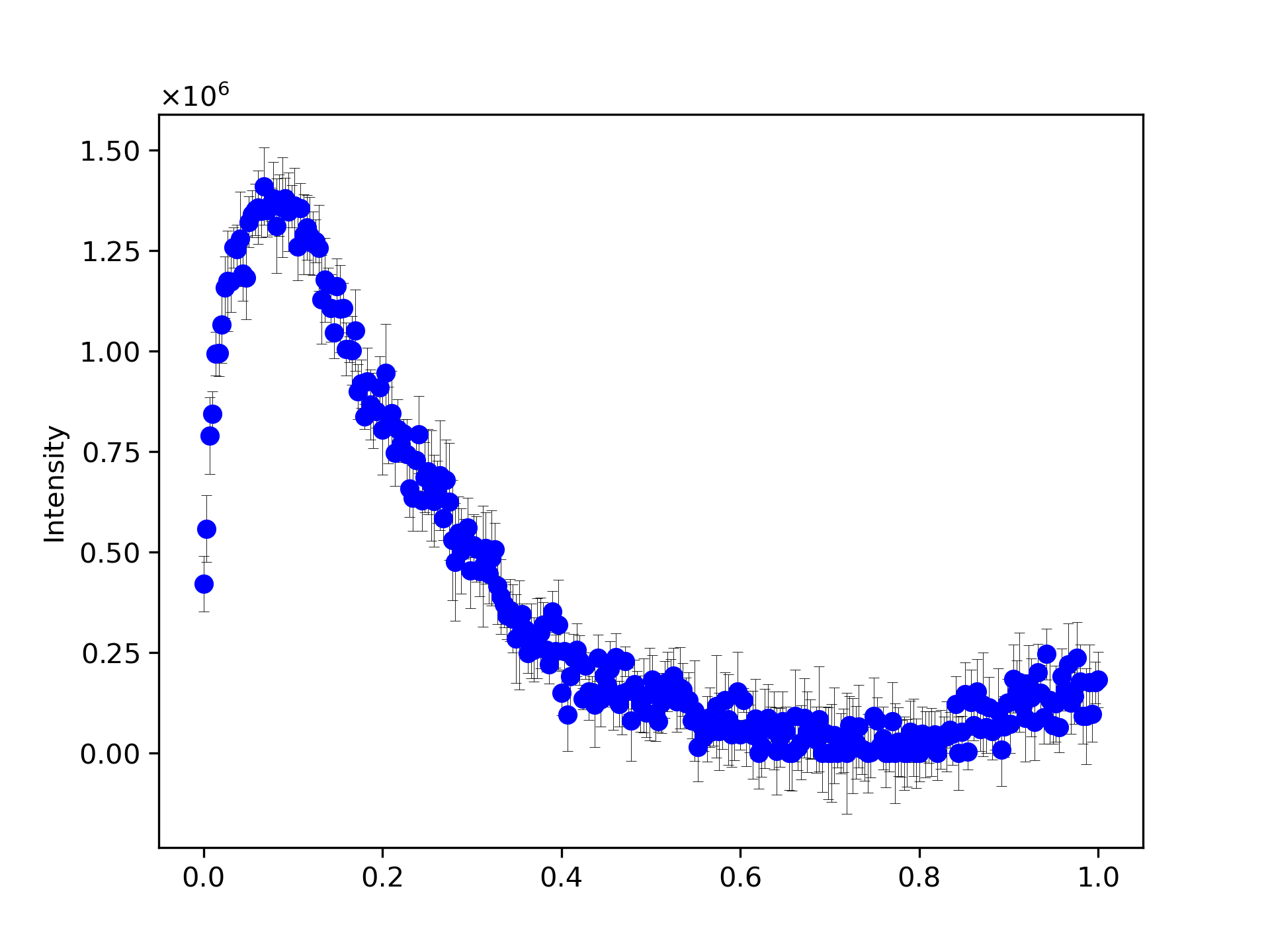 |
| 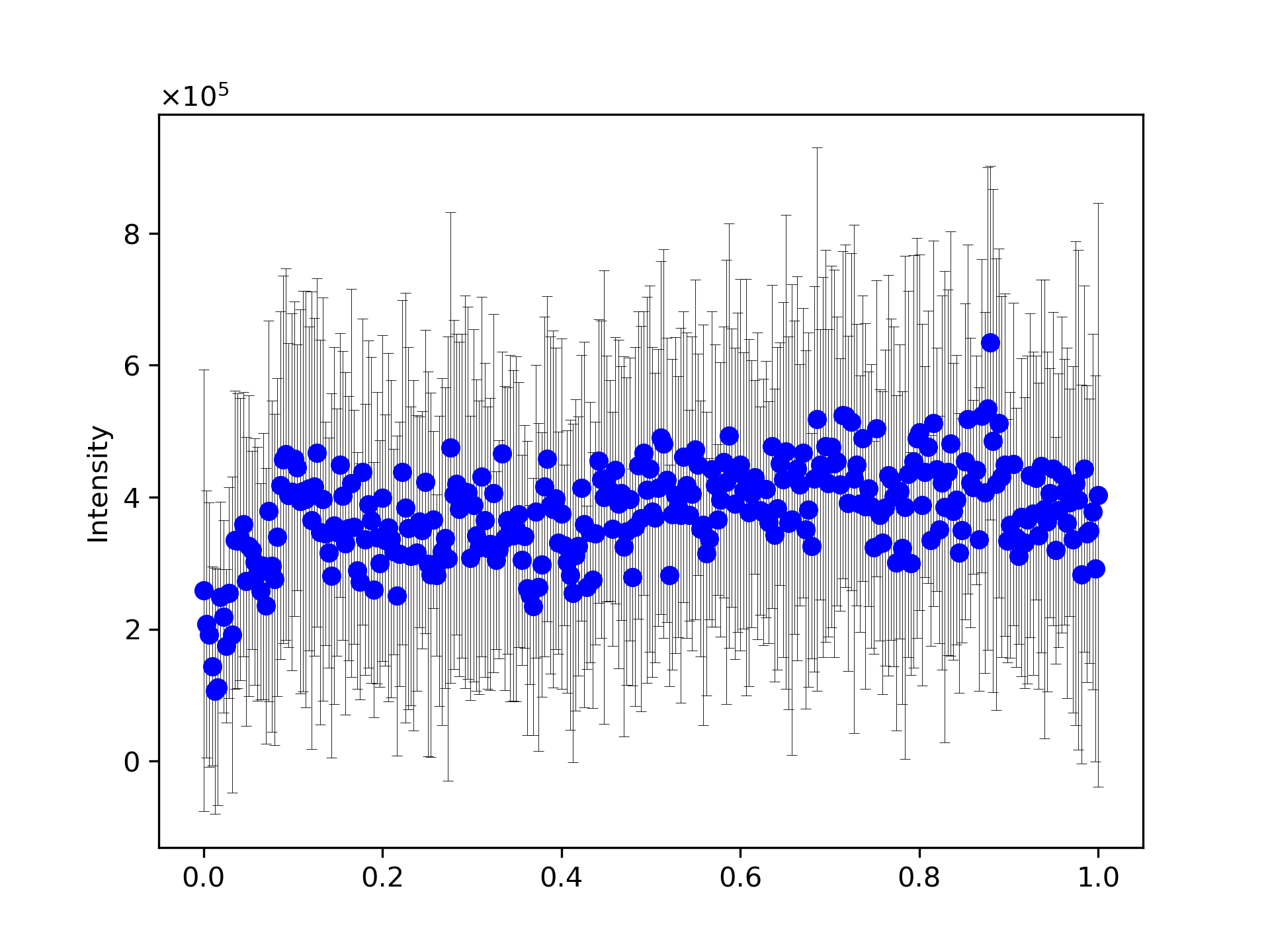 | 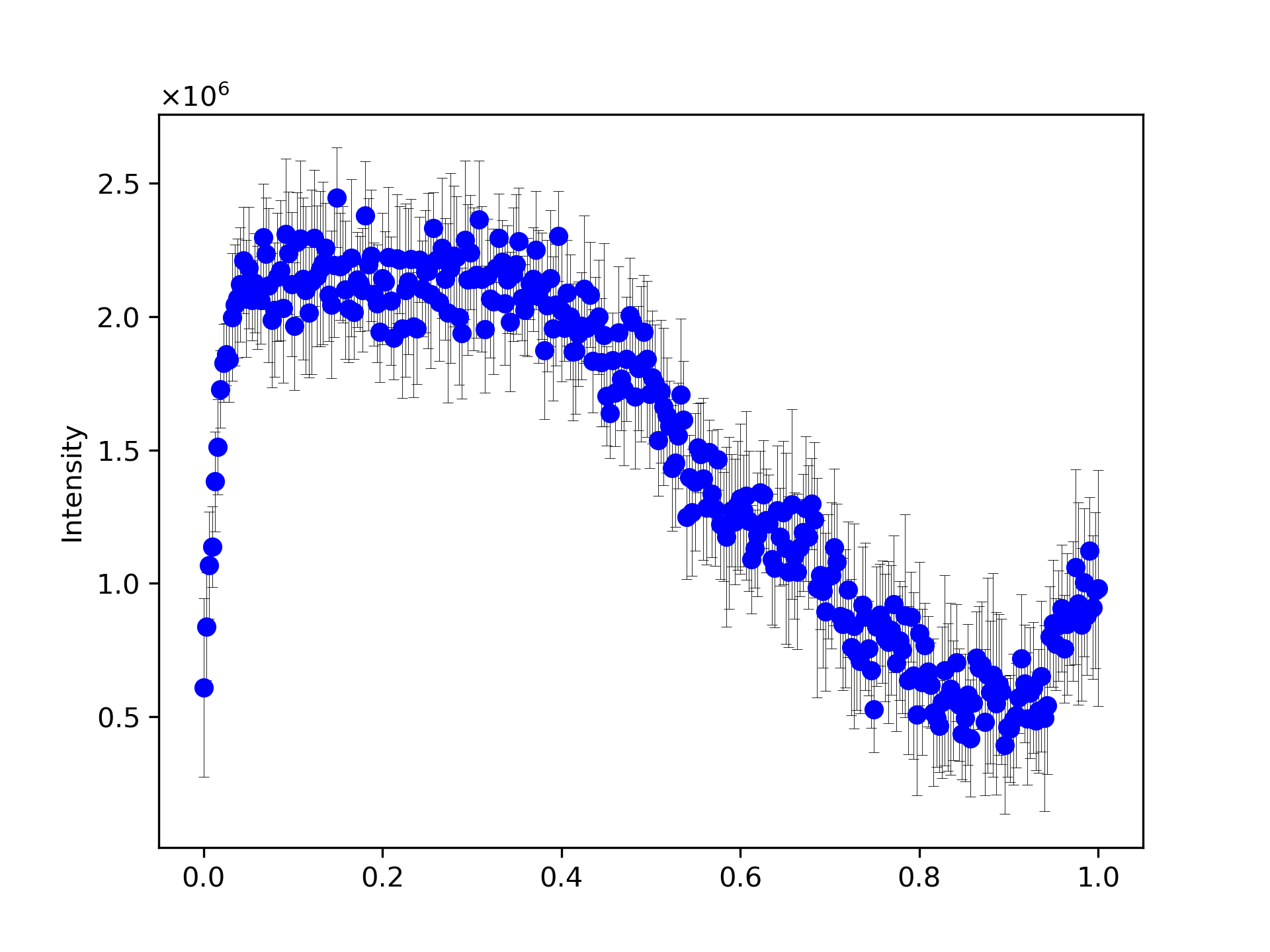 |
| 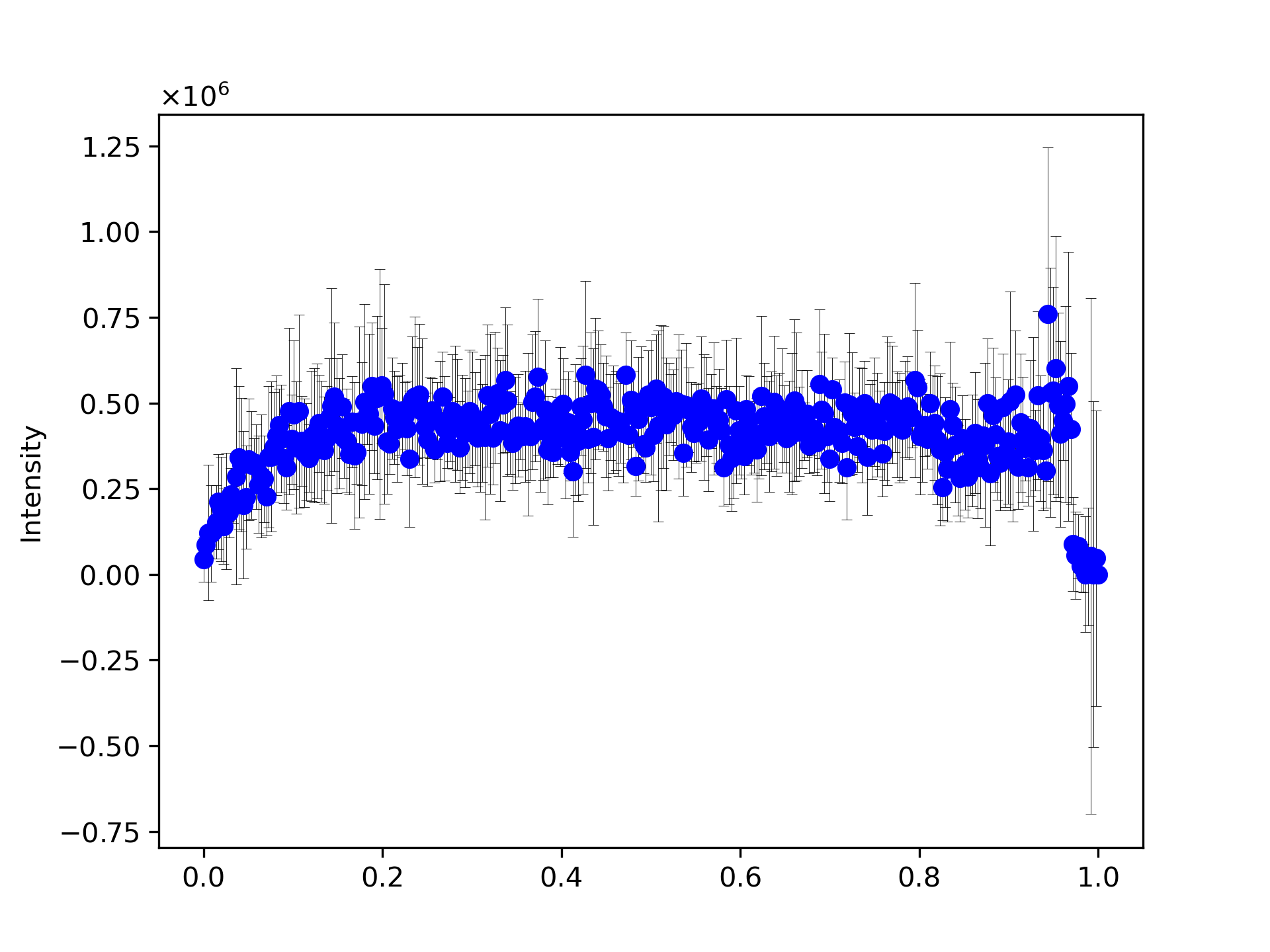 | 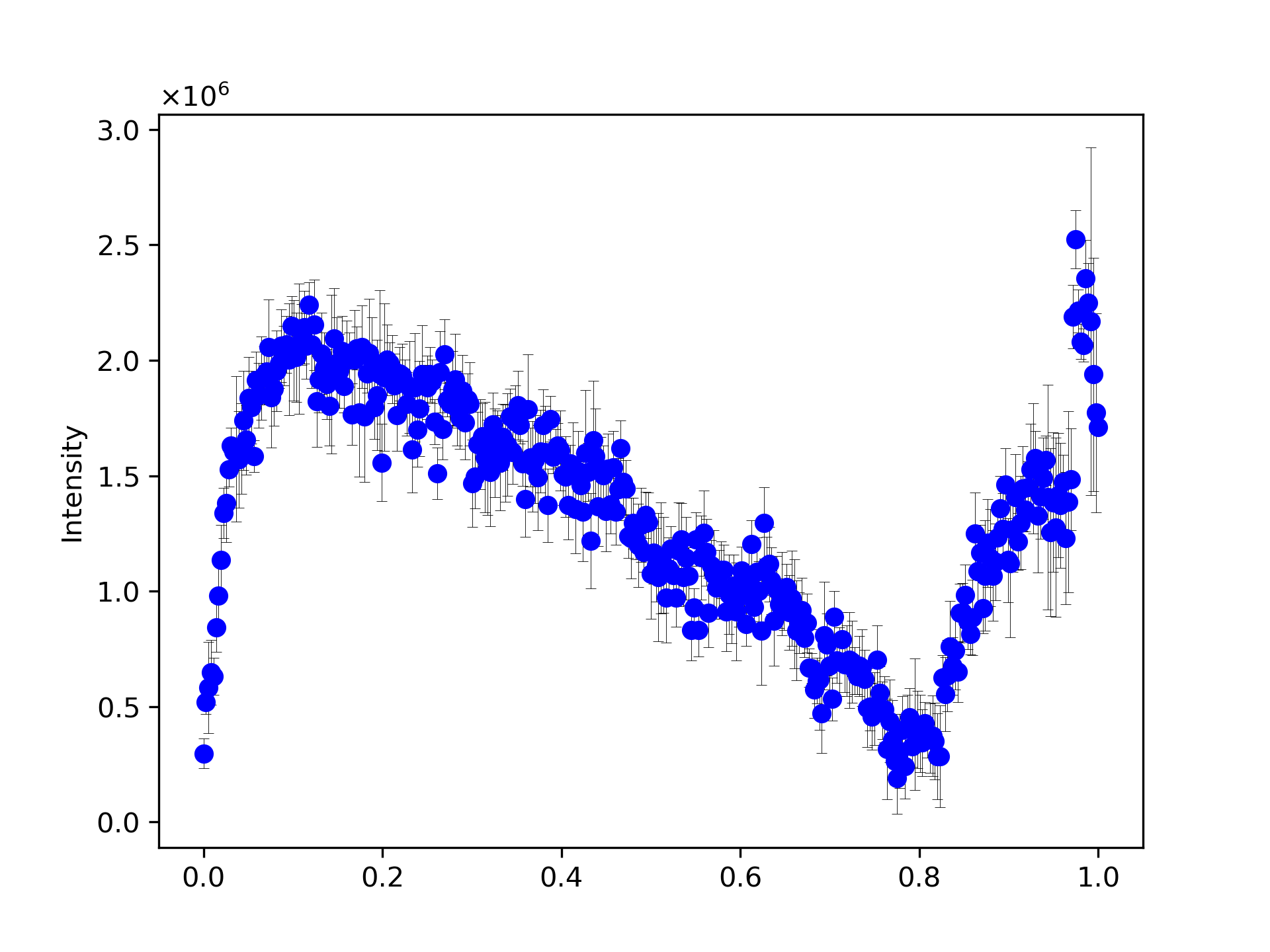 |
| 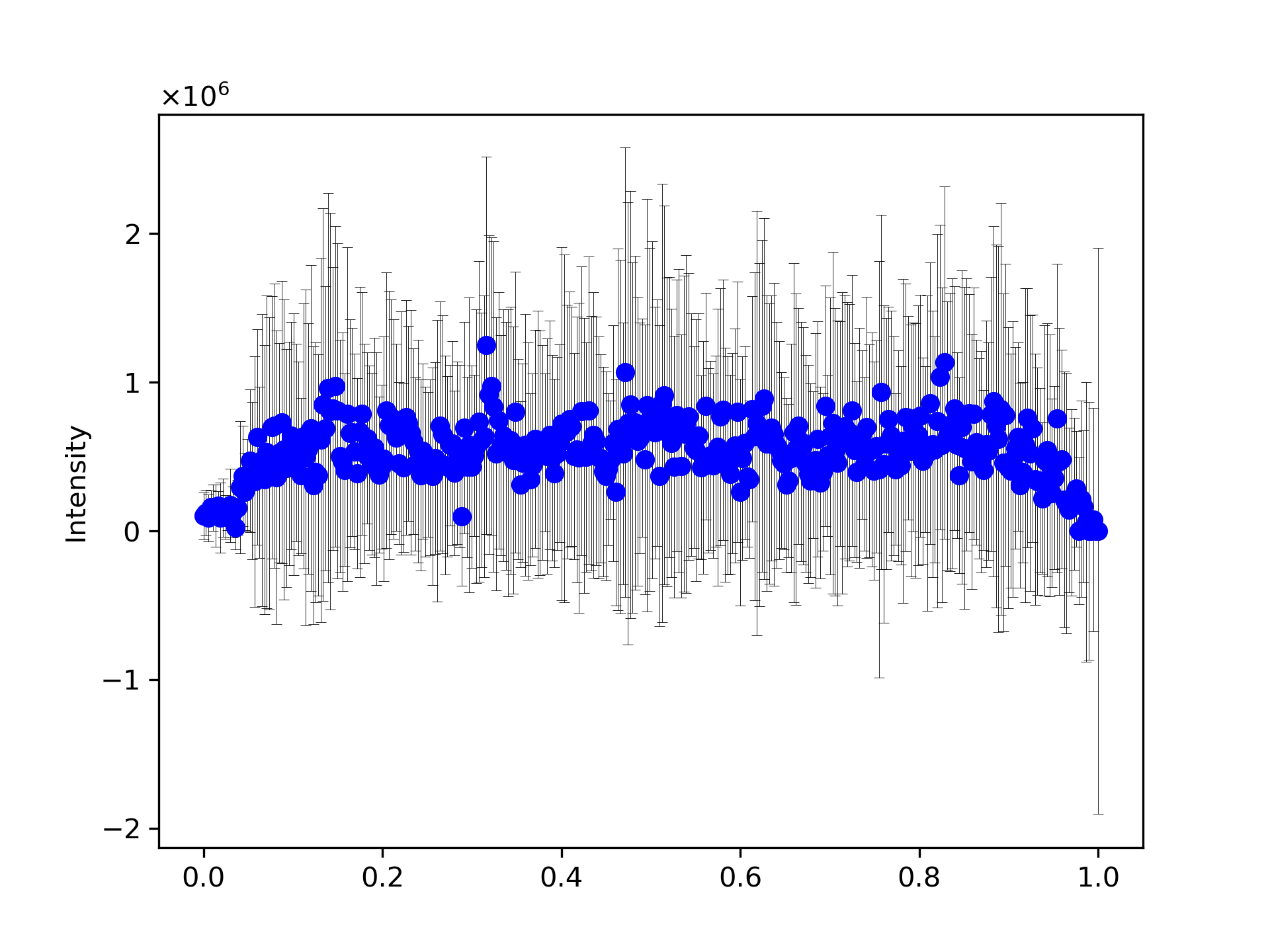 | 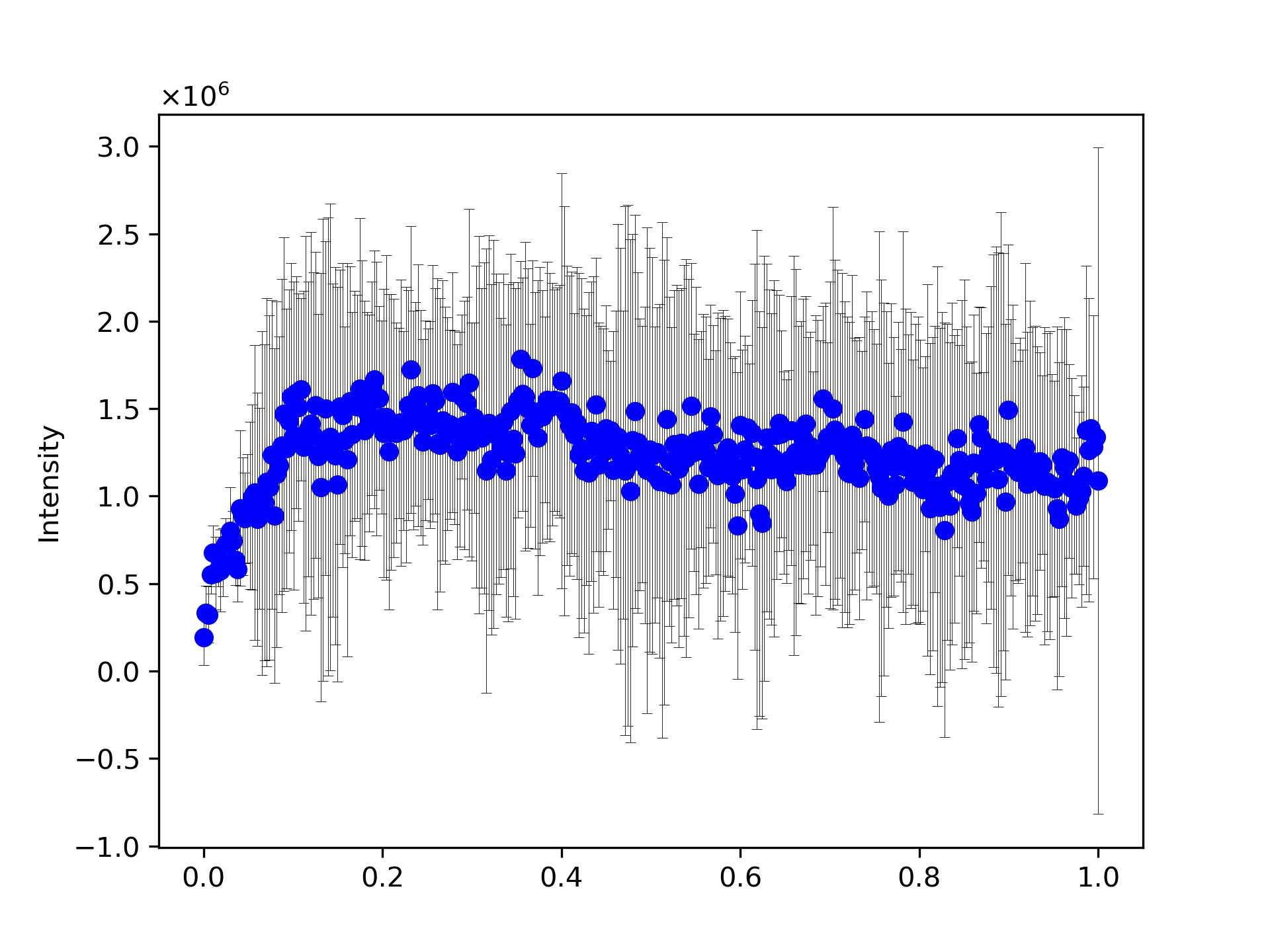 |

## S4.4 Code for plotting two integral trends simultaneously

| import matplotlib.pyplot as plt  import numpy as np  from f_fit import *  path = 'path/to/spectra/folder/'  num_sp = list(np.arange(101, 397, 1)) #the range of experiments that are being plotted, in our case we start from experiment 101 and end at 397  list_sp = [str(i) for i in num_sp]  #the following section reads the delays from the acqus file  t = []  for i in range(len(list_sp)):  acqus = open(path+list_sp[i]+"/acqus", "r").readlines()  for line in acqus:  if "##$DATE=" in line:  date = float(line.split("=")[1])  break  t.append(date)  count = 0  delays = [0] #initiating the list of delays which already contains the zero time point  for i in range(1,len(t)):  count += (t[i]-t[i-1])  delays.append(count)  delays = [i/3600 for i in delays] #converting the delays from seconds to hours  datax = delays #assigning the list of delays to the x-axis  datay = np.loadtxt("<folder>_modelfit/y_1.txt")[::-1]  dataz = np.loadtxt("<folder>_modelfit/y_2.txt")[::-1]  plt.plot(datax, datay, "g.") #plotting the first trend with green-coloured points  plt.plot(datax, dataz, "m.") #plotting the second trend with magenta-coloured points  plt.xlabel("Experimental time (h)", fontsize = "14")  plt.ylabel("Integral (a.u.)", fontsize = "14")  plt.ticklabel_format(style='sci', axis='y', scilimits=(0,0), useMathText = True)  plt.savefig("your_modelfit_integrals.png", transparent=False, dpi=600, format=None, metadata=None, bbox_inches=None, pad_inches=0.1, facecolor="auto", edgecolor="auto", backend=None) #saving the figure in .png format  plt.show()  exit() |
| --- |

Alternatively, the plot can be made by using simple experiment numbers on the x-axis.

| import matplotlib.pyplot as plt  import numpy as np  datax = np.arange(101, 397, 1).tolist() #the range of experiments that are being plotted, in our case we start from experiment 101 and end at 397  datay = np.loadtxt("<folder>_modelfit/y_1.txt")[::-1]  dataz = np.loadtxt("<folder>_modelfit/y_2.txt")[::-1]  plt.plot(datax, datay, "g.") #plotting the first trend with green-coloured points  plt.plot(datax, dataz, "m.") #plotting the second trend with magenta-coloured points  plt.xlabel("Experiment number", fontsize = "14")  plt.ylabel("Integral (a.u.)", fontsize = "14")  plt.ticklabel_format(style='sci', axis='y', scilimits=(0,0), useMathText = True)  plt.savefig("your_modelfit_integrals.png", transparent=False, dpi=600, format=None, metadata=None, bbox_inches=None, pad_inches=0.1, facecolor="auto", edgecolor="auto", backend=None) #saving the figure in .png format  plt.show()  exit() |
| --- |

## S4.5 Code for saving the hard model

The variable onecomp refers to a single component of the spectrum, while sumcomp represents the full spectrum with all the components included. Our example creates a 16k point array, this can be changed by the user when calling the zf function.

| sumcomp = None  if result:  onecomp = lor.copy()  onecomp *= em(onecomp, LB, SW)  onecomp *= qsin(onecomp, SSB)  onecomp = zf(onecomp, 16384)  onecomp = ft(onecomp, SI, dw, o1p, sf1)[0]  sumcomp = np.zeros_like(onecomp)  sumcomp += onecomp  np.save("your_16k_point_hard_model.npy", sumcomp) |
| --- |

# S5. Analysis of pseudo-2D relaxation measurements for olive oil sample

##

## S5.1. Result folder of intensity fit pseudo2D function

| Table S5.1.1: Output files in olive_oil_experiment_integral folder, their description and an example for the 1^st^ peak - (1.543 - 1.352 ppm). | |
| --- | --- |
| file name  [brief description] | example |
| 1.png 2.png  [Fitted plot of intensity value vs. delay with logarithmic x-axis. Obtained T_1_ value included] | 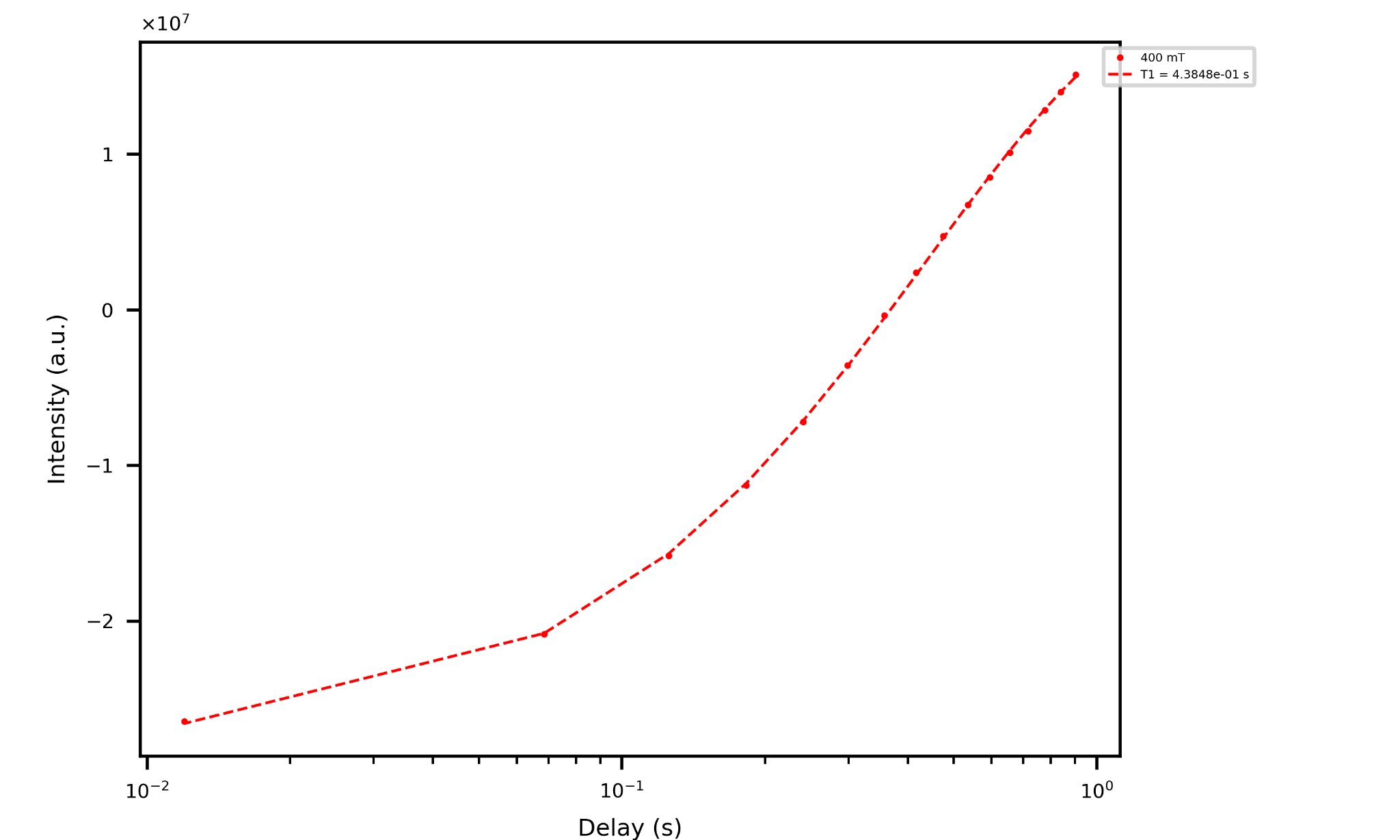 |
| Err_1  Err_2  [List of error values for obtained intensity points] | 1.06E+05  1.03E+05  1.06E+05  1.11E+05  1.04E+05  1.02E+05  1.04E+05  1.02E+05  1.03E+05  1.05E+05  1.05E+05  1.14E+05  1.16E+05  1.18E+05  1.26E+05  1.25E+05 |
| inp1_pseudo2D  [List of selected ranges for integration area] | 1.543e+00 1.352e+00  2.269e+00 2.090e+00 |
| pseudo2D_bsl_coeff  [List with values of coefficients for baseline correction, here no BC was done] | 0.00e+00 0.00e+00 0.00e+00 0.00e+00 0.00e+00  0.00e+00 0.00e+00 0.00e+00 0.00e+00 0.00e+00 |
| data_olive_oil_experiment_sp4  [output file with combined list of used parameters, the obtained fit parameters and their error analysis] | vide subsection S5.2 |
| Interval_1_sp1.png  Interval_2_sp1.png  [Fitted plot of integral value (with error) vs. delay with linear x-axis.] | 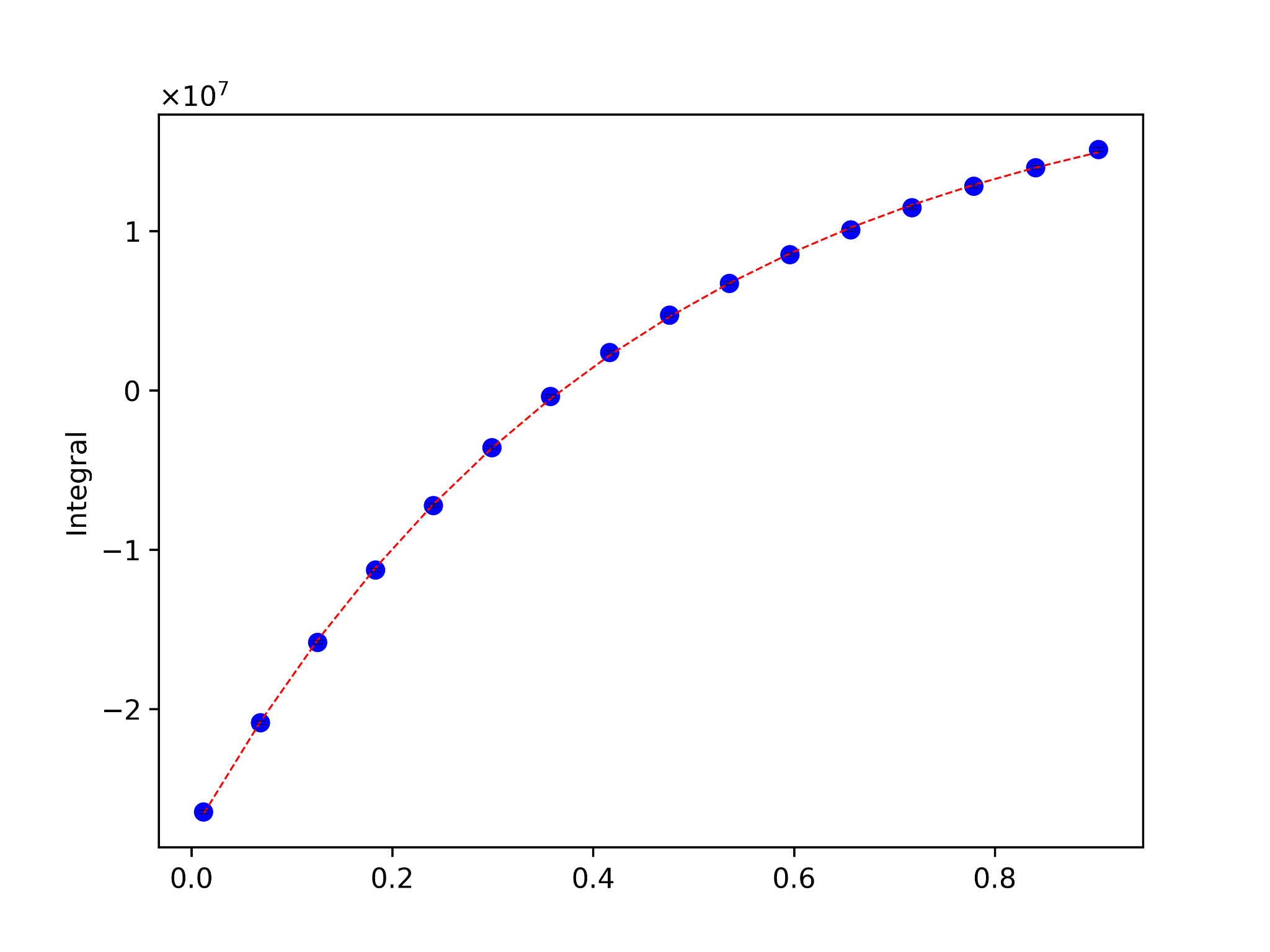 |
| Stack_I1.png  Stack_I2.png  [Plot with stacked view of the same integration area for different delays] | 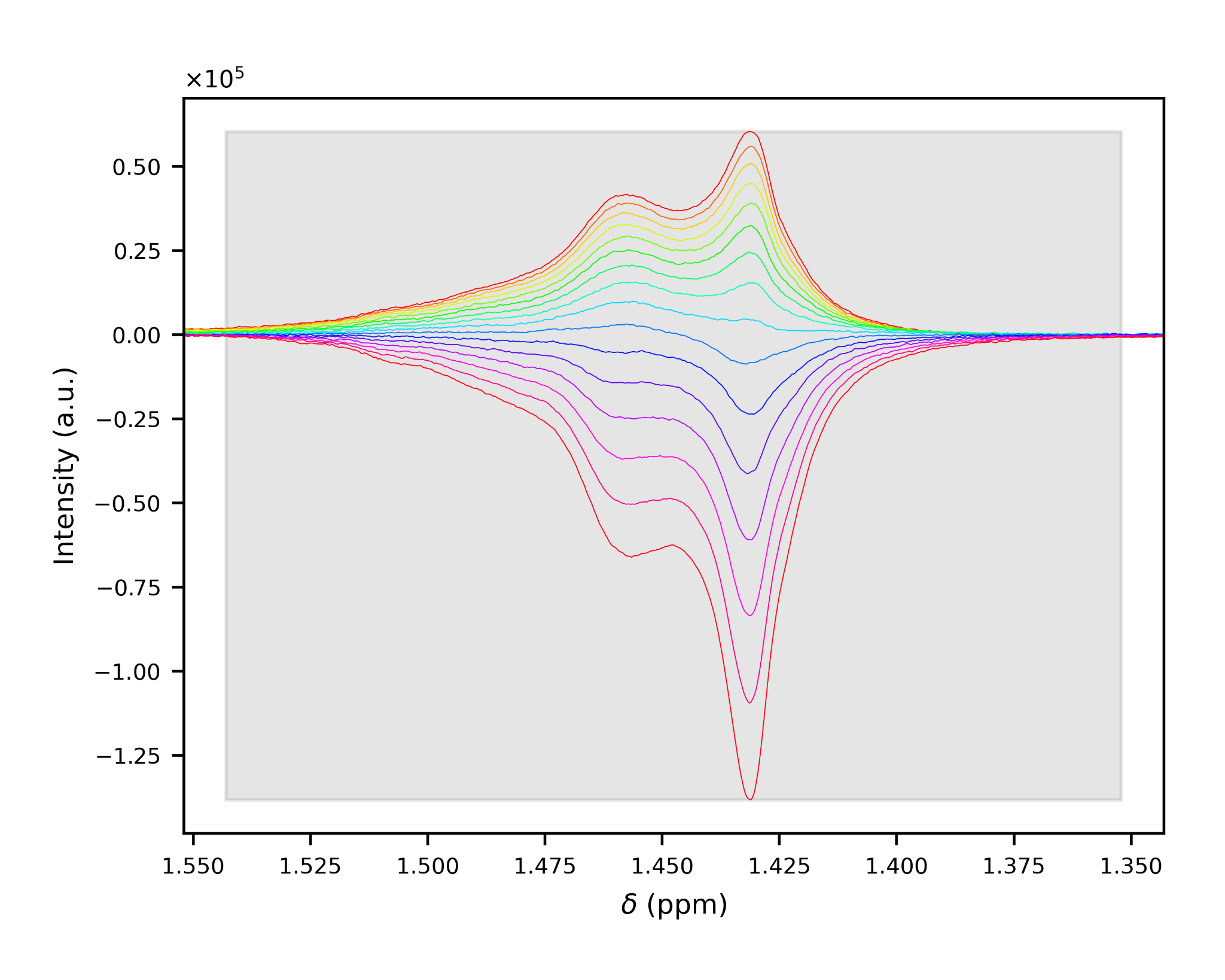 |
| t1  [output file with obtained T_1_ values and their error for each peak using mono- and biexponential fit, respectively. Biexponential function contains also fraction parameter f ] | \| n.peak \| T1 (s) \| err (s) \| f \|  \|  \| \| --- \| --- \| --- \| --- \| --- \| --- \| \| 1 \| 7.3E-01 \| 1.2E-01 \|  \|  \|  \| \| 1 \| 7.3E-01 \| 2.7E+04 \| 7.3E-01 \| 1.1E+04 \| 0.347 \| \| 2 \| 4.4E-01 \| 4.6E-03 \|  \|  \|  \| \| 2 \| 4.4E-01 \| 2.2E+05 \| 4.4E-01 \| 2.2E+05 \| 0.494 \| \| 3 \| 7.3E-01 \| 3.0E-01 \|  \|  \|  \| \| 3 \| 7.3E-01 \| 2.4E+04 \| 7.3E-01 \| 5.8E+04 \| 0.715 \| \| 4 \| 7.9E-01 \| 9.2E-02 \|  \|  \|  \| \| 4 \| 7.9E-01 \| 7.0E+04 \| 7.9E-01 \| 3.5E+04 \| 0.333 \| |
| x_1  X_2  [List of x-values, here the delays in seconds] | 0.90300  0.84067  0.77879  0.71734  0.65632  0.59573  0.53556  0.47580  0.41646  0.35751  0.29896  0.24081  0.18304  0.12565  0.06864  0.01200 |
| y_1  y_2  [List of y-values, here the intensity values for each delay] | 1.51E+07  1.40E+07  1.28E+07  1.15E+07  1.01E+07  8.53E+06  6.74E+06  4.73E+06  2.40E+06  -3.71E+05  -3.58E+06  -7.21E+06  -1.13E+07  -1.58E+07  -2.08E+07  -2.65E+07 |
| R1_1.csv  R1_2.csv  [.csv files containing the obtained R_1_ values and their error for each field] | 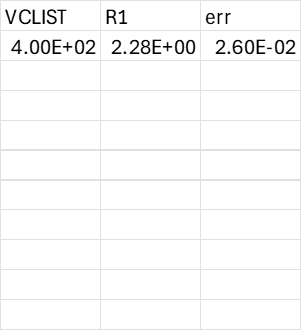 |
| R1_1.png  R1_2.png  [Plot for R_1_ values with error bar vs. magnetic field strength. In this case there is only one point since we are analyzing a single pseudo-2D] | 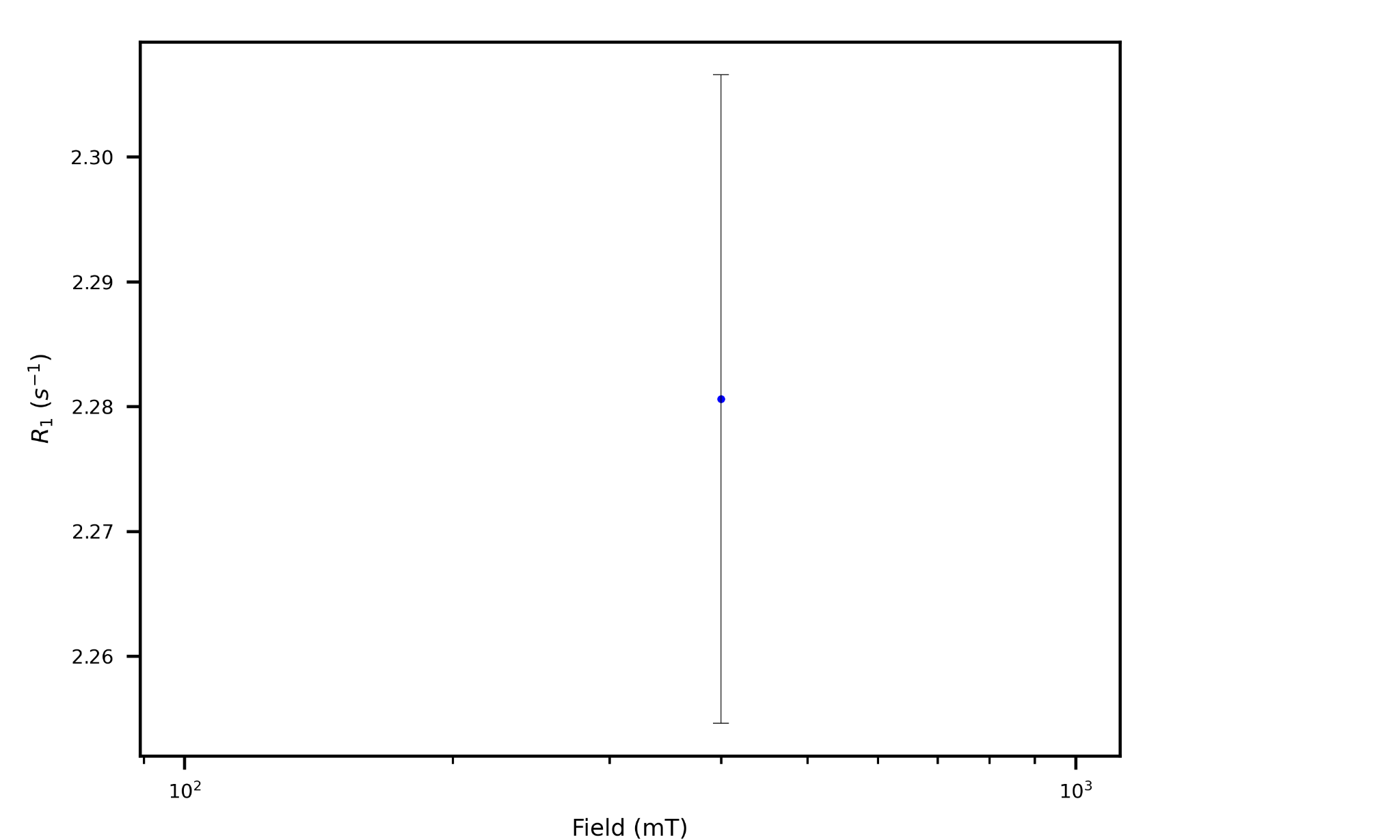 |

## S5.2. Output file for intensity_fit_pseudo2D - data_olive_oil_experiment_sp4

I/O INTERVALS: inp1_pseudo2D

SPECTRA PATH:

C:path/to/TRAGICO_analysis/olive_oil_experiment/1_Field/pdata/1

CALIBRATION: (2.50000:2.30000) ppm

in points

0 0 -1 -1 -2 -4 -5 -5 -9 -13 -8 -12 -12 -13 -14 -15

in ppm

0.00000 0.00000 -0.00019 -0.00019 -0.00038 -0.00076 -0.00095 -0.00095 -0.00171 -0.00247 -0.00152 -0.00228 -0.00228 -0.00247 -0.00266 -0.00285

Points:

1 0.903

2 0.841

3 0.779

4 0.717

5 0.656

6 0.596

7 0.536

8 0.476

9 0.416

10 0.358

11 0.299

12 0.241

13 0.183

14 0.126

15 0.069

16 0.012

VCLIST point: 1.00 T

Selected intervals (ppm):

1 1.5430 1.3520

2 2.2690 2.0900

====================================================================

N. interval: 1

N. point Integral Error

0 15124137.209 +/- 106019.621

N. interval: 2

N. point Integral Error

0 2990969.938 +/- 99261.398

------------------------------

N. PEAK: 1

------------------------------

Fit Parameters:

t1=-0.3580498917444226 A=24562177.147661746 a=-3345519.7260937165

------------------------------

N. PEAK: 2

------------------------------

Fit Parameters:

t1=-0.36946842974561367 A=4235692.914750983 a=-236842.65809241252

## S5.3. External function for intensity decay fit: fit_IR

| def fit_IR(x, y, name= None, err_bar=None, figura=True):  def IR_curve(t, t1):  return 1-2*np.exp(-t/t1)  def IR_residue(param, t, exp, result=False):  par = param.valuesdict()  t1 = 10**par['t1']  model = IR_curve(t, t1)  den = np.mean(model**2)-np.mean(model)**2  a = (np.mean(model**2)*np.mean(y)-np.mean(model*y)*np.mean(model))/den  A = (np.mean(model*y)-(np.mean(model)*np.mean(y)))/den  if not result:  return (a + A*model)-exp  else:  return a+A*model, A, a  param = lmfit.Parameters()  param.add('t1', value=0, min=-4, max=1)  minner = lmfit.Minimizer(IR_residue, param, fcn_args=(x, y))  result1 = minner.minimize(method='leastsq', max_nfev=10000, xtol=1e-8, ftol=1e-8)  popt1 = result1.params  report1 = lmfit.fit_report(result1)  func1, A, a = IR_residue(popt1, x, y, result=True)  popt1.add('A', value=A)  popt1.add('a', value=a)  #compute the mean squared deviation of the experimental points from the model function  RMSE1 = np.sqrt(np.mean((y-func1)**2))  fig=plt.figure()  fig.set_size_inches(3.59,2.56)  ax = fig.add_subplot(1,1,1)  ax.tick_params(labelsize=12)  ax.plot(x, y, 'o', c='k', markersize=4)  #ax.set_xscale('log')  if err_bar is not None:  ax.errorbar(x, y, yerr=err_bar, fmt='none', ecolor='k', elinewidth=0.2, capsize=2, capthick=0.2)  label_mod = ''  for key, values in popt1.items():  if key=='t1':  try:  label_mod+=f'{10**values.value:.5e}'+r' $\pm$ '+f'{np.abs(10**values.value*values.stderr/values.value):.5e}'  err1 = np.abs(10**values.value*values.stderr/values.value)  except:  label_mod+=f'{10**values.value:.5e}'+r' $\pm$ Nan'  err1 = None  ax.plot(x, func1, 'r--', lw=1, label='y = a + A (1 - 2 exp(-t/T1))\nfit: T1 = '+label_mod+' sec\n'+r'$\chi^2$ red: '+f'{result1.redchi:.5e}'+r', RMSE: '+f'{RMSE1:.5e}')  ax.set_xlabel('Delay (s)', fontsize=12)  ax.set_ylabel('Intensity (a.u.)', fontsize=12)  ax.ticklabel_format(axis='y', style='scientific', scilimits=(-3,3), useMathText=True)  ax.yaxis.get_offset_text().set_size(11)  ax.legend(fontsize=10)  plt.tight_layout()  if figura:  if name is None:  plt.show()  else:  plt.savefig(name+'.png', dpi=600)  plt.close()  return popt1.valuesdict(), func1, report1, err1, RMSE1 |
| --- |

## S5.4. Integral plots via intensity_fit_pseudo2D

| Peak 1 (1.352 - 1.543) | Peak 2 (1.352 - 1.543) |
| --- | --- |
| 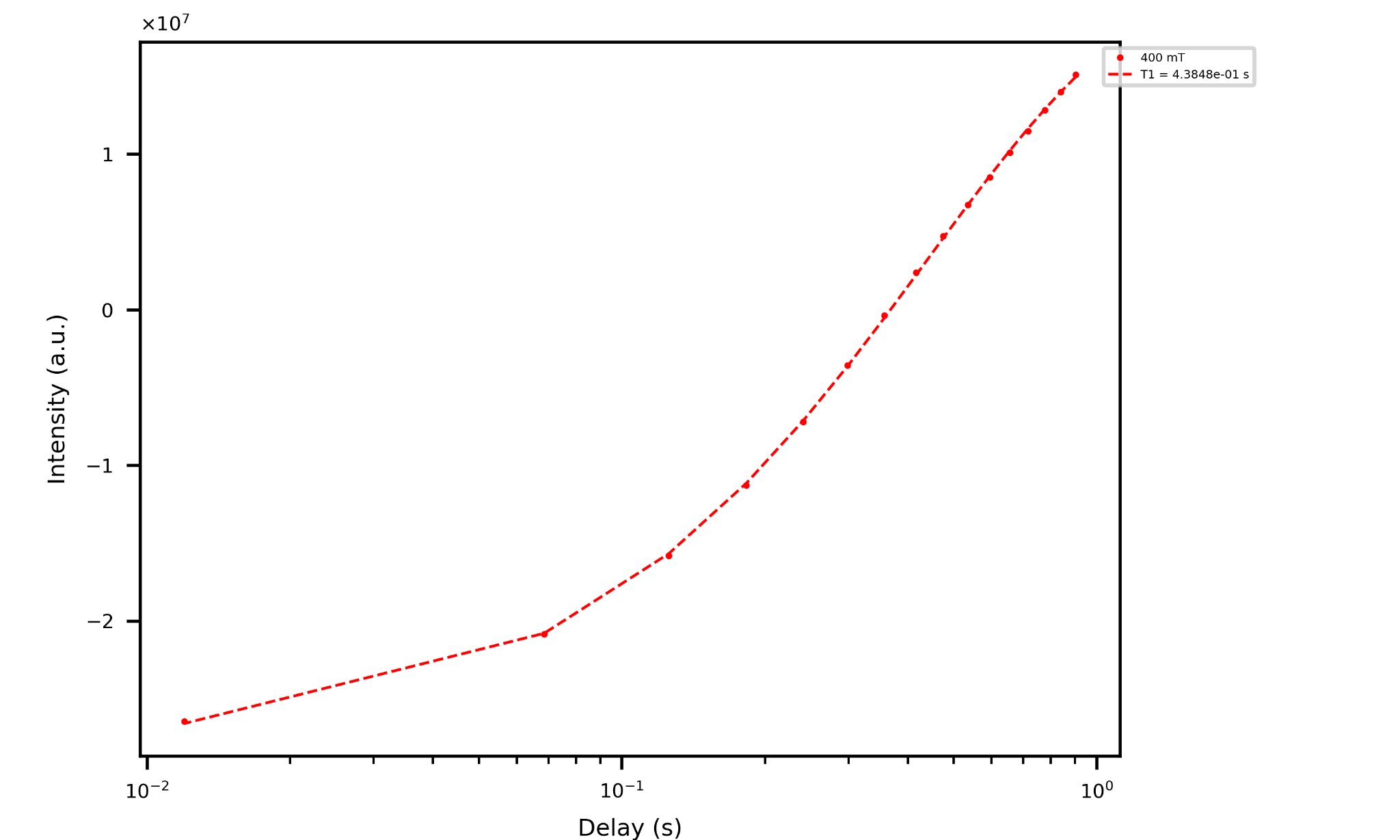 | 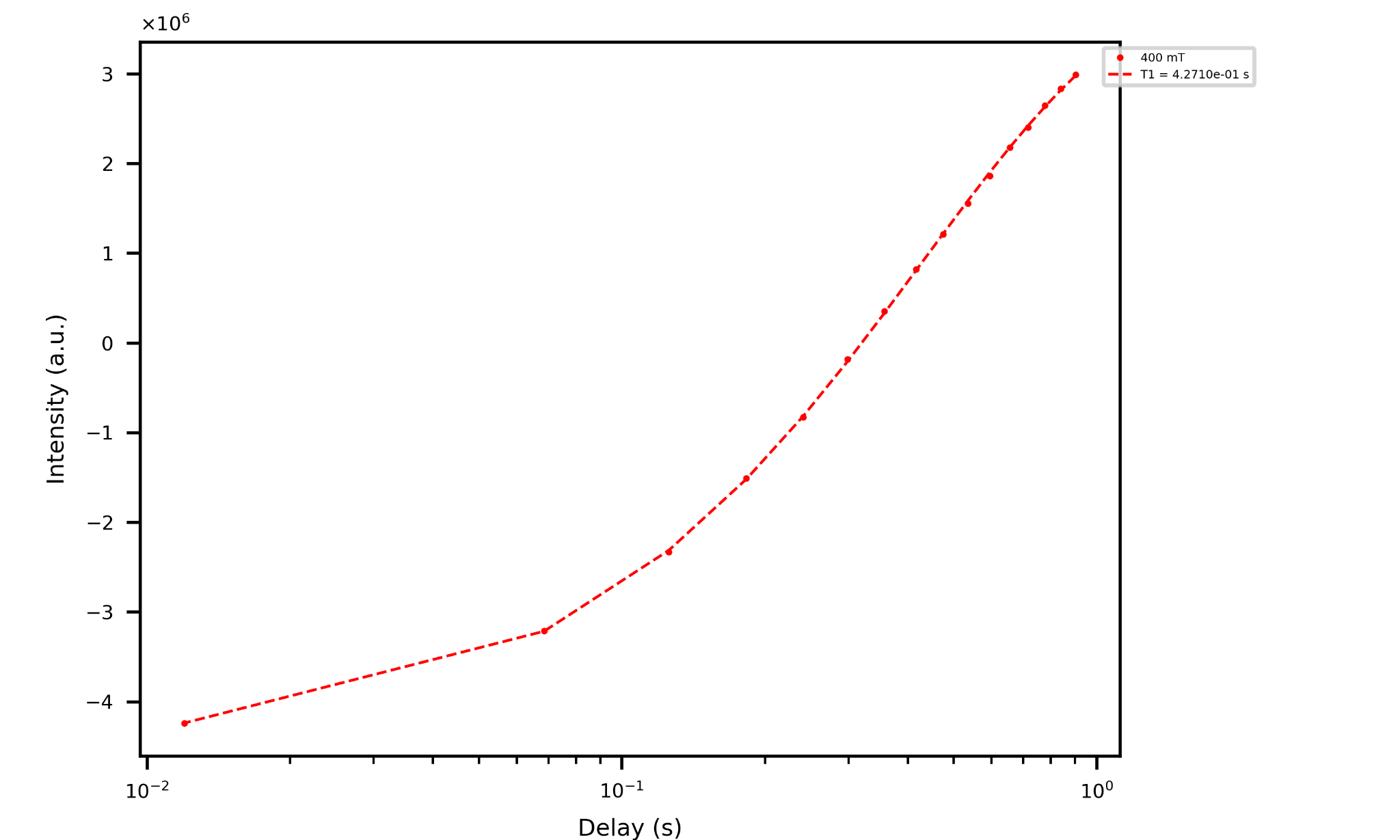 |

##

## S5.5. Input files for model_fit_pseudo2D

1^st^ Multiplet

INPUT1: inp_1

name ppm1 ppm2 v mult

true 1.54700 1.37200 1.43130 1

true 1.54700 1.37200 1.45813 1

INPUT2: inp_2

i ppm1 ppm2 k fwhm phi xg A B C D E

0 1.5 1.4 3.05e-03 1.95e-02 0.00e+00 2.00e-01 0.00e+00 0.00e+00 0.00e+00 0.00e+00 0.00e+00

1 1.5 1.4 3.27e-03 2.95e-02 0.00e+00 2.00e-01 0.00e+00 0.00e+00 0.00e+00 0.00e+00 0.00e+00

2^nd^ Multiplet

INPUT1: inp_1

name ppm1 ppm2 v mult

true 2.26900 2.09000 2.14332 1

true 2.26900 2.09000 2.15931 1

true 2.26900 2.09000 2.17149 1

true 2.26900 2.09000 2.18786 1

INPUT2: inp_2

i ppm1 ppm2 k fwhm phi xg A B C D E

0 2.3 2.1 2.19e-04 1.92e-02 0.00e+00 2.00e-01 0.00e+00 0.00e+00 0.00e+00 0.00e+00 0.000e+00

1 2.3 2.1 3.23e-04 1.34e-02 0.00e+00 2.00e-01 0.00e+00 0.00e+00 0.00e+00 0.000e+00 0.000e+00

2 2.3 2.1 3.91e-04 1.42e-02 0.00e+00 2.00e-01 0.00e+00 0.00e+00 0.00e+00 0.000e+00 0.000e+00

3 2.3 2.1 1.90e-04 1.74e-02 0.00e+00 2.00e-01 0.00e+00 0.00e+00 0.00e+00 0.00e+00 0.00e+00

## S5.6. Result folder of model fit pseudo2D function

| Table S5.6.1: Output files in olive_oil_experiment_modelfit folder, their description and an example for the 1^st^ multiplet - (1.372 - 1.547 ppm) and its first delay value. | |
| --- | --- |
| file name  [brief description] | example |
| 1.png  [vide Table S4.2] | 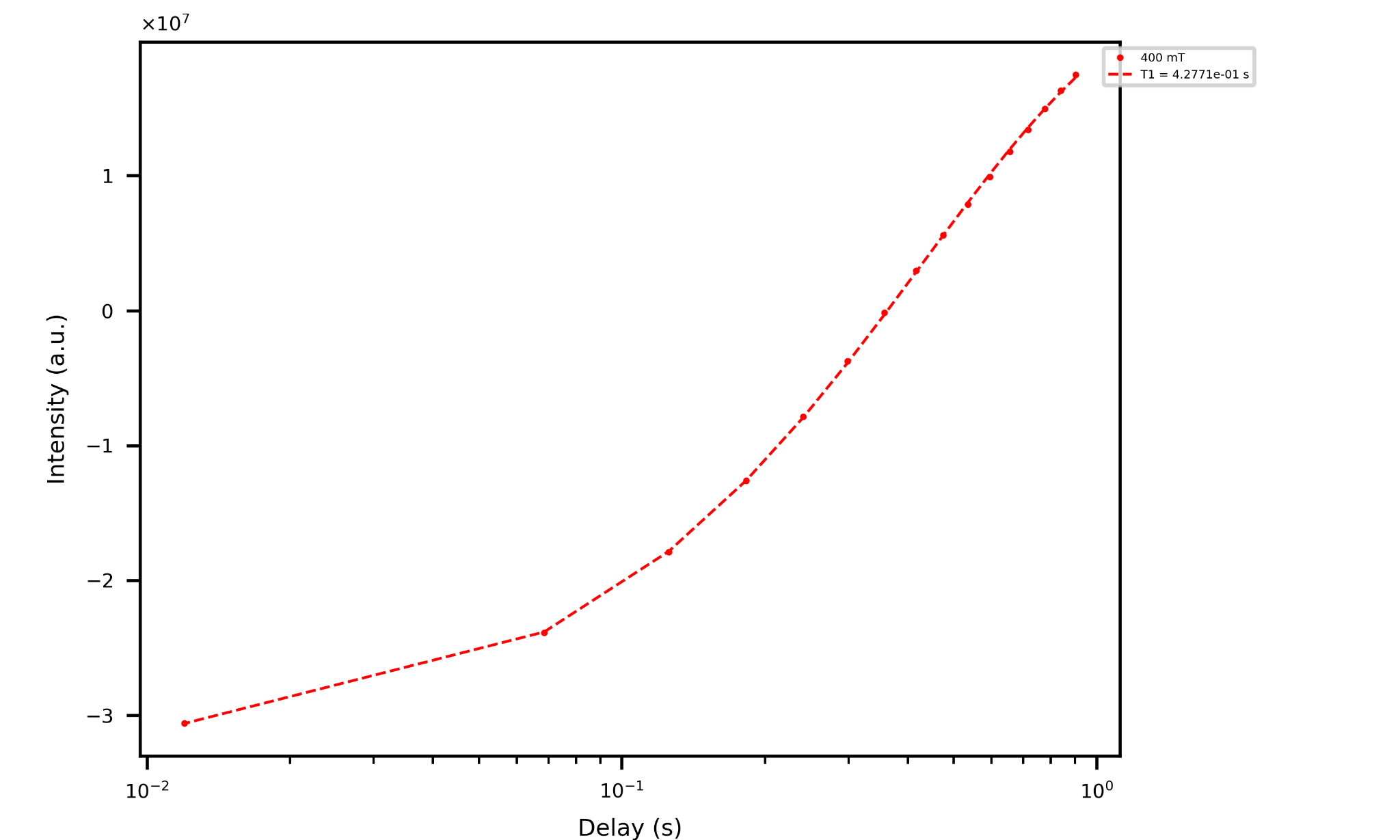 |
| Err_1  [vide Table S4.2] | 6.83E+05  6.57E+05  6.06E+05  5.54E+05  5.06E+05  4.90E+05  4.21E+05  3.31E+05  2.56E+05  0.00E+00  0.00E+00  7.03E+05  1.02E+06  1.43E+06  1.87E+06  2.36E+06 |
| input1_model  [Copy of 1^st^ input file containing information on selected range, position, type (true or false), number of peaks and their multiplicity (1-multiplet, 0-singlet)] | vide subsection S5.5 |
| input2_model  [Copy of 2^nd^ input file containing information on primal guesses on intensity k, linewidth fwhm, phase phi, gaussianity xg of each shape and its baseline correction coefficients A, B, C, D, E] | vide subsection S5.5 |
| R1_1.csv  [.csv files containing the obtained R_1_ values and their error for each field] | **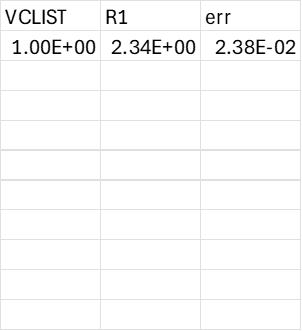** |
| R1_1.png  [Plot for R_1_ values with error bar vs. magnetic field strength In this case there is only one point since we are analyzing a single pseudo-2D] | **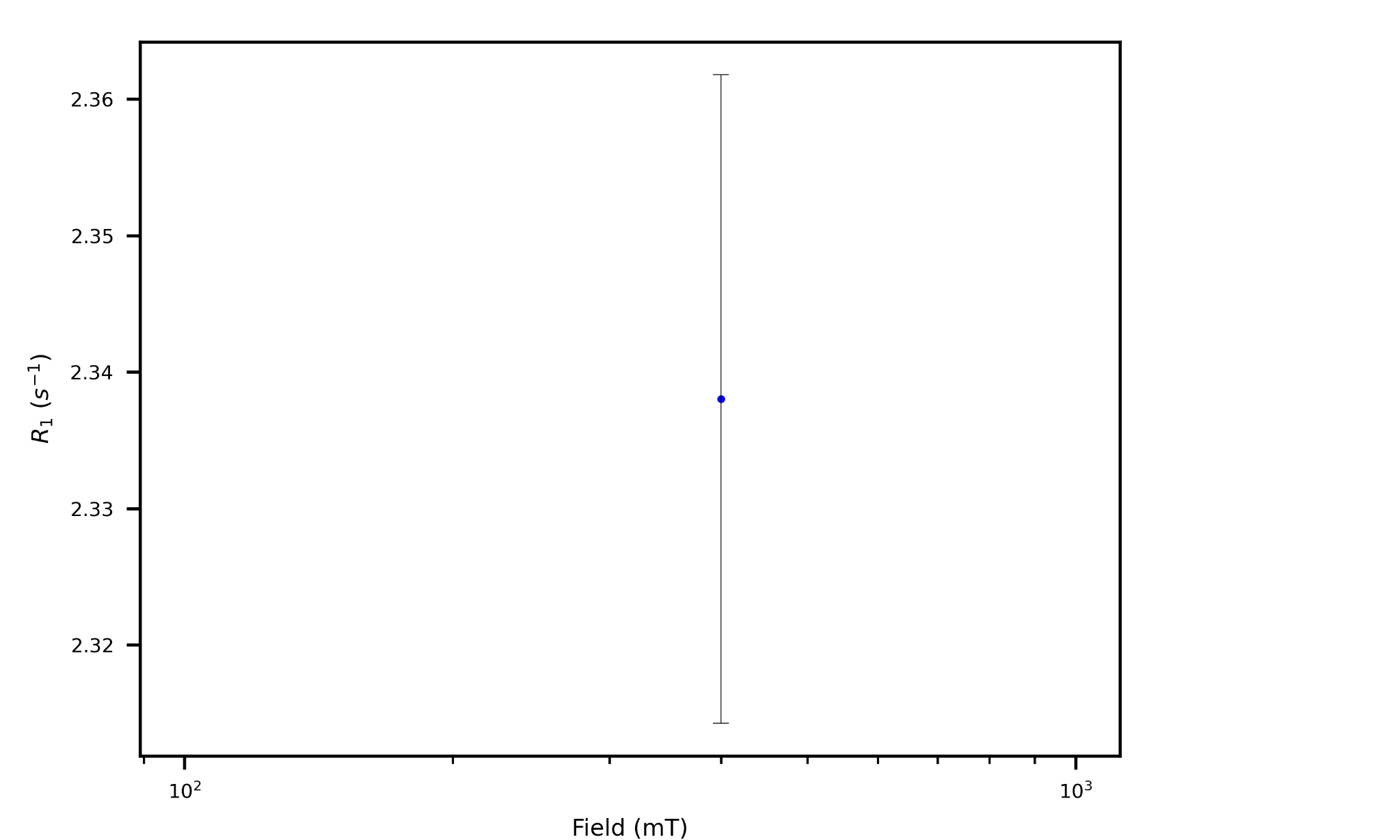** |
| VCLIST.txt  [List of values of magnetic fields [mT] taken from internal topspin file inside provided dataset (olive_oil_experiment/), or declared in main2.py] | 1.000e+00 |
| data_olio400_P1_I1.png  [Plot of original spectrum with fitted model , its baseline and residual line. The computed Gaussian functions are plotted with dotted lines. Figure generated for each delay.] | **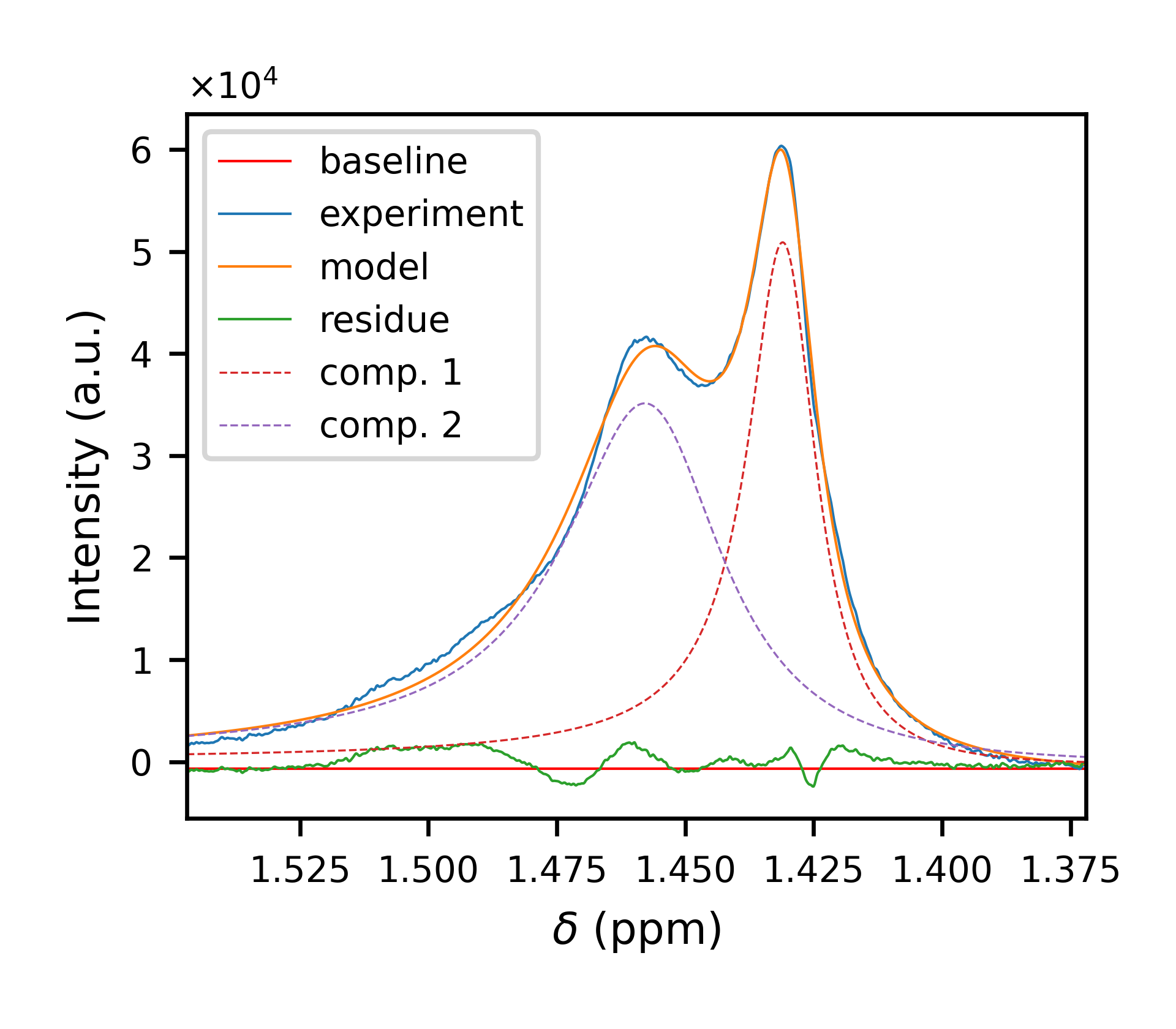** |
| x_1  [List of x-values, here the delays in seconds] | 0.90300  0.84067  0.77879  0.71734  0.65632  0.59573  0.53556  0.47580  0.41646  0.35751  0.29896  0.24081  0.18304  0.12565  0.06864  0.01200 |
| y_1  [List of y-values, here the intensity values for each delay] | 1.75E+07  1.63E+07  1.49E+07  1.34E+07  1.18E+07  9.92E+06  7.86E+06  5.60E+06  2.96E+06  -1.63E+05  -3.76E+06  -7.88E+06  -1.26E+07  -1.79E+07  -2.39E+07  -3.06E+07 |
| data_olio400_P1_I1_hist.png  [Residual histogram for given delay] | **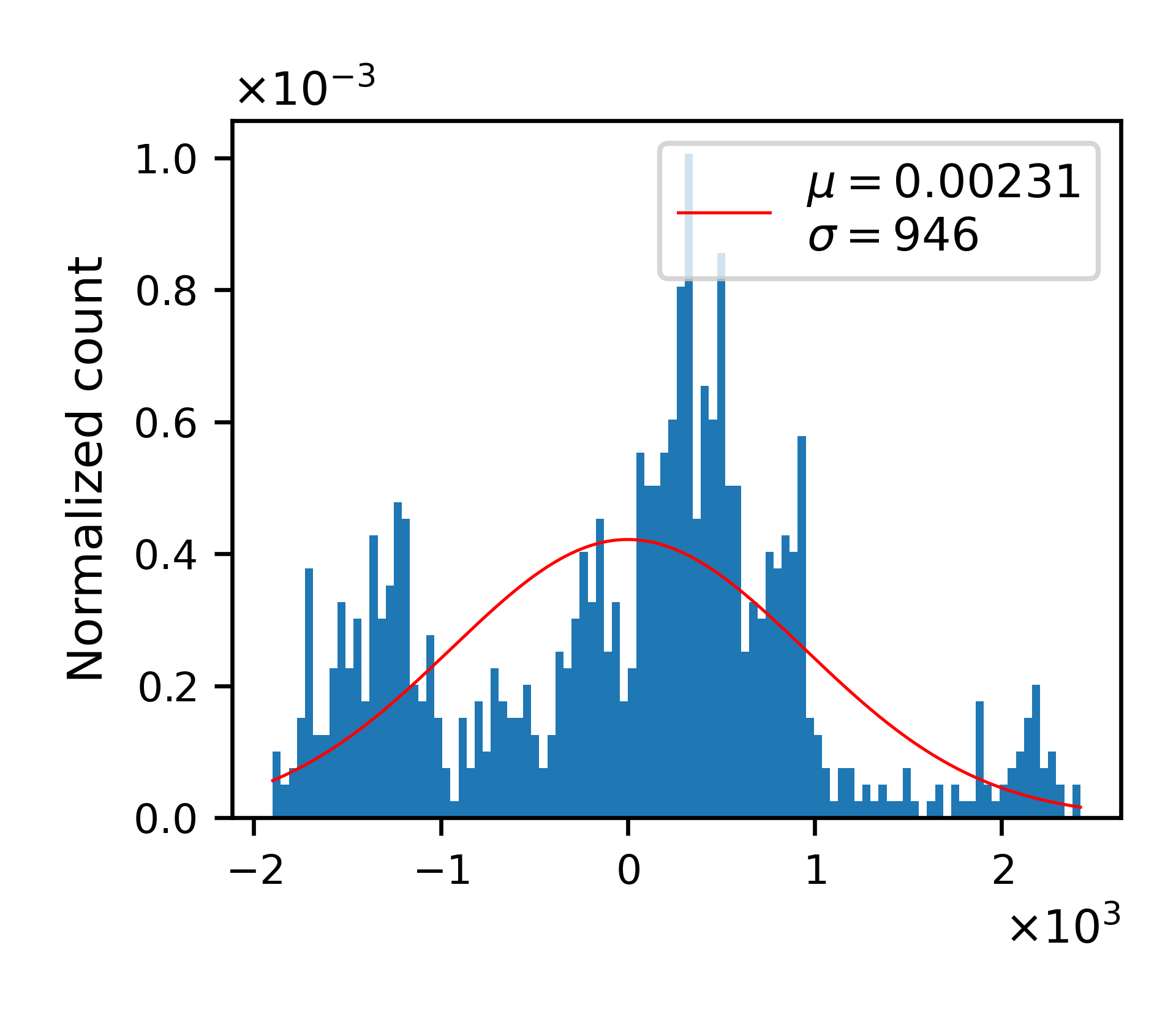** |
| data_olive_oil_experiment_sp4  [output file with combined list of used parameters, the obtained fit parameters and their error analysis] | vide subsection S5.7 |
| Peak_1.png  [Fitted plot of integral value (with error) vs. delay with linear x-axis.] | 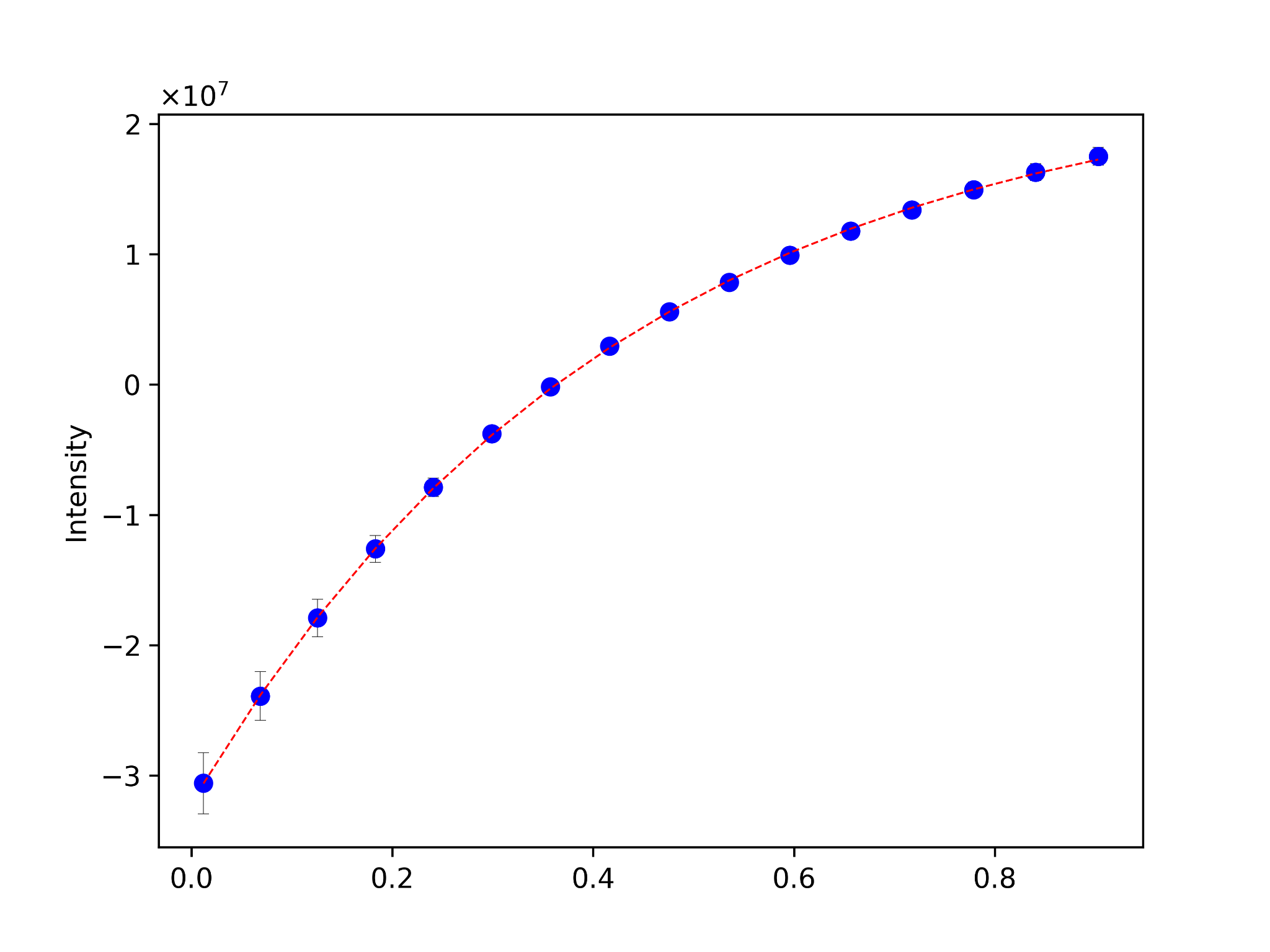 |
| data_olio400popt_sp0_I0_P0  [Internal system parameters] | {"unique_symbols": {"None": null, "gamfcn": {"__class__": "Callable", "__name__": "gamma", "pyversion": "3.12", "value": "gASVKwAAAAAAAACMHXNjaXB5LnNwZWNpYWwuX3NwZWNpYWxfdWZ1bmNzlIwFZ2FtbWGUk5Qu", "importer": "scipy.special._special_ufuncs"}, "E": 0, "B": 0.0, "ph_2": 0.15707963267464498, "ph_1": 0.14120627081893927, "True": 1, "erf": {"__class__": "Callable", "__name__": "erf", "pyversion": "3.12", "value": "gASVIQAAAAAAAACMFXNjaXB5LnNwZWNpYWwuX3VmdW5jc5SMA2VyZpSTlC4=", "importer": "scipy.special._ufuncs"}, "inf": Infinity, "shift_1": 1.4308439921816398, "little_endian": 1, "nan": NaN, "False": 0, "k_2": 0.0042232300050688565, "C": 0.0, "xg_2": 1.8503309995310246e-11, "lw_1": 0.015866509178052116, "D": 0.0, "pi": 3.141592653589793, "erfc": {"__class__": "Callable", "__name__": "erfc", "pyversion": "3.12", "value": "gASVIgAAAAAAAACMFXNjaXB5LnNwZWNpYWwuX3VmdW5jc5SMBGVyZmOUk5Qu", "importer": "scipy.special._ufuncs"}, "k_1": 0.0026366155709429684, "lw_2": 0.037120993711960745, "shift_2": 1.456744593684884, "xg_1": 1.5421386390102043e-11, "e": 2.718281828459045, "betalnfnc": {"__class__": "Callable", "__name__": "betaln", "pyversion": "3.12", "value": "gASVJAAAAAAAAACMFXNjaXB5LnNwZWNpYWwuX3VmdW5jc5SMBmJldGFsbpSTlC4=", "importer": "scipy.special._ufuncs"}, "A": -0.004422357622133183, "loggammafcn": {"__class__": "Callable", "__name__": "loggamma", "pyversion": "3.12", "value": "gASVLgAAAAAAAACMHXNjaXB5LnNwZWNpYWwuX3NwZWNpYWxfdWZ1bmNzlIwIbG9nZ2FtbWGUk5Qu", "importer": "scipy.special._special_ufuncs"}, "wofz": {"__class__": "Callable", "__name__": "wofz", "pyversion": "3.12", "value": "gASVIgAAAAAAAACMFXNjaXB5LnNwZWNpYWwuX3VmdW5jc5SMBHdvZnqUk5Qu", "importer": "scipy.special._ufuncs"}}, "params": [["shift_1", 1.4308439921816398, true, null, 0.4313, 2.4313000000000002, null, null, null, 1.4313, null], ["k_1", 0.0026366155709429684, true, null, -1, 1, null, null, null, 0.00304578, null], ["lw_1", 0.015866509178052116, true, null, 1e-08, 5, null, null, null, 0.0195096, null], ["ph_1", 0.14120627081893927, true, null, -0.15707963267948966, 0.15707963267948966, null, null, null, 0.0, null], ["xg_1", 1.5421386390102043e-11, true, null, 0, 1, null, null, null, 0.2, null], ["shift_2", 1.456744593684884, true, null, 0.4581299999999999, 2.4581299999999997, null, null, null, 1.45813, null], ["k_2", 0.0042232300050688565, true, null, -1, 1, null, null, null, 0.00327019, null], ["lw_2", 0.037120993711960745, true, null, 1e-08, 5, null, null, null, 0.0294886, null], ["ph_2", 0.15707963267464498, true, null, -0.15707963267948966, 0.15707963267948966, null, null, null, 0.0, null], ["xg_2", 1.8503309995310246e-11, true, null, 0, 1, null, null, null, 0.2, null], ["A", -0.004422357622133183, true, null, -Infinity, Infinity, null, null, null, 0.0, null], ["B", 0.0, false, null, -Infinity, Infinity, null, null, null, 0.0, null], ["C", 0.0, false, null, -Infinity, Infinity, null, null, null, 0.0, null], ["D", 0.0, false, null, -Infinity, Infinity, null, null, null, 0.0, null], ["E", 0, false, null, -Infinity, Infinity, null, null, null, 0, null]]} |

## S5.7. Output file for model_fit_pseudo2D - *data_olive_oil_experiment_sp4*

SPECTRA PATH:

C:path/to/TRAGICO_analysis/olive_oil_experiment/1_Field/pdata/1

CALIBRATION: (2.47000:2.30000) ppm

in points

0 0 -1 -1 -2 -4 -5 -5 -8 -12 -8 -12 -12 -13 -14 -15

in ppm

0.00000 0.00000 -0.00019 -0.00019 -0.00038 -0.00076 -0.00095 -0.00095 -0.00152 -0.00228 -0.00152 -0.00228 -0.00228 -0.00247 -0.00266 -0.00285

Points:

1 0.903

2 0.841

3 0.779

4 0.717

5 0.656

6 0.596

7 0.536

8 0.476

9 0.416

10 0.358

11 0.299

12 0.241

13 0.183

14 0.126

15 0.069

16 0.012

VCLIST point: 1.00 T

INPUT1: input1_2

n. peak name ppm1 ppm2 v mult

1 true 1.54700 1.37200 1.43130 1

1 true 1.54700 1.37200 1.45813 1

INPUT2: input2_2

n. peak i ppm1 ppm2 k fwhm phi xg A B C D E

1 0 1.5 1.4 3.05e-03 1.95e-02 0.00e+00 2.00e-01 0.00e+00 0.00e+00 0.00e+00 0.00e+00 0.00e+00

1 1 1.5 1.4 3.27e-03 2.95e-02 0.00e+00 2.00e-01 0.00e+00 0.00e+00 0.00e+00 0.00e+00 0.00e+00

===================================================================

===================================================================

(I: fit interval, P: N. point)

------------------------------

FIT RANGE: (1.55:1.37) ppm

I: 1 P: 1

------------------------------

Fit Report:

[[Fit Statistics]]

# fitting method = leastsq

# function evals = 1161

# data points = 920

# variables = 11

chi-square = 8.2386e+08

reduced chi-square = 906337.658

Akaike info crit = 12630.7275

Bayesian info crit = 12683.7957

## Warning: uncertainties could not be estimated:

xg_1: at boundary

ph_2: at boundary

xg_2: at boundary

[[Variables]]

shift_1: 1.43084399 (init = 1.4313)

k_1: 0.00263662 (init = 0.00304578)

lw_1: 0.01586651 (init = 0.0195096)

ph_1: 0.14120627 (init = 0)

xg_1: 1.5421e-11 (init = 0.2)

shift_2: 1.45674459 (init = 1.45813)

k_2: 0.00422323 (init = 0.00327019)

lw_2: 0.03712099 (init = 0.0294886)

ph_2: 0.15707963 (init = 0)

xg_2: 1.8503e-11 (init = 0.2)

A: -0.00442236 (init = 0)

B: 0 (fixed)

C: 0 (fixed)

D: 0 (fixed)

E: 0 (fixed)

n.peak Shift Integral

1 1.431 17503884.959 +/- 682532.714

------------------------------

FIT RANGE: (1.55:1.37) ppm

I: 1 P: 2

------------------------------

…

------------------------------

N. PEAK: 1

------------------------------

Fit Parameters:

t1=-0.3688489709098488 A=28124065.24482259 a=-4045457.3784022615

# S6. FDX2 longitudinal relaxation rates fit

## S6.1 Adapted target function for the regularization of IR intensity profiles in FDX fit

| def fit_IRprofile([function arguments]):  cal = np.abs(ppm_scale[0]-ppm_scale[1])+SR/sf1  cycle = -1  def f_residue(param, ppm_scale, data, tensor_red_list, delays, result=False):  nonlocal cycle  cycle += 1  print('fit peaks: '+f'{cycle:5g}',end='\r')  par = param.valuesdict()  integral_list_global = []  err_list_global = []  residuals_global = []  for idx in range(data.shape[0]):  lor_list = []  lor_ph0_list = []  comp_list = []  prev=0  spettro = data[idx,:].copy()  sim_spectra_tot = np.zeros_like(spettro, dtype='float64')  res_tot = []  res_tot_gradient = []  for ii in range(len(tensor_red_list)):    tensor_red = tensor_red_list[ii]  mult = tensor_red[:,-1]    lor_list.append([])  lor_ph0_list.append([])  comp_list.append([])  sx, dx, _ = f_fun.find_limits(tensor_red[0,1], tensor_red[0,2], ppm_scale)  sim_spectra = np.zeros_like(spettro, dtype='float64')    for jj in range(tensor_red.shape[0]):  lor = f_fun.t_voigt(t_aq, (par['shift_I'+str(ii+1)+'_V'+str(jj+1)+'_'+str(idx)]-cal-o1p)*sf1,  2*np.pi*par['lw_I'+str(ii+1)+'_V'+str(jj+1)+'_'+str(idx)]*sf1,  A=par['k_I'+str(ii+1)+'_V'+str(jj+1)+'_'+str(idx)],  phi=par['ph_I'+str(ii+1)+'_V'+str(jj+1)+'_'+str(idx)],  x_g=par['xg_I'+str(ii+1)+'_V'+str(jj+1)+'_'+str(idx)])  ### processing  lor *= f_fun.em(lor, LB, SW)  lor *= f_fun.qsin(lor, SSB)  ###  lor_ph0 = f_fun.t_voigt(t_aq, (par['shift_I'+str(ii+1)+'_V'+str(jj+1)+'_'+str(idx)]-cal-o1p)*sf1,  2*np.pi*par['lw_I'+str(ii+1)+'_V'+str(jj+1)+'_'+str(idx)]*sf1,  A=np.abs(par['k_I'+str(ii+1)+'_V'+str(jj+1)+'_'+str(idx)]),  phi=0, x_g=par['xg_I'+str(ii+1)+'_V'+str(jj+1)+'_'+str(idx)])  lor = f_fun.ft(lor, SI, dw, o1p, sf1)[0]  lor = np.conj(lor)[::-1].real  sim_spectra += lor  comp_list[ii].append(lor[sx:dx])  lor_ph0 = f_fun.ft(lor_ph0, SI, dw, o1p, sf1)[0]  lor_ph0 = np.conj(lor_ph0[::-1]).real  m=mult[jj]  if m==0:  prev=0  lor_list[ii].append(lor)  lor_ph0_list[ii].append(lor_ph0)  elif m!=0:  if m==prev:  lor_m = lor+lor_list[ii][-1]  lor_m_int = lor_ph0+lor_ph0_list[ii][-1]  del lor_list[ii][-1]  lor_list[ii].append(lor_m)  del lor_ph0_list[ii][-1]  lor_ph0_list[ii].append(lor_m_int)  else:  prev = m  lor_list[ii].append(lor)  lor_ph0_list[ii].append(lor_ph0_list)  sim_spectra_tot += sim_spectra  x = ppm_scale[sx:dx]  corr_baseline = par['E_'+str(idx)]*x**4 + par['D_'+str(idx)]*x**3 + par['C_'+str(idx)]*x**2 + par['B_'+str(idx)]*x + par['A_'+str(idx)]  if ii==0:  cost = np.sum((sim_spectra[sx:dx]+corr_baseline)*spettro[sx:dx].real)/np.sum((sim_spectra[sx:dx]+corr_baseline)**2)  lor_ph0_list[ii] = np.array(lor_ph0_list[ii])*np.abs(cost)  lor_list[ii] = np.array(lor_list[ii])*cost  comp_list[ii] = np.array(comp_list[ii])*cost  model = cost*(corr_baseline+sim_spectra[sx:dx])  res = model-spettro[sx:dx]  res_tot.append(res/max(spettro[sx:dx]))  res_tot_gradient.append((np.gradient(model)-np.gradient(spettro[sx:dx]))/max(np.gradient(spettro[sx:dx])))  [figures generation]    integral_in=[]  int_err = []  for ii in range(len(tensor_red_list)):  integral_in.append([])  int_err.append([])  tensor_red = tensor_red_list[ii]  sx, dx, _ = f_fun.find_limits(tensor_red[0,1], tensor_red[0,2], ppm_scale)  for jj in range(len(lor_list[ii])):  integral_in[ii].append(np.trapz(lor_list[ii][jj]))  Err = error_calc_num(ppm_scale, res_tot[ii]*max(spettro[sx:dx]), lor_ph0_list[ii][jj],  np.trapz(lor_ph0_list[ii][jj]), par['shift_I'+str(ii+1)+'_V'+str(jj+1)+'_'+str(idx)], sx, dx)  int_err[ii].append(Err)    integral_list_global.append(integral_in)  err_list_global.append(int_err)  residuals = []  for idxr,r in enumerate(res_tot):  residuals = np.concatenate([residuals, r])  residuals = np.concatenate([residuals, res_tot_gradient[idxr]])  x = ppm_scale  corr_baseline = par['E_'+str(idx)]*x**4 + par['D_'+str(idx)]*x**3 + par['C_'+str(idx)]*x**2 + par['B_'+str(idx)]*x + par['A_'+str(idx)]  model = cost*(corr_baseline+sim_spectra_tot)    [figures generation]  residuals_global.append(residuals)    integral_list_global = np.array([item for sublist in integral_list_global for item in sublist])  err_list_global = np.array([item for sublist in err_list_global for item in sublist])  #### inversion recovery fit  R1_list = []  res_tot = []  fmodel_list = []  for idx in range(integral_list_global.shape[1]):  res, fmodel = IR_residue(delays, 10**par['t1_'+str(idx+1)], integral_list_global[:,idx])  res_tot.append(res*10)  R1_list.append(1/10**par['t1_'+str(idx+1)])  fmodel_list.append(fmodel)  fmodel_list = np.array(fmodel_list)  res_IR = np.concatenate(res_tot)  residuals_global.append(res_IR)  [figures generation]  if not result:  return np.concatenate(residuals_global)  else:  return integral_list_global, err_list_global, R1_list    minner = lmfit.Minimizer(f_residue, param, fcn_args=(ppm_scale, data, tensor_red_list, delays))  result = minner.minimize(method='Nelder', max_nfev=10000)  params = result.params  result = minner.minimize(params=params, method='leastsq', max_nfev=30000)    popt = result.params  Int, Err, R1 = f_residue(popt, ppm_scale, data, tensor_red_list, delays, result=True)    return Int, Err, R1 |
| --- |

## S6.2 IR intensity profiles from TRAGICO

| Figure S6.2.1: Intensity profiles of ^13^C peaks derived from the fit of the HNS spectra. The profiles are ordered from 1 to 8 (see figure 2 in the main text). | |
| --- | --- |
| 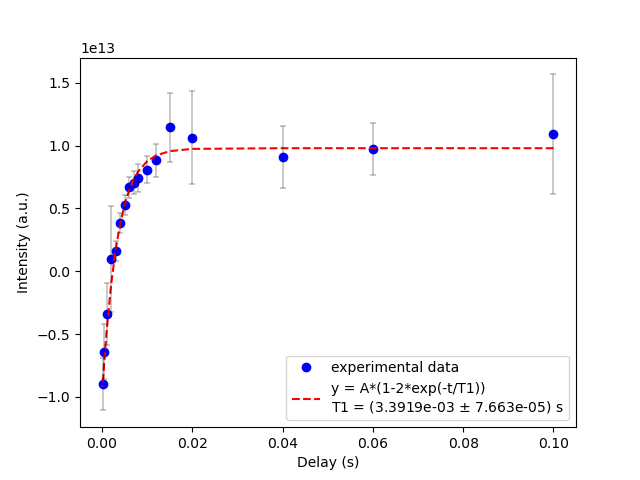 | 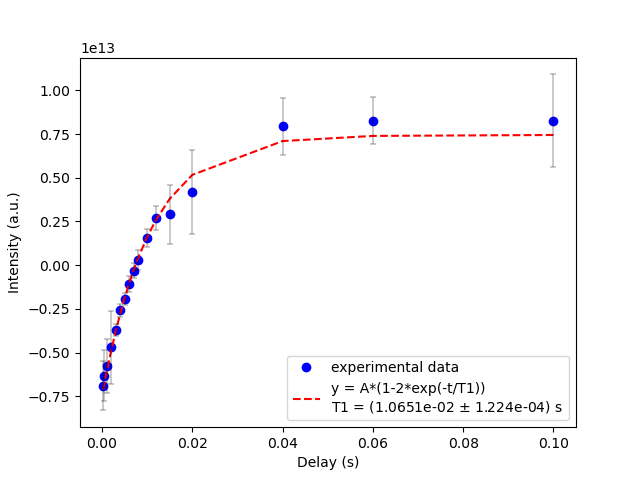 |
| 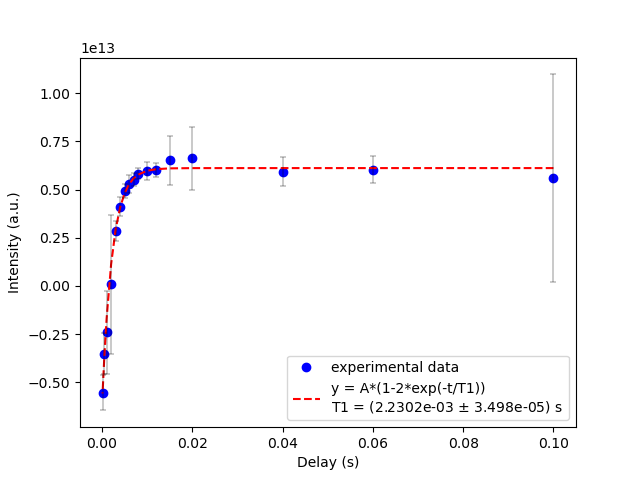 | 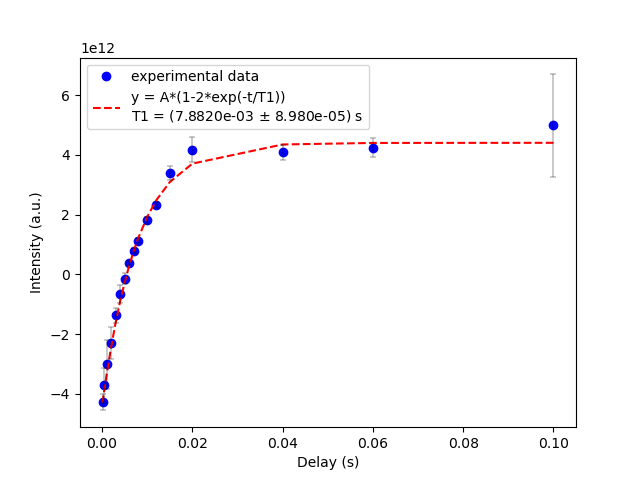 |
| 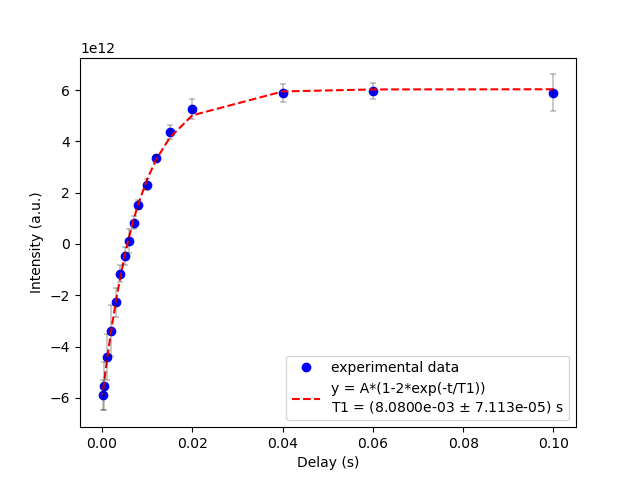 | 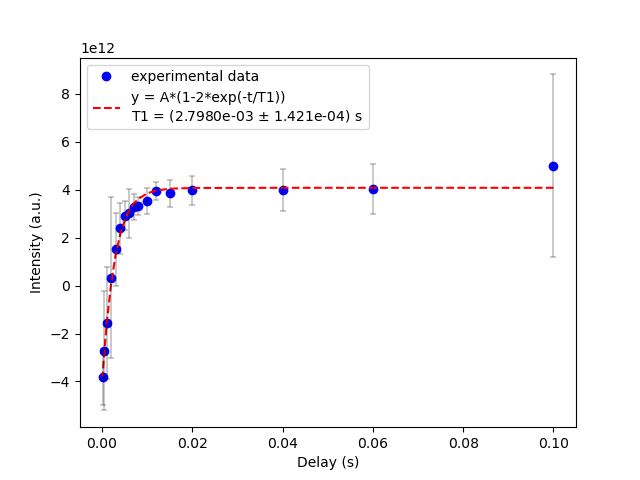 |
| 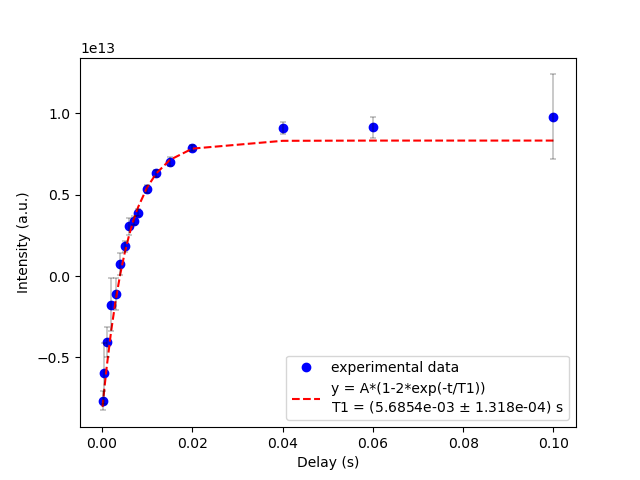 | 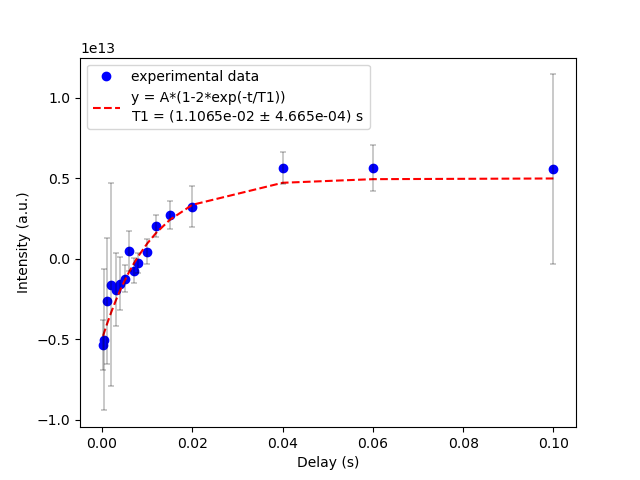 |

| Figure S6.2.2: Intensity profiles of ^13^C peaks derived from the fit of the LNS spectra. The profiles are ordered from 1 to 8 (see figure 2 in the main text). | |
| --- | --- |
|  |  |
|  |  |
|  |  |
|  |  |

## S6.3 IR intensity profiles from Topspin

| Figure S6.3.1: Intensity profiles of ^13^C peaks derived with Topspin of the HNS spectra. The profiles are ordered from 1 to 8 (see figure 2 in the main text). | |
| --- | --- |
|  |  |
|  |  |
|  |  |
|  |  |

| Figure S6.3.2: Intensity profiles of ^13^C peaks derived with Topspin of the LNS spectra. The profiles are ordered from 1 to 8 (see figure 2 in the main text). | |
| --- | --- |
|  |  |
|  |  |
|  |  |
|  |  |

## S6.4 T_1_-encoded ^13^C FDX2 spectra

|  |
| --- |
| Figure S6.4.1: Comparison of spectra acquired at different delays during the IR experiment, for the HNS dataset. |

# S7. Analysis of relaxation measurements at variable magnetic field

## S7.1 Code listing for intensity analysis

| from f_fit import *  import numpy as np  # folder containing the spectra  path = 'path/to/TRAGICO_analysis/GdCl3_EtGl_experiment/'  list_path = [int(f) for f in os.listdir(path) if not f.startswith('.')]  list_path = [str(f) for f in np.sort(list_path)][2:]  # list of delays  delays_list = [np.loadtxt(path+'/'+list_path[i]+'/vdlist')+3e-3 for i in range(len(list_path))]  # intensity fit  intensity_fit_pseudo2D(  path,  delays_list,  list_path,  prev_lims = True,  prev_coeff = True,  area = False, # True- Integration, False - Intensity  VCLIST = None,  cal_lim = (3.8460, 3.0150),  baseline = True,  doexp = True,  f_int_fit = None) |
| --- |

## S7.2 Code listing for spectra modeling

| from f_fit import *  import numpy as np  path = 'path/to/TRAGICO_analysis/GdCl3_EtGl_experiment/'  list_path = [int(f) for f in os.listdir(path) if not f.startswith('.')]  list_path = [str(f) for f in np.sort(list_path)][2:]  print(list_path)  delays_list = [np.loadtxt(path+list_path[i]+'/vdlist')+3e-3 for i in range(len(list_path))]  lim1 = {'shift':(-0.05,0.05), 'lw':(1e-4,0.05), 'ph':(-np.pi/20,np.pi/20),  'xg':(0.001,1), 'k':(0,1), 'C':(0,0), 'D':(0,0), 'E':(0,0)}  lim2 = {'shift':(0.9,1.1), 'lw':(0.9,1.1), 'ph':(0,0), 'xg':(0.9,1.10)}  model_fit_pseudo2D(  path,  delays_list,  list_path,  cal_lim = (3.8460, 3.0150),  fast = True,  dofit = True,  prev_fit = None,  limits1 = lim1,  limits2 = lim2,  prev_guess = True,  L1R = None,  L2R = None,  doexp=True,  f_int_fit=None) |
| --- |
